# Mechanical Taming of Hardy–Cope Rearrangements

**DOI:** 10.1021/acscentsci.6c00254

**Published:** 2026-05-07

**Authors:** Matthew J. Elardo, Mariia Kuznetsova, Jason D. Kaff, Kiyoshi J. Colon, Gregory L. Olsen, Paul R. McGonigal, Matthew R. Golder

**Affiliations:** ∥ Department of Chemistry and Molecular Engineering and Science Institute, 7284 University of Washington, Seattle, Washington 98195, United States; ‡ Department of Chemistry, 6396 Oxford University, Oxford OX1 3TA, United Kingdom; § Department of Chemistry, University of York, York YO10 5DD, United Kingdom

## Abstract

Bullvalene is a fluxional molecule that rapidly rearranges via low-barrier [3,3] sigmatropic rearrangements, allowing each of its 10 carbon atoms to exchange rapidly at room temperature, accessing all possible configurational permutations with submillisecond half-lives. We previously demonstrated that the incorporation of this fluxional core into polymeric systems imbues materials with force-responsive properties, as bullvalene’s fluxionality can reversibly “respond” to applied mechanical stress. However, direct observation of such rearrangements within the bullvalene core in response to applied force has remained elusive in soft materials because of the complexity of the resulting isomer distributions and the rapid thermal reversion of perturbed isomers. In this work, we utilize a polymer-chain-centered fluorinated bullvalene core to provide direct spectroscopic evidence of changes to the bullvalene isomer distribution in response to mechanical force. These findings position bullvalene as the first example in a unique class of mechanophores capable of undergoing *multiple* mechanochemical reactions without requiring an external stimulus for subsequent mechanochemical activity. We anticipate these results to provide key insights into the development of force-responsive materials with enhanced mechanical properties and macromolecular sensing capabilities.

## Graphical abstract

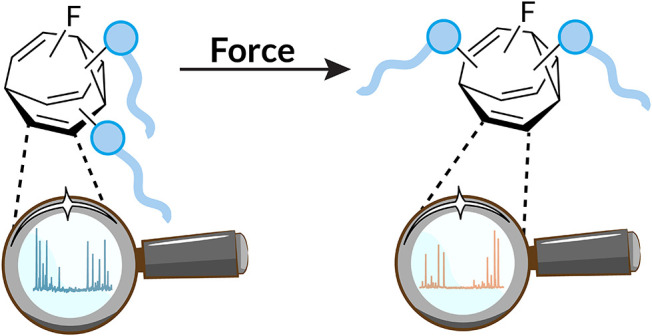

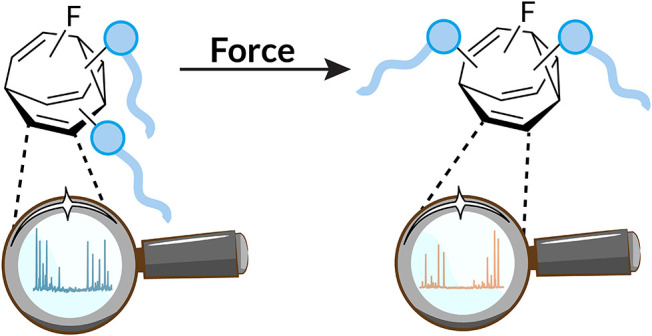

## Introduction

Early work from Staudinger demonstrated that mechanical force induces reactivity in large macromolecules via nonspecific backbone scission, lowering the molecular weight of a given polymer chain.^1^ It was later shown that polymer degradation was caused by homolytic bond cleavage to produce macroradicals in response to applied force, demonstrating direct cleavage of covalent bonds via applied mechanical force.^2,3^ The study of this phenomena (i.e., how chemical reactivity is influenced by mechanical forces) is known as *mechanochemistry*.^4^ While early studies in mechanochemistry focused on mechanically induced destructive events such as chain scission, in recent years, attention has shifted toward mechanically facilitated chemical transformations in a more productive manner.^5^ Specifically, recent advances in polymer mechanochemistry led to the development of force-responsive materials, often through the incorporation of molecular units known as *mechanophores*, or chemical units which respond chemoselectively to applied force.^5^ Mechanophores have subsequently found an array of uses, ranging from colorimetric damage sensing,^6^ stress relief via the release of stored length,^7^ and cargo-release for the delivery of small molecule payloads.^8^ Additionally, mechanophores have been designed to probe fundamental questions governing polymer mechanochemistry, for instance exploring the role of torsional forces and attachment geometry on force transduction^9–11^ in soft materials.

Mechanophores have been shown to influence reactivity by perturbing potential energy landscapes, unlocking avenues to reactivity that are thermodynamically or kinetically inaccessible under the influence of conventional stimuli (e.g., light, heat).^12–14^ Conventional mechanophores undergo covalent bond scission upon applied force, leading to reactions that are either irreversible or require external stimuli (e.g., irradiation with UV light) to induce reversibility. On the other hand, bullvalene (C_10_H_10_, Figure 1A) theoretically offers a platform upon which to develop a new class of *fully reversible* “fluxional” mechanophores.

**Figure 1:**
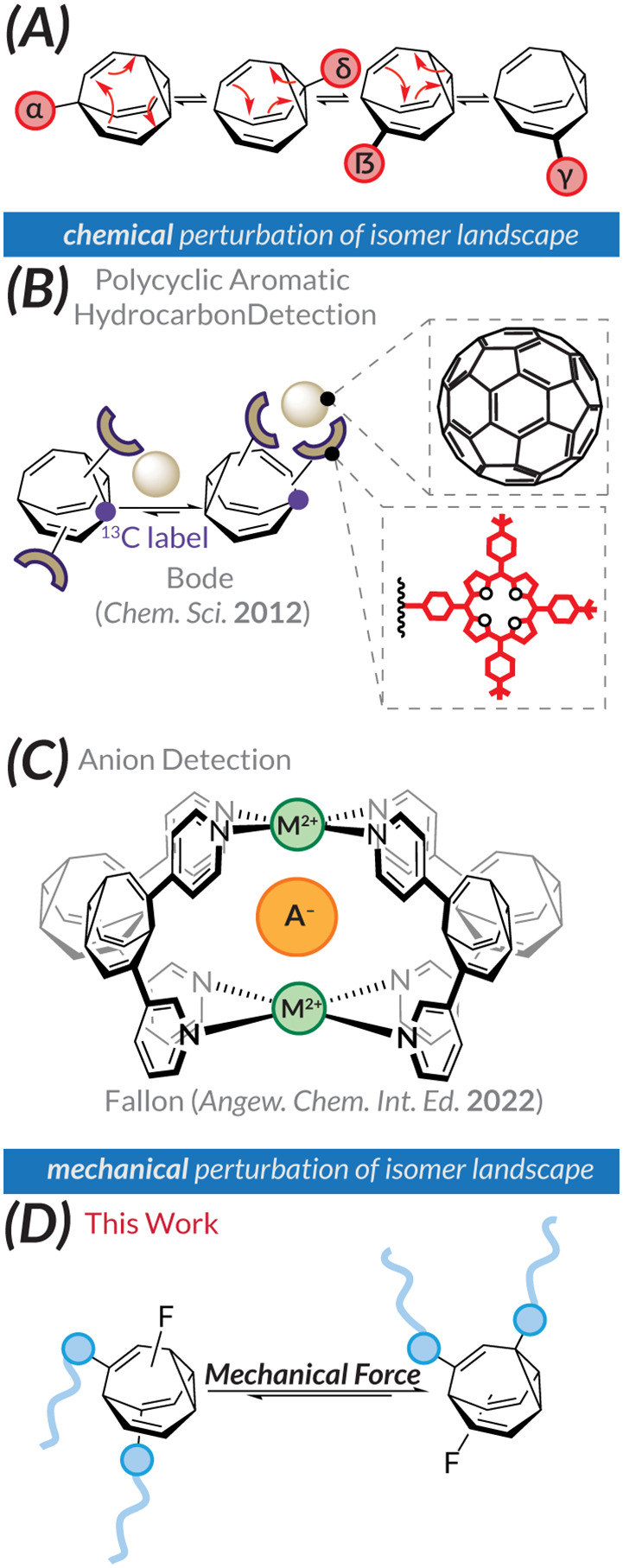
(A) Bullvalene samples all possible valence isomers through low-barrier Hardy–Cope rearrangements. (B, C) Previous studies utilize host–guest chemistry to *chemically* perturb bullvalene isomer distributions. (D) This work: bullvalene isomer distributions are perturbed by *mechanical force* in bullvalene-containing macromolecules.

Bullvalene is a tricyclic hydrocarbon cage which undergoes rapid Hardy–Cope rearrangements with a remarkably low activation barrier (*E*_a_ = 13.1 kcal/mol),^15–18^ allowing the bullvalene core to rapidly rearrange between over 1.2 million degenerate isomers at room temperature (*k* ≈ 4000 s^–1^ at 21 °C).^19^ Substituted bullvalenes exist as a complex mixture of isomers, wherein the Hardy–Cope rearrangement processes cause each carbon to interchange even at low temperatures; substituents may access each of the possible α, β, γ, and δ positions on the bullvalene core (Figure 1A).

We previously hypothesized that the incorporation of bullvalene into polymeric systems would modulate the thermomechanical properties of these systems by imbuing them with dynamic behavior. Indeed, previous results from our lab demonstrated that bullvalene incorporation into π-rich thermoplastics lowers their glass transition temperatures (*T*_g_) by conferring them with increased structural flexibility.^20^ We also previously demonstrated that the incorporation of bullvalene as a cross-linker in thermosetting polymer networks leads to glassy materials with increased hysteresis energies, higher Young’s moduli, and lower fragility indices.^21,22^ In addition to these polymeric materials, bullvalene linkers have been included in the structure of small-molecule organic glasses.^23^ We attributed such changes in bulk polymer properties to the fluxional nature of the bullvalene core. However, directly observing discrete changes to macromolecular bullvalene isomer distributions in response to mechanical force remained elusive. Central to this challenge in directly assessing changes to isomer distributions in bullvalene-containing polymers are (1) the small magnitude of structural changes induced by bullvalene rearrangements and (2) the resulting complexity of NMR spectra when the bullvalene unit is only a small fraction of the overall polymer mass. Previous work by Bode addressed similar challenges in molecular sensors by utilizing a ^13^C-labeled bullvalene core; ^13^C NMR analysis after introducing analyte revealed perturbed isomer populations stemming from bullvalene guest–host interactions (Figure 1B).^24,25^ Similarly, work by Fallon showed that the isomer distribution of pyridyl-substituted bullvalenes within Pd^2+^ or Pt^2+^ metal–organic cages converge to a single isomer in the presence of certain anions (Figure 1C).^26^ These works collectively demonstrate that the bullvalene isomer distribution can be perturbed by chemical means, and that the subsequent isomer analyses can be simplified with heteronuclear labels. Inspired by these findings, we sought to synthesize ^19^F-substituted bullvalenes to facilitate the analysis of bullvalene isomer distributions, even with very low bullvalene loadings in polymer systems. Notably, the incorporation of ^19^F substitution to the bullvalene core allows for the direct observation of bullvalene isomers via ^19^F NMR spectroscopy without interference from nuclei along a hydrocarbon polymer backbone. Additionally, the large spectral width of the ^19^F nucleus allows structurally similar bullvalene isomers to remain well-resolved in the ^19^F NMR spectrum based on the position of the fluorine substituent along the bullvalene core. We therefore hypothesized that by applying mechanical force to bullvalene-containing polymers and comparing the “force-perturbed” ^19^F NMR spectrum to an “equilibrium” (i.e., force-free) spectrum, we could directly sense the response of the bullvalene core to mechanical force (Figure 1D).

## Synthesis and Early Attempts at Probing Force-Induced Rearrangements

Our efforts toward a fluorinated bullvalene-centered polymer, akin to conventional chain-centered mechanophore^6^ approaches, commenced with the synthesis of fluorinated bullvalene bis­(propargyl) ether (**F-Bull-PE**). To begin, **COT** is brominated under classical conditions (Br_2_ followed by elimination with KO*t*Bu, 85% yield); the resulting **Br-COT** is then converted to the necessary **F-COT** under newly optimized Grignard conditions in 55% yield (). With the fluorine label introduced during the early stages of the synthesis, a formal cobalt-catalyzed [6 + 2] cycloaddition reaction of **F-COT** with **TBDMSO-Alkyne**,^27,28^ followed by subsequent silyl ether deprotection with TBAF, affords requisite **F-CA-Diol** in 35% yield. The penultimate bullvalene intermediate, **F-Bull-PE**, is then accessed by propargylation of **F-CA-Diol** (NaH and propargyl bromide, ≥95% yield)^29^ and subsequent photosensitized di-π-methane rearrangement (40% yield).^30–32^ Finally, copper-catalyzed azide–alkyne cycloaddition (CuAAC) reaction (utilizing PMDETA as the accelerating ligand and sodium ascorbate to regenerate Cu^I^) with **ATRP-Azide** delivers the target compound **F-Bull-ATRP** in ca. 2.5% overall yield from **COT**. This bifunctional initiator provides a means by which polymer chains can be grafted on both termini of the fluorinated bullvalene core via atom-transfer radical polymerization (ATRP) to deliver bullvalene-centered polymers (Scheme 1). This system provides two key features we deemed advantageous to our proposed analyses: (1) each polymer chain contains only one bullvalene molecule with a single ^19^F label, such that each signal in the resulting ^19^F spectrum represents a particular bullvalene isomer within an individual polymer chain, and (2) the location of the bullvalene unit in the center of the macromolecule maximizes the chance of activating force-induced rearrangements, as the stresses experienced by chemical bonds in polymers undergoing chain extension in dilute solution are known to be greatest at the center of the polymer chains.^33^

**Scheme 1:**
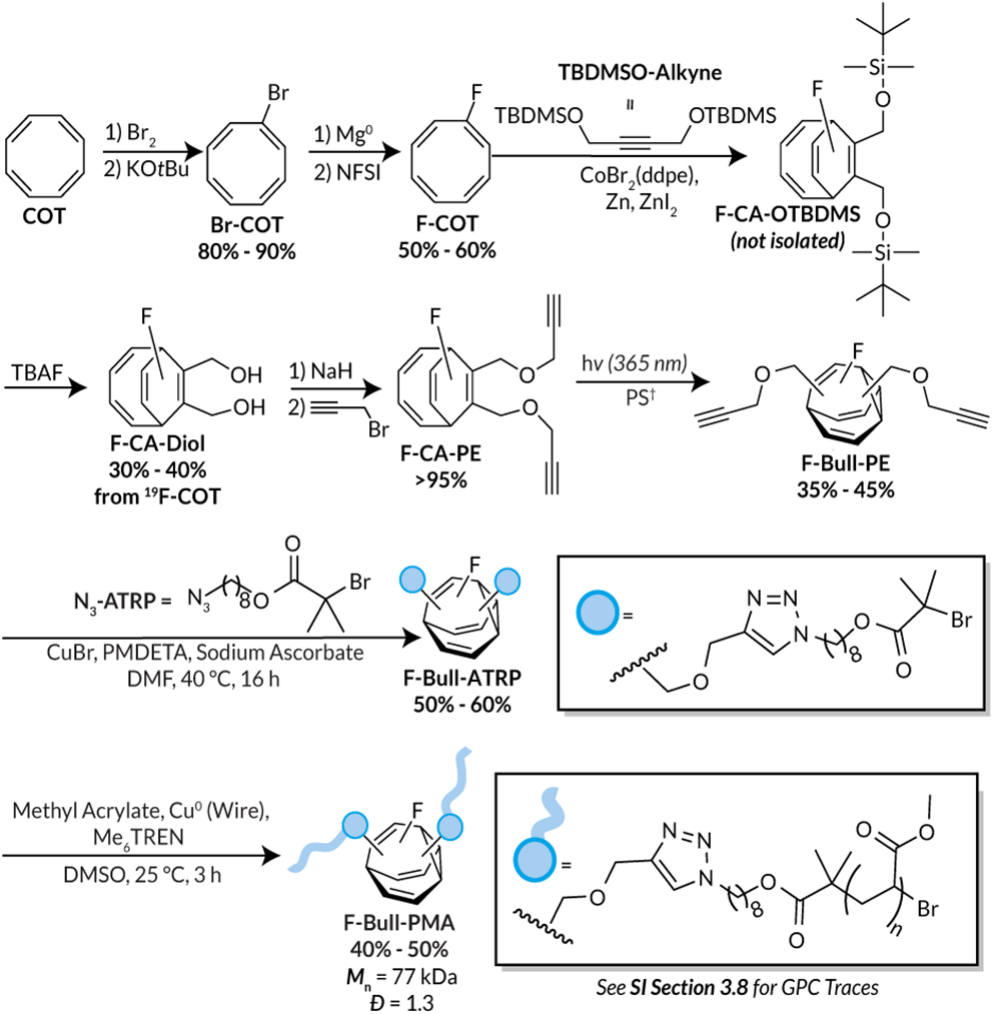
Synthesis of **F-Bull-PMA**

a PS = photosensitizer (thioxanthen-9-one).

With our synthesis optimized and bifunctional initiator in hand, we set out to investigate the network dynamics^27,34,35^ of this trisubstituted bullvalene system. We reasoned that any small differences in polymer backbone lengths following grafting would lead to insignificant differences in the corresponding ^19^F NMR spectra. Thus, we chose to model our system as an A,A,B substituted bullvalene, with the “A” units, modeled as methoxymethyl groups, representing the polymer chains and the “B” unit representing the fluorine label (**F-BullMeOMe**, Figure 2). Bullvalenes with such a substitution pattern exist as a mixture of 120 possible isomers, producing complex isomer networks which may be modeled computationally using our computational tool *bullviso* (Figure 2A; see for details).^36,37^ For each of the 120 possible constitutional isomers, we generated and optimized the geometries of 10 conformers at the at the PBE0-D3/def2-SV­(P) level of theory^38–53^ (i.e., 1200 structures in total). The lowest energy structure has one substituent on each alkene in a β-position. Several constitutional isomers are accessible within a few kcal·mol^–1^ (), giving rise to a mixture with ca. 20 isomers present at relative concentrations of ≥1% in equilibrium ().

**Figure 2:**
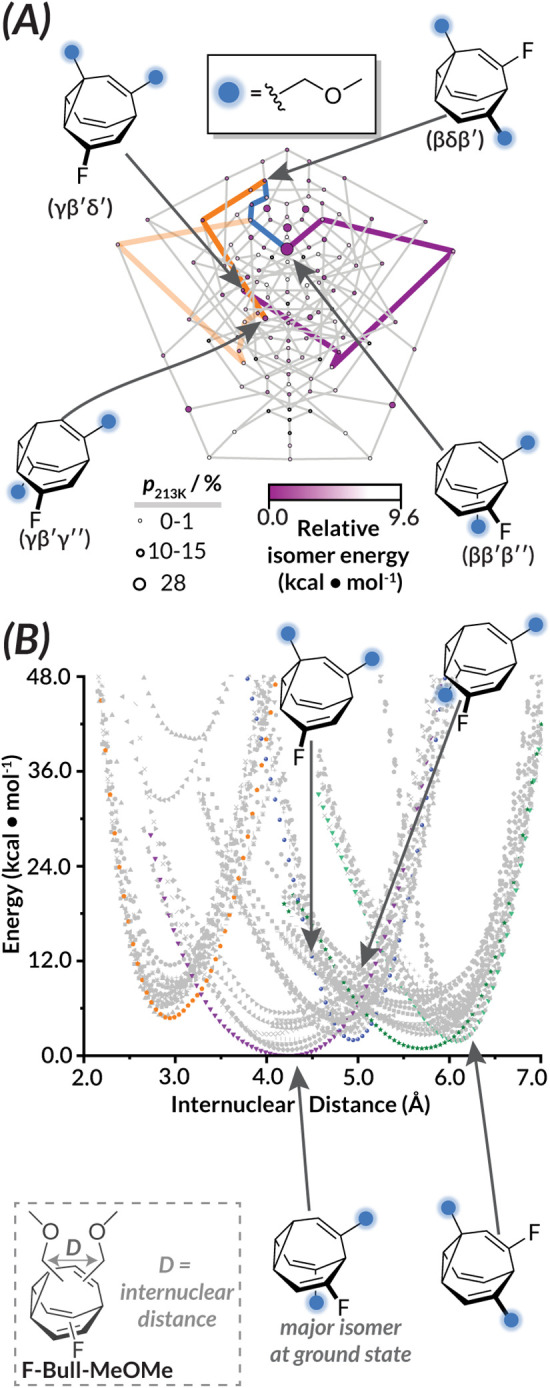
(A) Isomer network for the bullvalene system studied in this paper. Bolded lines indicate the shortest paths between depicted isomers. (B) Mechanical force perturbs the bullvalene isomer network potential energy surface via elongation of the internuclear distance between substituents.

To simulate the effects of stretching or compressing the structures present at thermal equilibrium, we performed relaxed potential energy surface scans as the distance between the two methylene groups connected to the bullvalene scaffold is expanded or compressed in 0.05 Å increments (see for details). We found that the energy content of each particular bullvalene isomer changes differentially in response to this “external force”. Each parabola in Figure 2B represents a different bullvalene isomer, where the width of the parabola is related to how sensitive a particular isomer is to geometric distortions. There are clear groupings, or “families”, of curves that result from this analysis, where each family of curves represents bullvalene isomers where the pendant chains are in the same relative positions and the location of the fluorine substituent is varied. The results for each unique bullvalene isomer are depicted as individual curves in .

Within each isomer family, the most stable isomer is depicted with a colored curve, and its structure is reproduced on the graph and its location in the network diagram in Figure 2A is indicated. For instance, isomer **ββ′β″** is predominant absent applied force, whereas isomers **γβ′δ′**, **γβ′γ″**, and **βδβ′** are predominant at increasing levels of mechanical stress. Thus, unlike typical mechanophores, mechanical perturbation has the potential to initiate a series of pericyclic reactions. For example, a minimum of four rearrangements connects isomers **ββ′β″** and **βδβ′** in the reaction network (Figure 2A). Only small deviations from equilibrium geometries (ca. 1.0–2.0 Å) are required to substantially alter the thermodynamic landscape for the isomer network and induce multiple sequential Hardy–Cope rearrangements. These values are well within the range of chain extension accessible by ultrasonication; CoGEF results in prior studies indicate that perturbations from equilibrium of ca. 2–3 Å ^54,55^ and up to ca. 10 Å^9^ are required to induce the observed mechanochemical reactions for some systems. We therefore concluded that the bullvalene isomer distribution should be significantly perturbed due to the applied mechanical stresses produced during sonication, encouraging us to continue our analysis in an attempt to verify the results experimentally. Importantly, early control experiments indicated that ultrasonication of bullvalene-containing polymers (*vide infra*) led to no degradation to the bullvalene core, even as the polymer chains underwent nonspecific backbone cleavage (i.e., chains were indeed experiencing mechanical stress; ).

With computational data supporting our initial hypothesis and encouraged by the fact that the bullvalene core itself did not decompose during ultrasonication, we set out to search for changes to the resulting isomer distributions after applying force to our system. Due to the inherent reversibility of bullvalene rearrangements, we required a method of “freezing” Hardy-Cope rearrangement processes to enable measurement of the perturbed isomer distributions without complications from thermal re-equilibration. For example, at 21 °C, individual fluorobullvalene isomers have half-lives of ca. 0.5–5.5 ms,^56^ making direct observation experimentally challenging on such a short time scale. To overcome such complications, we first attempted to saturate the bullvalene π bonds via a mild diimide reduction, a process which we and others have previously demonstrated affords static products.^20,57^ We were particularly attracted to this method because unlike heterogeneous reductions, which we anticipated to be operationally complicated to perform concomitantly with ultrasonication, diimide reductions are homogeneous upon addition of a sulfonylhydrazide, (e.g., *o*-nitrobenzenesulfonylhydrazide, NBSH),^58^ to a bullvalene solution.^20,57^ We thus reasoned we could attempt to reduce the bullvalene core during sonication, perform a similar experiment under force-free conditions (i.e., in a standard solution state system with magnetic stirring), and compare the resulting ^19^F NMR spectra to assess the impact of ultrasonication on isomer distribution (Figure 3A).

**Figure 3:**
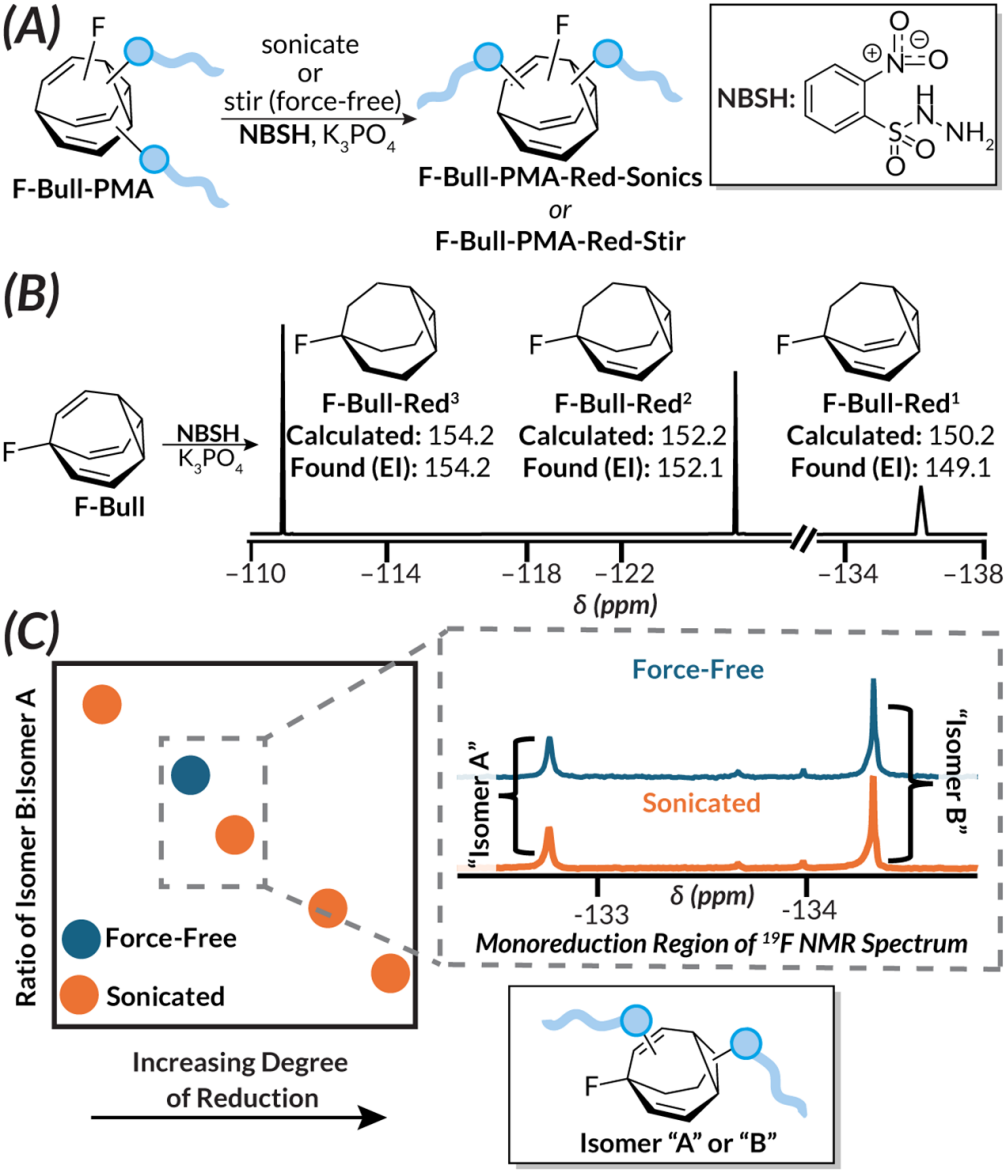
(A) Reaction scheme for bullvalene reduction. (B) Reduction of **F-Bull** as a small molecule model system revealed partial reduction products as the major products following bullvalene reduction. (C) Isomer ratios in polymeric samples correlated strongly with the extent of reduction, with similar results for sonicated samples and force-free-controls. EI = Electron impact ionization mass spectrometry. Calculated and found mass-to-charge ratios are given.

Unfortunately, this approach was hindered by two significant limitations. First, only partial reduction was observed, with leftover bullvalene signals present at −86.0 to −87.0 ppm (). Second, a comparison of these resulting NMR spectra to those of reduced fluorobullvalene () indicated that the majority of our reduced species had fluorine in the bridgehead position; major ^19^F NMR signals that we observed were arising from mixtures of mono-, di-, and trireduced species (Figure 3B; see for more details on the small molecule model and peak assignments). This finding indicated that the reduction conditions were too mild to reduce the electron-poor vinyl fluorides in our bullvalene system, a major limitation considering that >50% of the ground-state bullvalene isomers in **F-Bull-PMA** feature vinyl fluoride connectivity (). Despite this limitation, we reasoned that it still might be possible to evaluate differences in the isomer distributions by focusing on specific regions of the ^19^F NMR spectra by limiting our scope to isomers in monoreduced species with fluorine at the bridgehead position. Unfortunately, we observed significant variability in the resulting isomer distributions within a single experimental treatment based solely on the amount of NBSH added. In other words, adding more diimide to achieve a higher level of reduction led to changes in the relative ratios of isomers in the monoreduction region even when both samples were sonicated (Figure 3C). We reasoned that the diimide reduction was selective for particular π-bonds in the system, such that certain isomers formed rapidly after the first reduction (causing them to appear enriched at low NBSH loadings) but were also consumed rapidly during the second reduction (causing them to diminish at higher NBSH loadings). Thus, in order to effectively compare the isomer distributions between sonicated and force-free samples, we needed identical degrees of reduction, a requirement that we found hard to achieve experimentally due to differences in reduction kinetics between the sonochemical and force-free conditions. Furthermore, preliminary results to this effect indicated that as the extent of reduction between sonicated and force-free samples became increasingly similar, the resulting isomer distributions also became more similar (Figure 3C, inset). While complete structural elucidation of isomers “A” and “B” was not possible since the relative positioning of the polymer arms is not discernible by ^19^F NMR spectroscopy, small molecule models provide strong evidence that these isomers correspond to monoreduced systems wherein the ^19^F substituent occupies the α-position (Figure 3C; see for details). Collectively, these data strongly indicated that reduction rates were far slower than thermal rearrangement rates in our system, and encouraged us to consider alternative approaches to mechanically tame bullvalene Hardy–Cope rearrangements.

In short, it appeared that our initial approach was limited by unfavorable selectivity of the isomer trapping reaction and rapid thermal re-equilibration under our experimental conditions (ca. −7 to 25 °C, the lowest temperatures we could maintain for the extended reaction times required to furnish reduction products). We reasoned that the first problem could be addressed by changing the isomer trapping reaction to target a more reactive site on the bullvalene core, for example by opening the strained cyclopropane ring. The second problem could be addressed by performing all experiments at significantly lower temperatures (e.g., ca. −72 °C, the lowest practical temperature we were able to reach during ultrasonication while keeping the polymer solubilized), where background thermal rearrangements would be much slower. In addition to increasing the half-lives of mechanically perturbed bullvalene isomers for subsequent chemical trapping, slowing the Hardy–Cope rearrangement process would also enable us to presonicate our polymers before quenching under force-free conditions, allowing us to ensure that the reactivity remained constant between our “force-perturbed” samples and the corresponding “force-free” control. To this end, we wanted to probe Hardy–Cope rearrangement kinetics at low temperatures to see if such an approach would be feasible. We elected to use ^19^F exchange spectroscopy (^19^F-EXSY NMR) to evaluate the rearrangement rate constant. EXSY NMR methods have been used frequently in prior studies to measure rate constants for bullvalene rearrangements,^26,37,59–61^ providing a solid theoretical support for our work.

## Kinetic Studies

Prior studies suggest that rearrangement rate constants in substituted bullvalenes vary significantly^56,62^ depending on the particular isomer interconversion pathway being investigated (e.g., rate constants for fluorobullvalene range from ca. 200–2000 s^–1^ at 25 °C).^56^ Since a full analysis of the 360 (=120 isomers × 3 rearrangements per isomer) total possible rearrangement events in our trisubstituted system seemed impractical, we chose to directly investigate a representative rearrangement process to determine an approximate order-of-magnitude upper bound for the rearrangement kinetics in our system. We reasoned that by estimating the maximum rate constant of the thermal rearrangements, we could determine how practical it would be to actually measure changes to the dynamic equilibrium without loss of sensitivity due to thermal reversions of the force-perturbed isomers. As an emblematic example, we chose to evaluate the rate of a β–γ transition (Figure 1A), since these particular ^19^F NMR signals have relatively high intensities and are well-resolved from each other in the ^19^F NMR spectrum (Figure 4A). Furthermore, it was previously reported that the β–γ transition in fluorobullvalene has the largest rearrangement rate constant out of its four possible transitions,^59^ indicating that such a transition would provide an upper bound to *all* possible rearrangement rate constants. Unfortunately, our initial attempts to measure rate constants at the target experimental temperature (i.e., ca. −72 °C) were unsuccessful. No cross-peaks were evident during these low-temperature selective 1D-EXSY experiments due to excessively low rate constants; competitive *T*_1_ relaxation prohibited us from running experiments with longer mixing times. Instead, repeating the selective 1D-EXSY experiments at 0 °C produced well-resolved cross-peaks in the expected chemical shift range (Figure 4A), allowing for the measurement of the exchange rate constants at this temperature (Figure 4B). We then extrapolated these data to −72 °C using the Arrhenius equation (see for details), assuming a typical substituted bullvalene rearrangement activation barrier (*E*_a_ = ca. 11–17 kcal/mol).^37,56,63,64^ Conveniently for our trapping experiments, the extrapolated rate constants suggest that the isomers in our system have half-lives with a lower bound of ca. 5–10 min. At the same time, we also recognized that the *actual* half-lives of species in solution may be substantially different than those obtained from measuring a single rate constant (i.e., selective 1D-EXSY, *vide supra*), since each bullvalene isomer has 3 potential (reversible) rearrangement pathways. Therefore, to corroborate our EXSY data, we also modeled the expected half-lives of isomers **γβ′δ′**, **γβ′γ″**, and **βδβ′**, as these isomers are calculated to be enriched by ultrasonication (Figure 2B). Each transition-state geometry in the network was automatically produced using *bullviso* and subsequently optimized at the PBE0-D3/def2-SV­(P) level of theory (Figure 4C). Full kinetic modeling of the network was done by calculating 360 first-order rate constants at 213 K and running stochastic Monte Carlo simulations using the Kinetiscope software^62^ (see for details). The results of these computations for isomer **γβ′γ″** are shown in Figure 4D, where isomer **γβ′γ″** has been indicated in the network diagram, and the color of each edge on the graph represents the transition state energy of a particular rearrangement (similar figures for isomers **γβ′δ′** and **βδβ′** are reproduced in ). As shown in Figure 4D, kinetic modeling of the isomer network using these values, starting from 100% of isomer **γβ′γ″**, indicates an experimental half-life of ca. 25 h for isomer **γβ′γ″** at 213 K.^32,35,62^ This result, coupled with the experimental measurements performed to establish an upper-bound for individual rearrangement rate constants, encouraged us in our pursuit to slow down the thermal Hardy–Cope rearrangement process to facilitate isomer trapping. Notably, the longer half-lives determined by computation (when compared to those measured by EXSY NMR, *vide supra*) indicate that the particular bullvalene isomers we expect to become enriched by mechanical force have relatively slow rearrangement rates and/or are trapped in “kinetic wells”, an emergent property of bullvalene network dynamics that have been shown to extend the half-lives of particular bullvalene isomers.^32,35,62^ In other words, we reasoned that even the fastest interconverting isomers would be long-lived enough to facilitate isomer quenching without substantial thermal reversion, provided we could identify a suitable quenching reaction at −72 °C.

**Figure 4:**
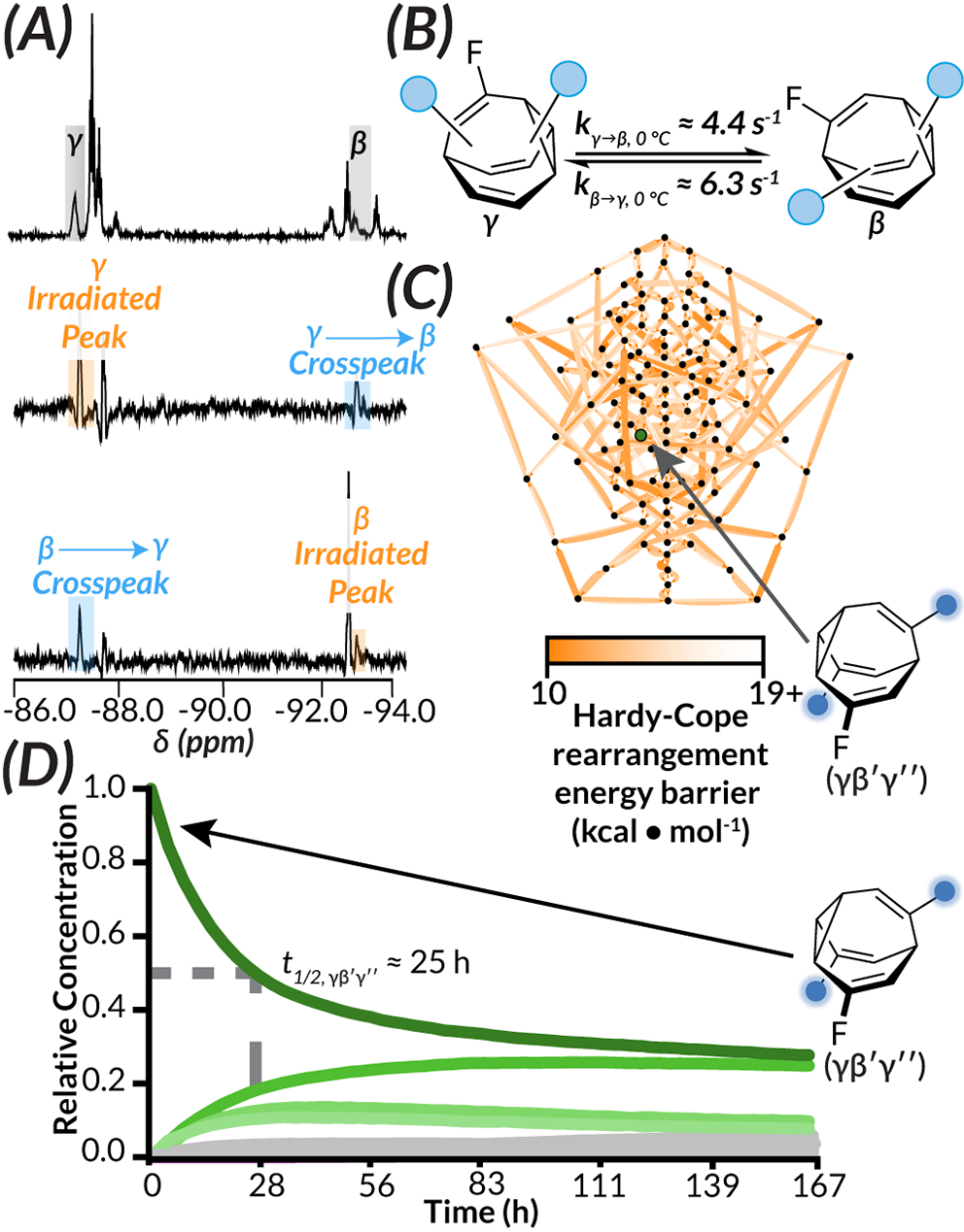
(A) ^19^F EXSY NMR spectroscopy reveals (B) a rate constant of ca. 6 s^–1^ for the reversible γ ⇄ β isomerization reaction. (C) Computational modeling of activation barriers for the full reaction network (D) enables kinetic modeling of the complete network equilibrium dynamics, revealing a half-life of ca. 25 h for isomer **γβ′γ″** at −60.15 °C.

## Low-Temperature Sonication and Isomer Trapping

With kinetics data in hand, we needed to select an isomer trapping reaction which would rapidly quell the Hardy–Cope rearrangement processes and operate selectively at low temperatures. We opted to utilize bromination of bullvalene’s vinyclopropane unit, a reaction that proceeds in <10 min at −75 °C to produce static dibromide intermediates.^65,66^ Additionally, since each cyclopropane unit has three potentially reactive sites and substitution at these sites is relatively uncommon in the overall isomer network, we reasoned that cyclopropyl unit reactivity should be much less sensitive to the substitution pattern than individual π-bonds (Figure 3B,C). Briefly, we prepared a dilute solution of **F-Bull-PMA** in diethyl carbonate (ca. 6 mg/mL) and sonicated (20 kHz pulsed sonication, 10.8 W/cm^2^, 0.5 s on/3.5 s off) for 1 h (total sonication “on” time) in a CO_2_/acetone bath (internal solution temperature during sonication = −66 to −70 °C). After sonication, the sample was rapidly split into two equal portions; one portion was kept cold (**F-Bull-PMA-Br**_**2**_ **-Cold**) and the other was allowed to warm to room temperature by placing it in a room-temperature (ca. 21.0–22.5 °C) water bath and allowing the solution to remain at this temperature for ca. 5 min (**F-Bull-PMA-Br**_**2**_ **-Warmed**). This process allowed **F-Bull-PMA-Br**_**2**_ **-Warmed** to re-equilibrate to the expected thermodynamic isomer distribution, clearing any potential mechanically driven “out-of-equilibrium” phenomena. **F-Bull-PMA-Br**_**2**_ **-Warmed** was then cooled back down to −72 °C (CO_2_/acetone bath, internal solution temperature at thermal equilibrium) and *both* samples were simultaneously quenched with Br_2_. Notably, we do observe some differences to the NMR spectra when comparing force-free samples (i.e., **F-Bull-PMA-Br**_**2**_ **-FF**) to samples that were sonicated and subsequently warmed to room temperature to re-equilibrate (i.e., **F-Bull-PMA-Br**_**2**_ **-Warmed**) (). However, control experiments indicate that following sonication and warming to room temperature, the original “equilibrium” isomer distribution is completely recovered; there are no differences in the ^19^F spectrum when comparing sonicated **F-Bull-PMA** samples to untreated **F-Bull-PMA** samples (i.e., **F-Bull-PMA-Sonics-Control** vs **F-Bull-PMA**; ) prior to Br_2_ addition. We therefore attribute these differences in NMR spectra “patterning” between force-free and re-equilibrated samples to changes in reactivtity with Br_2_ induced by sonochemical processing. For instance, sonochemical degradation of diethyl carbonate to form ethanol may increase the proportion of monobrominated bullvalene isomers which are quenched with ethanol instead of bromide, a process we observed by mass spectrometry to form minor side products under force-free conditions due to trace ethanol in the solvent (). Importantly, our experimental design accounts for these sonication-induced differences in reactivtiy by sonochemically processing control samples (**F-Bull-PMA-Br**_**2**_ **-Warmed**) and experimental samples (**F-Bull-PMA-Br**_**2**_ **-Cold**) at the same time. This workflow ensures that the force-perturbed (**F-Bull-PMA-Br**_**2**_ **-Cold**) and thermally re-equilibrated (**F-Bull-PMA-Br**_**2**_ **-Warmed**) samples were prepared under identical conditions (Figure 5A); therefore, any differences in the NMR patterning between samples cannot be attributed to changes in reactivity due to sonochemical processing. The samples were then analyzed by ^19^F NMR spectroscopy to probe for potential differences to the isomer distributions (Figures 5B and ): First, the isomers in the well-resolved region (ca. −91 to −100 ppm) were integrated, and the total integration of the region was normalized to 100. Average integrations for each isomer plus or minus one standard deviation are plotted in Figure 5C. Gratifyingly, statistical testing (two-tailed independent samples *t*-test) reveals significant differences (*n* = 3) in selectisomer integrations, up to ca. 5% enrichment or depletion due to applied force (Figure 5C), supporting our initial hypothesis (see for details on integration and statistical testing methodologies). Indeed, external force is able to tame Hardy–Cope rearrangements.

**Figure 5:**
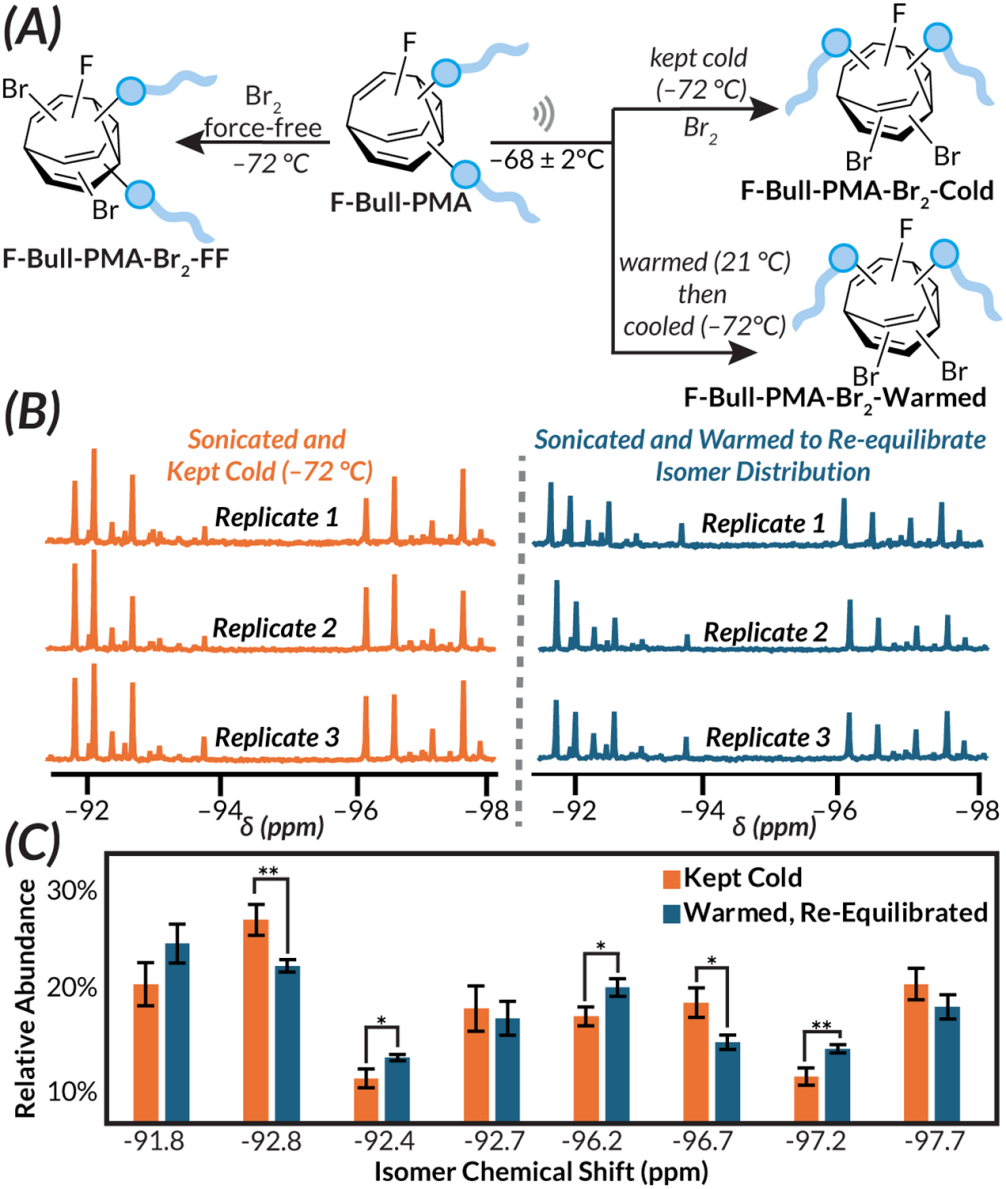
(A) Reaction scheme for the sonication and bromination of **F-Bull-PMA**. (B) ^19^F NMR spectroscopy analysis of sonicated **F-Bull-PMA** reveals distinct, reproducible isomer patterning differences between samples which were kept cold or warmed to re-equilibrate the isomer distribution. (C) Integration of signals in the diagnostic −90 to −100 ppm region reveals significant differences (as measured by two-tailed *t* tests; * = *p* < 0.05 and ** = *p* < 0.01; see for details) in relative isomer abundance between **F-Bull-PMA** samples which were kept cold or warmed to re-equilibrate the isomer distribution.

To further confirm the role of bullvalene mechanochemical activation in our system, we subjected small molecule analog **F-Bull-ATRP** to the same ultrasonic procedure used for PMA samples (Figure 6A). Importantly, we did not observe any significant difference between the isomer distributions between **F-Bull-ATRP-Br**_**2**_ **-Cold** and **F-Bull-ATRP-Br**_**2**_ **-Warmed** (Figure 6B). Small molecules, which are not long enough to experience mechanical stress during the turbulent flow of sonication, do not undergo the same mechanochemical reactions as larger polymers.^67,68^ This result therefore provides compelling evidence that the changes to the isomer patterning observed in the PMA sonication system (Figure 5B) are due to mechanical stress experienced by bullvalene in the larger macromolecules. While the resulting isomer distributions from our PMA samples (Figures 5B and ) are too complex for full structural assignment of the trapped bullvalene isomers, we were interested in using a small molecule model to further probe the reactivity of our quenching bromination reaction. Use of small molecule model **F-Bull-ATRP** also allowed us to confirm the reaction outcome following bullvalene bromination under force-free conditions by ^19^F NMR spectroscopy (Figure 6C) and high-resolution mass spectrometry (HRMS) (Figure 6D). The ^19^F NMR spectroscopy analysis shows good agreement between force-free small molecule ATRP initiator (**F-Bull-ATRP-Br**_**2**_ **-FF**) and PMA (**F-Bull-PMA-Br**_**2**_ **-FF**) samples, indicating that the reaction outcome was similar in this small molecule model system. Furthermore, HRMS revealed that only one equivalent of Br_2_ reacted, with no evidence of higher-order brominations. Importantly, these collective data support our initial assumption of monobromination in PMA samples (). While we do observe differences between bromination products in force-free model **F-Bull-ATRP-Br**_**2**_ **-FF** and re-equilibrated model **F-Bull-ATRP-Br**_**2**_ **-Warmed**, we once again (*vide supra*) attribute these discrpenacies to reactivity differences following sonochemical processing ().

**Figure 6:**
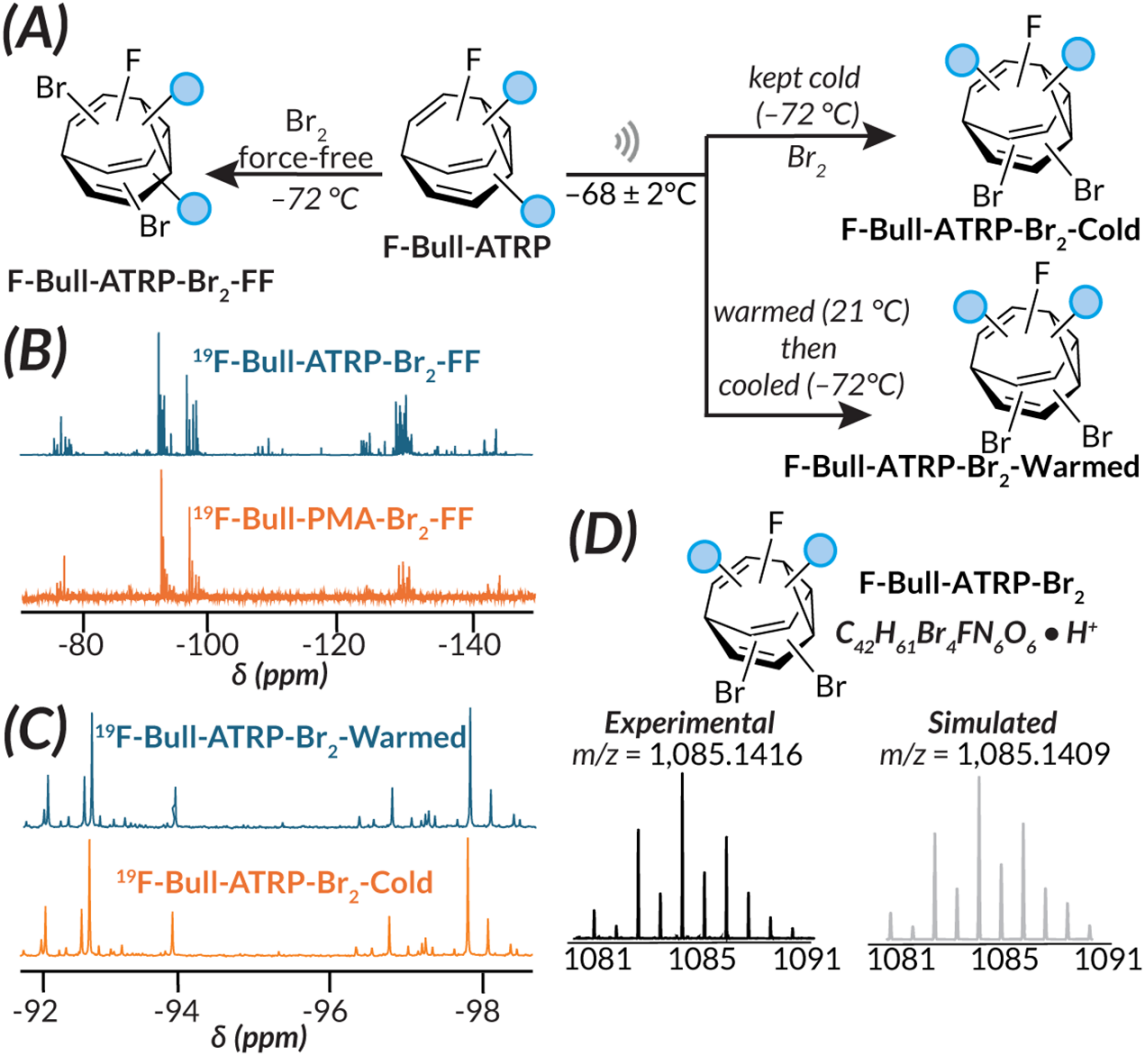
(A) Reaction scheme for the sonication and bromination of **F-Bull-ATRP**. (B) ^19^F NMR spectroscopy analysis of brominated samples demonstrates that the bromination reaction proceeds identically for both **F-Bull-ATRP** and **F-Bull-PMA**. (C) ^19^F NMR spectroscopy analysis of sonicated **F-Bull-ATRP** reveals no differences in the isomer patterning between samples which were kept cold or warmed to re-equilibrate the isomer distribution. (D) HRMS analysis of **F-Bull-ATRP-Br**_**2**_ **-FF** indicates that the reaction produces the desired product, with no evidence of decomposition products or higher-order halogenation.

## Conclusions

Bullvalene is a fluxional molecule which rapidly undergoes [3,3] sigmatropic Hardy–Cope rearrangements due to its “preorganized” tricyclic scaffold. Substituted bullvalenes therefore sample all possible valence isomers at room temperature, accessing a wide array of molecular geometries. Previous work in our lab showed that bullvalene incorporation into polymeric materials modulates their thermomechanical properties, a phenomenon we attribute to “molecular flexibility” of the bullvalene core. However, direct observations of bullvalene rearrangements in these “shapeshifting” soft materials have remained elusive. Herein we demonstrate through a mechanophore-centered polymer approach that the bullvalene isomer distribution changes in response to applied force; bullvalene bromination traps the sufficiently long-lived “out-of-equilibrium” population following mechanical taming of Hardy–Cope rearrangements. Notably, this effect is absent in molecular bullvalene models under analogous experimental conditions (Figure 6C). This work provides the first direct evidence of molecular rearrangements in bullvalene-containing macromolecules in response to applied force, highlighting bullvalene’s unique stature as a *fluxional mechanophore*. Notably, the fully reversible nature of the low-barrier Hardy–Cope rearrangements process also makes bullvalene the first example of a privlidged mechanophore class which can undergo multiple mechanochemical reactions, involving multiple sequential pericyclic steps, without requiring further manipulations for subsequent mechanochemical activity. We anticipate these findings to pave the way for force-responsive materials based on molecular fluxionality, leading to materials with heightened mechanical properties (e.g., by designing the bullvalene core to *reversibly* release stored length upon rearrangement and/or to be activated with tunable activation barriers) and superior analyte sensing capabilities (e.g., by sensing time-dependent analyte responses in out-of-equilibrium contexts).

## Supplementary Material







## Notes

Experimental and synthetic procedures, analytical spectra (NMR, MS), chromatograms (GPC, GC-MS), and computational details (PDF)
XYZ coordinates for ground-state DFT calculations (CSV)
XYZ coordinates for transition-state DFT calculations (CSV)
