# Supplementary material for: Mechanical Taming of Hardy–Cope Rearrangements
Source: ACS Cent Sci. 2026 May 7;12(6):766–76. doi: 10.1021/acscentsci.6c00254 (PMC13306599; doi:10.1021/acscentsci.6c00254)
Supplement: Supplementary file 1 [file oc6c00254_si_001.pdf]

Supporting Information For:

Mechanical Taming of Hardy-Cope Rearrangements

Matthew J. Elardo[1†], Mariia Kuznetsova[2,3†], Jason D. Kaff[1], Kiyoshi J. Colon[1], Gregory L. Olsen[1], Paul R. McGonigal[2*], & Matthew R. Golder[1*]

[1]Department of Chemistry and Molecular Engineering and Science Institute, University of Washington, Seattle, WA 98195, United States

[2]Department of Chemistry, Oxford University, Oxford, OX1 3TA, United Kingdom.

[3]Department of Chemistry, University of York, York, YO10 5DD, United Kingdom

† = *equal contributions*

*Corresponding authors: goldermr@uw.edu, paul.mcgonigal@chem.ox.ac.uk

Table Of Contents:

1.0: General Considerations ____ S2

2.0: Synthetic and Experimental Procedures ____ S4

2.1: Synthesis of Described Compounds ____ S4

2.2: Ultrasonication and Isomer Trapping Experiments ____ S16

3.0: Spectroscopic and Chromatographic Data ____ S32

3.1: ^{1}H NMR Spectra ____ S32

3.2: ^{19}F NMR Spectra ____ S58

3.3: ^{13}C NMR Spectra ____ S95

3.6: ^{19}F Exchange Spectroscopy (EXSY) Data ____ S106

3.7: Gas Chromatagrams and Mass Spectra ____ S113

3.8: GPC Chromatagrams ____ S124

4.0: Isomer Distribution Statistical Testing Results ____ S128

5.0: Computational Details ____ S133

5.1: DFT Calculations and Population Analysis At 213 K ____ S133

5.2: Isomer Stretching and Compressing Calculations ____ S154

5.3: Kinetic Modelling ____ S173

5.4: Python Script ____ S179

6.0: References ____ S194

1.0: GENERAL CONSIDERATIONS

Materials and Methods

All reagents were purchased from commercial suppliers and used as received unless otherwise noted. Glassware was flame dried or dried in an oven overnight at 120 °C before use. Anhydrous tetrahydrofuran (THF), dichloromethane (DCM), dimethyl formamide (DMF), and dimethyl sulfoxide (DMSO) were obtained from a JC Meyer solvent purification system and degassed either by sparging with nitrogen or by three successive freeze-pump-thaw cycles, as specified. Acetonitrile (MeCN) was stored over activated 3 Å molecular sieves for at least 3 days prior to use and was degassed by sparing with nitrogen or at by three successive freeze-pump-thaw cycles as specified. All moisture and air-sensitive reactions were performed under inert atmosphere (nitrogen) using standard Schlenk technique or, when noted, in a Vacuum Atmosphere OMNI glovebox or Innovative Technologies glovebox. SiliaFlash F60 (40-63 µm, 230-400 mesh) silica gel was used for column chromatography. Automated flash chromatography was performed using a Yamazen Smart Flash AKROS system. Sonication experiments were performed using a Branson Digital Sonifier SFX 550 running firmware version 1.3. The sonicator was equipped with a Branson Model 102-C Converter (Part number 101-135-066R) connected to a Sonics & Materials Full Wave ½" Probe (Part number 630-0217) with a Sonics & Materials 5" Glassware Adapter (Part number 830-00014). Sonication vessels were purchased from Ace Glass; product number 9844-19 was used for reactions requiring ≥10 mL solvent and product number 9843-25 was used for reactions requiring <10 mL solvent. Internal solution temperatures were monitored using a K-type digital thermocouple inserted into the solution through a punctured rubber septum. The sonicator was calibrated according to the literature method[1] (see **SI Section 2.2**). Photochemistry was performed using an EvoluChem PhotoRedOx Box (HepatoChem) with 365 nm LEDs.

Characterization

^{1}H nuclear magnetic resonance (^{1}H NMR) spectra were taken on a Bruker AVANCE-NEO at 500 MHz or on a Bruker AVANCE-III at 500 MHz. ^{13}C nuclear magnetic resonance (^{13}C NMR) spectra were recorded on a Bruker AVANCE-NEO at 126 MHz or on a Bruker AVANCE-III at 126 MHz with broadband decoupling. ^{19}F nuclear magnetic resonance (^{19}F NMR) spectra were taken on a Bruker AVANCE-NEO at 470 MHz or on a Bruker AVANCE-III at 470 MHz. Unless stated otherwise, ^{19}F NMR spectra were collected with ^{1}H decoupling. ^{1}H NMR spectra were taken in chloroform-*d* with TMS and $CFCl_3$ added ($CDCl_3$, referenced to residual $CHCl_3$ at δ = 7.16 ppm) or deuterated acetonitrile (CD_3CN, referenced to residual CH_3CN at δ = 1.94 ppm). ^{13}C NMR spectra were taken in chloroform-*d* ($CDCl_3$, referenced to solvent at δ = 77.16 ppm). ^{19}F NMR spectra were taken in chloroform-*d* with TMS and $CFCl_3$ added ($CDCl_3$, referenced to $CFCl_3$ at δ = 0.00 ppm) or in acetone-d_6 ($CO(CD_3)_2$), unreferenced). Spectra were analyzed on MestreNova software (version 14.2.3-29241) and Bruker TopSpin (version 4.5.0). Chemical shifts are reported in parts per million (ppm); splitting patterns are assigned as s (singlet), d (doublet), t (triplet), q (quartet), m (multiplet), and br (broad); coupling constants, *J*, are reported in hertz (Hz). All NMR spectra are reported at room temperature unless stated otherwise (298 K = 24.85 °C). The software program EXSYCalc by MestreLab Research[2] was used to extract exchange rate constants from a series of 1D NOESY experiments.

Gas chromatography–mass spectrometry (GC-MS) data were collected on an Agilent 6890 gas chromatograph coupled to an Agilent 5973 quadrupole with mass spectrometer with electron impact ionization (EI-MS). The analyte was prepared at a concentration of ca. 0.1 mg/mL in dichloromethane. High resolution electron spray ionization mass spectrometry (ESI-HRMS) experiments were collected on a Thermo LTQ Orbitrap mass spectrometer. The samples were prepared at a concentration of ca. 0.1 mg/mL in methanol. Methanol or MeCN/H_2O (+ 0.1% formic acid) were used as the mobile phases for HRMS, as specified. Ammonium (NH_4^+) was added as needed to induce adduct formation and facilitate ionization.

Analytical gel permeation chromatography (GPC) data were collected on Agilent 1260 HPLC equipped with a Wyatt 8-angle DAWN NEON light-scattering detector, ViscoStar NEON viscometer, and Optilab NEON refractive index detector. GPC samples were analyzed at a flow rate of 1.0 mL/min in chloroform (stabilized with 0.5 – 1.0% ethanol) through two Agilent PLgel MIXED-C columns at 35 °C. A *dn/dc* value of 0.0381 (estimated *via* on-line measurement by using the 100% mass recovery method) was taken for all poly(methyl acrylate) (PMA) samples.

Statistical testing was performed using IBM SPSS 31.0.0.0 version (117). Significance results are reported as p-values from two-tailed t-tests, where * = $p \leq 0.05$, ** = $p \leq 0.01$, and *** = $p \leq 0.001$

No unusally high or unexpected hazards were ecountered. All chemical manipulations were performed in a positive-pressure glovebox or a chemical fume hood using standard techniques.

2.0: SYNTHETIC AND EXPERIMENTAL PROCEDURES

2.1: SYNTHESIS OF DESCRIBED COMPOUNDS

Synthesis of bromocyclooctatetra-1,3,5,7-ene (Br-COT)

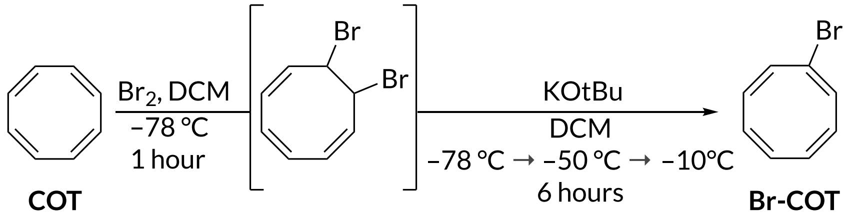

A 250 mL Schlenk flask was charged with a magnetic stirring bar and was flamed dried under high vacuum. The flask was backfilled with nitrogen, and cyclooctatetra-1,3,5,7-ene (COT, 6.0 g, 58 mmol, 6.5 mL, 1.0 eq) was dissolved in anhydrous dichloromethane (40 mL), added to the Schlenk flask. The yellow solution was stirred in a –78 °C (dry ice/acetone) bath for 5 minutes to equilibrate the solution temperature. Bromine (9.5 g, 82 mmol, 3.1 mL, 1.0 eq.) was added to an oven-dried vial and dissolved in anhydrous DCM (17 mL). The Br_2 solution was added to the COT solution dropwise *via* syringe at a flow rate of 175 μL/min using a syringe pump (addition time ca. 1.75 hours). Over the course of the addition, the yellow COT solution turned slightly reddish orange. After the addition was completed, the resulting solution was stirred at –78 °C for 1 hour, during which time the red color of the solution faded back to yellow. Potassium *tert*-butoxide (9.2 g, 82 mmol, 1.4 eq.) was added to the solution in four equal portions (2.3 g per portion) under a stream of nitrogen, with 10 minutes between each addition. The resulting pale yellow suspension was stirred at –78 °C for 1 hour, becoming more yellow/beige as it stirred. The suspension was then warmed to –50 °C (dry ice/50% methanol in water) and stirred at this temperature for 3 hours. The resulting brown suspension was warmed to –10 °C (dry ice/20% methanol in water) and stirred for 2 more hours. The brown-red suspension was then poured into 90 mL of 2% (v/v) acetic acid in deionized water, and magnesium sulfate (~25 g) was added to promote phase separation. The reaction vessel was rinsed with diethyl ether (2 x 25 mL) and added to the acetic acid slurry. The orange suspension was stirred for 15 minutes, then was poured into a separatory funnel. The organic phase was separated and diluted into diethyl ether (100 mL), and the organic phase was washed subsequently with water (2 x 75 mL), saturated aqueous sodium bicarbonate (2 x 75 mL), and saturated aqueous sodium thiosulfate (1 x 75 mL). The combined aqueous phases were back extracted with diethyl ether (1 x 150 mL), and the combined organic phase was dried with brine (1 x 150 mL) followed by sodium sulfate. The cherry red organic phase was filtered, reduced *in vacuo*, and the dark red crude liquid was purified *via* silica plug eluting with pentane (200 mL) to deliver the product as a yellowish-orange liquid (8.0 g, 76%).

Characterization Data for ***Br-COT****:*
^1H NMR (500 MHz, $CDCl_3$) δ 6.22 (br, 1H), 5.97 – 5.76 (m, 5H), 5.64 (br, 1H). *NMR spectroscopy is in agreement with literature data*[3].

Synthesis of fluorocyclooctatetra-1,3,5,7-ene (F-COT)

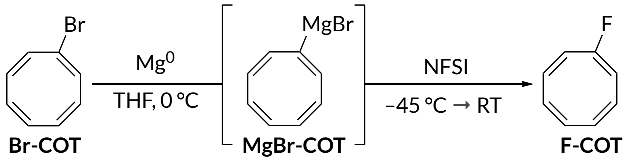

A 250 mL 3-necked flask was charged with a magnetic stirring bar and a pressure-equalized addition funnel, and the apparatus was flamed dried under high vacuum. The reaction setup was backfilled with nitrogen, and magnesium turnings (1.7 g, 68 mmole, 2.5 eq.) were added to flask. The flask was evacuated and refilled with nitrogen three times, and anhydrous THF (50 mL) was added. The magnesium suspension was cooled to 0 °C in an ice bath, and **Br-COT** (5.0 g, 27 mmole, 1.0 eq.) was dissolved in anhydrous THF (60 mL) and added to the addition funnel. The **Br-COT** solution was added to the stirred turnings at 0 °C at approximately 1 mL/minute (addition time ca. one hour), and the resulting yellow solution was stirred for 1.5 hours at 0 °C, during which time the solution became dark forest green (almost black), indicating the formation of the Grignard reagent, **MgBr-COT**. Complete conversion of **Br-COT** to **MgBr-COT** may be further confirmed by quenching an aliquot with D_2O and analyzing *via* GC-MS.

Separately, a 500 mL Schlenk flask was flame dried and charged with *N*-fluorobenzenesulfonimide (NFSI, 8.6 g, 27.3 mmol, 1.0 eq.), and the flask was evacuated and refilled with nitrogen 3 times. The NFSI was dissolved in anhydrous THF (54 mL) and cooled to –45 °C (dry ice/acetonitrile). The chilled solution was sparged for one hour with nitrogen, and the **MgBr-COT** solution was added dropwise to the NFSI solution *via* cannula transfer over a period of 30 minutes (ca. 3.5 mL/minute addition rate). The resulting orange solution was stirred at –45 °C and allowed to slowly warm to RT over the course of 16 hours. The dark red crude solution was quenched with 50 mL of saturated aqueous ammonium chloride, and the organic phase was separated and washed with saturated aqueous ammonium chloride (3 x 50 mL). The aqueous phase was back extracted with diethyl ether (3 x 50 mL), and the combined organic phase was dried with brine (1 x 100 mL) followed by sodium sulfate. The dark red crude solution was filtered and carefully concentrated *in vacuo*, keeping the bath temperature at 25 °C and the pressure above 100 torr, to a volume of ca. 30 mL. The resulting dark red solution was filtered over a plug of silica eluting with 100 mL pentane. During the plug, a yellow liquid initially elutes, and residual NFSI/NHSI (carried through the plug by residual polar solvent) precipitated as the plug was rinsed with pentane. The resulting suspension was refiltered over the same silica plug, eluting again with pentane (100 mL), and the process was repeated as necessary until no more solids precipitated (usually two plugs is sufficient). The resulting yellow filtrate was collected and carefully concentrated *in vacuo*, again keeping the bath temperature at 25 °C and the pressure higher than 100 torr. Due to the volatility of the product, the compound was not evaporated to dryness and was instead isolated as a yellowish-orange solution in residual THF (ca. 3-5 mL THF remaining). The yield was calculated *via* quantitative ^{19}F NMR spectroscopy taking fluorobenzene as an internal standard and the solution was used immediately in the next step (1.9 g, 58%). *Optimization tables for this reaction are provided below* (**Tables S1-S2**)

Characterization Data for ***F-COT:***
^{1}H NMR (500 MHz, $CDCl_3$) δ 5.91 – 5.85 (m, 2H), 5.85 – 5.77 (m, 3H), 5.61 (br, 2H).

^{19}F NMR (470 MHz, $CDCl_3$) δ -92.80.

^{13}C NMR (126 MHz, $CDCl_3$) δ 156.35 (d, J = 248.1 Hz), 133.44 (d, J = 3.6 Hz), 132.20, 131.50.

GC-MS (EI): Calculated for C_8H_7F [$M^{•+}$]: 122.1; found: 122.1

Table S1: Optimization table for synthesis of **F-COT** *via* **Li-COT**.

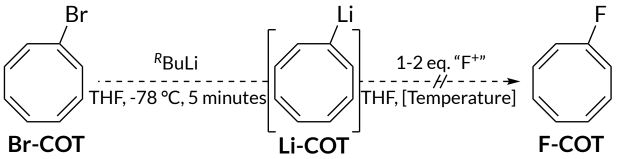

Temperature:	Alkylithium:	"F+" Source:	Yield [^{19}F NMR] (%):
–78 °C	*n*-Butyllithium	1.2 eq. NFSI	**<5%**
–78 °C	*tert*-Butyllithium	1.2 eq. NFSI	**30%** *(poor reproducibility)*
–78 °C	*tert*-Butyllithium	1.5 eq. NFSI; Inverse addition	**5%**
–78 °C g –40 °C g RT	*tert*-Butyllithium	1.5 eq. NFSI	**<1%**
–78 °C	*tert*-Butyllithium	1.5 eq. Selectfluor	**<1%**

Table S2: Optimization table for synthesis of **F-COT** *via* **MgBr-COT**.

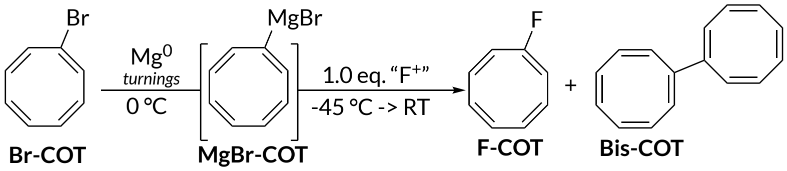

Solvent:	[COT]:	"F+" Source:	Yield [^{19}F NMR] (%):
THF	0.6 M	NFSI	**10%** *(with unquantified amounts of* ***Bis-COT****)*
**THF**	**0.17 M**	**NFSI**	**50-60%** *(No* ***Bis-COT*** *formation)*
THF	0.17 M	Selectfluor	**<1%** *(recovered* ***COT*** *from unreacted* ***MgBr-COT****)*
Ether	0.17 M	NFSI	**<1%** *(No* ***MgBr-COT*** *formation even with I_2 or $C_2H_4Br_2$ entrainment)*

Synthesis of 2,2,3,3,10,10,11,11-Octamethyl-4,9-dioxa-3,10-disiladodec-6-yne (TBDMSO-Alkyne)

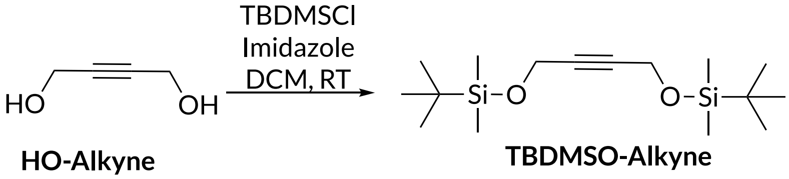

A 1-L round-bottomed flask was charged with a magnetic stirring bar, *tert*-butyldimethylsilyl chloride (TBDMSCl, 77 g, 510 mmol, 2.2 eq.), imidazole (47 g, 700 mmol, 3.0 eq.), and 1,4-butynediol (20 g, 230 mmol, 1.0 eq.). Dichloromethane was added to the flask (465 mL), and the suspension was stirred for 24 hours. The suspension was filtered over celite, and the colorless residue was rinsed with dichloromethane (3 x 50 mL). The yellow filtrate was washed with saturated aqueous sodium bicarbonate (3 x 100 mL) and water (1 x 200 mL) and the combined aqueous phase was back extracted with dichloromethane (1 x 100 mL). The combined organic phase was dried with brine (1 x 100 mL) and concentrated to a volume of ca. 100 mL *in vacuo*. The crude pale-yellow solution was passed over a plug of silica, eluting with 100 mL DCM, to deliver a colorless filtrate which was further concentrated to deliver the product **TBDMSO-Alkyne** as a colorless, transparent, viscous liquid (70 g, 96%).

Characterization Data for ***TBDMSO-Alkyne:***
^1H NMR (500 MHz, $CDCl_3$) δ 4.32 (s, 4H), 0.89 (s, 18H), 0.10 (s, 12H). *NMR spectroscopy is in agreement with literature data*[4].

Synthesis of [1,2-Bis(diphenylphosphino)ethane]cobalt dibromide ($CoBr_2$(dppe))

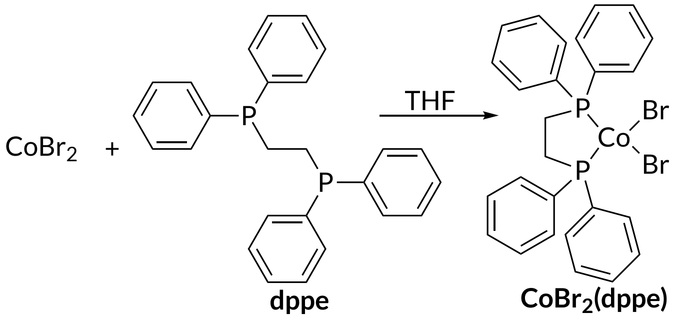

Cobalt(II) bromide ($CoBr_2$, 50 g, 230 mmol, 1.0 eq.) was added to a 2-L flame dried three-necked flask with a flame-dried magnetic stirring bar. The flask was then evacuated and refilled with nitrogen three times. Dry THF (500 mL) was added to the reaction flask and stirred until the cobalt fully dissolved, delivering a dark blue solution. Subsequently, 1,2-bis(diphenylphosphino)ethane (dppe, 91 g, 230 mmol, 1.0 eq.) was added under a stream of nitrogen, and the resulting dark green suspension was stirred at room temperature for 16 hours. The reaction mixture was filtered, and the dark green precipitate was washed with pentane (3 x 100 mL) and dried under high vacuum for 16 hours, yielding the catalyst as a forest green powder (140 g, 99%). The catalyst was used directly in future reactions with no further characterization or purification.

Synthesis of ^{19}F Cycloadduct Diol (F-CA-Diol)

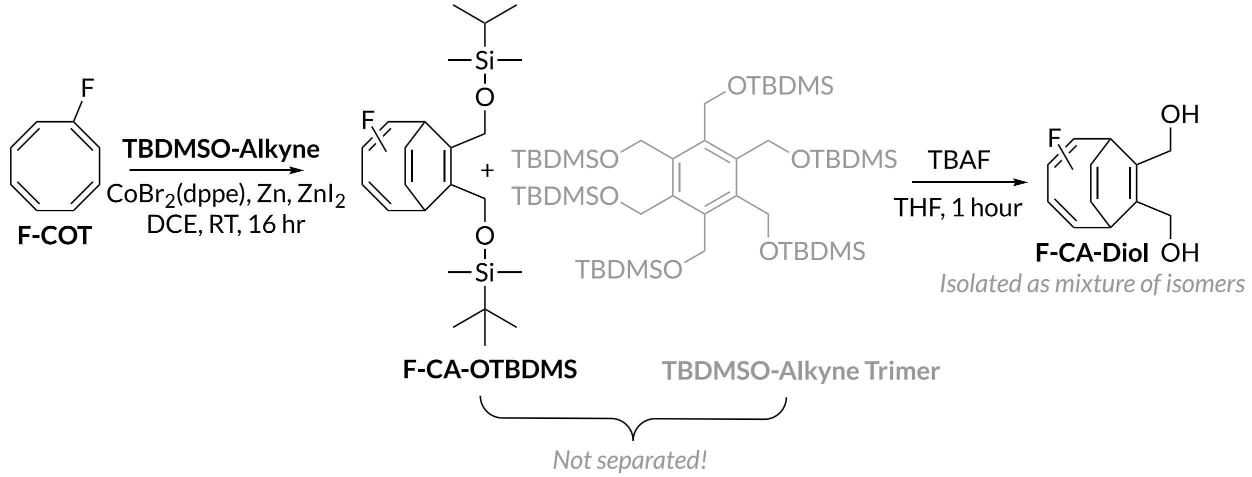

A 250-mL Schlenk flask was charged with a stirring bar and flame dried under high vacuum. To this flask was charged $CoBr_2$(dppe) (15 g, 25 mmol, 0.80 eq.), zinc dust (3.2 g, 49 mmol, 1.5 eq), and ZnI_2 (11 g, 35 mmol, 1.1 eq.). The catalyst mixture was suspended in dry dichloroethane (DCE, 40 mL), which had previously been sparged with nitrogen for one hour. The dark black suspension was stirred for 5 minutes at 35 °C. **F-COT** (4.0 g, 33 mmol, 1.0 eq. [mmol of **F-COT** based on NMR yield of previous step]) was added to the catalyst mixture at once as a solution in 10 mL of dried, sparged DCE. **TBDMSO-Alkyne** (21 g, 68 mmol, 2.1 eq.) was dissolved in dried, sparged DCE (50 mL) and was added to the reaction mixture portion-wise over the course of 5-10 minutes. The resulting dark black suspension was stirred at 35 °C for 48 hours under an atmosphere of nitrogen. The crude mixture was poured into 100 mL of diethyl ether to precipitate out the catalyst (which forms a brown precipitate), and the mixture was filtered over a plug of neutral alumina eluting with diethyl ether (150 mL). The process of filtering over neutral alumina and rinsing with ether was performed until the filtrate emerged as a clear yellow solution; usually one or two plugs is sufficient. The crude solution was concentrated to deliver the crude mixture as a yellow-red oil. The material was isolated as a mixture of **F-CA-OTBDMS** isomers along with a trimer formed from the **TBDMSO-Alkyne** which does not separate on silica gel (but is easily removed and isolated after the next reaction). Thus, quantitative ^{19}F NMR was used to measure an NMR yield taking fluorobenzene as an internal standard, and the material was telescoped to the next step with no further purification (5.8 g, 40%; see **NMR Spectra** below for crude ^{1}H and ^{19}F spectra).

Crude **F-CA-OTBDMS** (5.8 g, 13 mmol, 1.0 eq. [mmol of **F-CA-OTBDMS** based on NMR yield of previous step]) mixed with **TBDMSO-Alkyne Trimer** was added to a flame-dried 250-mL Schlenk flask with stirring bar, and the material was dissolved in anhydrous THF (26 mL) to deliver an amber solution. To this solution was added tetrabutylammonium fluoride (TBAF) as a 1M solution in THF (79.1 mL, 79.1 mmol, 6.1 eq.) in one portion. On addition, the solution immediately turns dark, cherry red. After stirring for one hour, the reaction is complete (verify consumption of starting material *via* TLC with 1% EtOAc in hexanes, stained with ceric ammonium molybdate, starting material R_f = 0.69). The crude mixture was washed with saturated aqueous ammonium chloride (3 x 75 mL), and the combined aqueous phases were back extracted with DCM (1 x 50 mL). The combined organic phases were dried with brine (1 x 100 mL) then sodium sulfate. The dried organic phase was filtered and concentrated, and the red oily crude material was purified *via* flash chromatography with a 50%g80% EtOAc in hexanes gradient, delivering the product **F-CA-Diol** isomer mixture as a pale-yellow powder (2.4 g, 89% from **F-CA-OTBDMS**; 36% from **F-COT**).

Characterization Data for ***F-CA-Diol:***
^{1}H NMR (500 MHz, $CDCl_3$) δ 6.38 – 6.06 (overlapping peaks, 1H), 5.88 – 5.49 (overlapping peaks, 4H), 4.44 – 4.14 (overlapping peaks, 4H), 3.78 – 3.38 (overlapping peaks, 2H), 2.75 – 2.41 (br, 2H).

^{19}F NMR (470 MHz, $CDCl_3$) δ -73.89, -100.66, -115.93, -152.94.

^{13}C NMR (126 MHz, $CDCl_3$) δ 141.83, 136.21 (d, *J* = 8.9 Hz), 125.01, 122.38, 121.32, 121.22, 119.06, 102.90 (d, *J* = 29.2 Hz), 60.57, 40.90 (d, *J* = 30.1 Hz), 38.28, 37.53.

R_f = 0.23 (80% ethyl acetate/hexanes). *Preferred Stain: Ceric Ammonium Molybdate*

Synthesis of ^{19}F-Cycloadduct Propargyl Ether (F-CA-PE)

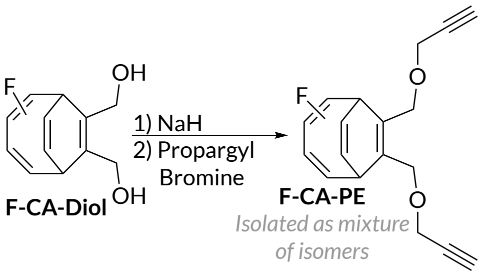

A 50-mL Schlenk flask was charged with a stirring bar and flame dried under high vacuum. To the dried flask was added NaH (60% dispersion in oil; 990 mg total for 590 mg NaH, 25 mmol, 6.0 eq.). The flask was evacuated and refilled with nitrogen 3 times and cooled to 0 °C on an ice bath. Anhydrous THF (3 mL) was added to the NaH, and the solid was suspended in the solvent and stirred for 1 minute. The NaH was allowed to settle, and the solvent was removed *via* syringe. This process was repeated two more times to fully remove oil from the NaH, and the rinsed NaH was suspended in anhydrous THF (10 mL). **F-CA-Diol** (860 mg, 4.1 mmol, 1.0 eq.) was dissolved in anhydrous THF (6 mL) and added to the chilled NaH suspension dropwise over 5 minutes (ca. 1 mL/min addition rate). The reaction was stirred for 30 minutes at 0 °C, and propargyl bromide (80% solution (wt/wt) in toluene, 3.1 mL, 3.4 g propargyl bromide, 28.7 mmol, 7.0 eq.) was added in one portion to the beige suspension at 0 °C. The resulting brown suspension was allowed to stir at 0 °C for 16 hours, slowly warming to RT. The crude mixture was quenched by careful addition of ice-cold water at 0 °C (10 mL), and the aqueous phase was separated and extracted with DCM (3 x 15 mL). The combined organic phases were dried first with brine (1 x 50 mL) then with sodium sulfate. The dried organic phase was filtered, and the solvent was removed *in vacuo*. The crude product was purified *via* silica plug, eluting with 30% ethyl acetate in hexanes (100 mL), and the resulting yellow filtrate was concentrated to deliver the product **F-CA-PE** as a red liquid which freezes on storage at –20 °C (1.0 g, 99%).

Characterization Data for ***F-CA-PE:***
^{1}H NMR (500 MHz, $CDCl_3$) δ 6.34 – 6.01 (m, 1H), 5.90 – 5.48 (m, 4H), 4.35 – 4.01 (m, 8H), 3.73 – 3.40 (m, 2H), 2.47 – 2.40 (m, 2H).

^{19}F NMR (470 MHz, $CDCl_3$) δ -74.26, -101.04, -116.20, -153.77.

^{13}C NMR (126 MHz, $CDCl_3$) δ 171.12, 141.58, 136.09 (d, *J* = 8.3 Hz), 131.62 (d, *J* = 22.3 Hz), 129.03, 128.31 (d, *J* = 2.7 Hz), 128.22, 125.97, 124.76, 124.64, 122.13, 120.91 (d, *J* = 29.2 Hz), 120.89, 118.88 (d, *J* = 2.1 Hz), 102.83, 102.59, 79.74 (d, *J* = 9.0 Hz), 74.62 (d, *J* = 7.9 Hz), 74.41, 65.86, 65.72, 65.57, 60.38, 56.76 (d, *J* = 11.7 Hz), 56.52, 40.49 (d, *J* = 30.7 Hz), 37.63, 36.92, 21.03, 14.19

Synthesis of ^{19}F-Propargyl Ether Bullvalene (F-Bull-PE)

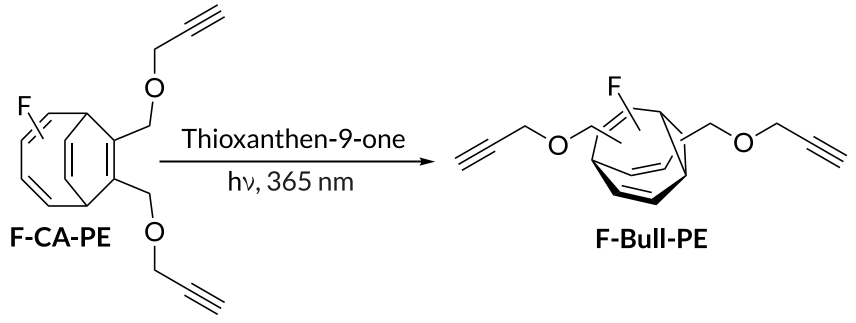

To a vial was added **F-CA-PE** (1.0 g, 3.6 mmol, 1.0 eq.) and thioxanthene-9-one (38 mg, 0.1 mmole, 3 mole%). The materials were dissolved in acetone (10 mL) and added to a vial with a stirring bar. The red solution was placed in an EvoluChem PhotoRedOx box, irradiated with UV light (λ = 365 nm), and stirred at RT for 20 hours. The acetone was removed *in vacuo*, and the crude red oil was purified *via* flash chromatography using 85% hexanes/15% ethyl acetate/1% triethylamine as the mobile phase. The product **F-Bull-PE** was eluted as a yellow oil (400 mg, 39%).

Characterization Data for ***F-Bull-PE:***
^{1}H NMR (500 MHz, $CDCl_3$) δ 6.22 – 5.59 (br, 4H), 4.19 – 3.91 (br, 8H), 2.95 – 2.03 (br, 5H).

^{19}F NMR (470 MHz, $CDCl_3$) δ -86.37 – -86.72 (br), -86.77 – -86.92 (br), -87.00 – -87.19 (br), -87.31 – -87.60 (br), -90.79 – -91.21 (br), -91.52 – -91.73 (br), -92.32 – -92.78 (br), -92.87 – -93.01 (br), -93.26 – -93.45 (br), -93.85 – -94.14 (br), -147.16 – -148.69 (br), -148.69 – -149.47 (br), -159.23 – -160.44 (br), -160.80 – -161.61 (br).

^{13}C NMR (126 MHz, $CDCl_3$) δ 136.38 – 135.25 (br.), 133.46 – 132.33 (br.), 131.19 (d, *J* = 14.8 Hz), 130.66 (d, *J* = 15.0 Hz), 129.47 – 127.67 (br.), 126.15 – 125.76 (br.), 125.90 – 125.29 (br.), 125.01 – 124.50 (br.), 124.20 (d, *J* = 14.9 Hz), 123.46 (d, *J* = 13.6 Hz), 122.80 (d, *J* = 15.7 Hz), 122.52 (d, *J* = 8.8 Hz), 122.25 (d, *J* = 14.8 Hz), 100.96 – 99.73 (br., overlapping peaks), 90.59 – 90.20 (br.), 89.44 – 88.78 (br.), 80.13 – 78.57 (br., overlapping peaks), 75.77 – 73.40 (br., overlapping peaks), 57.87 – 56.92 (br.), 57.32 – 55.10 (br., overlapping peaks), 30.77 – 24.71 (br., overlapping peaks), 23.47 – 16.50 (br., overlapping peaks).

HRMS (ESI): calculated for $C_{18}H_{18}FO_2N$ [M•NH_4^+]: 302.1551; found: 302.1557
R_f = 0.21 (15% ethyl acetate/85% hexanes/1% added triethylamine). *Preferred Stain: Potassium Permanganate*

Synthesis of 8-azido-octan-1-ol (N_3-Oct-OH)

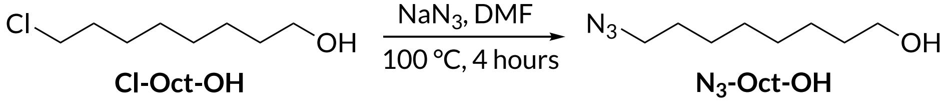

A 250 mL round-bottomed flask was charged with a stirring bar, 8-chloro-octan-1-ol (7.0 g, 7.2 mL, 43 mmol, 1.0 eq), and sodium azide (3.8 g, 58 mmol, 1.4 eq). The mixture was suspended in DMF (128 mL) and heated to 100 °C for 4 hours. The DMF was removed *in vacuo*, and the residue was passed through a silica plug eluting with DCM (50 mL). The filtrate was washed with 5% aqueous LiCl (10 x 65 mL). The combined aqueous phases were back-extracted with DCM (1 x 100 mL), and the combined organic phase was washed with brine and dried over sodium sulfate. The solvent was removed *in vacuo* to deliver the product **N_3-Oct-OH** as an amber, slightly pink liquid (7.1 g, 98%).

*Characterization Data for **N_3-Oct-OH**:*

^{1}H NMR (500 MHz, $CDCl_3$) δ 3.64 (t, J = 6.7 Hz, 2H), 3.25 (t, J = 7.0 Hz, 2H), 1.64 – 1.53 (m, 4H), 1.35 (br., overlapping peaks 8H), 1.25 (br, 1H). *NMR data in accordance with literature values.*[5]

Synthesis of 8-azidooctyl 2-bromo-2-methylpropanoate (N_3-ATRP)

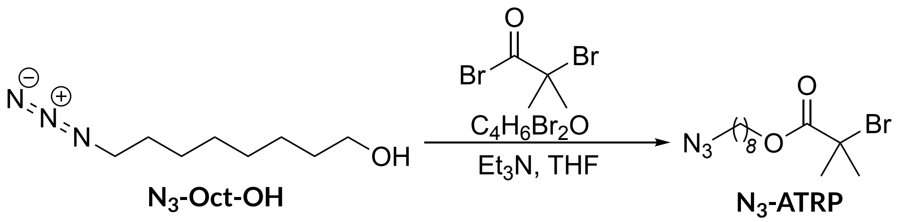

To a flame-dried 250-mL Schlenk flask with stirring bar was added **N_3-Oct-OH** (7.1 g, 42 mmol, 1.0 eq.) and triethylamine (6.4 mL, 4.6 g, 46 mmol, 1.1 eq.). The mixture was dissolved in anhydrous THF (120 mL), and the solution was cooled to 0 °C on an ice bath. To this mixture was added α-bromoisobutyryl bromide (5.7 mL, 11 g, 46 mmol, 1.1 eq.) dropwise. The resulting suspension was stirred at 0 °C for one hour, then was warmed to RT and stirred for a further 16 hours. The suspension was poured into diethyl ether (150 mL) and filtered over celite. The residue was rinsed with diethyl ether (3 x 10 mL), and the filtrate was concentrated *in vacuo*. The crude yellow oil was purified *via* flash chromatography, eluting with a gradient from 0%g15% ethyl acetate in hexanes. The product **N_3-ATRP** was eluted as a colorless liquid (9.9 g, 74%).

*Characterization Data for **N_3-ATRP**:*

^{1}H NMR (500 MHz, $CDCl_3$) δ 4.17 (s, 2H), 3.26 (t, J = 7.0 Hz, 2H), 1.93 (s, 6H), 1.73 – 1.53 (m, 4H), 1.44 – 1.29 (m, 8H). *NMR data is in accordance with literature values*[6].

R_f = 0.47 (5% ethyl acetate/hexanes). *Preferred Stain: Ceric Ammonium Molybdate*

Synthesis of ^{19}F-Bullvalene ATRP Initiator (F-Bull-ATRP)

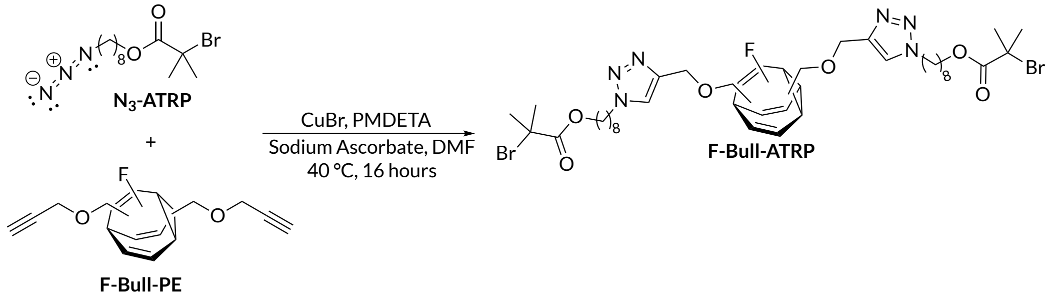

In a nitrogen filled glovebox, CuBr (40 mg, 0.30 mmol, 0.20 eq.) was suspended in anhydrous, deoxygenated DMF (2.8 mL) and deoxygenated *N,N,N',N'',N''*-pentamethyldiethylenetriamine (PMDETA, 0.120 mL, 97 mg, 0.6 mmol, 0.40 eq.) was added, forming an aquamarine solution. Separately, and also in the glovebox, **F-Bull-PE** (400 mg, 1.4 mmol, 1.0 eq.), **N_3-ATRP** (1.6 g, 5.0 mmol, 3.6 eq.), and sodium ascorbate (110 mg, 0.60 mmol, 0.40 eq.) were dissolved together in anhydrous, deoxygenated DMF (1.9 mL). The reagent solution was added to the CuBr solution, and the vial was capped and sealed with electrical tape. The sealed reaction mixture was cycled out of the box and stirred at 40 °C in an aluminum pie-block for 5 hours. After stirring 5 hours, the originally deep blue/aquamarine solution became a brown suspension, at which point another aliquot of sodium ascorbate (1 scoopula tip, ~30 mg) was added. Additionally, more CuBr was added until the solution became deep greenish-blue again (1-2 scoopula tips, ~50-100 mg). The vial was capped and sealed with electrical tape again and stirred at 40 °C for a further 16 hours. After stirring overnight, the solution turned reddish brown. TLC analysis (85% hexanes/15% ethyl acetate) indicated complete conversion of **F-Bull-PE** (staining with cerium ammonium molybdate; starting material R_f = 0.39), and the reaction was poured over a neutral alumina plug and rinsed with ethyl acetate (100 mL). The resulting colorless solution was concentrated to a volume of 50 mL, and the organic phase was washed with 5% aqueous lithium chloride solution (10 x 100 mL) to remove DMF. The combined aqueous phases were back extracted with ethyl acetate (1 x 100 mL), and the combined organic phases were washed with brine (1 x 200 mL), dried with sodium sulfate, filtered, and concentrated *in vacuo* to deliver the crude product as a yellow oil. The crude oil was purified *via* flash chromatography eluting with a 0%g5%g10% MeOH in DCM gradient (all mixtures had additional 1% triethylamine added). The product **F-Bull-ATRP** eluted as an orange-brown oil (740 mg, 57%).

Characterization Data for ***F-Bull-ATRP****:*

^{1}H NMR (500 MHz, $CDCl_3$) δ 7.52 (br. s, 2H), 5.98 – 5.69 (br. m, 3H), 4.70 – 4.48 (br. m, 4H), 4.32 (br. t, *J* = 7.3 Hz, 4H), 4.15 (br. t, *J* = 6.6 Hz, 4H), 4.07 – 3.88 (br. m, 4H), 3.02 (q, *J* = 7.3 Hz, 8H), 1.92 (br. s, 12H), 1.34 (br. m, 20H).

^{19}F NMR (470 MHz, $CDCl_3$) δ -86.58, -86.87, -87.16, -87.55, -91.52, -92.73, -93.05, -93.43, -93.90, -147.76, -148.99, -159.99, -161.14.

^{13}C NMR (126 MHz, $CDCl_3$) δ 171.85, 122.44, 66.11, 63.23, 56.17, 51.57, 50.41, 45.99, 30.91, 30.40, 29.04, 28.98, 28.40, 26.53, 25.79, 22.25, 19.15, 9.14.

HRMS (ESI): calculated for $C_{42}H_{62}Br_2FN_6O_6N$ [M•H$^+$]: 925.3061; found: 925.3046

R_f = 0.23 (5% methanol/95% dichloromethane/1% triethylamine). *Preferred Stain: Ceric Ammonium Molybdate*

Synthesis of ^{19}F-Cycloadduct (F-CA)

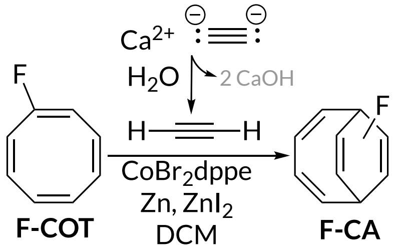

Calcium carbide (43 g, 670 mmol, 32 eq.) was added to a 250-mL 3-necked flask and suspended in chloroform (75 mL). In a separate 25-mL flame dried Schlenk flask, $CoBr_2$(dppe) (2.6 g, 4.2 mmol, 0.2 eq.), zinc dust (827 mg, 13 mmol, 0.60 eq.), and ZnI_2 (2.7 g, 8.4 mmol, 0.40 eq.) were added. The flask was evacuated and refilled with nitrogen 3 times, and the catalyst mixture was suspended in DCE which had previously been sparged with nitrogen for 1 hour (20 mL). The dark green suspension was stirred for 10 minutes, and **F-COT** (3.7 g, 30 mmol, 1.0 eq. [mmol of **F-COT** based on NMR yield of previous step]) was added in one portion *via* syringe as a solution in residual THF from the previous reaction. The calcium carbide suspension was fitted with a hose adapter, which was connected to a drierite tube using Tygon tubing, and the other end of the drierite tube was fitted with more Tygon tubing capped with a long needle. Water (1 mL) was slowly added to the carbide suspension with vigorous stirring until noticeable gas evolution was produced from the needle, purging the system with acetylene. At this point, the nitrogen flow was turned off to seal the **F-COT** reaction mixture under inert atmosphere. The acetylene needle was inserted into the **F-COT** solution, and the reaction vessel was punctured with a needle to sparge the system with acetylene for ~1 minute. The needle was removed and replaced with an empty balloon, and water was added slowly (~1 mL) until the balloon began inflating. More water was added slowly to the carbide solution periodically over the course of 8 hours to ensure that the reaction mixture was continuously sparged with acetylene and that the balloon remained inflated, and the reaction mixture was stirred at room temperature (around 10 mL water was added throughout the day). After 8 hours, 2 mL of water was carefully added to the carbide suspension to inflate the balloon, and the system was left to stir 16 hours at room temperature under a positive pressure of acetylene. The **F-COT** reaction mixture was subsequently poured into diethyl ether (100 mL) to precipitate [Co], and the brown suspension was filtered over neutral alumina. The alumina plug was rinsed with ether (50 mL), and the solvent was removed *in vacuo*. The resulting amber-red oil was filtered over a plug of silica eluting with pentane (200 mL), and the beige filtrate was concentrated to deliver the product **F-CA** as a yellow oil (2.2 g, 69%).

*Characterization Data for **F-CA**:*
^{1}H NMR (500 MHz, $CDCl_3$) δ 6.38 – 6.05 (m, 2H), 6.00 – 5.52 (m, 6H), 3.60 – 3.15 (m, 1H).

^{19}F NMR (470 MHz, $CDCl_3$) δ -77.04, -101.46, -114.90, -146.63.

^{13}C NMR (126 MHz, $CDCl_3$) δ 168.86 (d, *J* = 283.9 Hz), 167.74, 155.15 (d, *J* = 267.9 Hz), 144.24 (d, *J* = 41.7 Hz), 141.86, 141.14, 139.46 (d, *J* = 3.6 Hz), 138.97 (d, *J* = 20.0 Hz), 138.79 (d, *J* = 4.5 Hz), 135.50 (d, *J* = 9.0 Hz), 125.71, 124.44 (d, *J* = 18.8 Hz), 124.27, 122.97, 122.50 (d, *J* = 27.3 Hz), 122.12, 121.93, 121.31, 120.85 (d, *J* = 11.8 Hz), 120.55 (d, *J* = 12.8 Hz), 120.37, 119.17 (d, *J* = 12.6 Hz), 119.02 (d, *J* = 2.6 Hz), 102.55 (d, *J* = 29.2 Hz), 98.74 (d, *J* = 11.7 Hz), 90.89, 89.57, 37.88 (d, *J* = 30.3 Hz), 37.42 (d, *J* = 26.3 Hz), 34.89 (d, *J* = 2.6 Hz), 34.70, 34.18, 33.87 (d, *J* = 6.4 Hz), 29.82 (d, *J* = 10.3 Hz).

Synthesis of ^{19}F-Bullvalene (F-Bull)

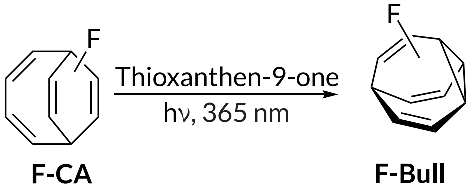

To a vial was added **F-CA** (2.2 g, 15 mmol, 1.0 eq.) and thioxanthene-9-one (160 mg, 0.40 mmol, 3.0 mole%). The materials were dissolved in acetone (22 mL) and the somewhat cloudy yellow solution was filtered through a 0.45 µm PTFE syringe filter. The resulting transparent yellow solution was added to a vial with a stirring bar, and the vial was irradiated with UV light (λ = 365 nm) while stirring. The solution was stirred at RT under UV irradiation for 20 hours. The acetone was removed *in vacuo*, and the crude beige solid was purified *via* flash chromatography using 99% hexanes/1% triethylamine as the mobile phase. The resulting colorless powder was further purified by recrystallization from hexanes (~5 mL solvent for ~1.4 g pre-purified product), delivering the product **F-Bull** as a colorless powder (760 mg, 35%).

Characterization Data for ***F-Bull:***

^{1}H NMR (500 MHz, $CDCl_3$) δ 6.03 – 5.67 (br. m, 6H), 2.41 – 2.24 (br., 3H).

^{19}F NMR (470 MHz, $CDCl_3$) δ -86.54 (br.), -94.03 (br.), -150.19.

^{13}C NMR (126 MHz, $CDCl_3$) δ 131.73 (d, J = 35.4 Hz), 123.21 (d, J = 15.4 Hz), 90.77 (d, J = 162.6 Hz), 20.51. *NMR data is in accordance with literature values*[7]

R_f = 0.50 (99% hexanes/1% triethylamine). *Preferred Stain: Potassium Permanganate*

Synthesis of ^{19}F-Bullvalene Centered poly(methyl acrylate) (F-Bull-PMA)

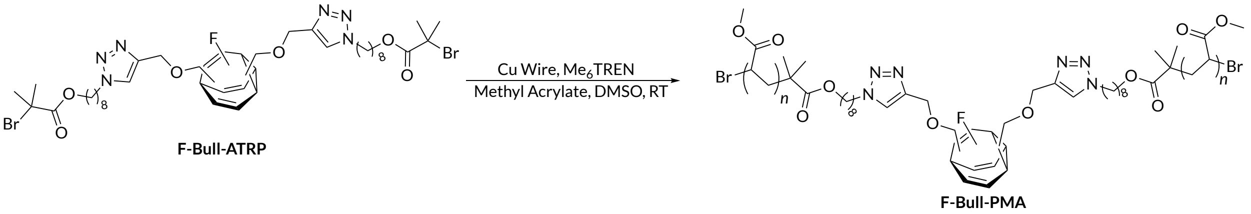

A 25-mL Schlenk flask was charged with a stirring bar and freshly cut copper wire (~2 cm, 14 AWG gauge) and flame dried under high vacuum. The flask was evacuated and backfilled with nitrogen a total of 3 times. Methyl acrylate was filtered over a plug of activated basic alumina to remove inhibitor. **F-Bull-ATRP** (110 mg, 0.10 mmol) and freshly filtered methyl acrylate (5.9 mL, 5.6 g, 65 mmol, 570 eq.) were dissolved in anhydrous DMSO (8.8 mL) and added to the flask *via* syringe. The colorless solution was subjected to 3 freeze-pump-thaw cycles, and after the third cycle the flask was backfilled with nitrogen. To the solution was added Tris[2-(dimethylamino)ethyl]amine (Me$_6$TREN, 60 µL, 53 mg, 0.23 mmol, 2.0 eq.) *via* degasses syringeand the flask was stirred at room temperature. After stirring for 3 hours, the solution had become yellow, and the viscosity had significantly increased. The reaction was quenched by opening it to air and removing the copper wire, and the mixture was diluted into dichloromethane (60 mL). The organic phase was washed with water (3 x 75 mL) to remove DMSO, dried with brine (1 x 50 mL), and the solution was concentrated under reduced pressure to a volume of ca. 25–30 mL. This solution was poured into 250 mL of methanol at –78 °C, and the resulting white goopy precipitate was removed with forceps and squeezed to remove excess solvent. The resulting goopy precipitate was transferred to a vial and dried on high vacuum overnight to deliver the product **F-Bull-PMA** as a colorless, goopy, amorphous solid (2.5 g, 44%). An aliquot of the polymer was redissolved in ethanol-stabilized chloroform and filtered through a 0.22 µm PTFE syringe filter for GPC analysis: M_n = 77 kDa, M_w = 100 kDa, Đ = 1.3, *dn/dc* (100% mass recovery assumed) = 0.0381.

*Characterization Data for **F-Bull-PMA**:*
^{1}H NMR (500 MHz, $CDCl_3$) δ 3.65 (s, 3H), 2.39 – 2.24 (m, 1H), 2.01 – 1.25 (m, 2H).

^{19}F NMR (470 MHz, $CDCl_3$) δ -86.54, -86.86, -87.16, -87.56, -91.34, -93.05, -93.44, -147.71, -148.91, -160.00, -161.13.

Synthesis of 2-Nitrobenzenesulfonylhydrazide (NBSH)

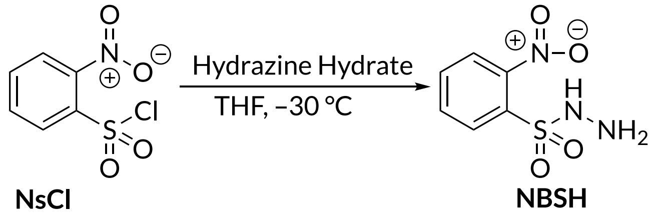

A 50-mL Schlenk flask was charged with a stirring bar and flame-dried under high vacuum. To this flask was added 2-nitrobenzenesulfonyl chloride (NsCl, 6.0 g, 27 mmol, 1.0 eq.). The flask was evacuated and refilled with nitrogen 3 times, and the solid was dissolved in anhydrous THF (27 mL). The flask was cooled to –30 °C (monitor with internal thermometer, dry ice/70% ethylene glycol in ethanol cold bath), and hydrazine monohydrate (3.4 mL, 3.4 g, 68 mmol, 2.5 eq.) was added dropwise, ensuring the internal temperature did not exceed –25 °C. The solution was stirred at –30 °C for 30 minutes, and the reaction was checked by TLC (40% ethyl acetate/hexanes, product R_f = 0.23) for complete consumption of NsCl (starting material R_f = 0.60). Once all the NsCl had been consumed, the suspension was diluted into room temperature ethyl acetate (54 mL) and the organic phase was washed with ice cold 10% (w/v) aqueous

sodium chloride (5 x 40 mL), making sure to keep the organic phase in contact with the aqueous phase for ≤1 minute. The organic phase was dried over ice cold sodium sulfate and decanted slowly into 325 mL of stirring hexanes at RT. The resulting colorless precipitate was collected by filtration, rinsed with hexanes (3 x 20 mL) and dried overnight at RT under high vacuum, delivering the product **NBSH** as a colorless powder (3.6 g, 61%). The product can be stored in a –20 °C freezer under an atmosphere of nitrogen for ~2 weeks with no appreciable loss in potency.

Characterization Data for ***NBSH****:*
^{1}H NMR (500 MHz, CD_3CN) δ 8.11 – 8.03 (m, 1H), 7.91 – 7.78 (m, 3H), 6.91 (br, 1H), 3.99 (br, 2H). *NMR data is in agreement with literature values*[8].

2.2: ULTRASONICATION AND ISOMER TRAPPING EXPERIMENTS

Calibration of Sonicator
The sonicator was calibrated according to the literature method[1]. First, the leads of a K-type thermocouple were placed into a dewar and secured in place with electrical tape. The dewar was tared, deionized water (162.81 g) was poured into it, and the water's mass was recorded. A mark was made on the sonicator at a height of ½" from the bottom of the probe tip. The dewar was fitted to the sonicator with the probe tip lowered until the marked line was just submerged into the water. Once the temperature of the water stabilized, this value was recorded as the temperature for t = 0 seconds and continuous sonication was started (10% amplitude). The water temperature was recorded at 15 second intervals for 3 minutes. The power P of the sonicator (in units W = J • s^{-1}) was determined *via* **Equation S1**:

$$P = c * \frac{dT}{dt} * m \qquad \textbf{(Equation S1)}$$

Where c is the specific heat of water (4.179 J • g^{-1} • $°C^{-1}$), $\frac{dT}{dt}$ is the slope of the trendline relating the change in temperature to the change in time (°C • s^{-1}), and m is the mass of water (g). The intensity I of the sonicator (in units W/cm^2) was subsequently determined by dividing the power by the surface area of the probe tip (1.27 cm^2 for a circular probe tip with ½" diameter, as used herein). This process was repeated at increments of 10% amplitude for all amplitudes up to 100%, and the data was used to construct a calibration curve relating the intensity I to the % amplitude (**Figure 1**).

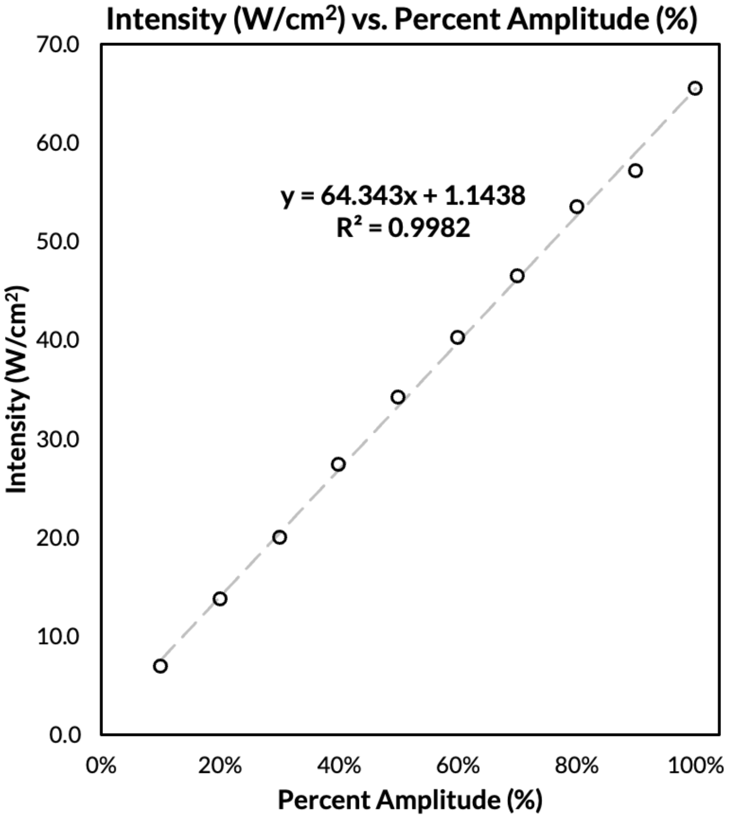

Figure S1: Calibration curve relating sonication intensity (W/cm^2) to % amplitude for the sonication experiments described used herein.

Sonochemical F-Bull-PMA Reduction Experiment (Room Temperature)

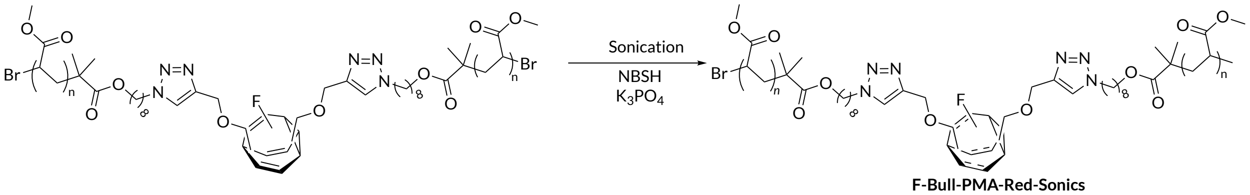

F-Bull-PMA (M_n = 91 kDa, 170 mg, 1.9 μmol, 1.0 eq.) was dissolved in 5 mL of acetonitrile. This solution was sparged, along with blank acetonitrile solvent, with nitrogen for 15 minutes. Separately, a sonication vessel was charged with K_3PO_4 (40 mg, 0.19 mmol, 100 eq.) and the flask was fitted to the sonicator. The vessel was capped with two septa, and one septum was pierced with a nitrogen balloon. Through the other septum was inserted a K-type thermocouple. The phosphate was dissolved in DI water (3 mL), and 21 mL of sparged blank acetonitrile were added to the solution. The sonication vessel was placed in a cold bath with a temperature between ca. –10 °C and –20 °C (dry ice/90% ethylene glycol in ethanol) and continuous sonication was started (20% amplitude, 14.01 W/cm^2). Once the temperature was stable at 22 °C ± 3 °C, the PMA solution was injected to the solution, and NBSH was added (100 mg, 0.50 mmol, 250 eq.). More NBSH was added in 3 additional portions at 24-minute intervals (410 mg total, 1.9 mmol, 1000 eq.), and the solution was continuously sonicated for 2 total hours, adding dry ice to the cold bath as necessary to keep the temperature within the range of 22 °C ± 3 °C. After 2 hours, the solution was diluted into ethyl acetate (30 mL), and the yellow organic phase was washed with saturated aqueous sodium bicarbonate (2 x 75 mL) and water (2 x 75 mL). The washed organic phase was dried with brine (1 x 75 mL) and dried with sodium sulfate, filtered, and concentrated. The crude polymer was redissolved in DCM (~0.5 mL)

and precipitated by the addition of methanol at –78 °C to the solution (1.5 mL). The solvent was decanted, and the residue washed with methanol at –78 °C 3 times. The resulting material was dried on high vacuum overnight, yielding **F-Bull-PMA-Red-Sonics** as a slightly beige goopy solid (73 mg, 43%).

Characterization Data for ***F-Bull-Red-Sonics:***
^{1}H NMR (500 MHz, $CDCl_3$) δ 3.63 (br, 3H), 2.28 (br, 1H), 2.02 – 1.29 (br overlapping peaks, 2H).

^{19}F NMR (470 MHz, $CDCl_3$) δ -92.26, -92.97, -96.37, -108.11, -120.80, -121.59, -121.92, -132.77, -134.32, -135.33, -145.93.

Force-Free ^{19}F-Bull-PMA Reduction Control Experiment (Room Temperature)

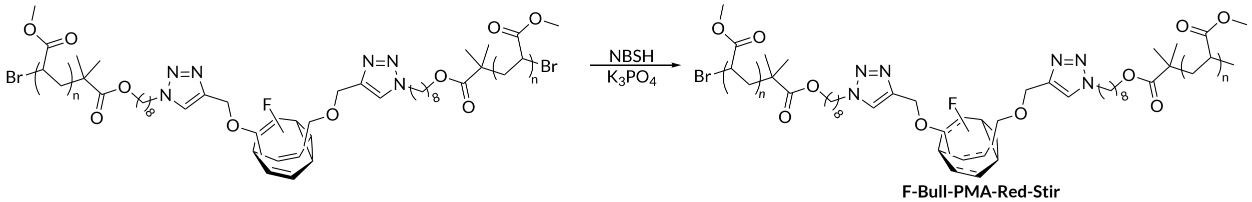

F-Bull-PMA

F-Bull-PMA-Red-Stir

F-Bull-PMA (M_n = 91 kDa, 170 mg, 1.8 µmol, 1.0 eq.) was dissolved in 25 mL of acetonitrile and sparged with nitrogen for 15 minutes. Separately, a 50 mL round-bottomed flask was charged with K_3PO_4 (37.8 mg, 0.18 mmol, 100 eq.) and the phosphate was dissolved in DI water (3 mL). The sparged PMA solution was added to the phosphate solution and NBSH (390 mg, 1.8 mmol, 1000 eq.) was added in one portion. The solution was stirred overnight, and the crude yellow solution was diluted into ethyl acetate (30 mL). The organic phase was washed with saturated aqueous sodium bicarbonate (2 x 75 mL) and water (2 x 75 mL). The washed organic phase was dried with brine (1 x 75 mL) and dried with sodium sulfate, filtered, and concentrated. The crude polymer was redissolved in DCM (~0.5 mL) and precipitated by the addition of methanol at –78 °C to the solution (1.5 mL). The solvent was decanted, and the residue washed with methanol at –78 °C (3x). The resulting material was dried on high vacuum overnight, yielding **F-Bull-PMA-Red-Stir** as a slightly beige goopy solid (78 mg, 47%).

Characterization Data for ***F-Bull-Red-Stir***:
^{1}H NMR (500 MHz, $CDCl_3$) δ 3.63 (s, 3H), 2.29 (s, 1H), 2.15 – 0.98 (m, 2H).

^{19}F NMR (470 MHz, $CDCl_3$) δ -83.70, -92.27, -92.78, -92.97, -93.93, -94.01, -94.20, -94.38, -96.45, -97.44, -107.82, -108.00, -108.11, -120.80, -121.59, -121.90, -129.64, -132.77, -134.32, -134.86, -135.17, -135.39, -145.99.

Sonochemical F-Bull-PMA Reduction Experiment (–7.5 °C)

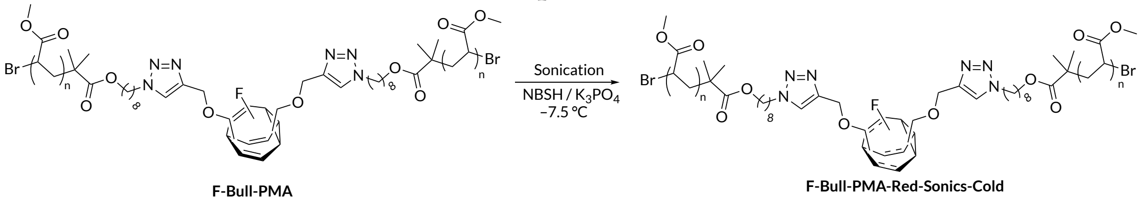

F-Bull-PMA (M_n = 91 kDa, 180 mg, 1.9 µmol, 1.0 eq.) was dissolved in 5 mL of acetonitrile. This solution was sparged, along with blank acetonitrile solvent, with nitrogen for 15 minutes. Separately, a sonication vessel was charged with K_3PO_4 (61 mg, 2.9 mmol, 150 eq.) and the flask was fitted to the sonicator. The vessel was capped with two septa, and one septum was pierced with a nitrogen balloon. Through the other septum was inserted a K-type thermocouple. The phosphate was dissolved in DI water (3 mL), and 13 mL of sparged blank acetonitrile were added to the solution. The sonication vessel was placed in a 2L jacketed beaker connected to a recirculating chiller set to –10 °C. The beaker was filled with a 40% (v/v) solution of ethylene glycol in DI water, and a stirring bar was added. Pulsed sonication (1 s on/11 s off) was started (20% amplitude, 14.01 W/cm^2), and the cooling beaker was stirred to maintain even cooling. Once the internal temperature of the phosphate solution stabilized at –7.5 °C, the PMA solution was injected to the solution, and NBSH was added (624.8 mg, 2.9 mmol, 1,500 eq.) as a solution in sparged acetonitrile (8 mL). After 8 hours of total time (40 minutes sonication time), additional NBSH was added as a solid (62 mg, 0.30 mmol, 150 eq.). The solution was sonicated for 80 more minutes of sonication "on" time (16 additional hours) for a total of 2 hours of sonication "on" time (24 hours total). The resulting yellow solution was diluted into ethyl acetate (30 mL), and the yellow organic phase was washed with saturated aqueous sodium bicarbonate (2 x 75 mL) and water (2 x 75 mL). The washed organic phase was dried with brine (1 x 75 mL) and dried with sodium sulfate, filtered, and concentrated. The crude polymer was redissolved in DCM (~0.5 mL) and precipitated by the addition of methanol at –78 °C to the solution (1.5 mL). The solvent was decanted, and the residue washed with methanol at –78 °C 3 times. The resulting material was dried on high vacuum overnight, yielding **F-Bull-PMA-Red-Sonics-Cold** as a slightly beige goopy solid (110.3 mg, 63%).

Characterization Data for ***F-Bull-Red-Sonics-Cold:***
^{1}H NMR (500 MHz, $CDCl_3$) δ 3.62 (br., 3H), 2.30 – 2.24 (br., 1H), 2.05 – 1.02 (br. overlapping peaks, 2H).

^{19}F NMR (470 MHz, $CDCl_3$) δ -86.25, -86.54, -92.27, -92.48, -92.77, -92.97, -93.42, -93.93, -96.42, -97.41, -107.99, -108.11, -120.80, -121.59, -121.92, -122.22, -122.49, -123.12, -132.77, -133.67, -133.99, -134.34, -134.84, -135.15, -135.36, -145.97, -147.16, -147.38, -159.31.

Force-Free F-Bull-PMA Reduction Control Experiment (–7.5 °C)

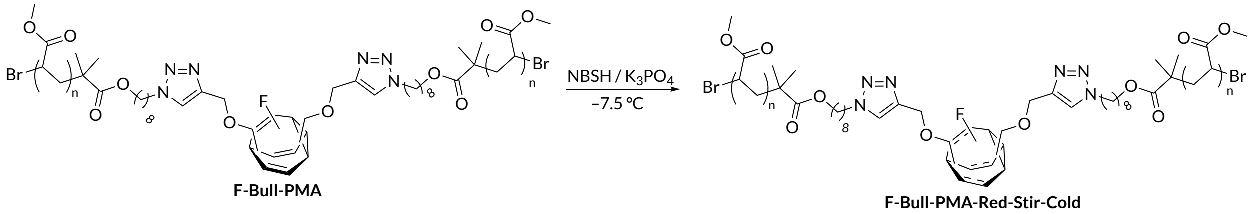

F-Bull-PMA (M_n = 91 kDa, 150 mg, 1.7 μmol, 1.0 eq.) was dissolved in 15 mL of acetonitrile and sparged with nitrogen for 15 minutes. Separately, a 50 mL round-bottomed flask was charged with K_3PO_4 (53 mg, 0.30 mmol, 150 eq.) and the phosphate was dissolved in DI water (3 mL). The sparged PMA solution was added to the phosphate solution and NBSH (540 mg, 2.5 mmol, 1500 eq.) was added in one portion. After stirring for 8 hours, additional NBSH (54 mg, 0.30 mmol, 150 eq.) was added, and the solution was stirred 16 more hours. The crude yellow solution was diluted into ethyl acetate (30 mL), and the organic phase was washed with saturated aqueous sodium bicarbonate (2 x 75 mL) and water (2 x 75 mL). The washed organic phase was dried with brine (1 x 75 mL) and sodium sulfate, filtered, and concentrated. The crude polymer was redissolved in DCM (~0.5 mL) and precipitated by the addition of methanol at –78 °C (1.5 mL). The solvent was decanted, and the residue washed with methanol at –78 °C 3 times. The resulting material was dried on high vacuum overnight, yielding **F-Bull-PMA-Red-Stir-Cold** as a slightly beige goopy solid (81 mg, 53%).

*Characterization Data for **F-Bull-Red-Stir-Cold**:*
^{1}H NMR (500 MHz, $CDCl_3$) δ 3.63 (br, 3H), 2.39 – 2.21 (br, 1H), 2.05 – 1.08 (br m., overlapping peaks 2H).

^{19}F NMR (470 MHz, $CDCl_3$) δ -86.25, -86.53, -92.26, -92.47, -92.76, -92.97, -93.43, -93.93, -96.43, -120.80, -121.59, -121.90, -132.77, -133.67, -133.99, -134.32, -135.37, -145.97, -147.13, -147.39, -148.37, -159.31, -160.42.

Force-Free F-Bullvalene Reduction Experiment, Small Molecule Model Experiment

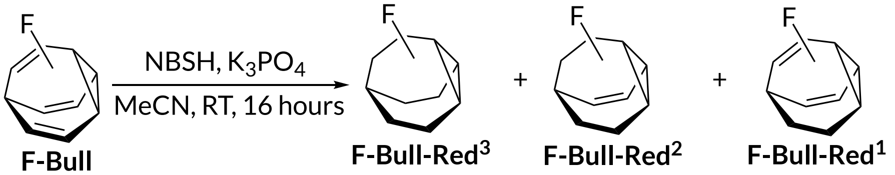

F-Bull (50 mg, 0.30 mmol, 1.0 eq.) and K_3PO_4 (43 mg, 0.20 mmol, 0.60 eq.) were added to a 7-mL vial and dissolved in acetonitrile (5.0 mL). NBSH (440 mg, 2.0 mmol, 6.0 eq.) was added as a solid in one portion, and the suspension was stirred 16 hours. On stirring, the suspension went from colorless to yellow then to orange. The suspension was filtered over celite, and the residue was rinsed with ethyl acetate (3 x 3 mL). The filtrate was collected and washed with saturated aqueous sodium bicarbonate (3 x 25 mL), and the organic phase was dried with brine (1 x 25 mL) and sodium sulfate. The yellow organic phase was filtered and concentrated *in vacuo*, and the crude yellow oil was diluted over a plug of silica eluting with hexanes (50 mL). The resulting colorless filtrate was collected and concentrated *in vacuo*, delivering the product **F-Bull-Red^n** as a beige oil consisting of a mixture of bullvalene species at various stages of reduction (33 mg, 63%).

Characterization Data for ***F-Bull-Red^n*:**
^{1}H NMR (500 MHz, $CDCl_3$) δ 5.95 – 5.84 (overlapping peaks, 5H), 5.76 – 5.70 (overlapping peaks, 2H), 5.69 – 5.57 (overlapping peaks, 3H), 3.79 – 3.69 (overlapping peaks, 2H), 2.40 – 1.77 (overlapping peaks, 78H), 1.35 – 0.97 (overlapping peaks, 12H).

^{19}F NMR (470 MHz, $CDCl_3$) δ -97.72, -110.09, -124.00, -135.98.

^{13}C NMR (126 MHz, $CDCl_3$) δ 166.51 (d, J = 257.0 Hz), 134.93 (d, J = 36.3 Hz), 132.21 (d, J = 35.4 Hz), 124.69 (d, J = 17.3 Hz), 122.93 (d, J = 16.8 Hz), 100.42 (d, J = 22.7 Hz), (96.64 d, J = 166.2 Hz), (95.87 d, J = 165.3 Hz), 93.78 (d, J = 163.9 Hz), 68.11, 43.58, (34.54 d, J = 25.5 Hz), (34.39 d, J = 25.5 Hz), 31.05, 30.87 (d, J = 25.4 Hz), 29.84, 28.65, 27.40, 26.76, 26.00 (d, J = 2.7 Hz), 25.75, 23.56, 22.41, 20.94 (d, J = 14.1 Hz), 20.29 (d, J = 5.0 Hz), 19.19 (d, J = 13.2 Hz), 16.80, 16.48, 16.31, 15.44.

GC-MS (EI): Calculated for $C_{10}H_{15}F$ [**F-Bull-Red³**, $M^{\bullet+}$]: 154.2; found: 154.2
GC-MS (EI): Calculated for $C_{10}H_{13}F$ [**F-Bull-Red²**, $M^{\bullet+}$]: 152.2; found: 152.1 ***[There are two species in the GC-MS with this m/z value]***
GC-MS (EI): Calculated for $C_{10}H_{11}F$ [**F-Bull-Red¹**, $M^{\bullet+}$]: 150.2; found: 149.1

Assignment of Isomers in F-Bull-Red^n
The ^{19}F NMR spectrum (**Figure S44**) indicates the presence of 4 major species following reduction. The assignment of these species as representing bullvalene isomers that have been mono-, di- or tri-reduced was made on the basis of GC-MS data (**Figures S87-S90**). The assignment of the ^{19}F atom to the bridgehead position in these reduced species was made on the basis of ^{13}C NMR data and ^{13}C DEPT-135 data (**Figures S74-S77**). First, the bridgehead carbons were identified based on the literature values (ca. 95-100 ppm)[7]. Notably, there are 3 clear signals in this region, corresponding to the 3 main signals observed in the ^{19}F NMR spectrum. Furthermore, each of these carbons has a large coupling constant of ca. 160 Hz, characteristic of a $^1J_{C\text{-}F}$ coupling (**Figure S75**). Additionally, these signals are absent in the DEPT-135 NMR, and are the only signals to disappear during this experiment (**Figures S76**), confirming that the bridgehead position bears a quaternary carbon in all 3 species. Since the ^{19}F atom is the only substituent in the small-molecule model, these findings confirm that the 3 major signals observed in the ^{19}F NMR spectrum are arising exclusively from bridgehead fluorinated systems at differing levels of reduction.

Additionally, the presence of CH_2 signals in the 20-40 ppm range with coupling constants of 25-30 Hz, which correspond to aliphatic groups alpha to the ^{19}F substituent, are a good indication that the major species are arising from reduction of π-bonds alpha to the fluorine atom (**Figure S77**). This, coupled with the fact that the cyclopropyl resonances at <20 ppm are CH groups (and are therefore unfunctionalized), provides additional support for the assignment of the fluorine substituent to the bridgehead position.

There is one additional minor peak (<5% relative integration) in the ^{19}F spectrum at δ = –98 ppm, indicating a C_{sp2}-F bond (**Figure S44**). This resonance therefore indicates the presence of an unreduced vinyl fluoride in a partially reduced species; this assignment is further corroborated by the presence of a small signal in the ^{13}C spectrum at δ = 167 ppm which bears a strong $^1J_{C\text{-}F}$ coupling (J = 250 Hz, **Figure S74**). This signal is too low in intensity to appear in the DEPT-135 experiments; its low intensity suggests it likely arises from the same species as the minor signal in the ^{19}F spectrum (**Figure S44**). Analysis of the GC-MS spectrum indicates the presence of two isomers with identical m/z = 152.2, corresponding to the di-reduced species **F-Bull-Red²**. We thus attributed this minor isomer to a vinyl-fluoride in which the other two, more reactive π-bonds have been fully reduced.

Figure S2 shows the proposed structures of the reduced bullvalene products, as well as the potential structures of other isomers which are **not** observed. The chemical shifts and relative proportions of each isomer, as extracted from ^{19}F NMR analysis, are reported, and spectral features from the DEPT-135 experiment which were used to assign structures are indicated.

Thus, the 3 main signals in the ^{19}F spectrum represent bridgehead-substituted species at varying levels of reduction, and the fourth, minor signal in the ^{19}F spectrum represents a vinyl fluoride wherein the other two π-bonds have been reduced. The assignment of the remaining 3 peaks to the mono-, di-, or tri-reduced species were made based on changes in the relative intensities of each respective peak in response to increased loadings of the reducing agent **NBSH**. Furthermore, there is some literature precedent indicating that the ^{19}F shifts of allylic fluorides shift to less negative chemical shifts following saturation of the π-bond. For instance, the ^{19}F signal for 1-fluoroindene appears at –201 ppm[9], whereas the corresponding indane features a resonance at –160 ppm[10]. Additionally, 1-fluorocyclooct-2-ene has a resonance at –173 ppm[9,11] compared to –160 ppm for fluorocyclohexane[12] **(Figure S3)**.

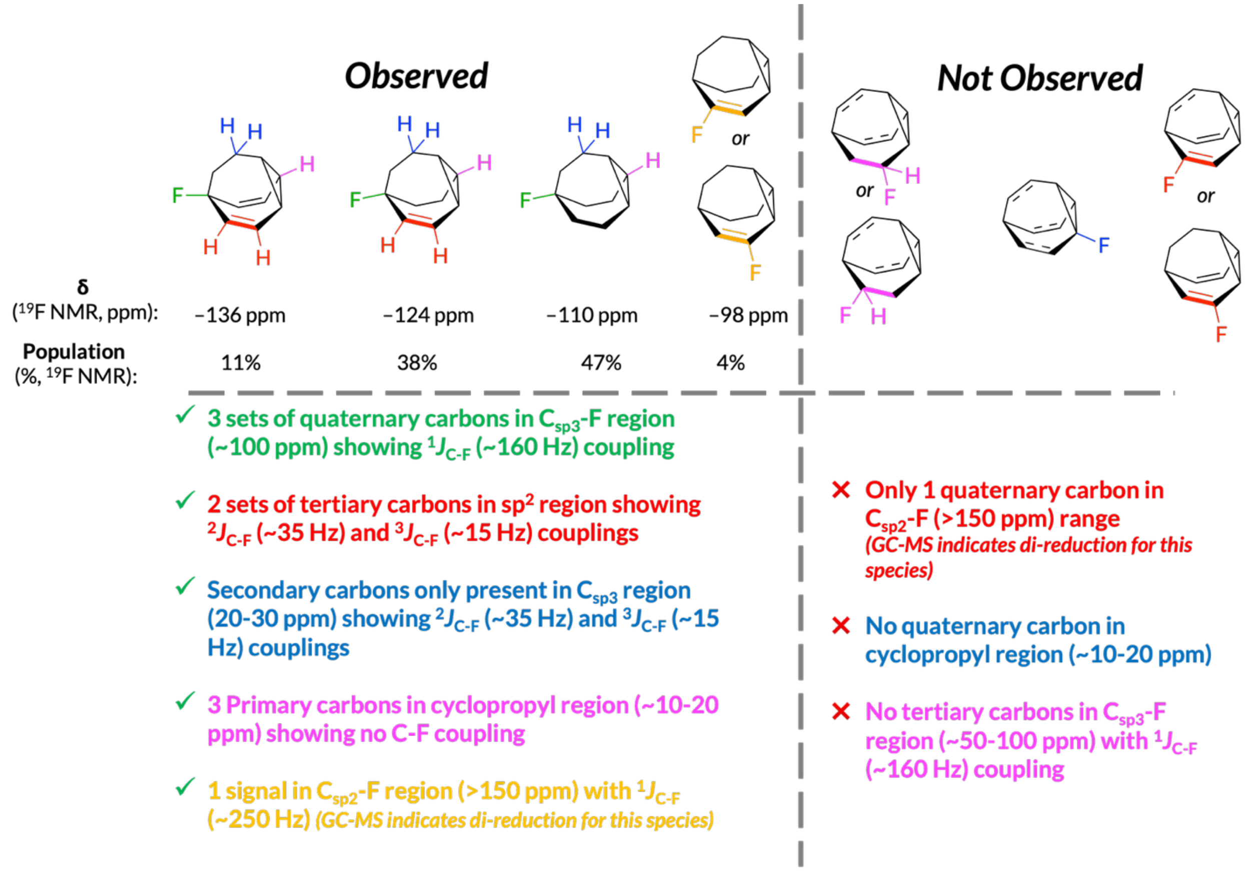

Figure S2: Schematic showing possible reduction products expected *via* reduction of **F-Bull**. Spectral features from the ^{13}C DEPT-135 experiments which support fluorination predominately at the bridgehead position are highlighted.

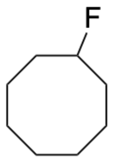

–159.7 ppm

A. L'Heureux, F. Beaulieu, C. Bennett, D. R. Bill, S. Clayton, F. LaFlamme, M. Mirmehrabi, S. Tadayon, D. Tovell and M. Couturier, *J. Org. Chem.*, 2010, **75**, 3401–3411.

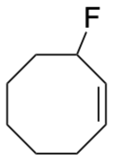

–173.4 ppm

1) G. Haufe, G. Alvernhe, D. Anker, A. Laurent and C. Saluzzo, *J. Org. Chem.*, 1992, **57**, 714–719.

2.) S. Bloom, J. L. Knippel, M. G. Holl, R. Barber and T. Lectka, *Tetrahedron Lett.*, 2014, **55**, 4576–4580.

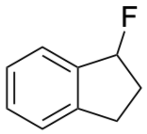

–159.9 ppm

A. Vasilopoulos, D. L. Golden, J. A. Buss and S. S. Stahl, *Org. Lett.*, 2020, **22**, 5753–5757.

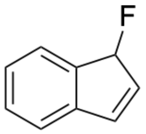

–201.0 ppm

S. Bloom, J. L. Knippel, M. G. Holl, R. Barber and T. Lectka, *Tetrahedron Lett.*, 2014, **55**, 4576–4580.

Figure S3: Precedent showing shifts to more positive (less negative) chemical shifts following π-bond reduction in some allylic fluorides.

F-Bull-PMA Isomer Trapping *via* Bromination Experiment

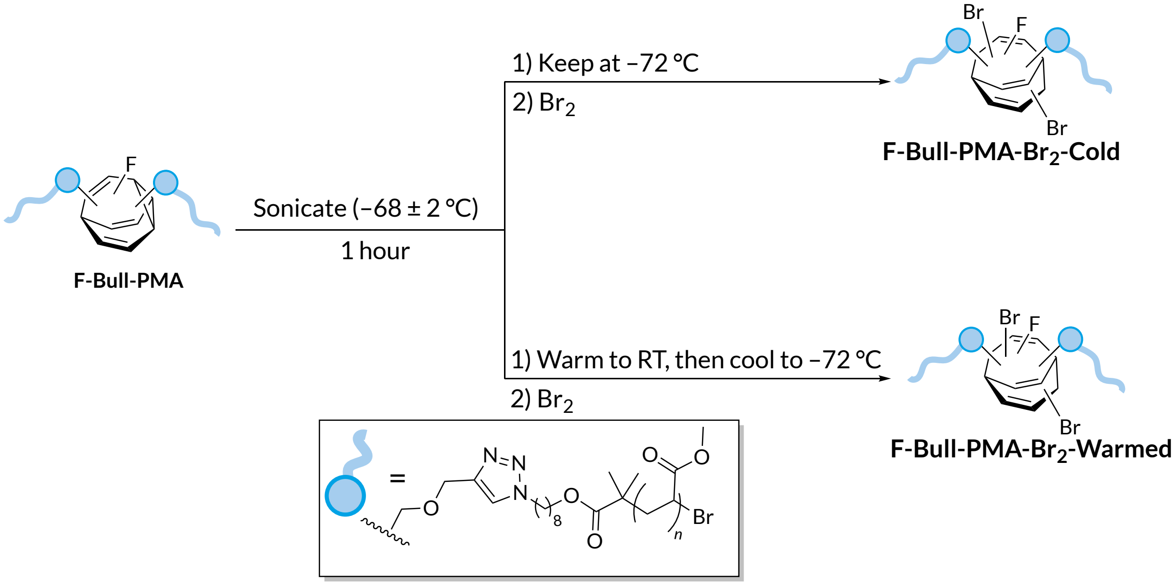

F-Bull-PMA (M_n = 77 kDa, 300 mg, 3.9 µmol, 1.0 eq.) was dissolved in diethyl carbonate (49 mL, ca. 6 mg/mL) and transferred to a sonication vessel. The mass of the solution (48 g) was recorded, and the flask was fitted with a glass stopper and septum. The septum was pierced with a K-type thermocouple lead to monitor the internal temperature of the solution, and the flask was fitted to the sonicator. The solution was cooled in a dry ice/acetone bath until the temperature stabilized at –68 °C, and pulsed sonication (0.5 s on/3.5 s off, 15% amplitude, 10.8 W/cm^2) was started. Sonication was continued for 1 hour of total sonication "on" time (8 hours experiment time), and dry ice was periodically added to the cooling dewar to ensure the internal temperature remained within the range of –68 °C ± 2 °C. After 1 hour of sonication "on" time, the contents of the flask were poured into a 100 mL round bottomed flask with stirring bar which had been precooled on a –78 °C cooling bath (internal temperature = –72 °C) for one hour and was covered in aluminum foil to exclude light. Half of the solution (24 g) was removed from this flask *via* pipette and transferred to a tared beaker, measuring the amount removed gravimetrically. To the remaining solution in the cooling bath was immediately added a solution of bromine in anhydrous dichloromethane (720 µL of a 5.7 mg/mL solution, 4.1 mg, 26 µmol, 13 eq.) in one portion, and the resulting solution was stirred for one hour. The amount of time elapsed from the end of sonication to complete addition of bromine was measured to be ca. 3 minutes. The solution which had been removed from the round-bottom flask and transferred to a beaker was subsequently transferred to a 100-mL RBF with stirring bar which was covered with aluminum foil to exclude light. The internal temperature of the solution was monitored, and the solution was gradually allowed to warm to RT by placing it in a room-temperature water bath. Once the internal temperature reached 21 °C, the flask was left to sit for an additional ca. 5 minutes at this temperature to allow the bullvalene isomer mixture to re-equilibrate. The flask was subsequebtly submerged in a dry ice/acetone bath and stirred for 15 minutes to allow the solution temperature to equilibrate. To the cooled solution was added a solution of bromine in anhydrous dichloromethane (720 µL of a 5.7 mg/mL solution, 4.1 mg, 26 µmol, 13 eq.) in one portion, and the resulting solution was stirred for one hour. Both of the resulting red-orange solutions were worked up separately by diluting with ethyl acetate (30 mL), washing subsequently with saturated aqueous sodium thiosulfate (1 x 40 mL), saturated aqueous sodium bicarbonate (1 x 40 mL), and brine (1 x 40 mL). The washed organic phases were dried over sodium sulfate, filtered, and concentrated to a volume of ca. 0.5–1.0 mL. The resulting oils were triturated by the addition of methanol at –78 °C (2 mL), the supernatants decanted, and the materials were dried on high vacuum overnight to deliver the products **F-Bull-PMA-Br$_2$-Cold** (120 mg, 77%) and **F-Bull-PMA-Br$_2$-Warmed** (110 mg, 72%) as colorless, goopy solids.

Characterization data for ***F-Bull-PMA-Br$_2$-Cold:***
^{1}H NMR (500 MHz, $CDCl_3$) δ 3.61 (br, 3H), 2.37 – 2.18 (br, 1H), 1.97 – 0.89 (br. overlapping peaks, 2H).

^{19}F NMR (470 MHz, $CDCl_3$) δ -73.61, -75.64, -76.15, -76.73, -77.38, -77.71, -77.96, -78.07, -78.25, -79.65, -86.84, -91.87, -92.09, -92.16, -92.43, -92.62, -92.74, -93.81, -96.25, -96.67, -97.25, -97.71, -97.96, -123.41, -123.77, -124.20, -124.69, -128.39, -128.77, -129.03, -129.39, -129.43, -129.49, -129.64, -129.97, -130.05, -130.15, -130.26, -130.36, -130.55, -130.60, -131.19, -135.17, -144.27.

GPC: M_n = 57 kDa, M_w = 64 kDa, Đ = 1.1, *dn/dc* = 0.0381

Characterization data for ***F-Bull-PMA-Br$_2$-Warmed:***
^{1}H NMR (500 MHz, $CDCl_3$) δ 3.66 (br, 3H), 2.55 – 2.09 (br, 1H), 1.97 – 0.89 (br. overlapping peaks 2H).

^{19}F NMR (470 MHz, $CDCl_3$) δ -75.64, -76.16, -76.74, -77.38, -77.71, -77.96, -78.08, -78.25, -79.65, -91.87, -92.16, -92.43, -92.62, -92.74, -93.14, -93.81, -96.25, -96.68, -97.25, -97.71, -97.96, -108.99, -123.42, -123.77, -124.20, -124.69, -128.78, -129.03, -129.43, -129.69, -130.05, -130.16, -130.28, -130.36, -130.86, -131.20, -135.18, -137.96, -140.20, -142.50, -142.89, -144.28, -144.41.

GPC: M_n = 60 kDa, M_w = 68 kDa, Đ = 1.1, *dn/dc* = 0.0381

Force Free F-Bull-PMA Isomer Trapping *via* Bromination Experiment

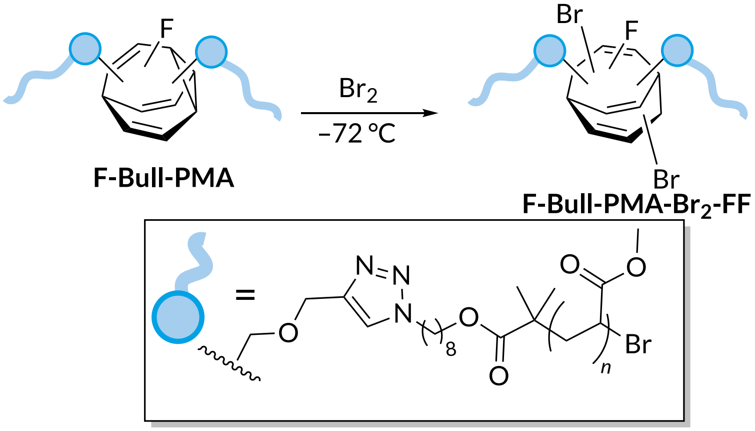

F-Bull-PMA (M_n = 77 kDa, 130 mg, 1.7 µmol, 1.0 eq.) was added to a 50-mL round-bottomed flask with stirring bar and dissolved in diethyl carbonate (22 mL). The flask was covered in aluminum foil to exclude light and stirred at –78 °C for 15 minutes. Bromine in anhydrous dichloromethane (600 µL of a 6.0 mg/mL solution, 3.6 mg, 23 µmol, 13.0 eq.) was added in one portion. The solution was stirred for one hour, and the orange-red solution was diluted in ethyl acetate (50 mL) and added to a separatory funnel. The organic phase was washed sequentially with saturated aqueous sodium thiosulfate (2 x 75 mL), saturated aqueous sodium bicarbonate (1 x 75 mL), and brine (1 x 75 mL). The resulting colorless solution was dried over sodium sulfate and concentrated to a volume of ca. 0.5–1.0 mL. The resulting oil was triturated by the addition of methanol at –78 °C (2 mL), the supernatant was decanted, and the material were dried on high vacuum overnight to deliver the product **F-Bull-PMA-Br$_2$-FF** (120 mg) as a colorless, goopy solid. The solid was redissolved in ethanol-stabilized chloroform and filtered through a 0.22 µm PTFE syringe filter for GPC analysis: M_n = 88 kDa, M_w = 112 kDa, Đ = 1.3, *dn/dc* (100% mass recovery assumed) = 0.0381.

Characterization Data for ***F-Bull-PMA-Br$_2$-FF:***
^{1}H NMR (500 MHz, $CDCl_3$) δ 3.67 – 3.63 (br, 3H), 2.39 – 2.19 (br, 1H), 2.03 – 1.38 (br overlapping peaks, 2H).

^{19}F NMR (470 MHz, $CDCl_3$) δ -75.67, -75.92, -76.18, -76.77, -77.37, -86.86, -91.87, -92.08, -92.16, -92.43, -92.73, -93.15, -93.43, -93.82, -96.25, -96.67, -97.24, -97.70, -123.80, -128.79, -129.05, -129.43, -129.46, -129.66, -129.71, -130.08, -130.18, -130.28, -130.39, -142.61, -144.32, -144.45.

Force Free F-Bull-ATRP Isomer Trapping *via* Bromination Small Molecule Model Experiment

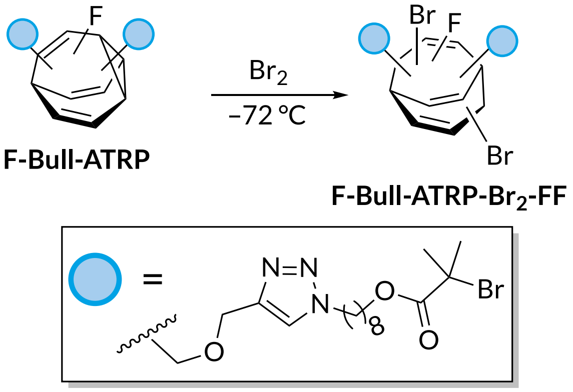

F-Bull-ATRP (50 mg, 0.050 mmol, 1.0 eq.) was added to 250 mL round-bottomed flask with a stirring bar and dissolved in diethyl carbonate (100 mL). The flask was covered in aluminum foil to exclude light and stirred at –78 °C for 15 minutes. Bromine in anhydrous dichloromethane (660 µL of 170 mg/mL solution, 110 mg, 0.70 mmol, 13 eq.) was added in one portion. The solution was stirred for one hour, and the orange-red solution was diluted in ethyl acetate (50 mL) and added to a separatory funnel. The organic phase was washed sequentially with saturated aqueous sodium thiosulfate (2 x 75 mL), saturated aqueous sodium bicarbonate (1 x 75 mL), and brine (1 x 75 mL). The resulting colorless solution was dried over sodium sulfate and concentrated under reduced pressure, delivering the product **F-Bull-ATRP-Br$_2$-FF** as a brownish-yellow oil (55 mg, 94%). The crude product was directly analyzed by ^{1}H NMR spectroscopy, ^{19}F NMR spectroscopy, and HRMS (ESI).

*Characterization Data for **F-Bull-ATRP-Br$_2$-FF**:*
^{1}H NMR (500 MHz, $CDCl_3$) δ 7.59 – 7.48 (m, 2H), 5.72 – 5.44 (m, 3H), 4.69 – 4.49 (m, 5H), 4.35 – 3.96 (m, 21H), 3.89 – 3.37 (m, 12H), 2.11 – 1.71 (m, 9H), 1.54 – 0.89 (m, 58H).

^{19}F NMR (470 MHz, $CDCl_3$) δ -75.61, -76.13, -76.78, -77.37, -77.70, -77.96, -78.32, -91.87, -92.16, -92.42, -92.73, -93.79, -96.27, -96.67, -97.24, -97.70, -97.94, -107.33, -108.00, -108.96, -111.13, -117.14, -123.39, -123.75, -124.19, -124.70, -127.01, -128.75, -129.05, -129.37, -129.40, -130.03, -130.24, -130.25, -130.41, -130.69, -130.76, -131.16, -135.16, -135.28, -136.64, -137.93, -140.18, -142.45, -142.53, -144.28, -144.31.

HRMS (ESI): Calculated for $C_{42}H_{62}Br_4FN_6O_6$ [M•H$^+$]: 1,085.1409; found: 1,085.1416
HRMS (ESI): Calculated for $C_{42}H_{62}Br_3ClFN_6O_6$ [M•H$^+$, –Br +Cl]: 1,041.1913; found: 1,041.1915
HRMS (ESI): Calculated for $C_{44}H_{67}Br_3FN_6O_7$ [M•H$^+$, –Br +OEt]: 1,051.2569; found: 1,051.2552

Sonochemical F-Bull-ATRP Isomer Trapping *via* Bromination Small Molecule Control Experiment

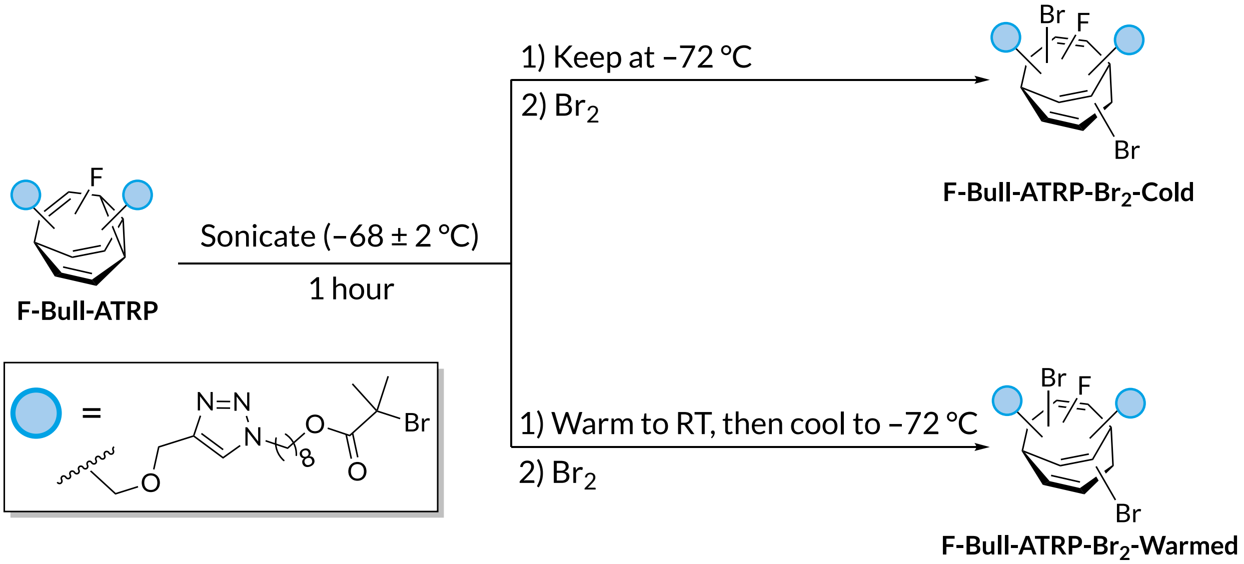

F-Bull-ATRP (96 mg, 0.10 mmol, 1.0 eq.) was added to a sonication vessel and dissolved in diethyl carbonate (16 mL, ca. 6 mg/mL). The mass of the solution (13 g) was recorded, and the flask was capped with a glass stopper and rubber septum. The septum was pierced with a K-type thermocouple to monitor the internal temperature, and the flask was fitted to the sonication vessel. The flask was submerged into a dry ice/acetone cold bath, and once the temperature reached –68 °C, pulsed sonication was started (0.5 s on/3.5 s off, 15% amplitude, 10.8 W/cm^2). Dry ice was added to the bath to ensure the internal solution temperature remained in the range –68 ± 2 °C. After 1 hour of sonication "on" time (8 hours total time), the contents of the flask were poured into a 25 mL RBF with stirring bar which had been covered with aluminum foil and pre-cooled to –78 °C (internal temperature = –72 °C)for one hour. Half of the solution (6.30 g) was transferred to a tared beaker *via* pipette, and to the remaining solution was added bromine as a solution in anhydrous dichloromethane (658 µL of a 164.5 mg/mL solution, 108.2 mg, 0.7 mmol, 13.1 eq.) in one portion. The flask was stirred at –78 °C for one hour. The other half of the solution was transferred to a 25 mL RBF with stirring bar, and the flask was covered with aluminum foil. The internal temperature of the solution was monitored, and the solution was gradually allowed to warm to RT by placing it in a room-temperature water bath. Once the internal temperature reached 21 °C, the flask was left to sit for an additional ca. 5 minutes at this temperature to allow the bullvalene isomer mixture to re-equilibrate. The flask was subsequebtly submerged in a dry ice/acetone bath and stirred for 15 minutes to allow the solution temperature to equilibrate. Bromine was subsequently added as a solution in dichloromethane (658 µL of a 164.5 mg/mL solution, 108.2 mg, 0.7 mmol, 13.0 eq.) in one portion. The solution was allowed to stir at –78 °C for one hour. Both solutions were worked up separately by diluting into ethyl acetate (10 mL) and washing sequentially with saturated aqueous sodium thiosulfate (1 x 25 mL), saturated aqueous sodium bicarbonate (1 x 25 mL), and brine (1 x 25 mL). The organic phases were dried over sodium sulfate, filtered, and concentrated under reduced pressure to deliver the crude products **F-Bull-ATRP-Br$_2$-Cold** (95 mg) and **F-Bull-ATRP-Br$_2$-Warmed** (50 mg) as beige oils.

Characterization data for ***F-Bull-ATRP-Br$_2$-Cold:***
^{1}H NMR (500 MHz, $CDCl_3$) δ 7.61 – 7.46 (overlapping peaks, 2H), 6.31 – 5.45 (overlapping peaks, 4H), 4.93 – 3.84 (overlapping peaks, 26H), 2.39 – 2.15 (overlapping peaks, 11H), 1.90 (s, 19H), 1.78 – 0.79 (overlapping peaks, 62H).

^{19}F NMR (470 MHz, $CDCl_3$) δ -73.56, -77.38, -77.71, -78.26, -83.55, -88.49, -90.16, -92.10, -92.63, -92.73, -93.82, -96.67, -97.14, -97.70, -97.96, -107.38, -123.43, -124.23, -128.80, -129.04, -129.45, -129.53, -130.07, -130.11, -130.29, -130.37, -130.45, -130.69, -130.89, -131.01, -131.17, -143.37, -144.32.

Characterization data for ***F-Bull-ATRP-Br$_2$-Warmed:***
^{1}H NMR (500 MHz, $CDCl_3$) δ 7.64 – 7.47 (overlapping peaks, 2H), 6.26 – 5.47 (overlapping peaks, 2H), 5.08 – 3.10 (overlapping peaks, 27H), 1.91 (s, 13H), 1.85 – 0.63 (overlapping peaks, 43H).

^{19}F NMR (470 MHz, $CDCl_3$) δ -73.54, -75.62, -76.13, -76.74, -77.38, -77.99, -78.26, -83.55, -88.47, -90.14, -92.09, -92.14, -92.62, -92.72, -93.82, -96.66, -97.04, -97.10, -97.15, -97.69, -97.96, -98.26, -98.34, -107.40, -123.45, -124.24, -128.82, -129.08, -129.51, -129.68, -130.08, -130.47, -130.77, -131.19, -131.71, -132.54, -142.49, -142.90, -143.38, -144.09, -144.25, -144.33.

F-Bull-PMA Sonication Stability Control

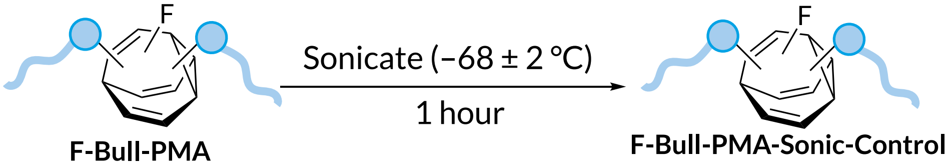

F-Bull-PMA (M_n = 77 kDa, 153 mg, 2.0 µmol, 1.0 eq.) was dissolved in diethyl carbonate (25 mL) and transferred to a sonication vessel. The flask was fitted with a glass stopper and septum, and the septum was pierced with a K-type thermocouple lead to monitor the internal temperature of the solution. The flask was fitted to the sonicator, and the solution was cooled in a dry ice/acetone bath until the temperature stabilized at –68 °C. Pulsed sonication (0.5 s on/3.5 s off, 15% amplitude, 10.8 W/cm^2) was started. Sonication was continued for 1 hour of total sonication "on" time (8 hours experiment time), and dry ice was periodically added to the cooling dewar to ensure the internal temperature remained within the range of –68 °C ± 2 °C. After 1 hour of sonication "on" time, the solution was diluted with ethyl acetate (30 mL) and washed subsequently with saturated aqueous sodium thiosulfate (1 x 25 mL), saturated aqueous sodium bicarbonate (1 x 25 mL), and brine (1 x 25 mL). The washed organic phase was dried over sodium sulfate, filtered, and concentrated to a volume of ca. 0.5–1.0 mL. The resulting oil was triturated by the addition of methanol at –78 °C (2 mL), the supernatant was decanted, and the material was dried on high vacuum overnight to deliver the product **F-Bull-PMA-Sonics-Control** (75 mg, 49%) as a colorless, goopy solid.

Characterization Data for ***F-Bull-PMA-Sonics-Control****:*
^{1}H NMR (500 MHz, $CDCl_3$) δ 3.81 – 2.89 (br, 3H), 2.59 – 1.98 (br., 1H), 2.13 – 0.93 (br. overlapping peaks, 2H).

^{19}F NMR (470 MHz, $CDCl_3$) δ -82.52, -86.55, -86.84, -87.13, -87.53, -91.29, -93.04, -93.42, -147.69, -148.94, -159.92, -161.06.

GPC: M_n = 48 kDa, M_w = 53 kDa, Đ = 1.1 *dn/dc* = 0.0381

3.0: SPECTROSCOPIC AND CHROMATOGRAPHIC DATA

3.1: ¹H NMR SPECTRA

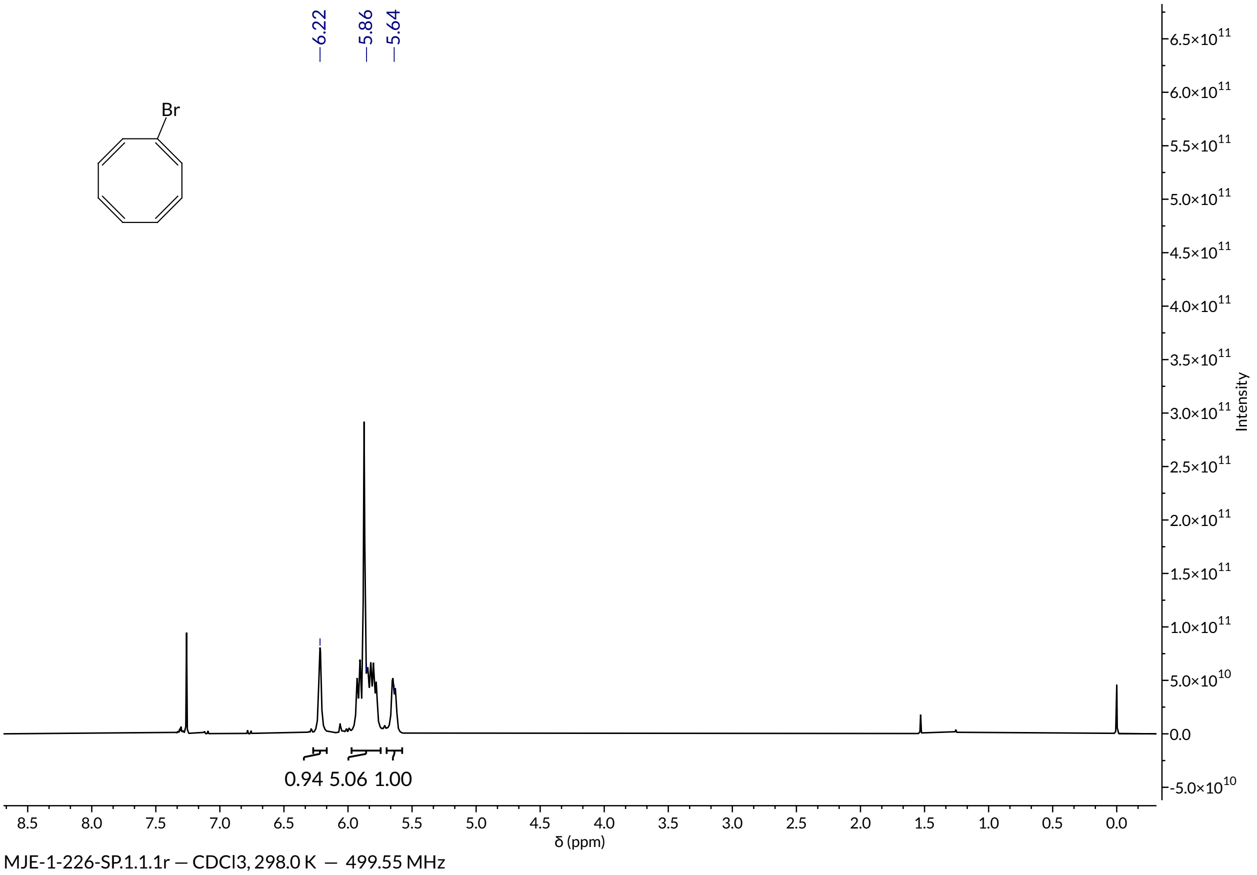

Figure S4: ^{1}H NMR spectrum of **Br-COT** ($CDCl_3$, 500 MHz)

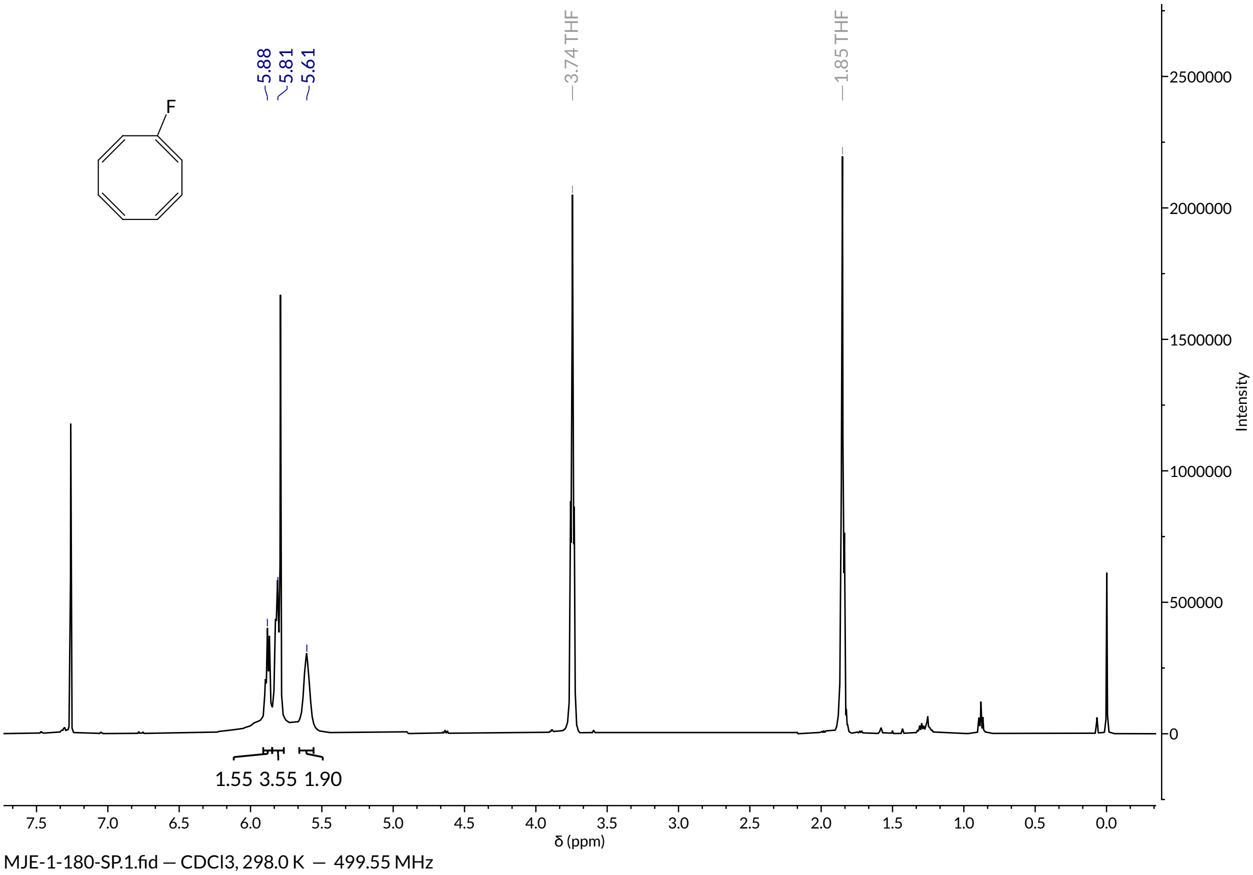

Figure S5: ^{1}H NMR spectrum of **F-COT** ($CDCl_3$, 500 MHz)

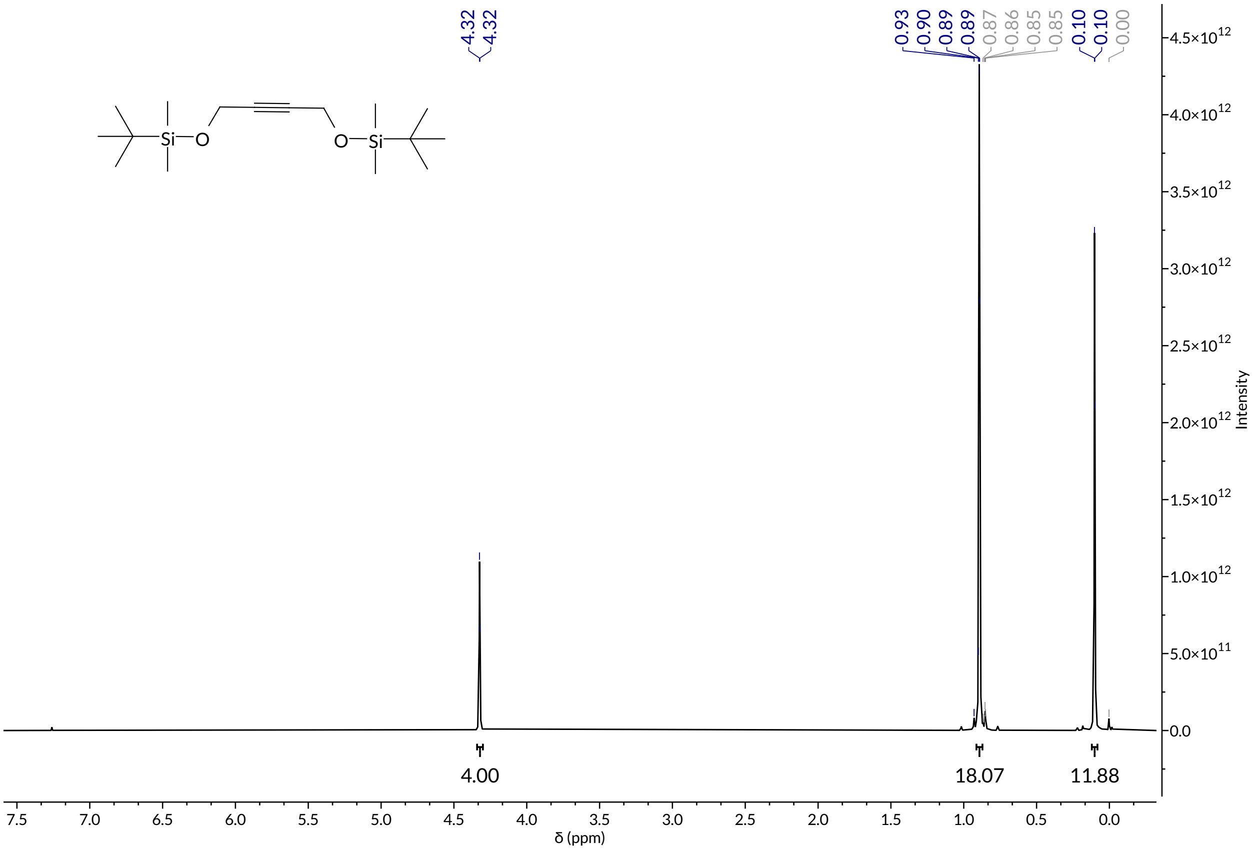

Figure S6: ^1H NMR spectrum of **TBDMSO-Alkyne** ($CDCl_3$, 500 MHz)

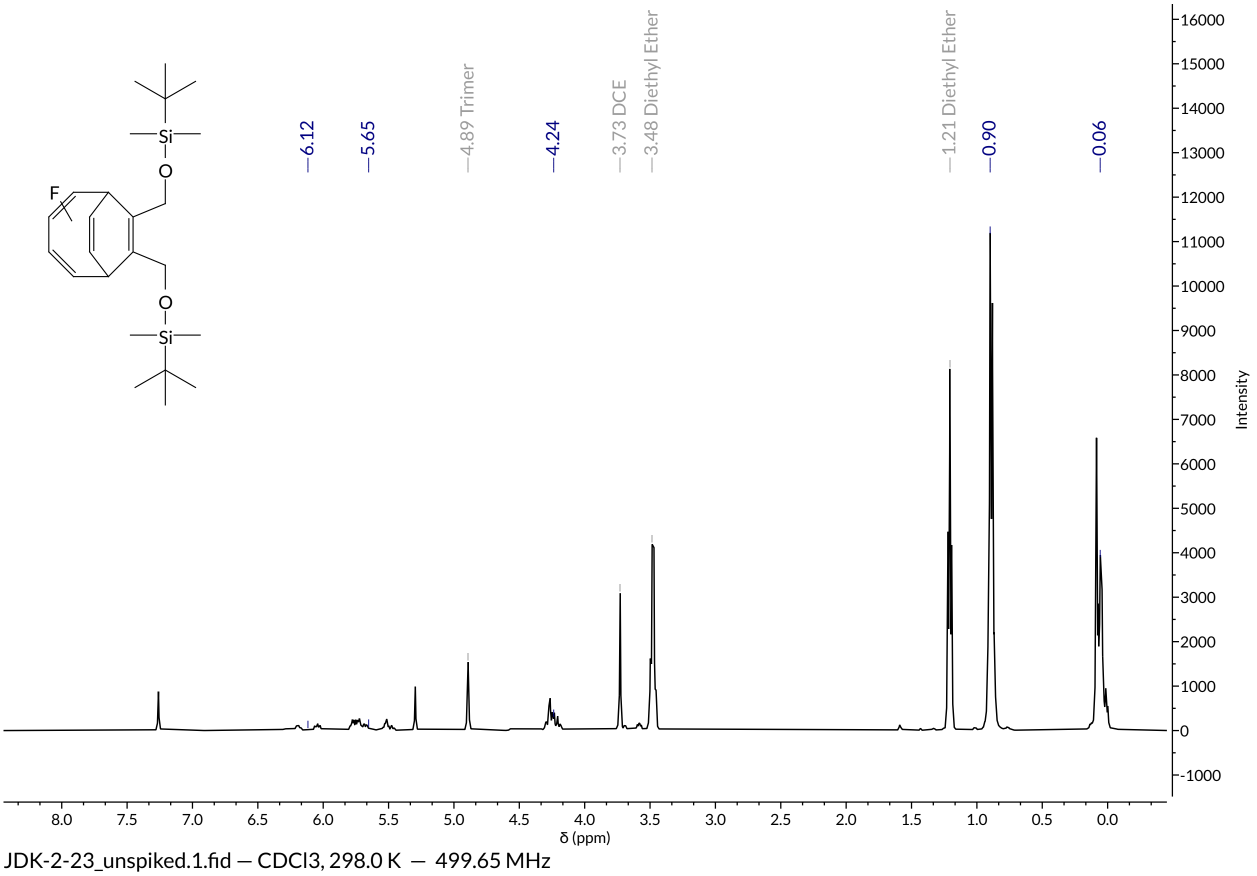

Figure S7: ^{1}H NMR spectrum of crude **F-CA-OTBDMS** ($CDCl_3$, 500 MHz)

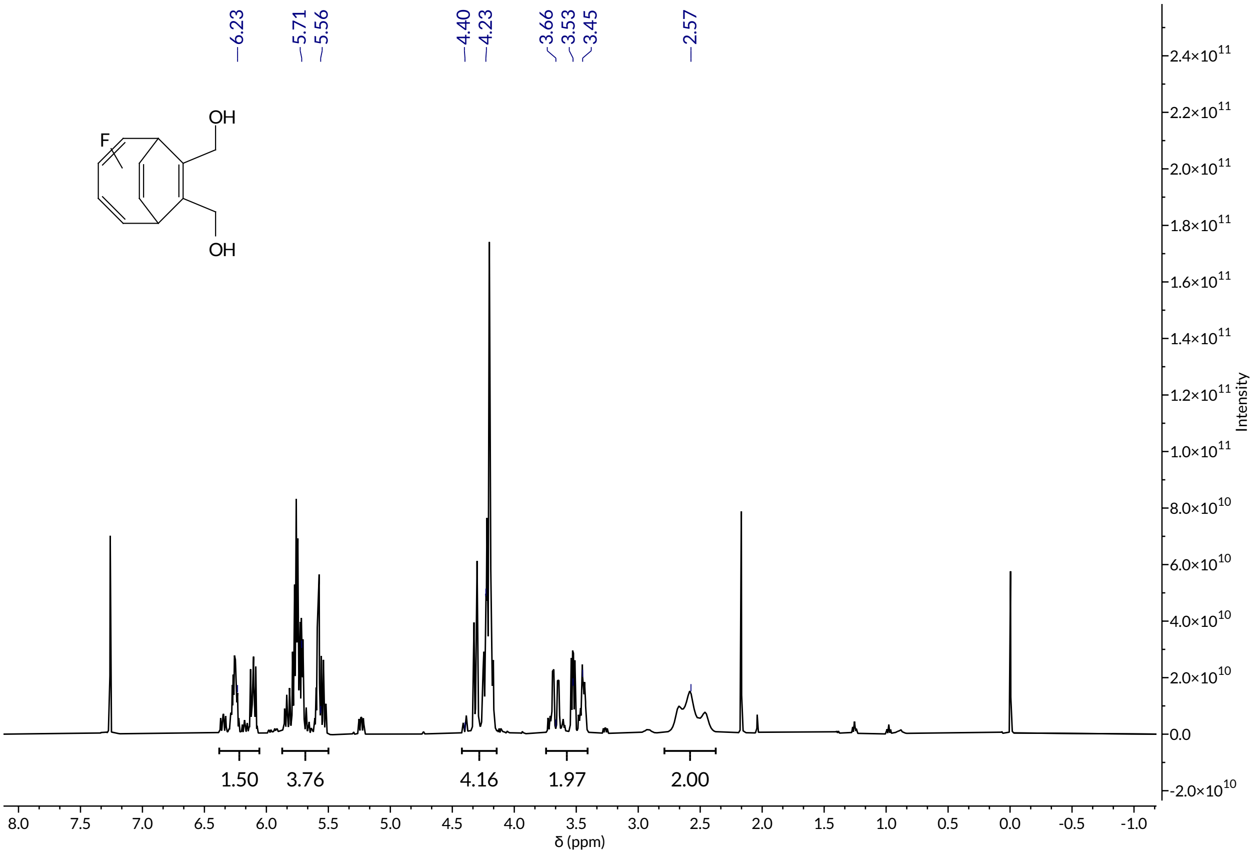

Figure S8: ^{1}H NMR spectrum of **F-CA-Diol** ($CDCl_3$, 500 MHz)

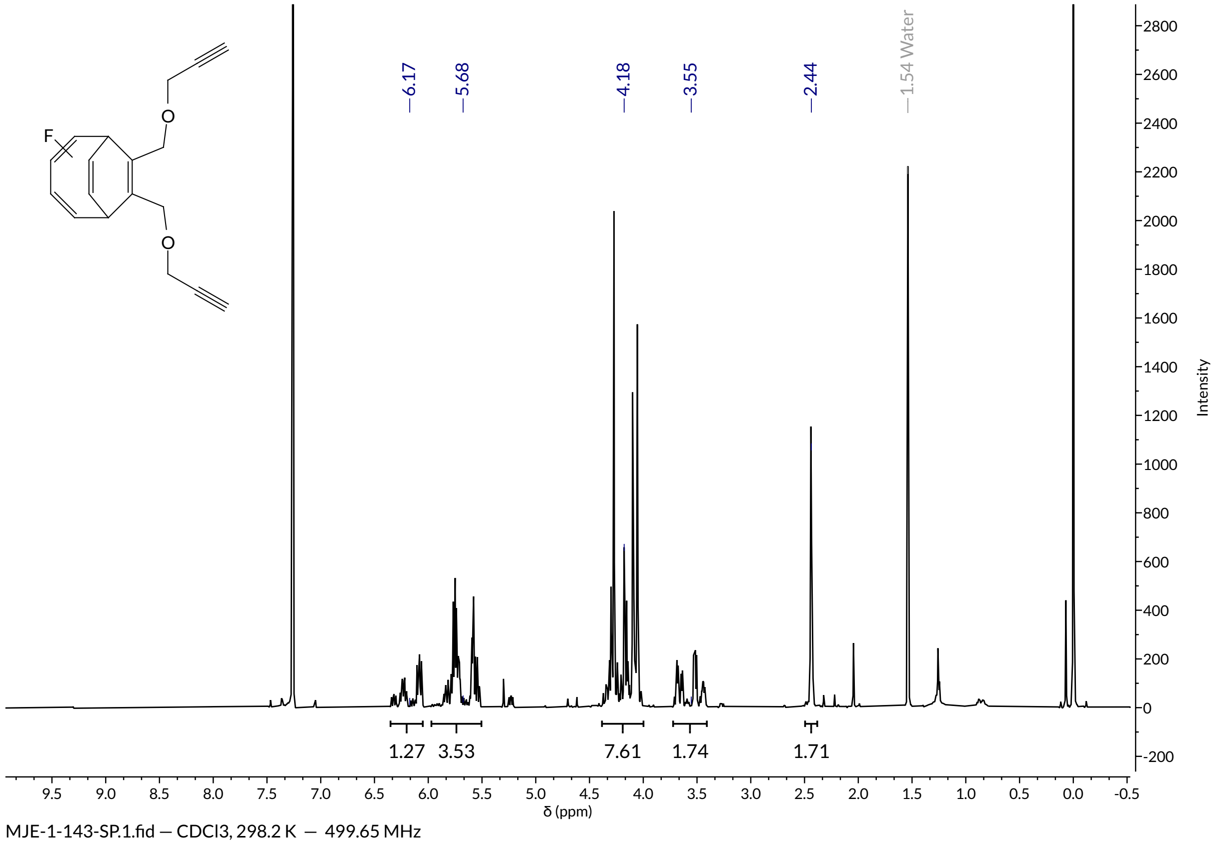

Figure S9: ^{1}H NMR spectrum of **F-CA-PE** ($CDCl_3$, 500 MHz)

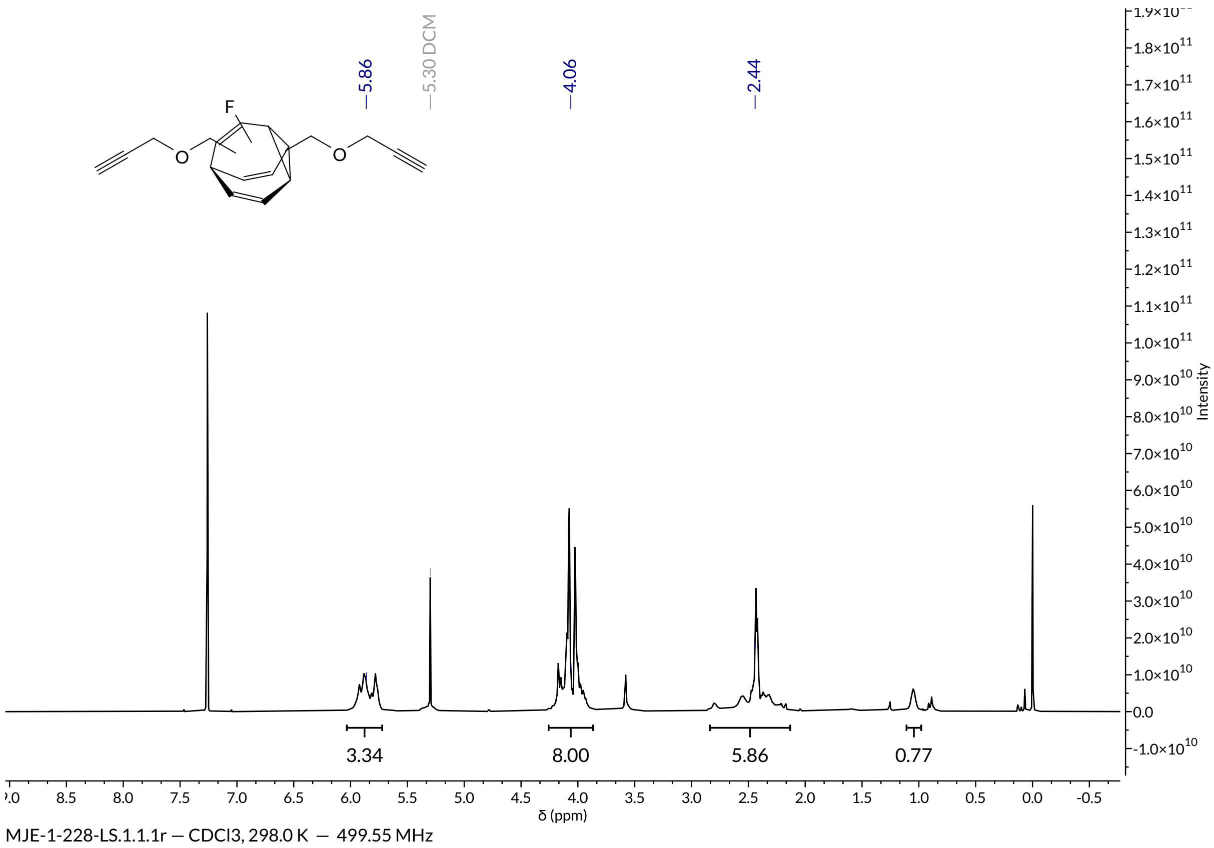

Figure S10: ^{1}H NMR spectrum of **F-Bull-PE** ($CDCl_3$, 500 MHz)

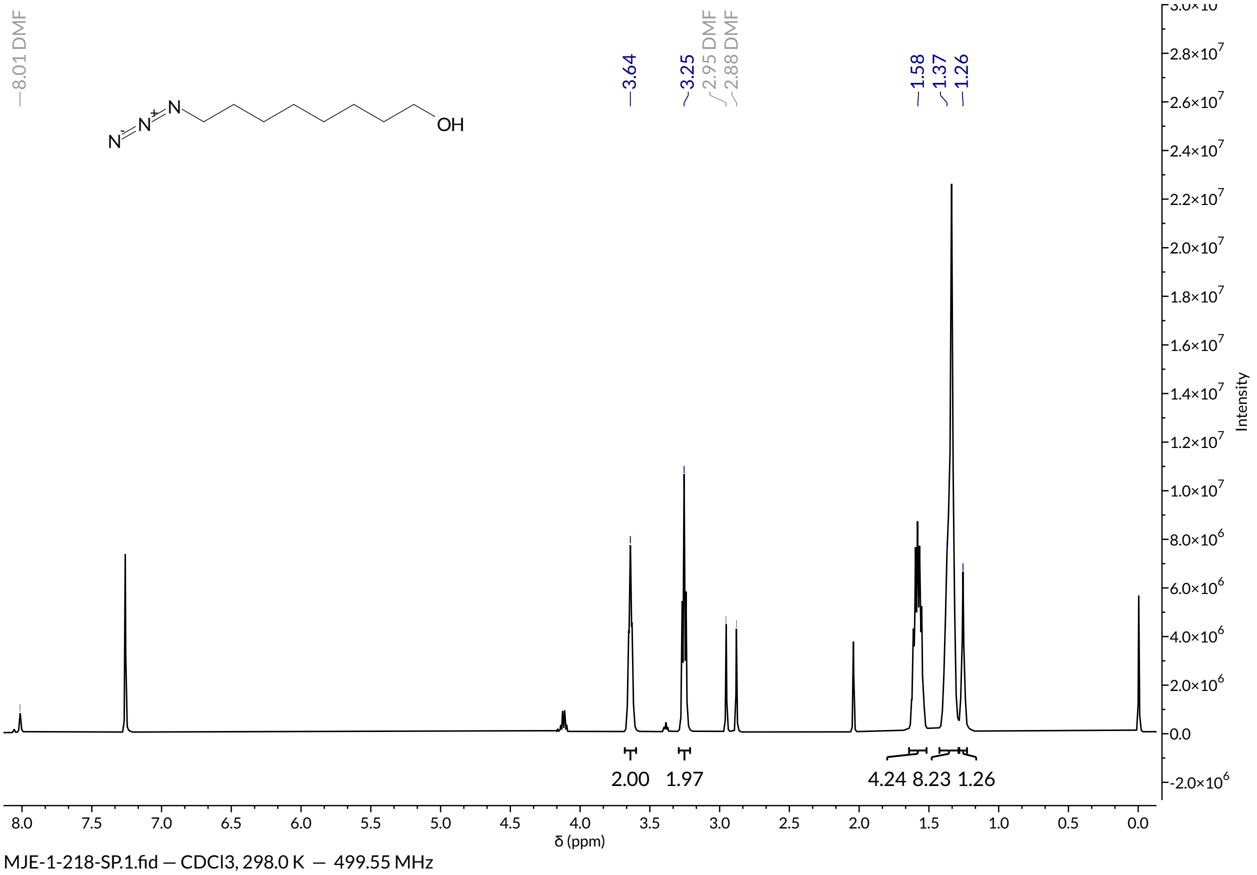

Figure S11: ^{1}H NMR spectrum of **N_3-Oct-OH** ($CDCl_3$, 500 MHz)

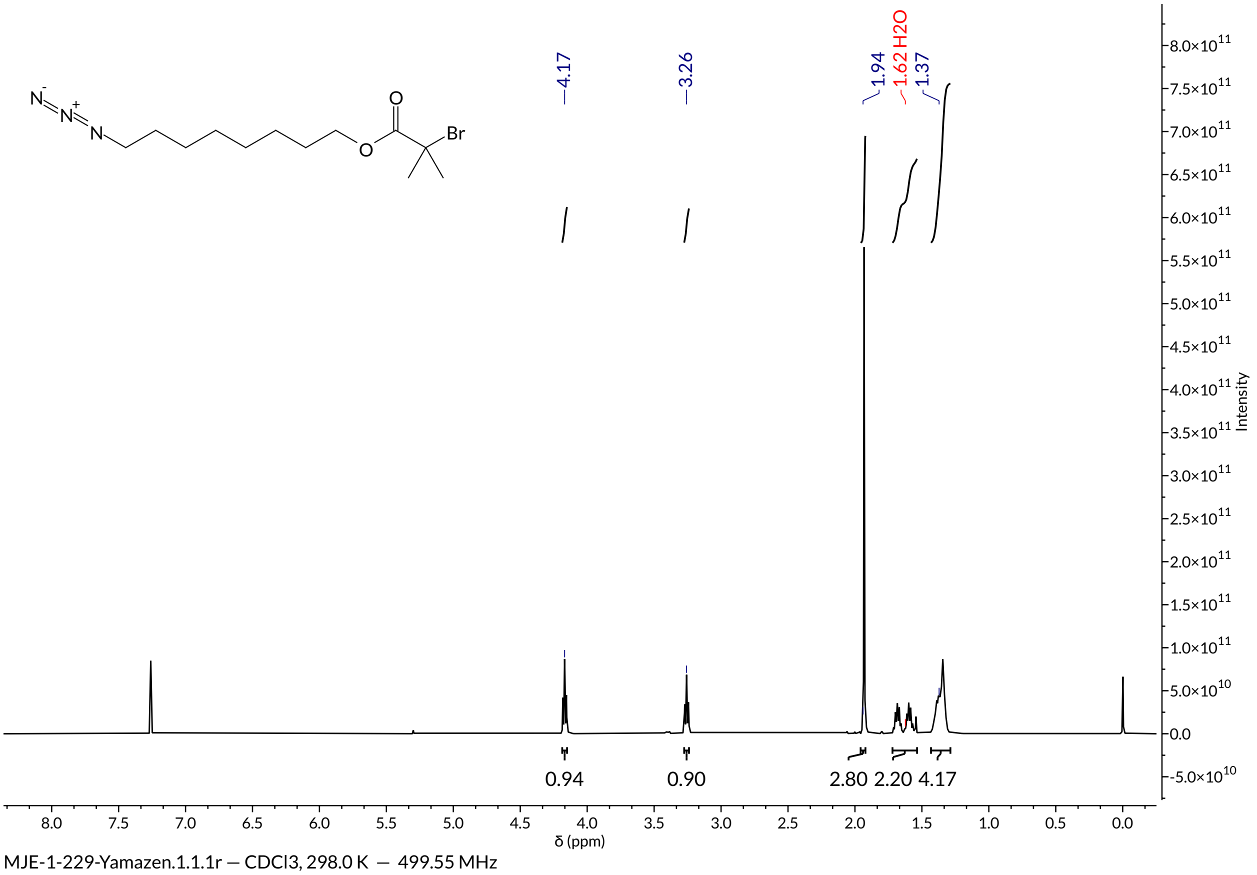

Figure S12: ^{1}H NMR spectrum of **N_3-ATRP** ($CDCl_3$, 500 MHz)

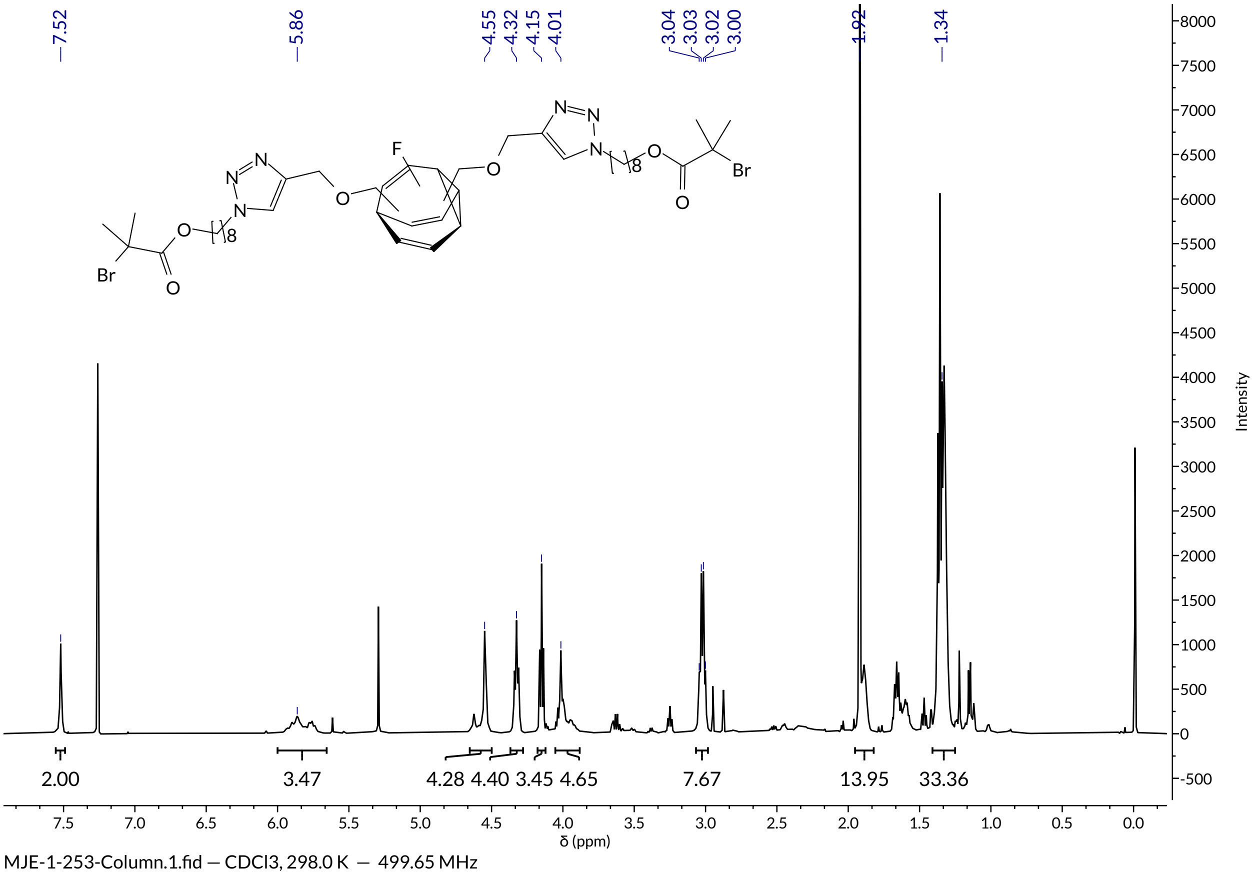

Figure S13: ^{1}H NMR spectrum of **F-Bull-ATRP** ($CDCl_3$, 500 MHz)

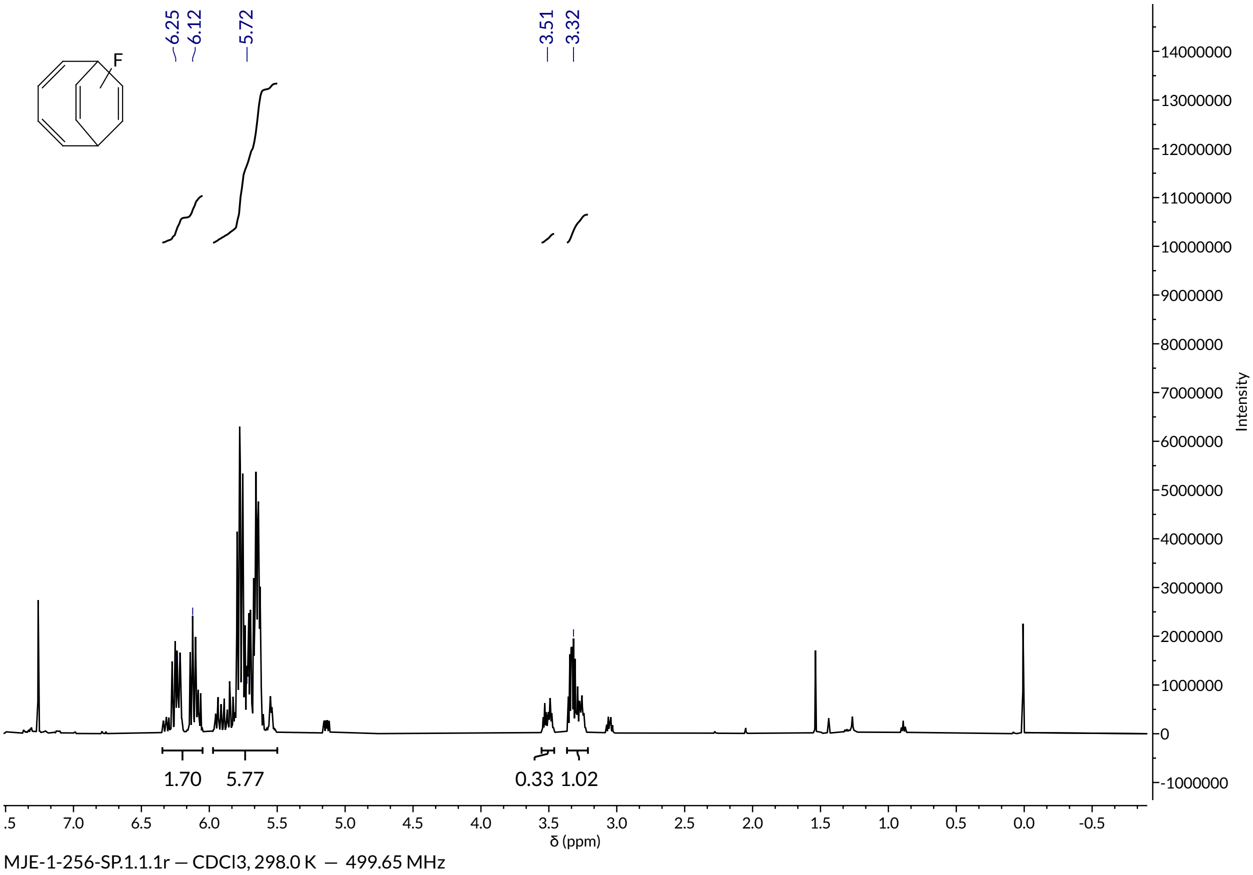

Figure S14: ^{1}H NMR spectrum of **F**-**CA** ($CDCl_3$, 500 MHz)

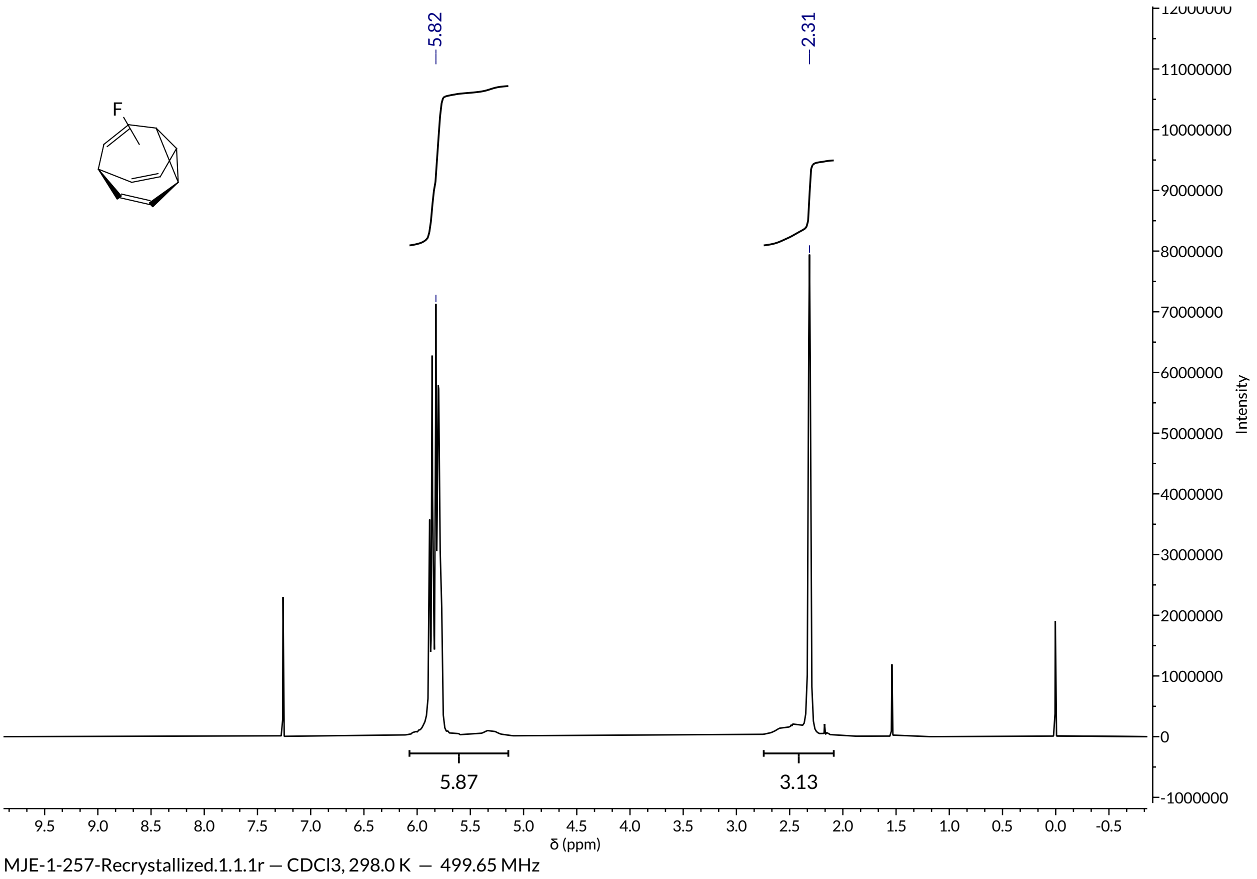

Figure S15: ^{1}H NMR spectrum of **F**-**Bull** ($CDCl_3$, 500 MHz)

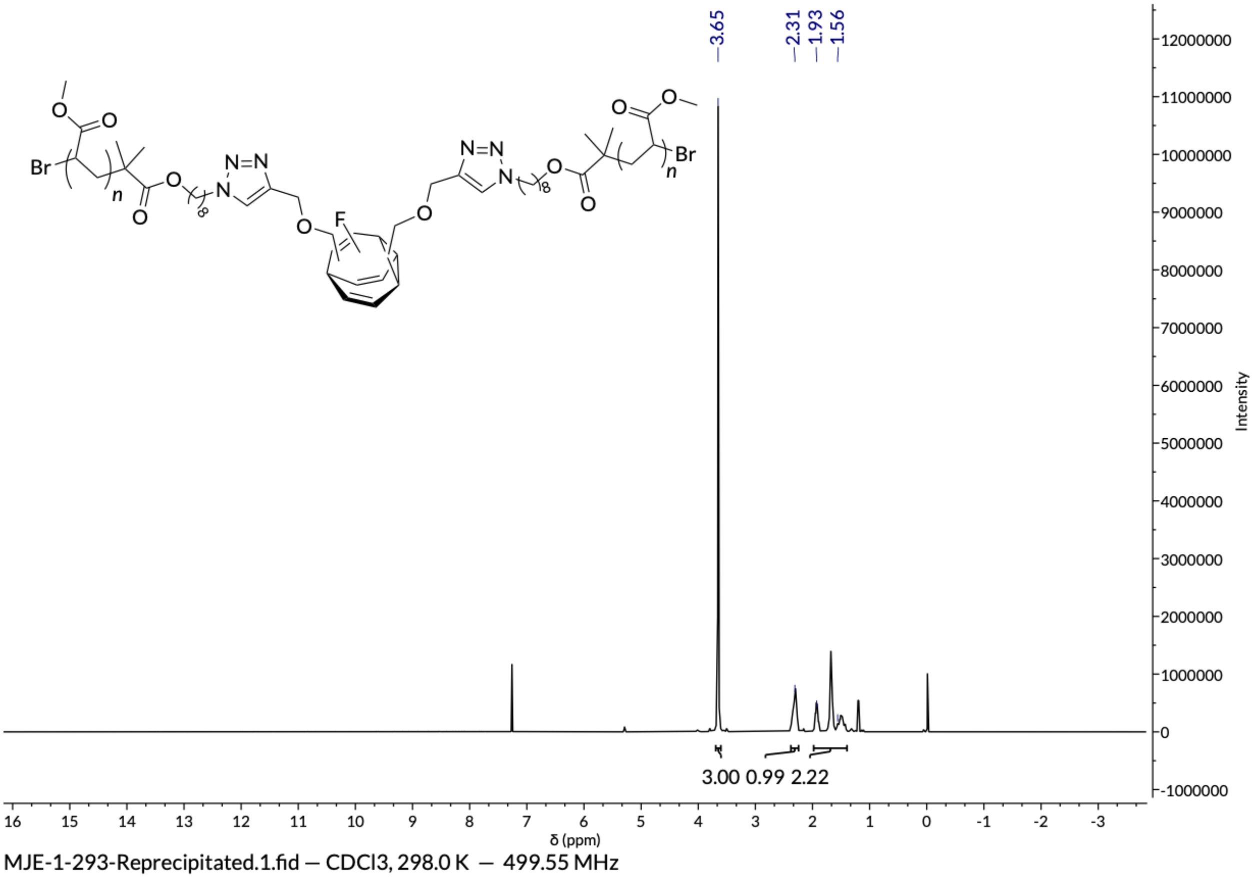

Figure S16: ^{1}H NMR spectrum of **F**-**Bull**-**PMA** ($CDCl_3$, 500 MHz)

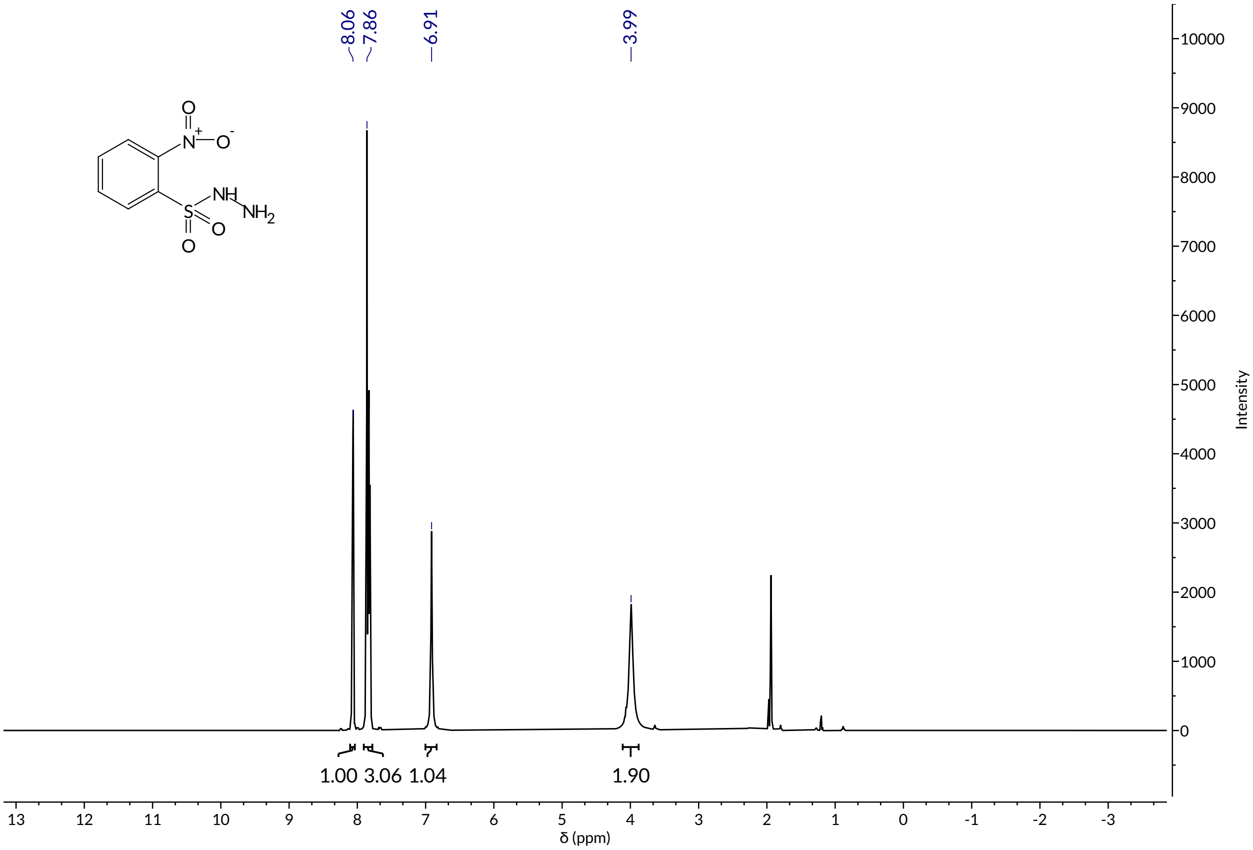

Figure S17: ^{1}H NMR spectrum of **NBSH** (CD_3CN, 500 MHz)

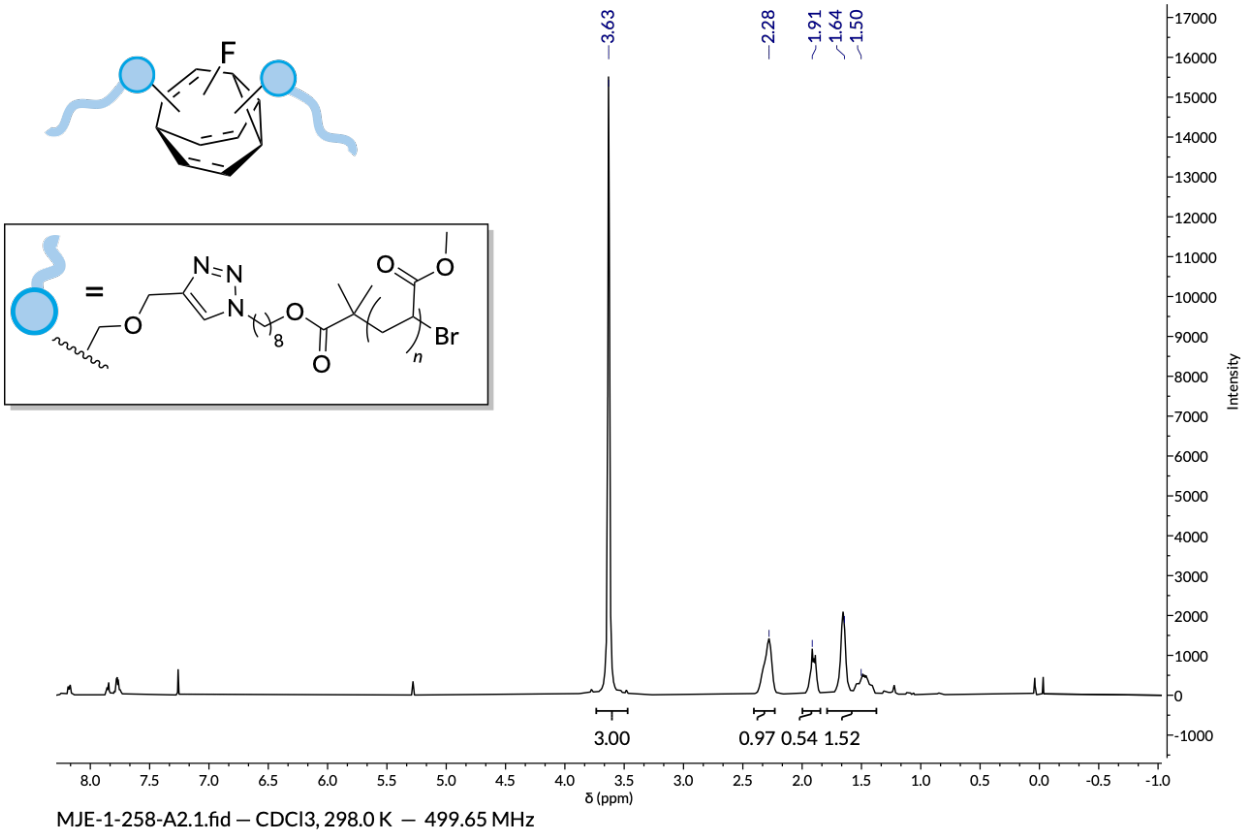

Figure S18: ^{1}H NMR spectrum of **F**-**Bull**-**PMA-Red-Sonics** ($CDCl_3$, 500 MHz)

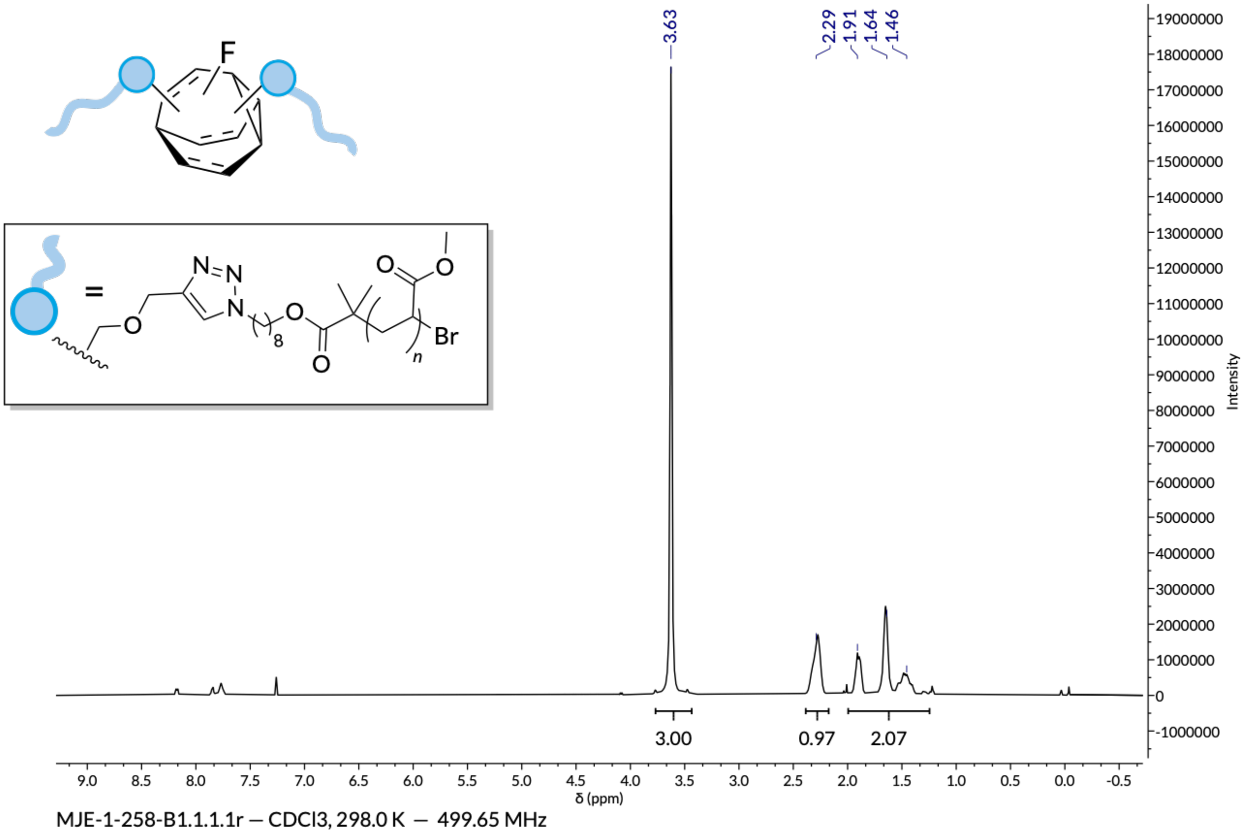

Figure S19: ^{1}H NMR spectrum of **F**-**Bull**-**PMA-Red-Stir** ($CDCl_3$, 500 MHz)

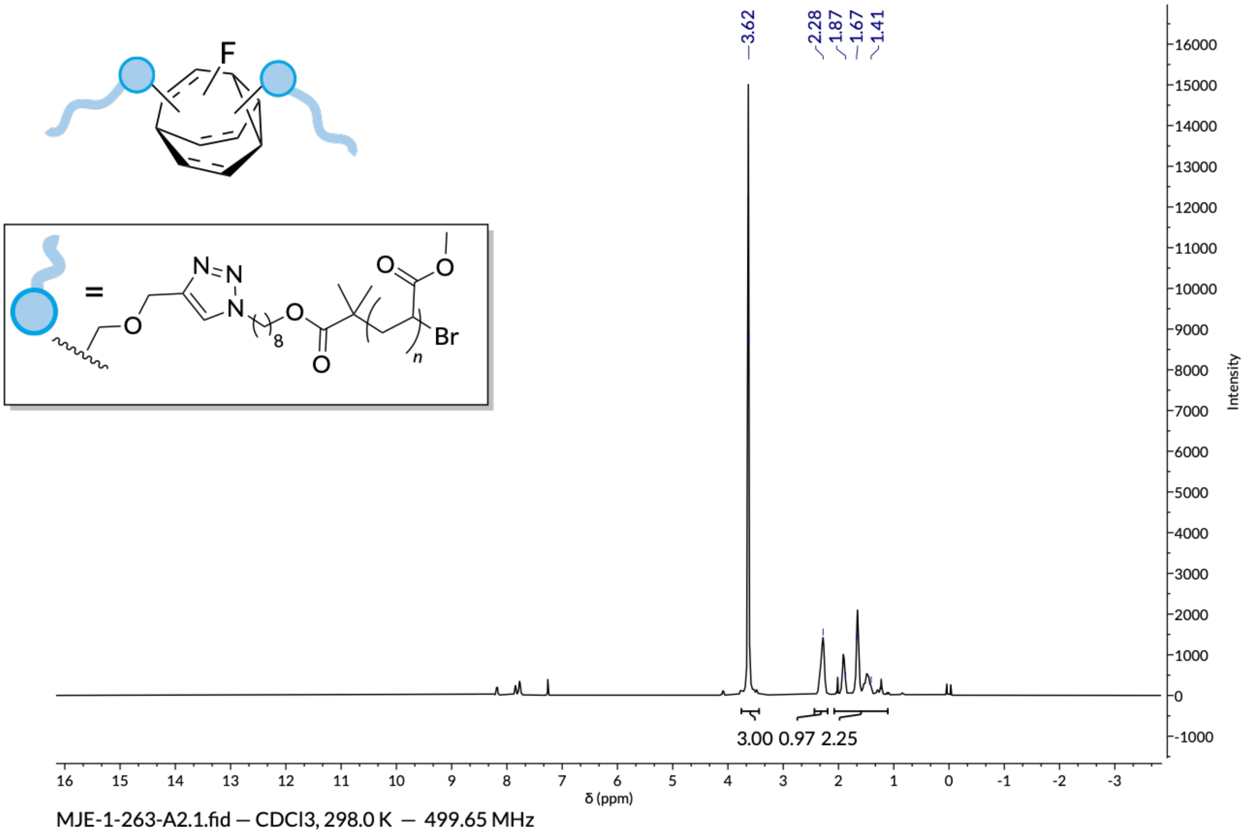

Figure S20: ^{1}H NMR spectrum of **F-Bull-PMA-Red-Sonics-Cold** ($CDCl_3$, 500 MHz)

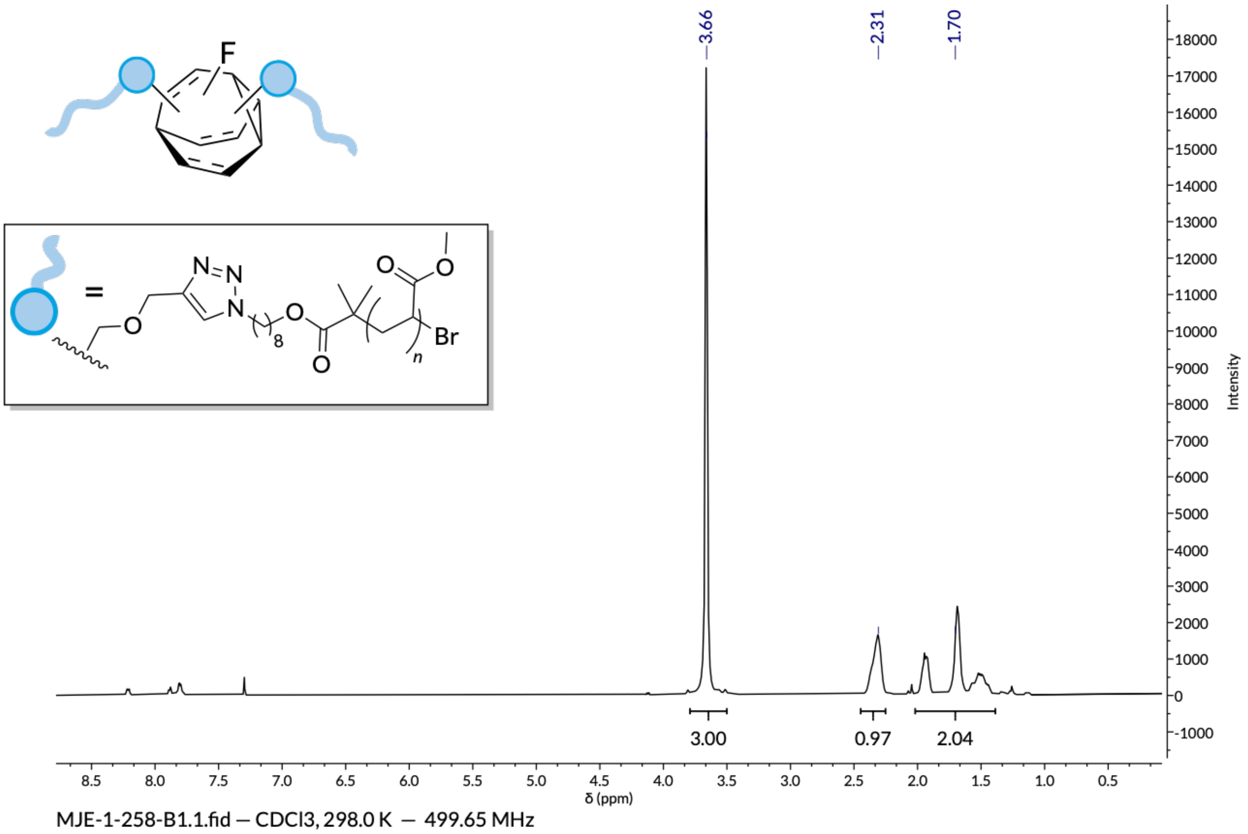

Figure S21: ^{1}H NMR spectrum of **F**-**Bull**-**PMA-Red-Stir-Cold** ($CDCl_3$, 500 MHz)

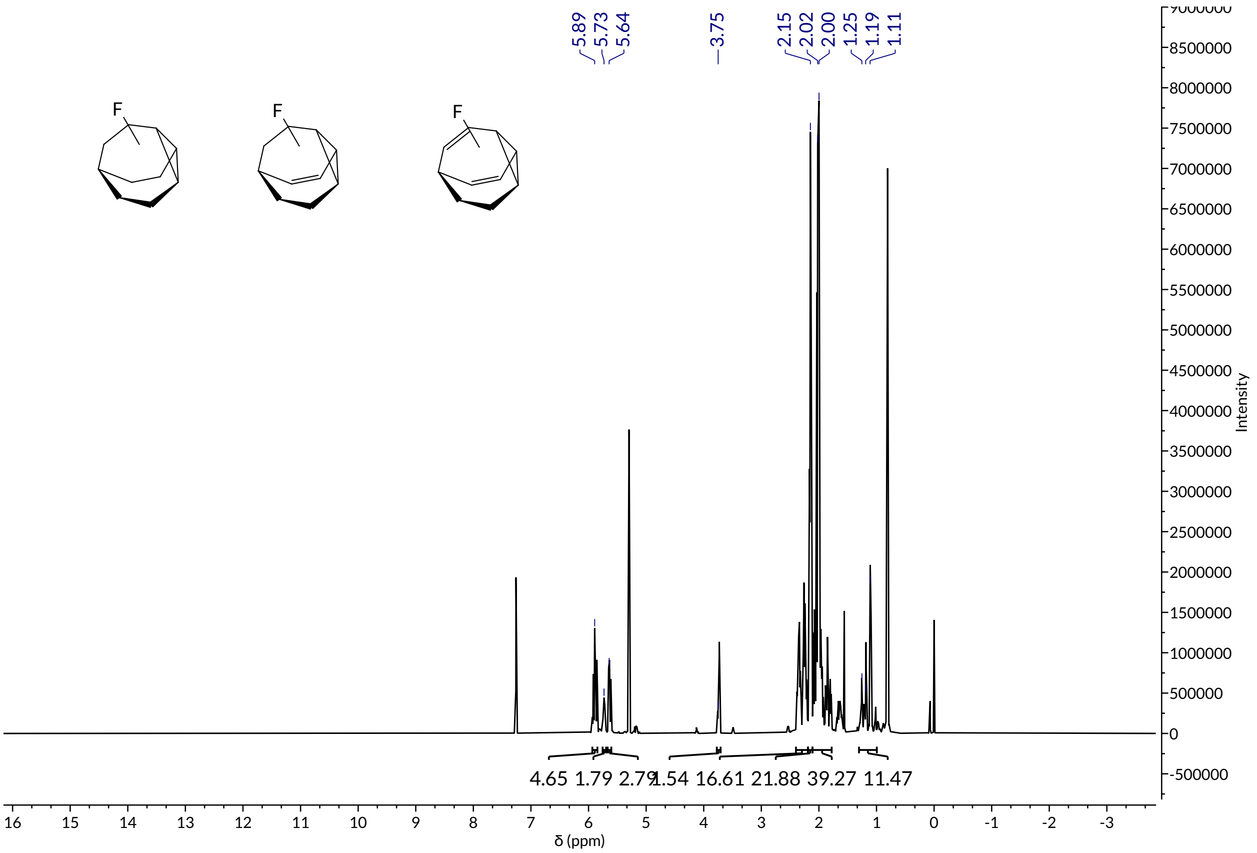

MJE-1-259-SP.1.1.1r — CDCl3, 298.0 K — 499.65 MHz

Figure S22: ^{1}H NMR spectrum of **F**-**Bull**-**Red**^{n} ($CDCl_3$, 500 MHz)

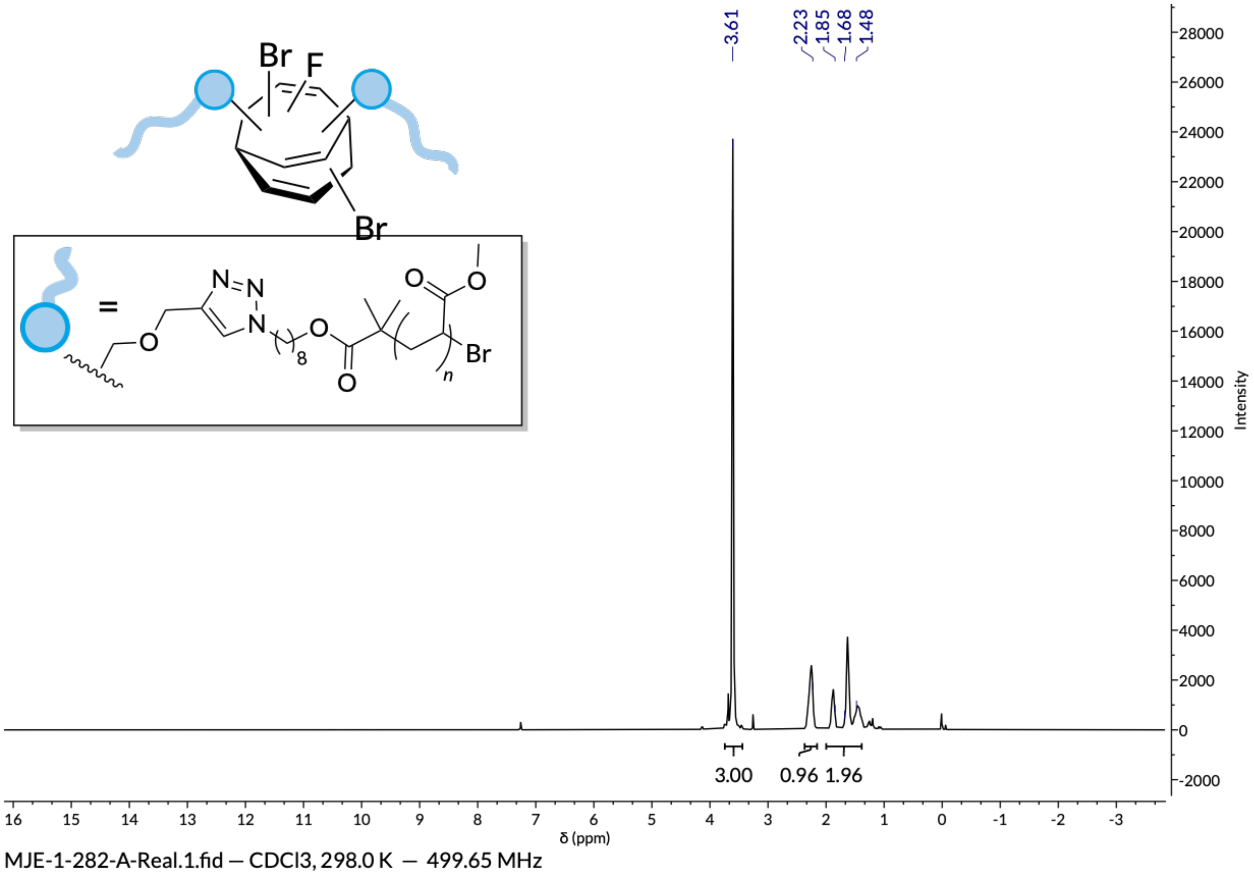

Figure S23: ^{1}H NMR spectrum of **F**-**Bull**-**PMA-Br$_2$-Cold** ($CDCl_3$, 500 MHz)

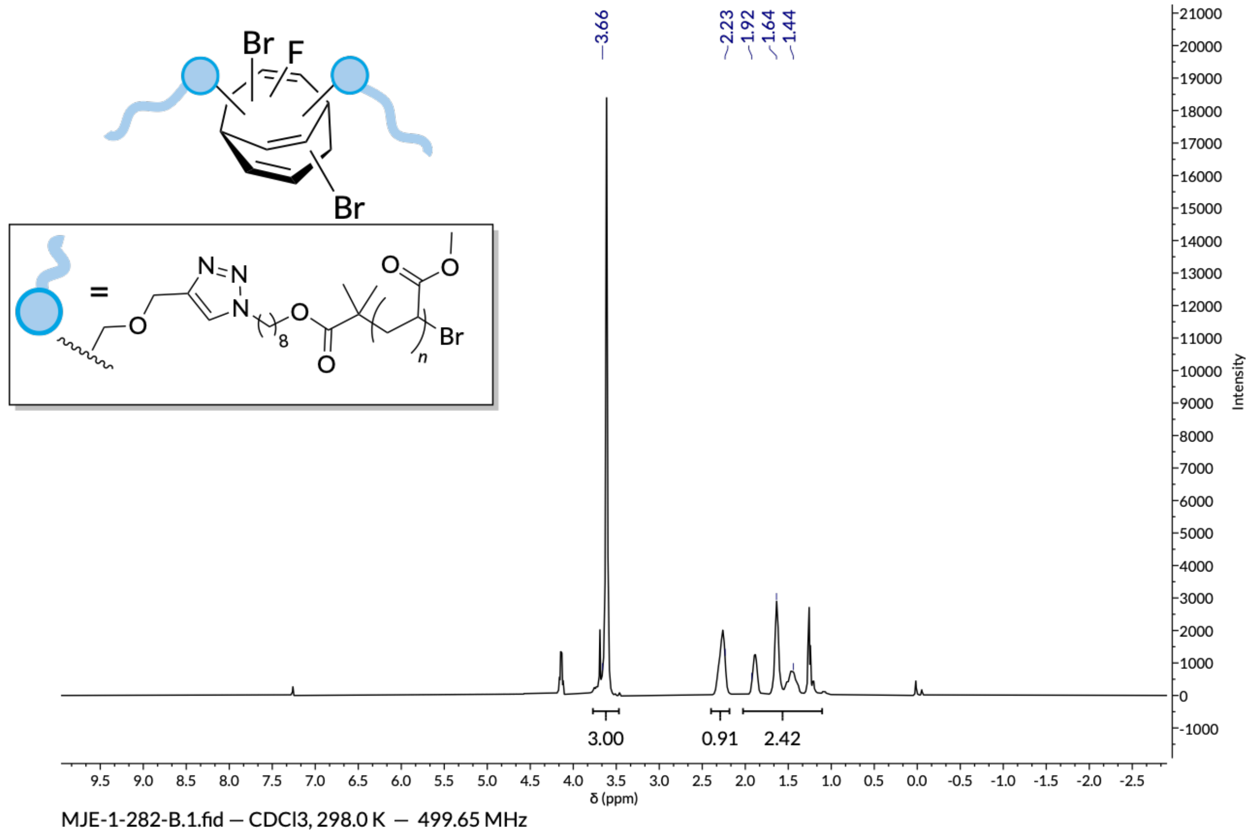

Figure S24: ^{1}H NMR spectrum of **F**-**Bull**-**PMA-Br$_2$-Warmed** ($CDCl_3$, 500 MHz)

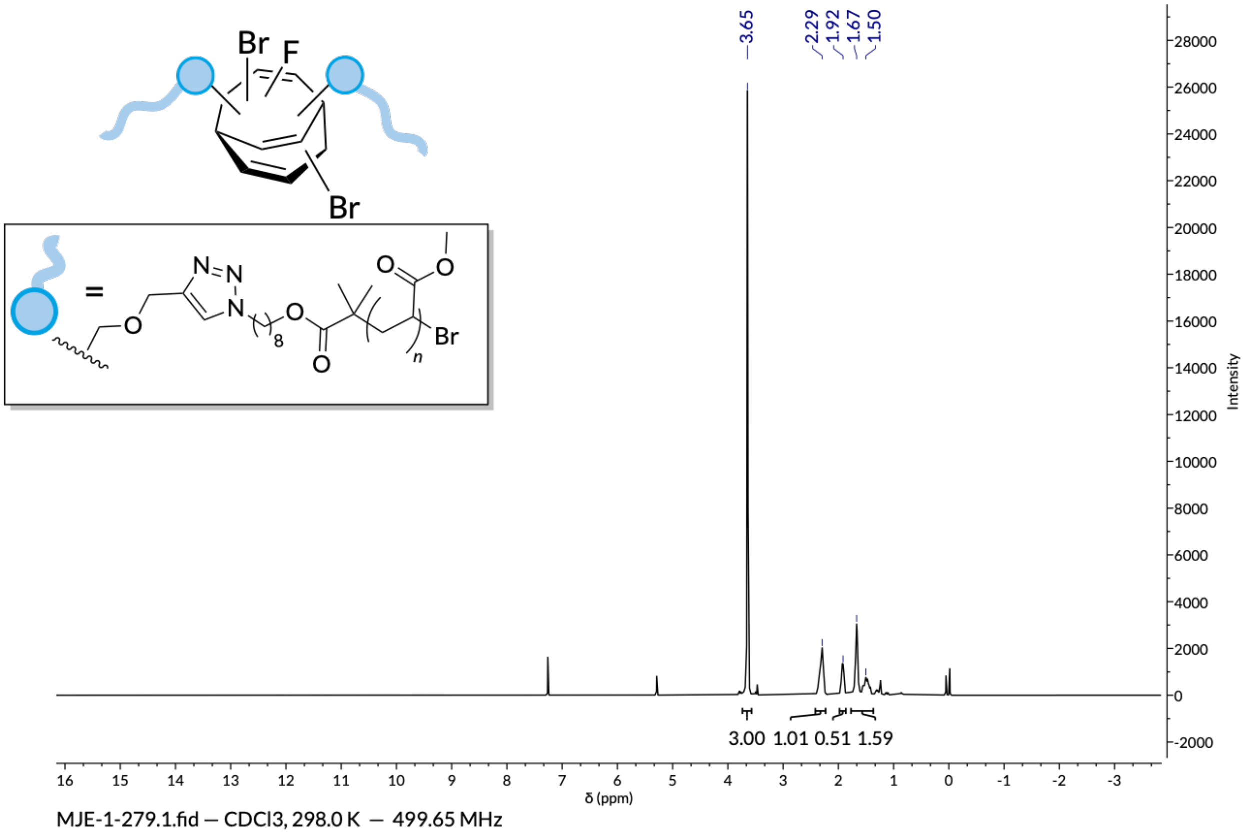

Figure S25: ^{1}H NMR spectrum of **F**-**Bull**-**PMA-Br$_2$-FF** ($CDCl_3$, 500 MHz)

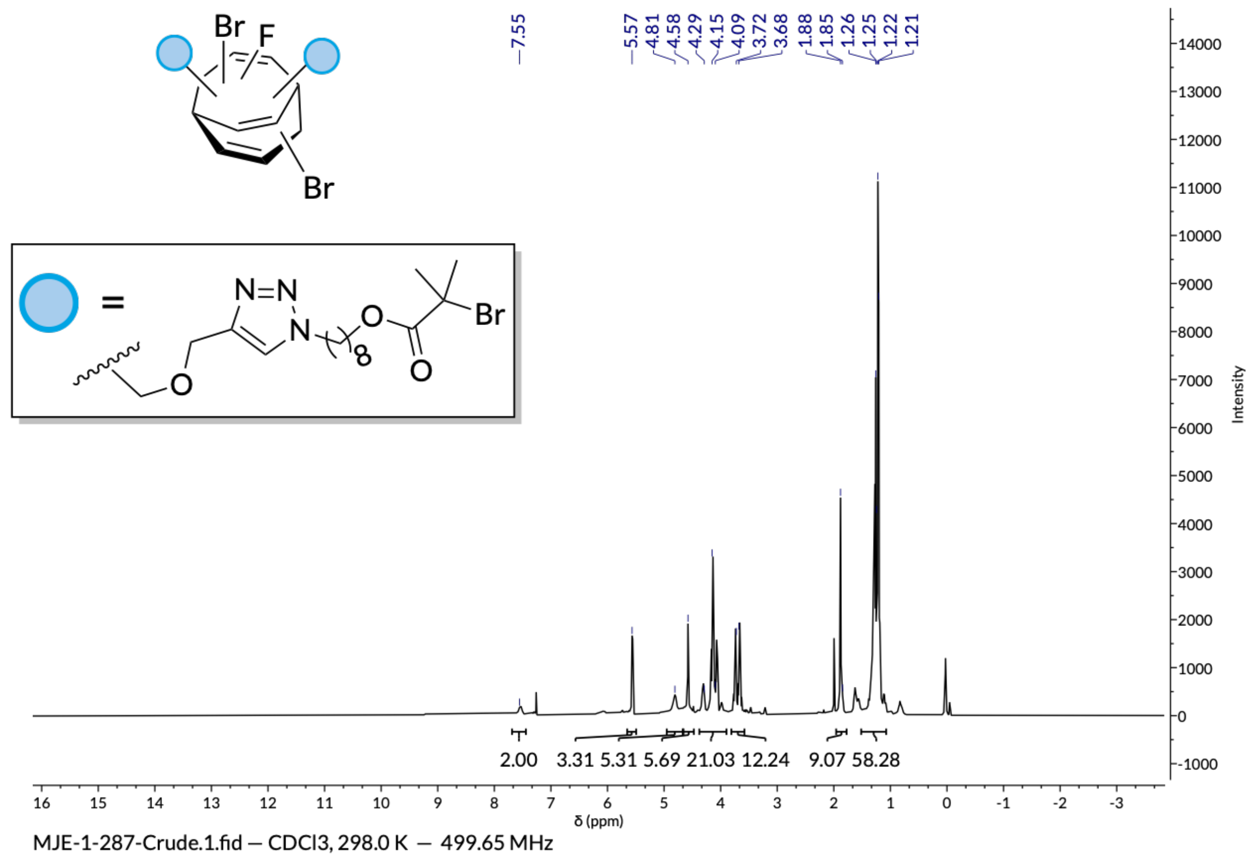

Figure S26: ^{1}H NMR spectrum of **F**-**Bull**-**ATRP-Br$_2$-FF** ($CDCl_3$, 500 MHz)

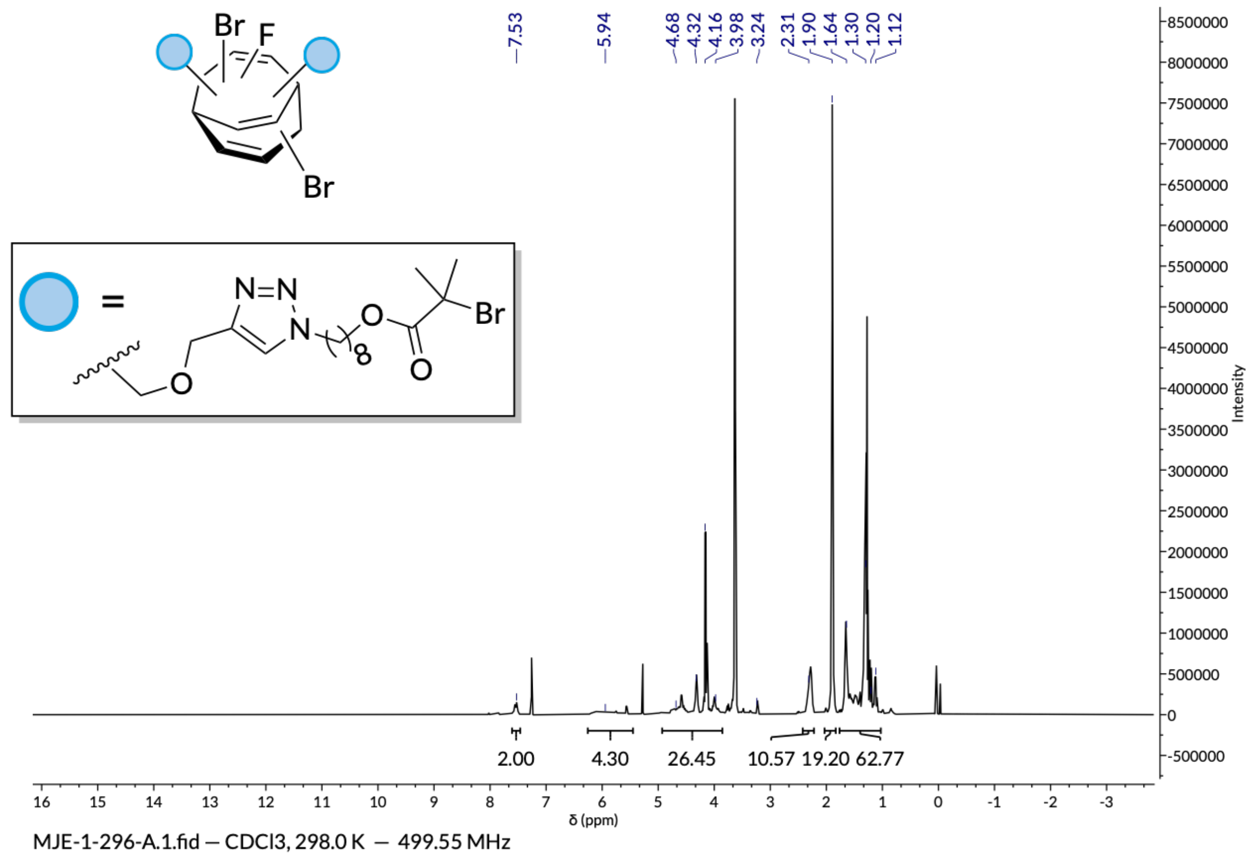

Figure S27: ^{1}H NMR spectrum of **F**-**Bull**-**ATRP-Br$_2$-Cold** ($CDCl_3$, 500 MHz)

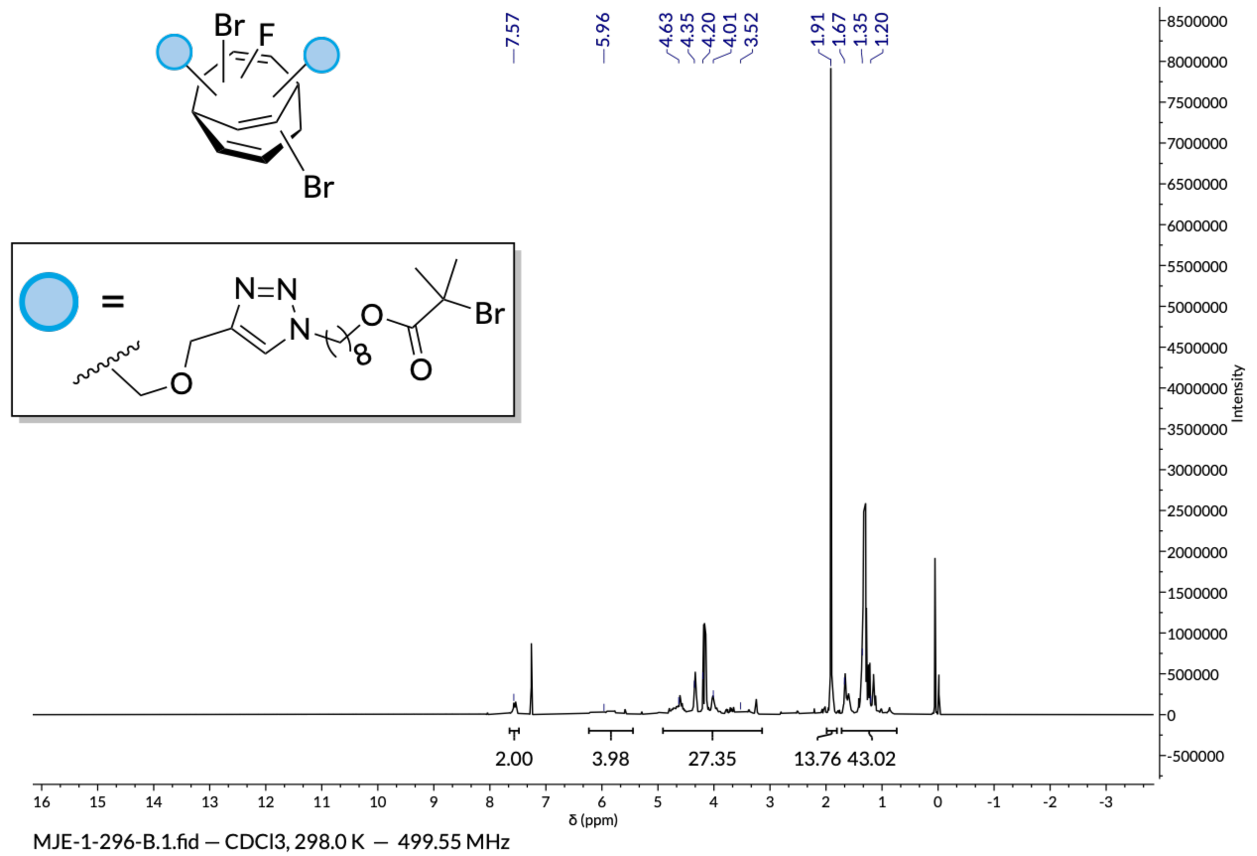

Figure S28: ^{1}H NMR spectrum of **F**-**Bull**-**ATRP-Br₂-Warmed** ($CDCl_3$, 500 MHz)

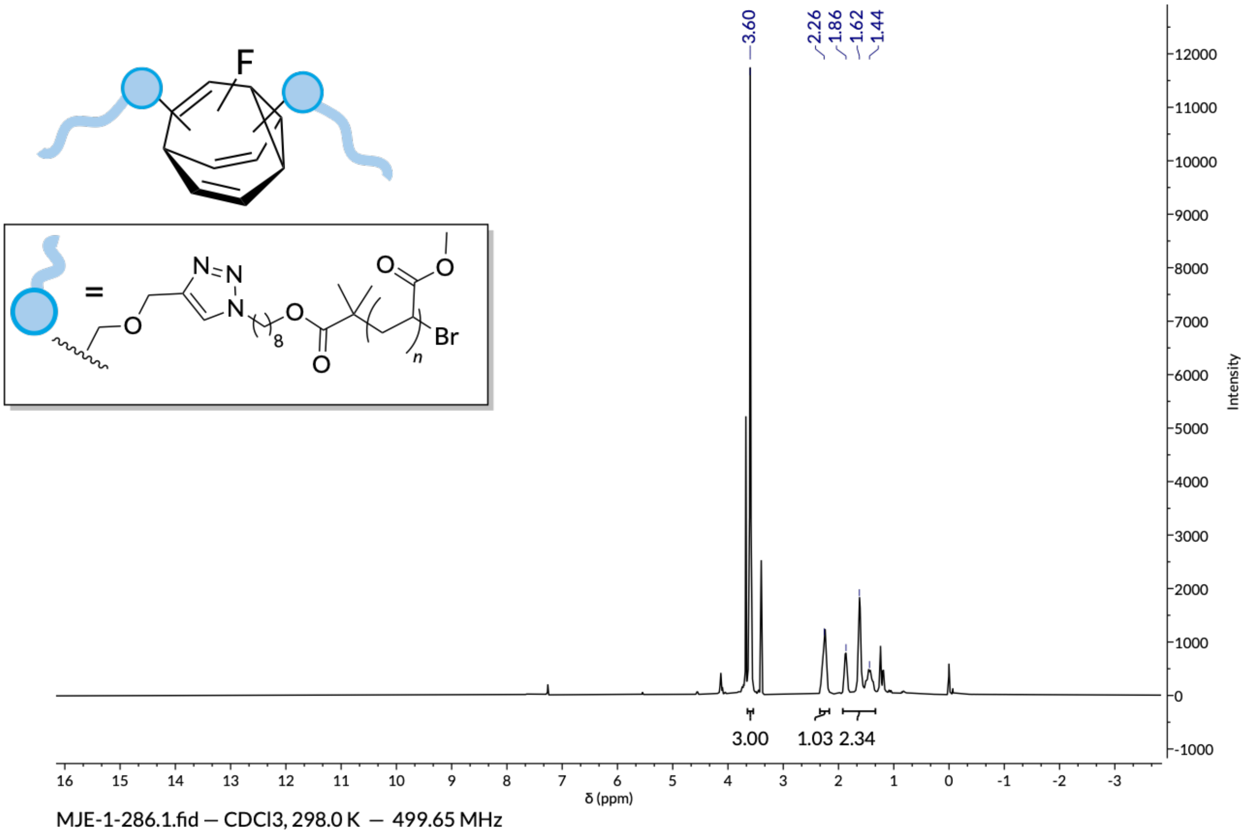

Figure S29: ^{1}H NMR spectrum of **F**-**Bull**-**PMA-Sonics-Control** ($CDCl_3$, 500 MHz)

3.2: ^{19}F NMR SPECTRA

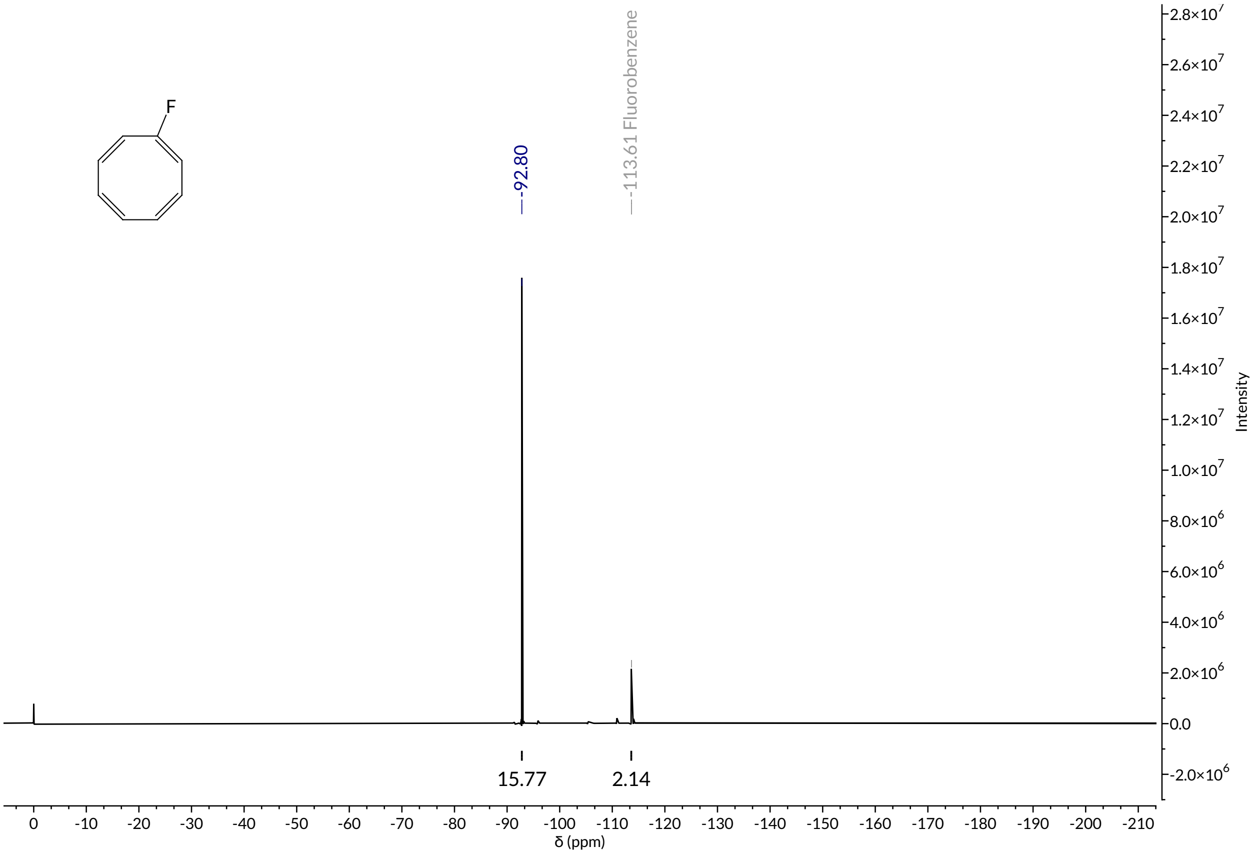

Figure S30: ^{19}F NMR spectrum of **F-COT** with **fluorobenzene internal standard** ($CDCl_3$, 470 MHz)

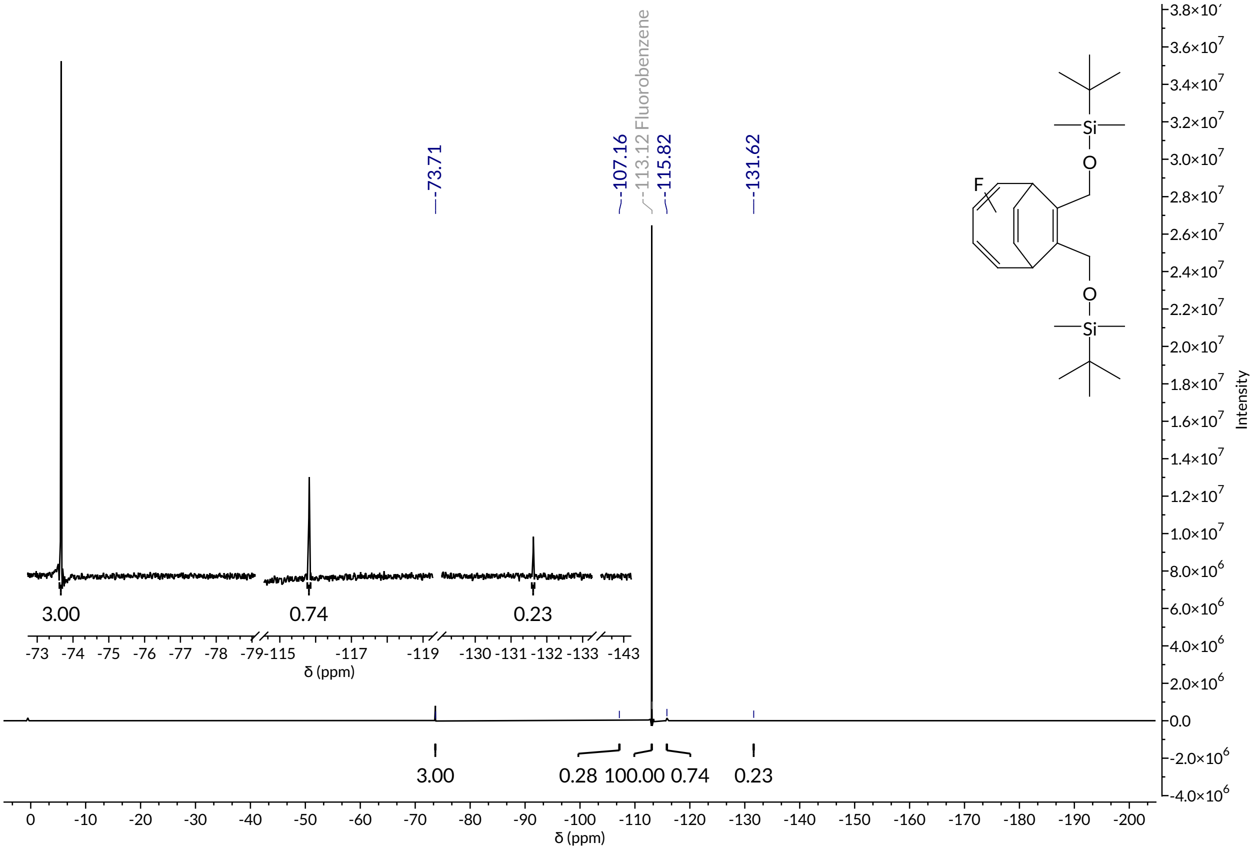

JDK-2-23_unspiked.2.1.1r — CDCl3, 298.0 K — 470.09 MHz

Figure S31: ^{19}F NMR spectrum of **F-CA-OTBDMS** with **fluorobenzene internal standard** ($CDCl_3$, 470 MHz).

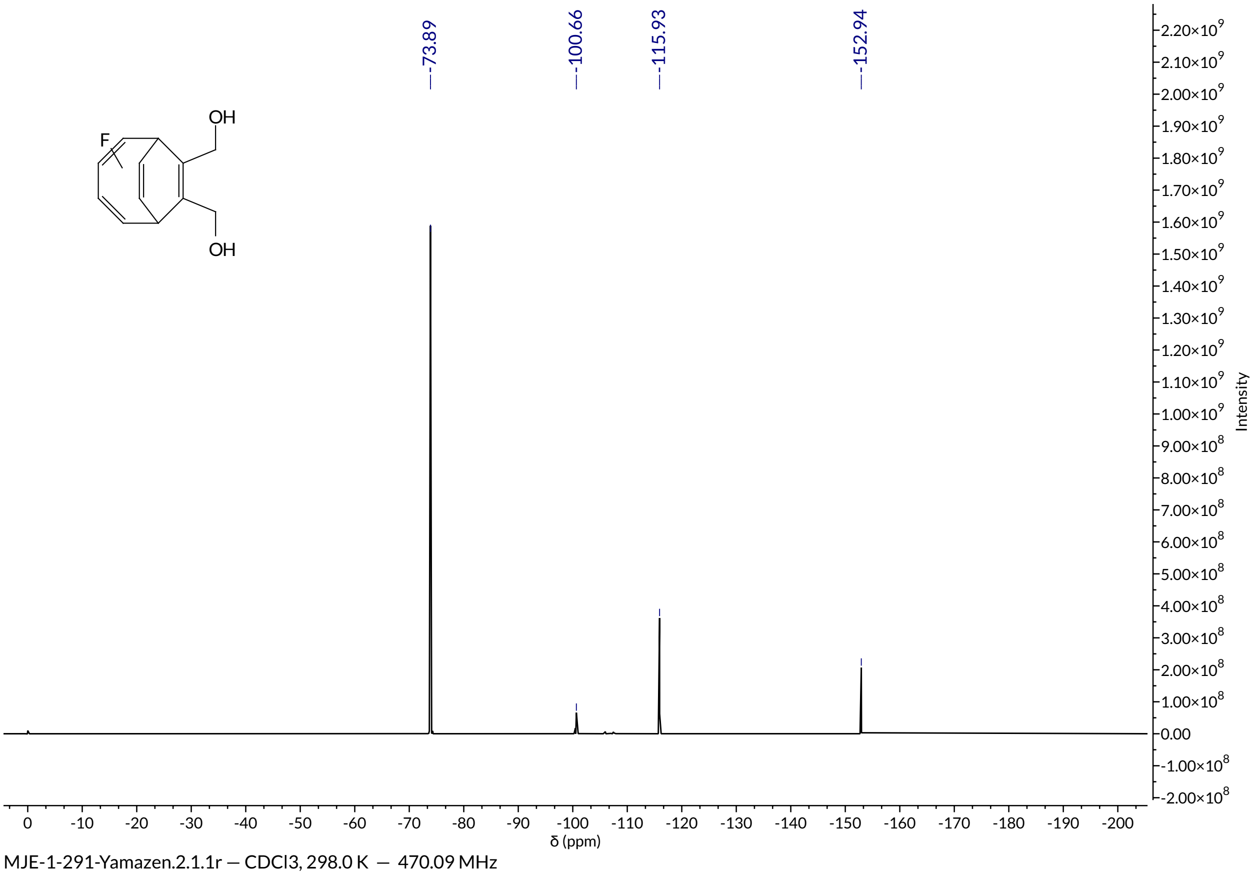

Figure S32: ^{19}F NMR spectrum of **F-CA-Diol** ($CDCl_3$, 470 MHz)

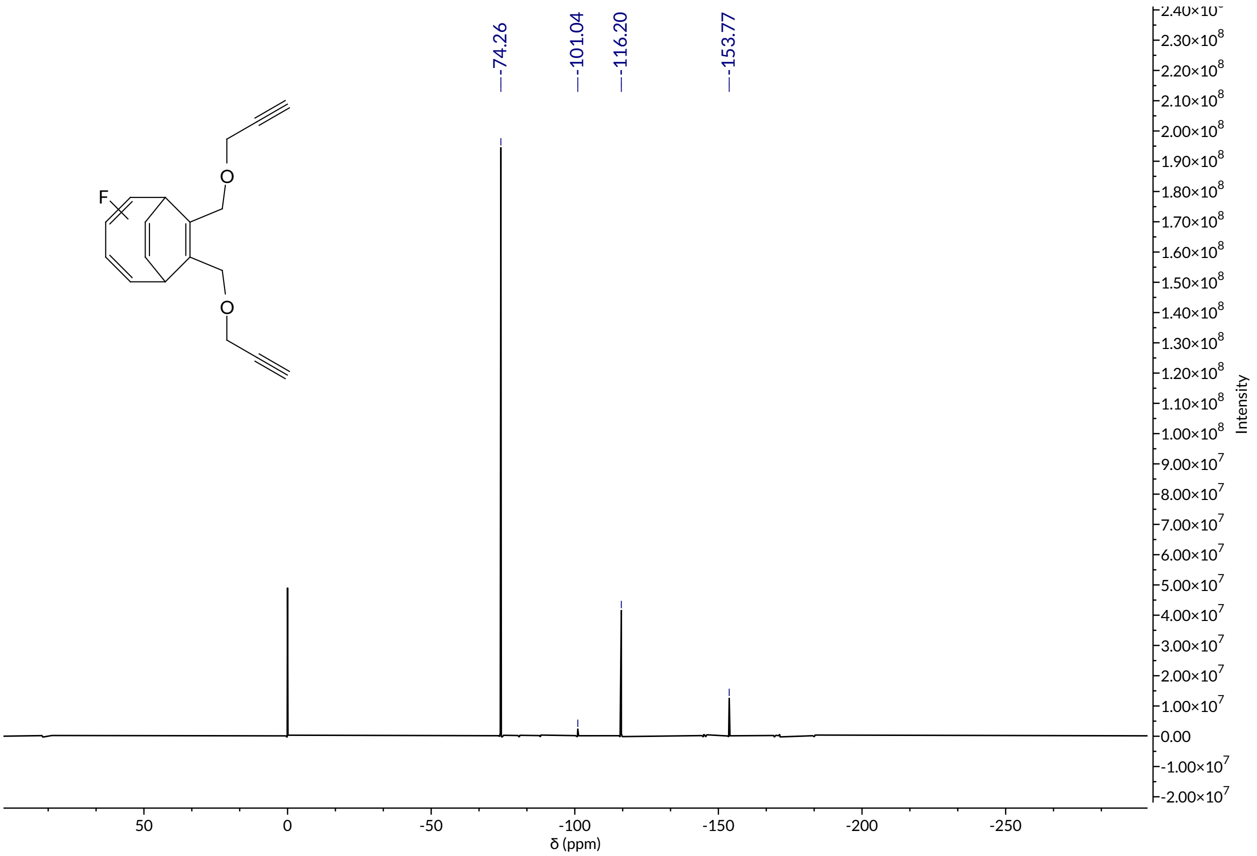

MJE-1-172-SP.2.1.1r — CDCl3, 298.0 K — 470.09 MHz

Figure S33: ^{19}F NMR spectrum of **F**-**CA-PE** ($CDCl_3$, 470 MHz)

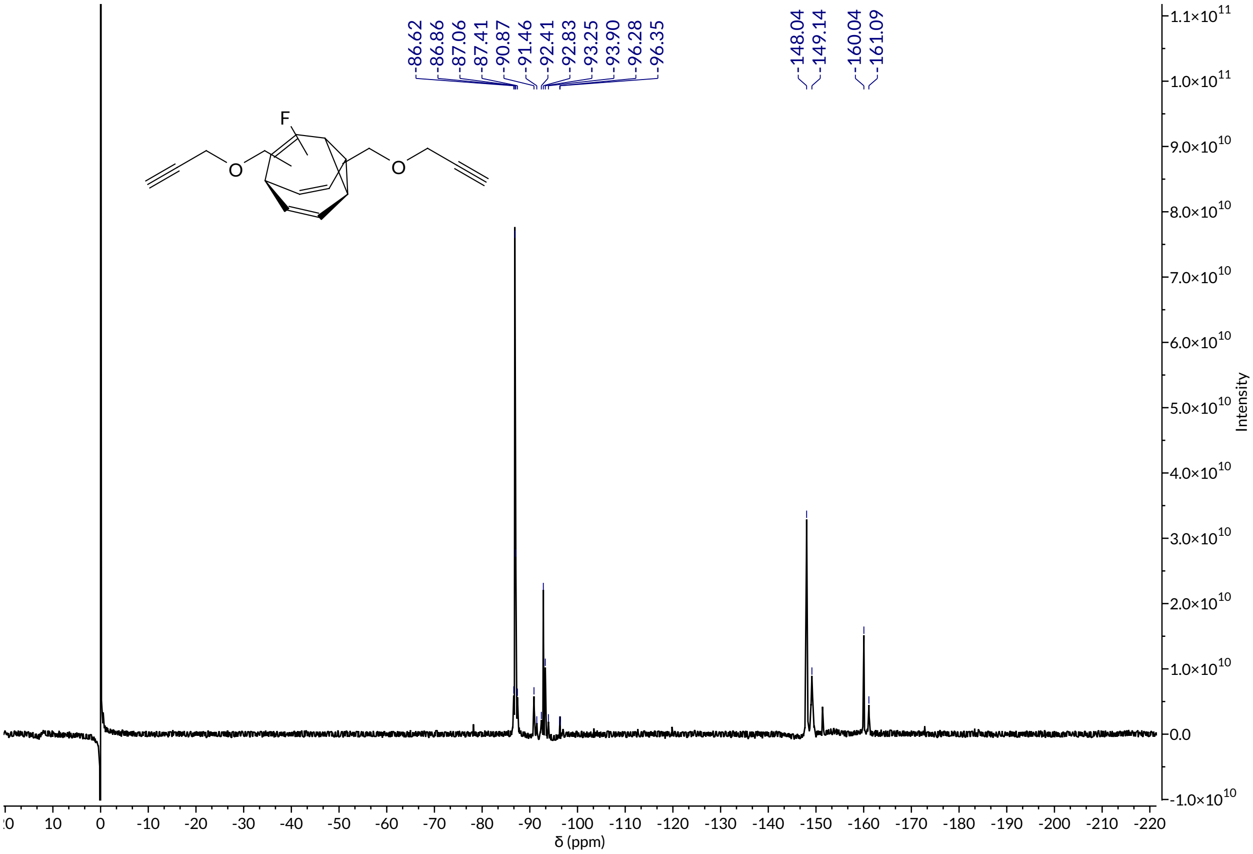

Figure S34: ^{19}F NMR spectrum of **F-Bull-PE** ($CDCl_3$, 470 MHz)

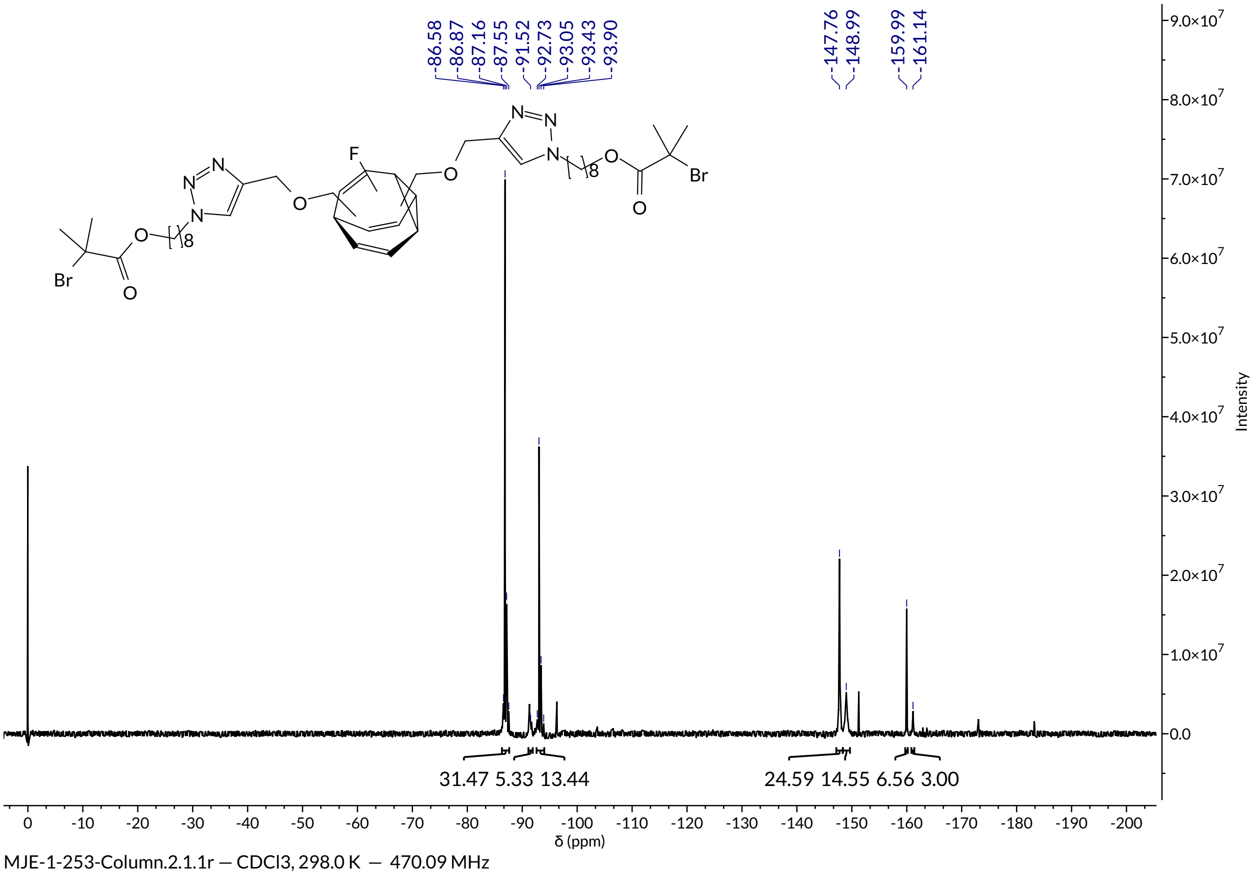

Figure S35: ^{19}F NMR spectrum of **F-Bull-ATRP** ($CDCl_3$, 470 MHz)

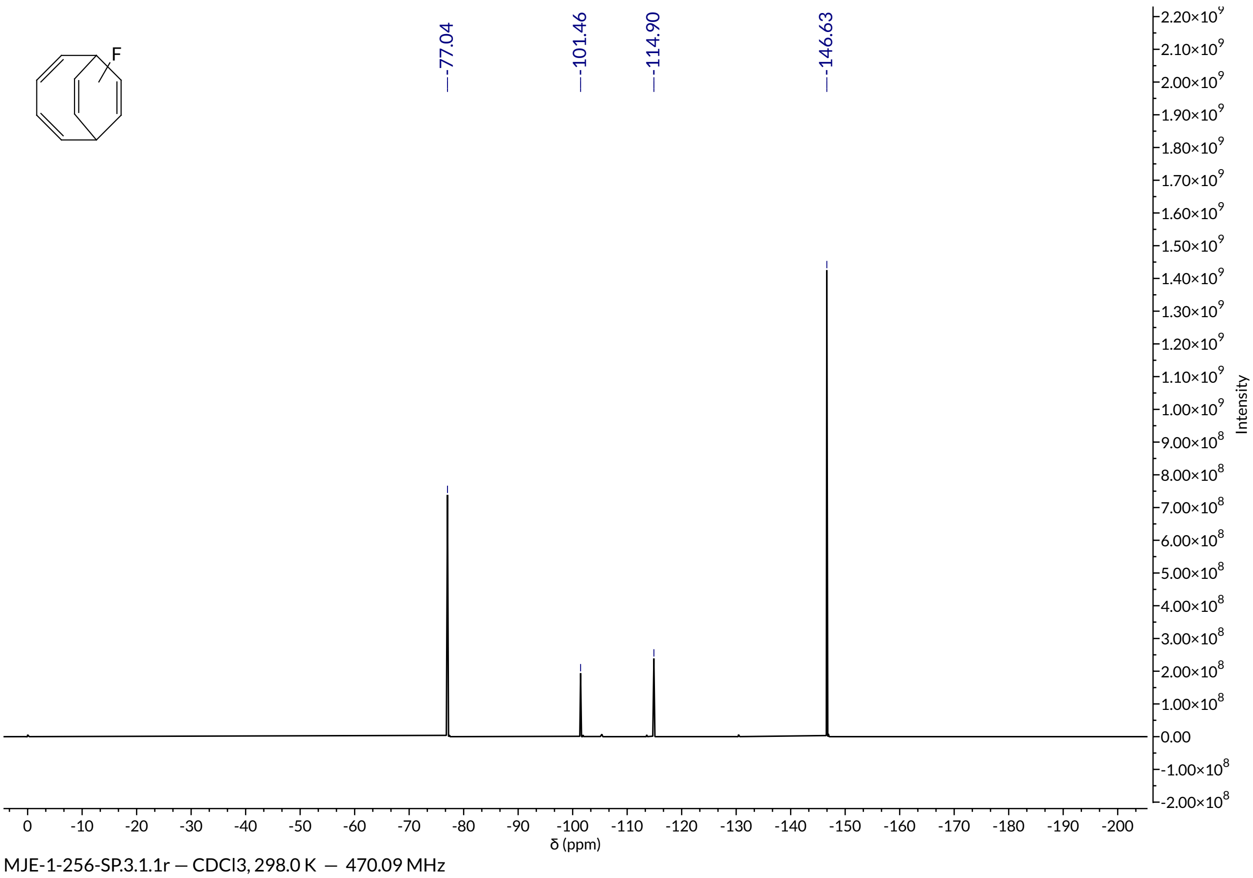

Figure S36: ^{19}F NMR spectrum of **F**-**CA** ($CDCl_3$, 470 MHz)

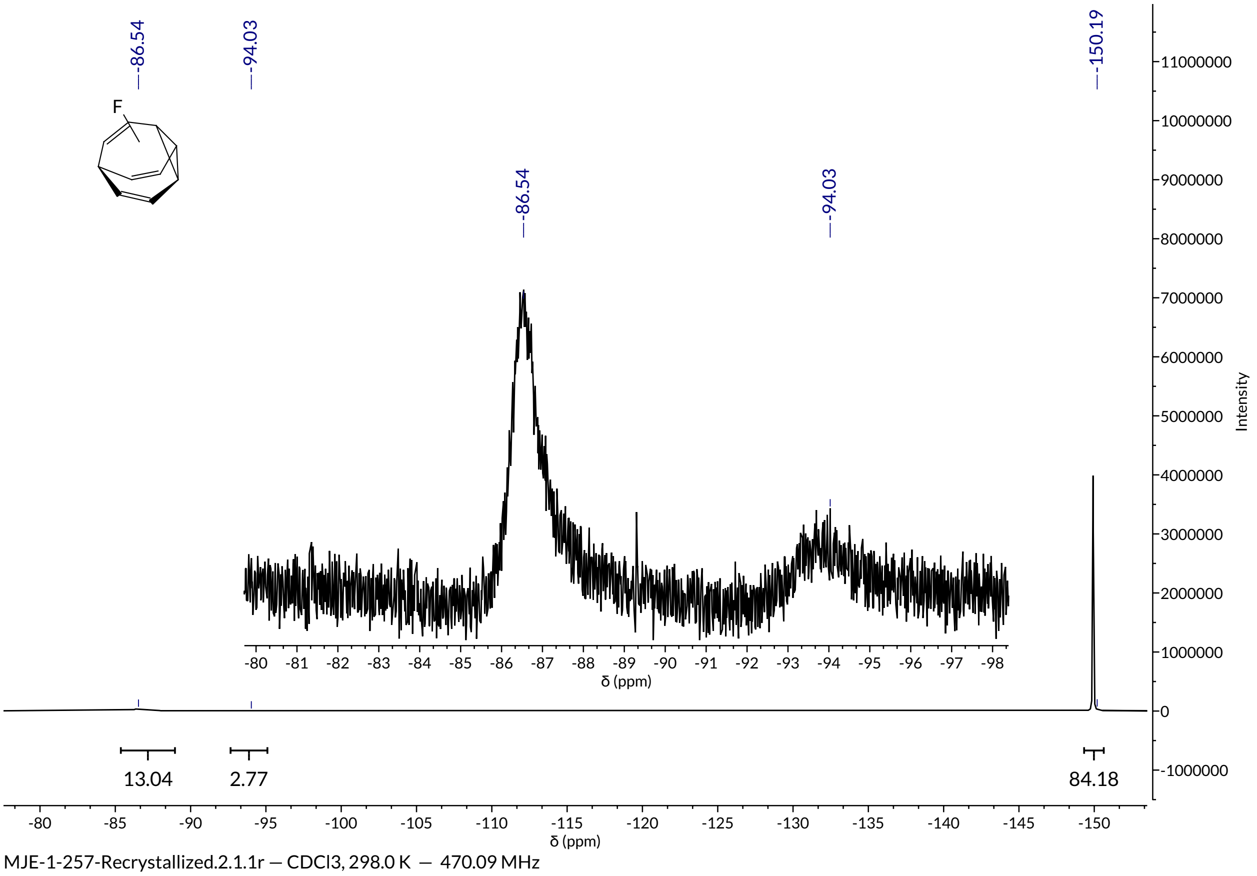

Figure S37: ^{19}F NMR spectrum of **F-Bull**. Inset shows a zoomed in region of the –80 - –100 ppm range ($CDCl_3$, 470 MHz).

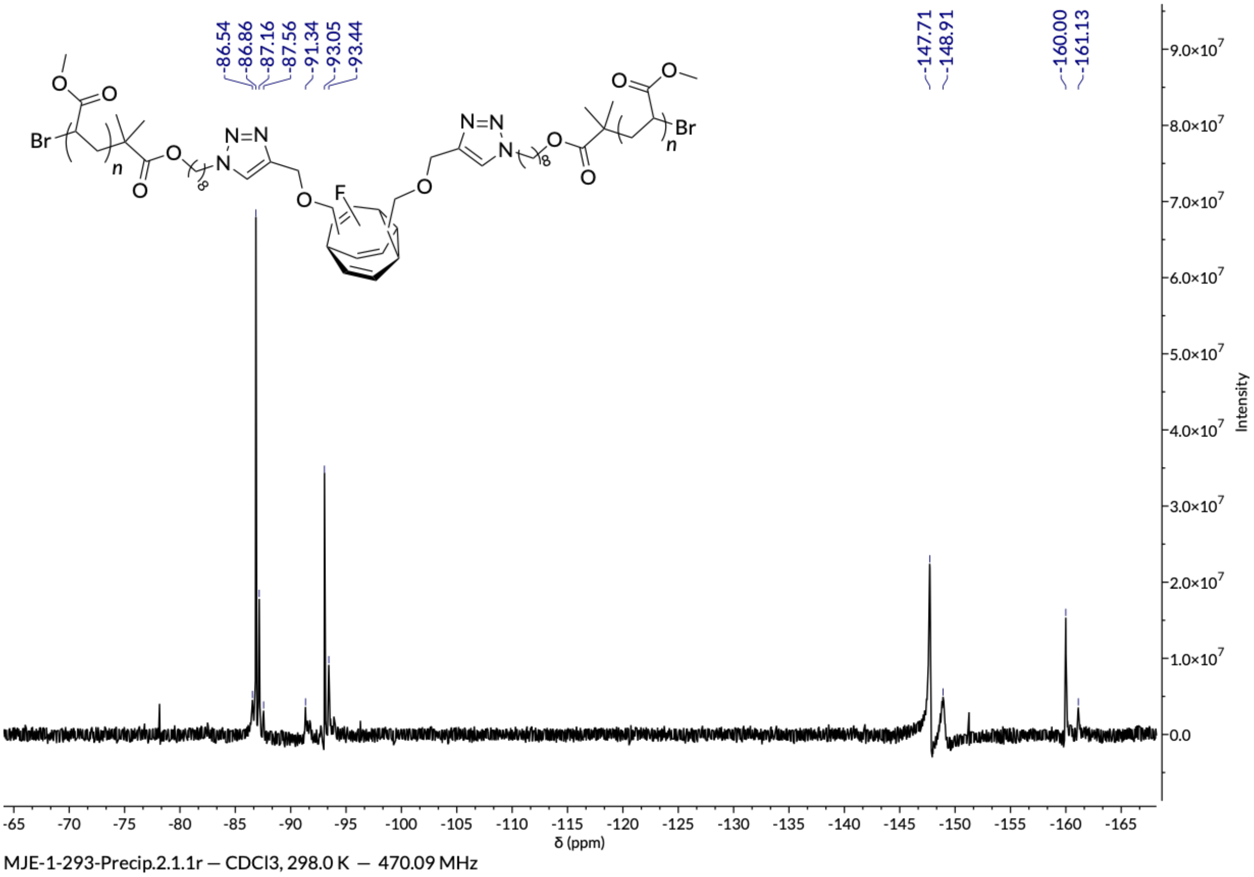

Figure S38: ^{19}F NMR spectrum of **F**-**Bull-PMA** ($CDCl_3$, 470 MHz)

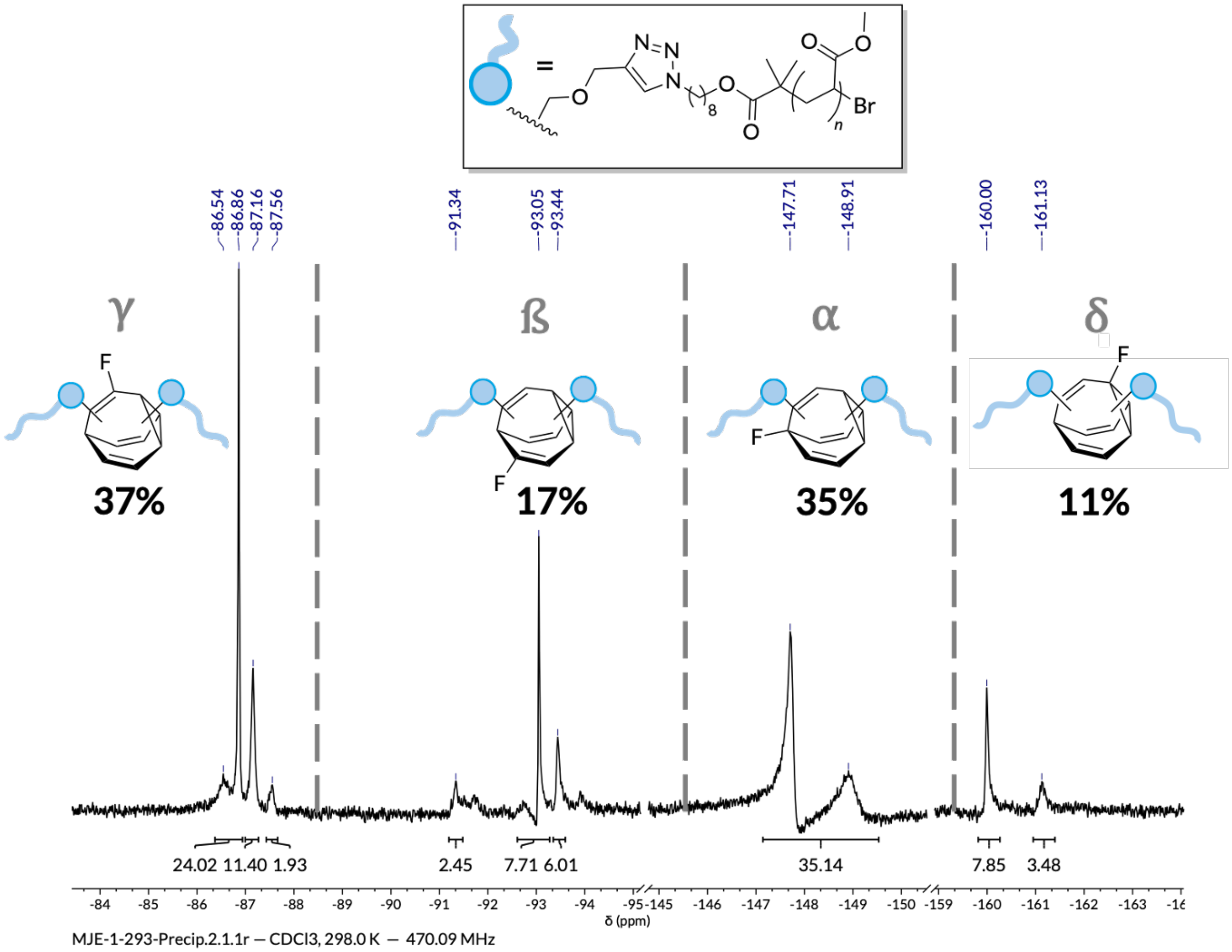

Figure S39: Annotated ^{19}F NMR spectrum of **F-Bull-PMA** ($CDCl_3$, 470 MHz)

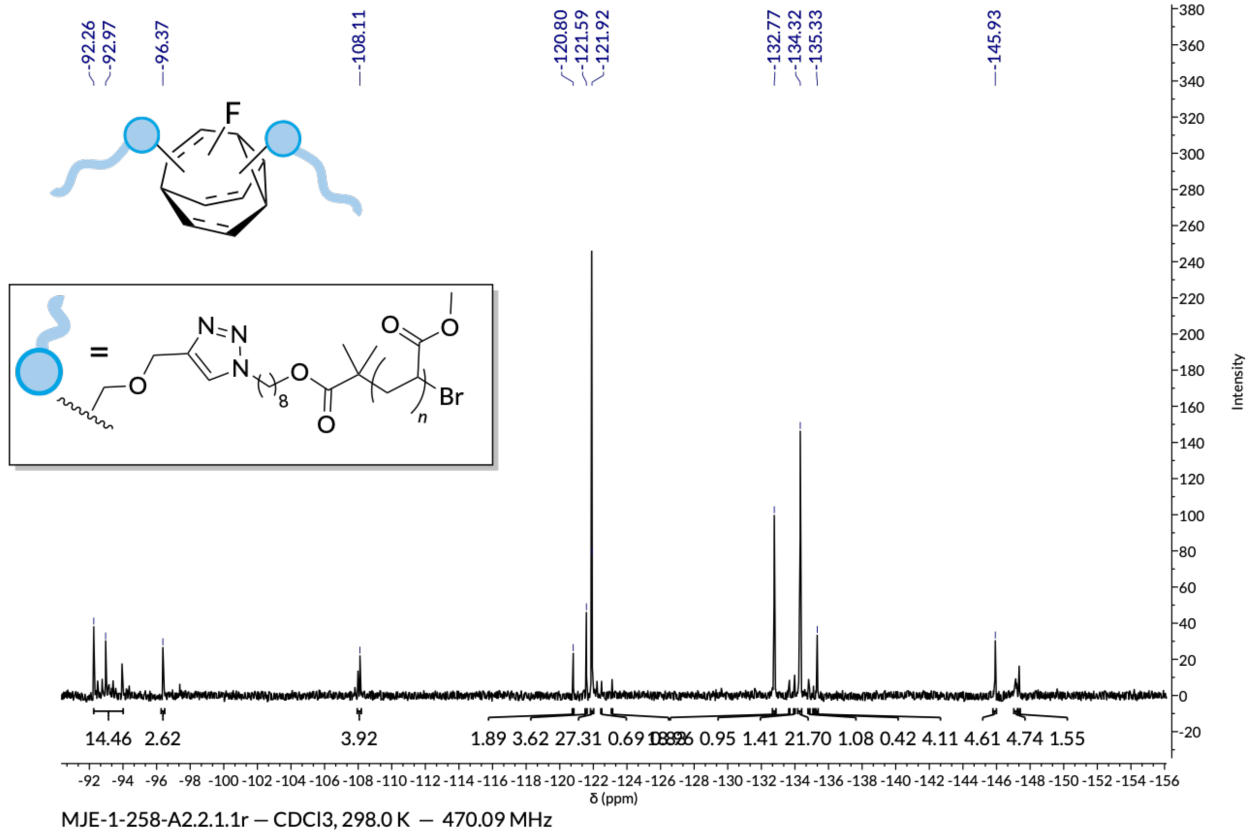

Figure S40: ^{19}F NMR spectrum of **F-Bull-PMA-Red-Sonics** ($CDCl_3$, 470 MHz)

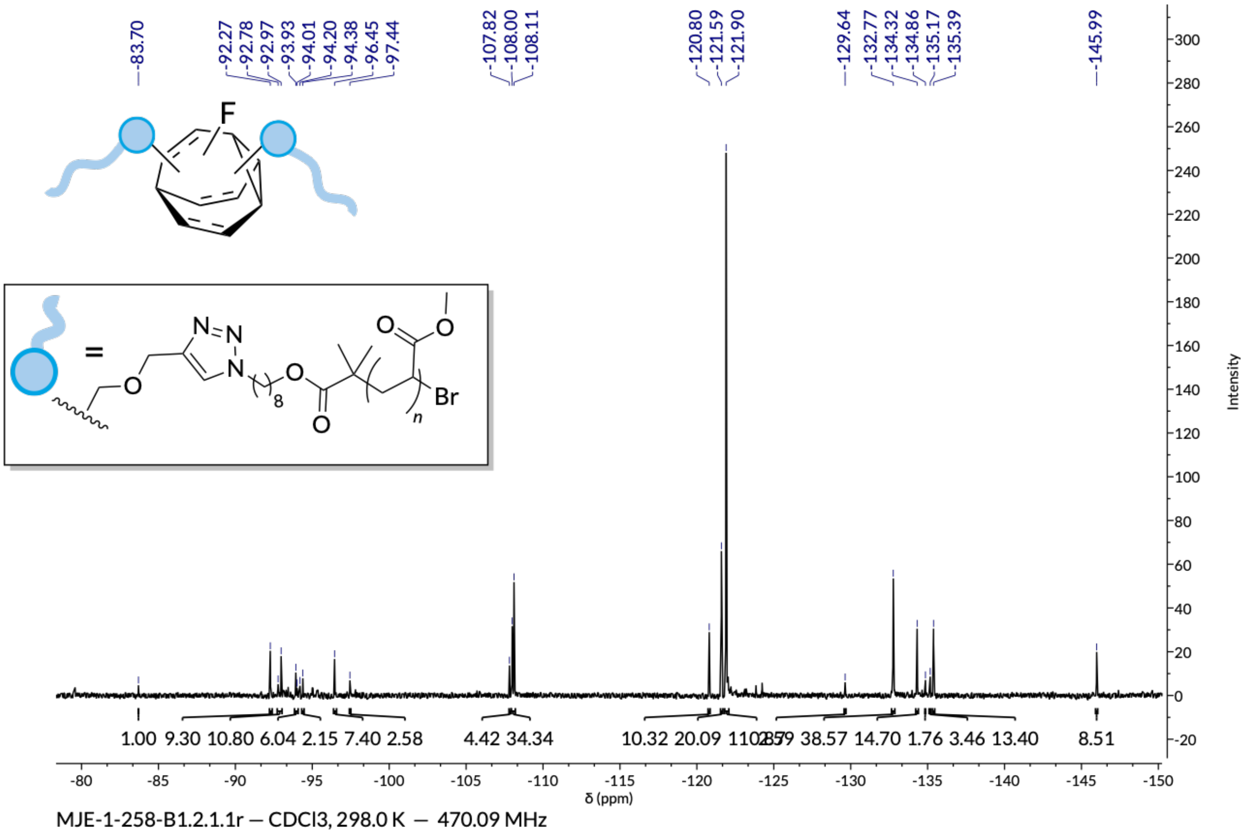

Figure S41: ^{19}F NMR spectrum of **F-Bull-PMA-Red-Stir** ($CDCl_3$, 470 MHz)

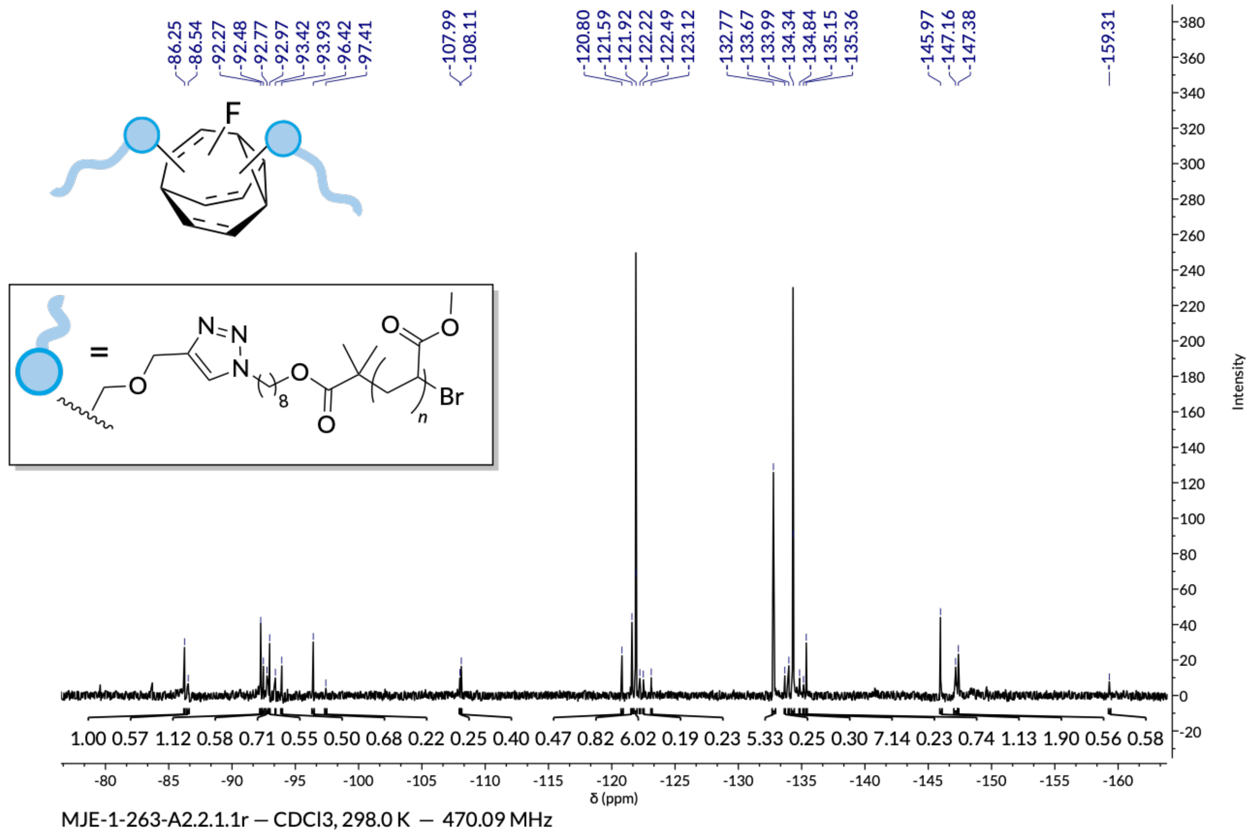

Figure S42: ^{19}F NMR spectrum of **F-Bull-PMA-Red-Sonics-Cold** ($CDCl_3$, 470 MHz)

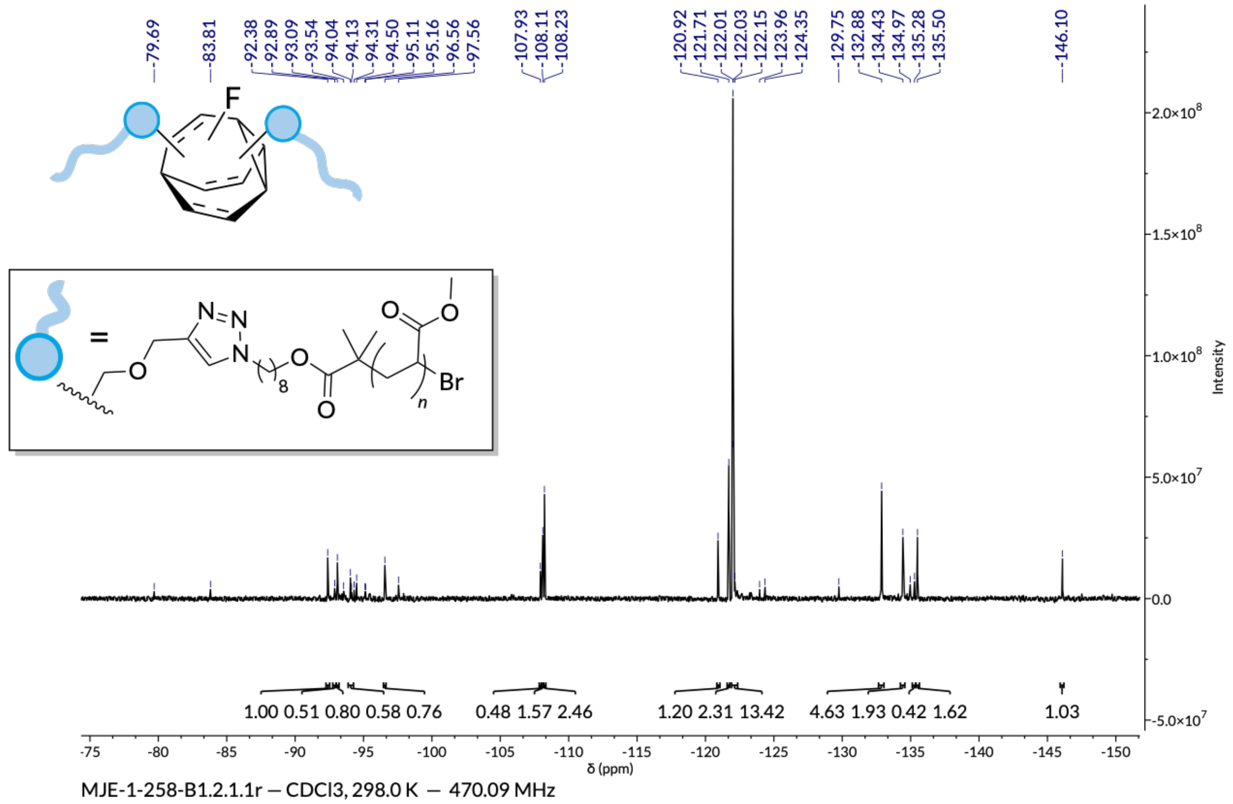

Figure S43: ^{19}F NMR spectrum of **F-Bull-PMA-Red-Stir-Cold** ($CDCl_3$, 470 MHz)

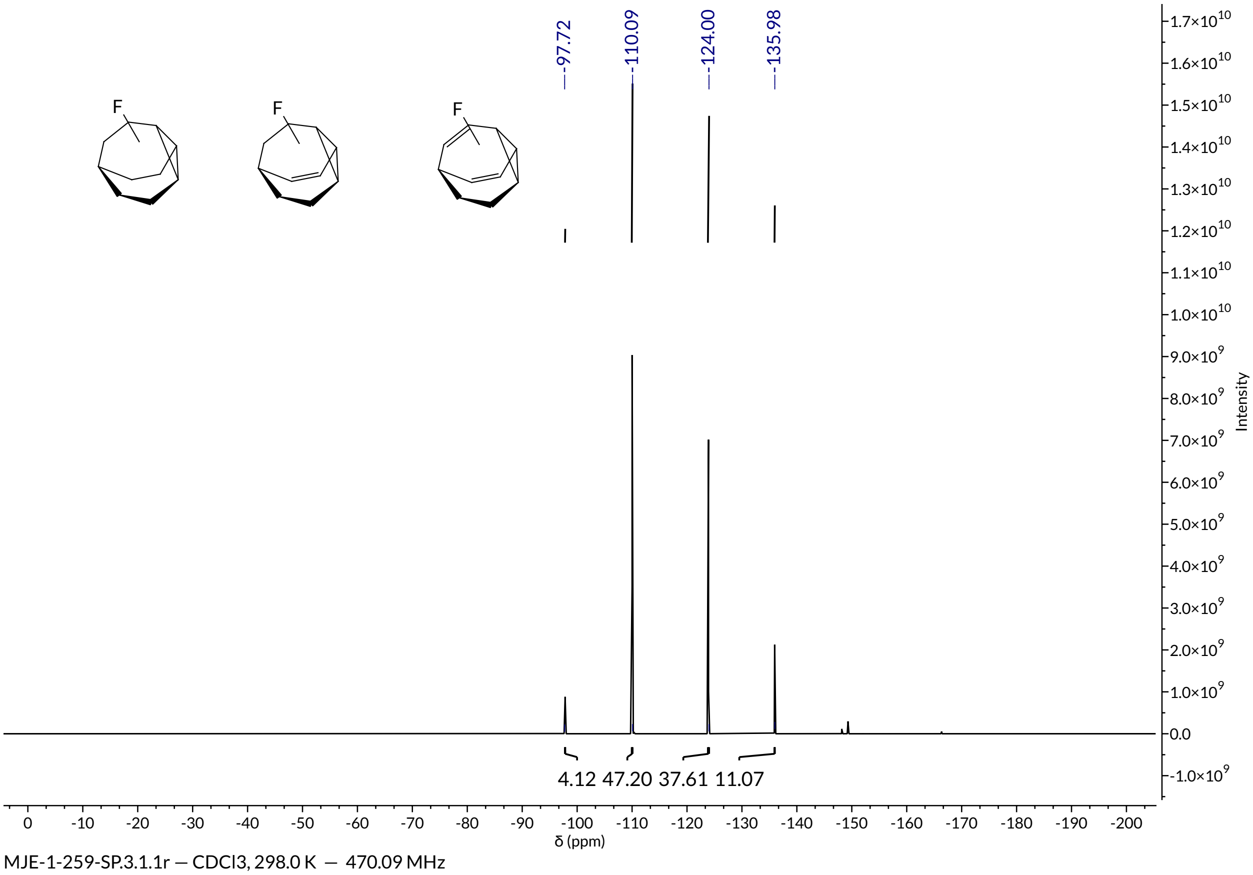

Figure S44: ^{19}F NMR spectrum of **F-Bull-Red^{n}** (CDCl$_3$, 470 MHz)

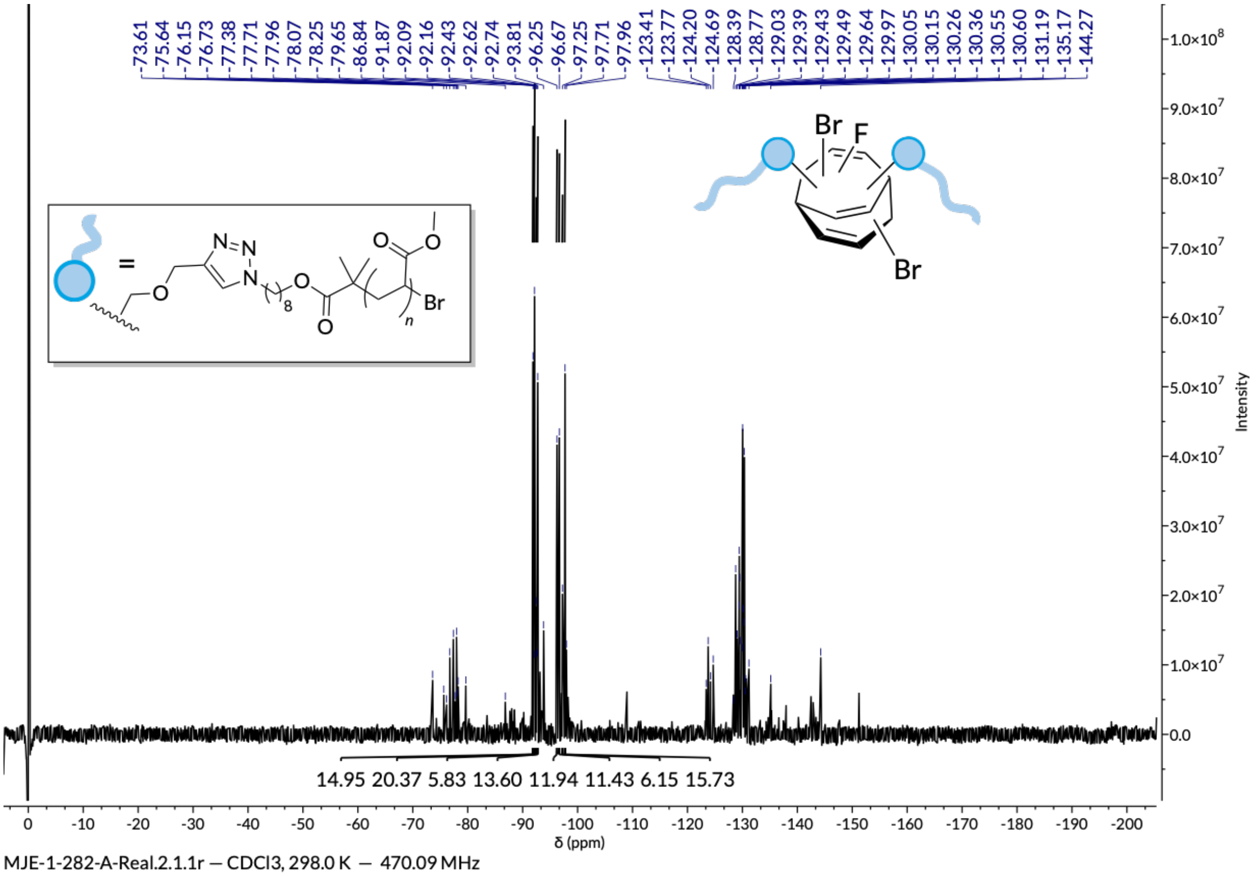

Figure S45: ^{19}F NMR spectrum of **F-Bull-PMA-Br$_2$-Cold** ($CDCl_3$, 470 MHz)

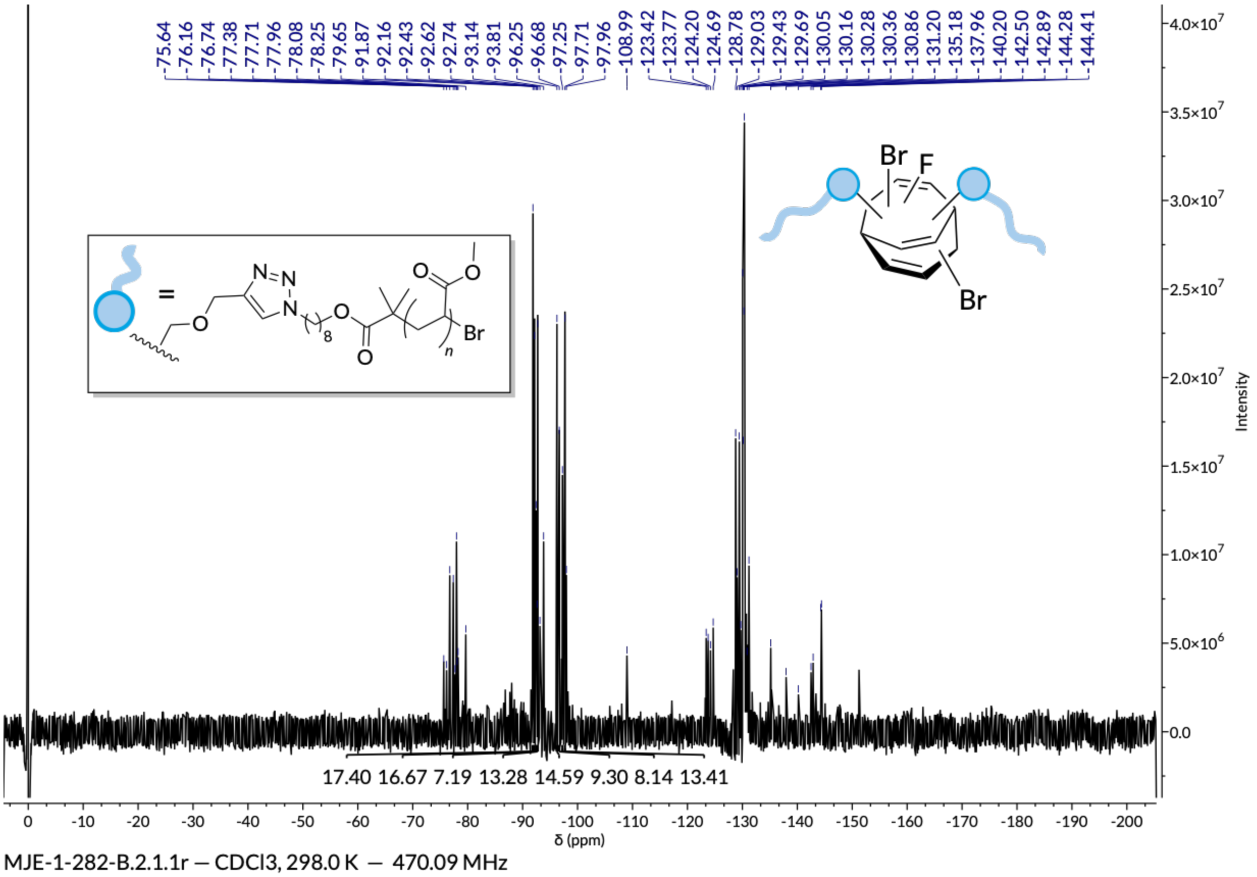

Figure S46: ^{19}F NMR spectrum of **F-Bull-PMA-Br$_2$-Warmed** ($CDCl_3$, 470 MHz)

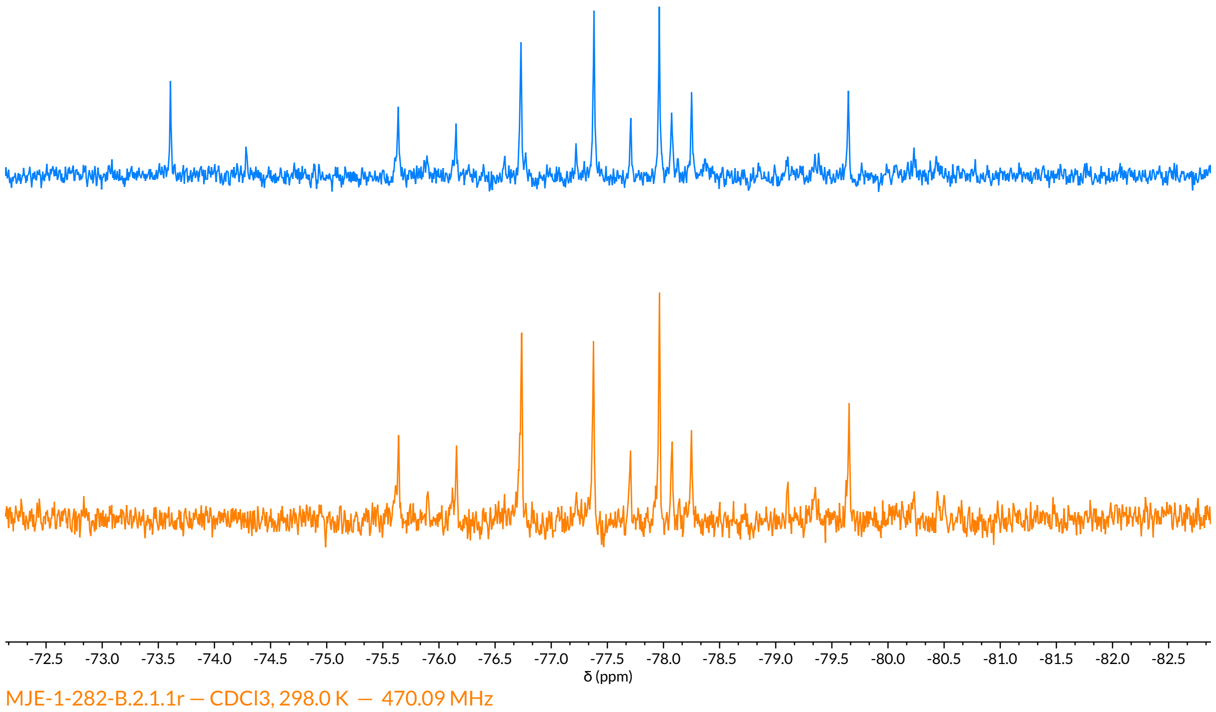

Figure S47: Stacked ^{19}F NMR spectrum of **F-Bull-PMA-Br$_2$-Cold** (top) and **F-Bull-PMA-Br$_2$-Warmed** (bottom) zoomed into the –70 to –80 ppm region ($CDCl_3$, 470 MHz)

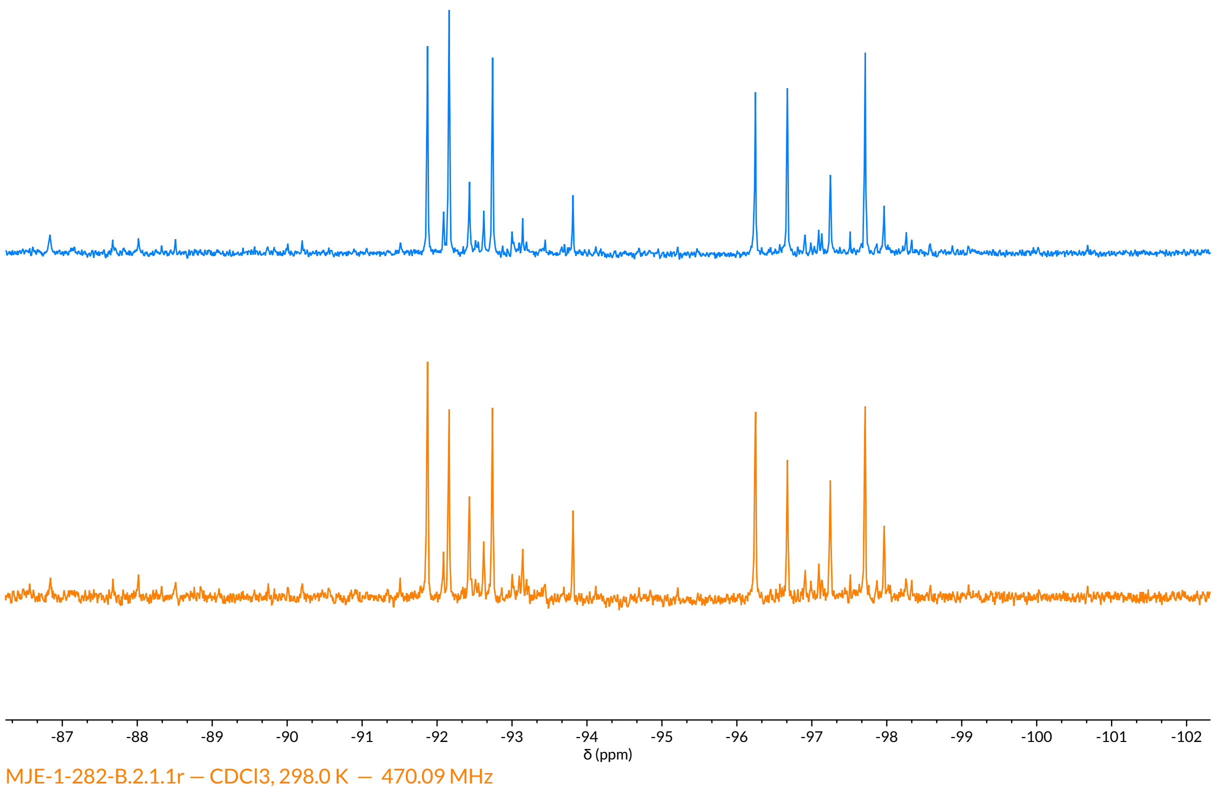

Figure S48: Stacked ^{19}F NMR spectrum of **F-Bull-PMA-Br$_2$-Cold** (top) and **F-Bull-PMA-Br$_2$-Warmed** (bottom) zoomed into the –90 to –100 ppm region ($CDCl_3$, 470 MHz)

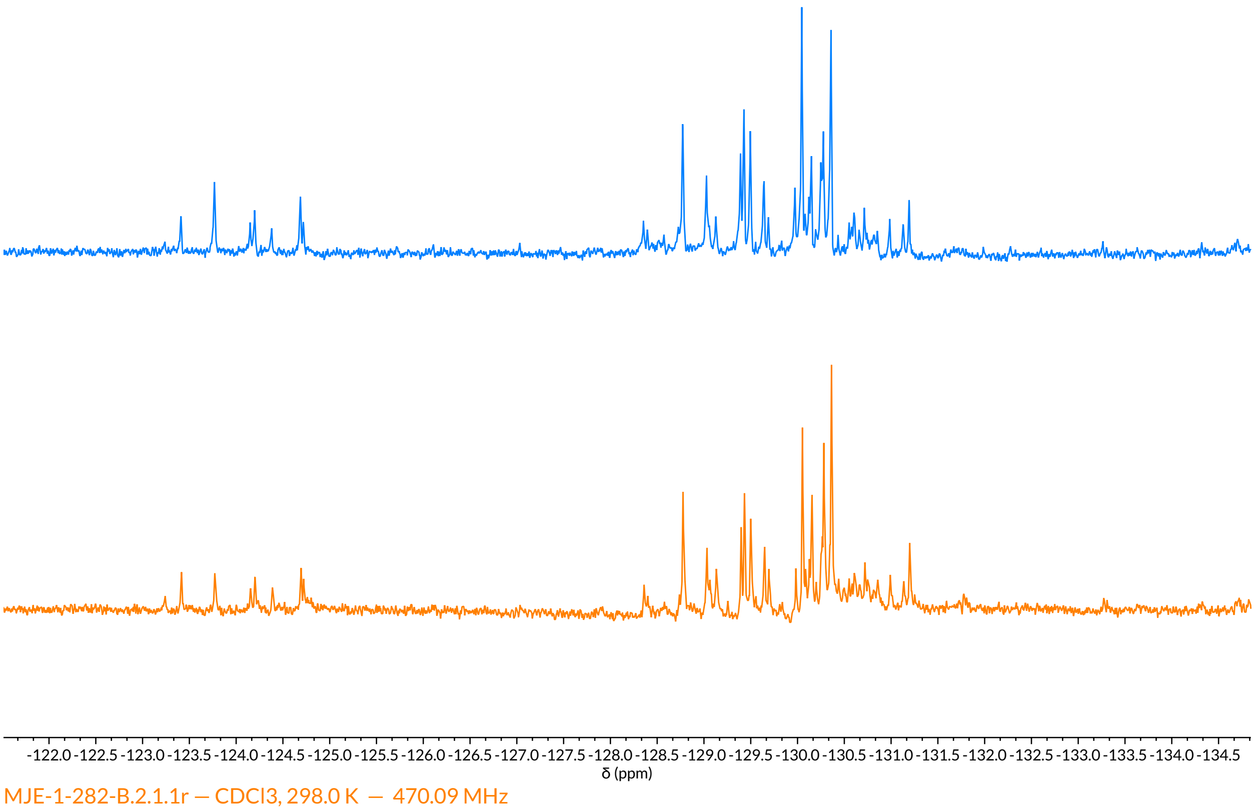

Figure S49: Stacked ^{19}F NMR spectrum of **F-Bull-PMA-Br$_2$-Cold** (top) and **F-Bull-PMA-Br$_2$-Warmed** (bottom) zoomed into the –120 to –130 ppm region ($CDCl_3$, 470 MHz)

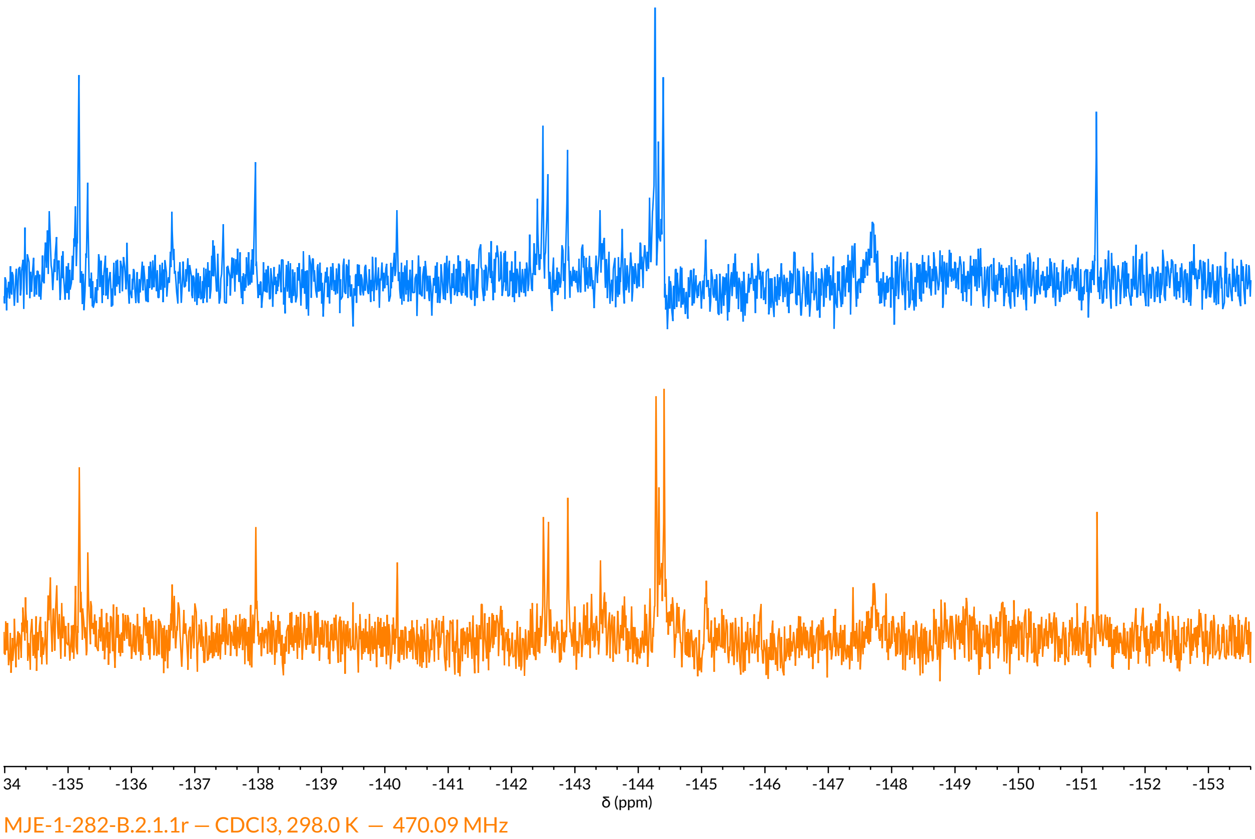

Figure S50: Stacked ^{19}F NMR spectrum of **F-Bull-PMA-Br$_2$-Cold** (top) and **F-Bull-PMA-Br$_2$-Warmed** (bottom) zoomed into the –130 to –150 ppm region ($CDCl_3$, 470 MHz)

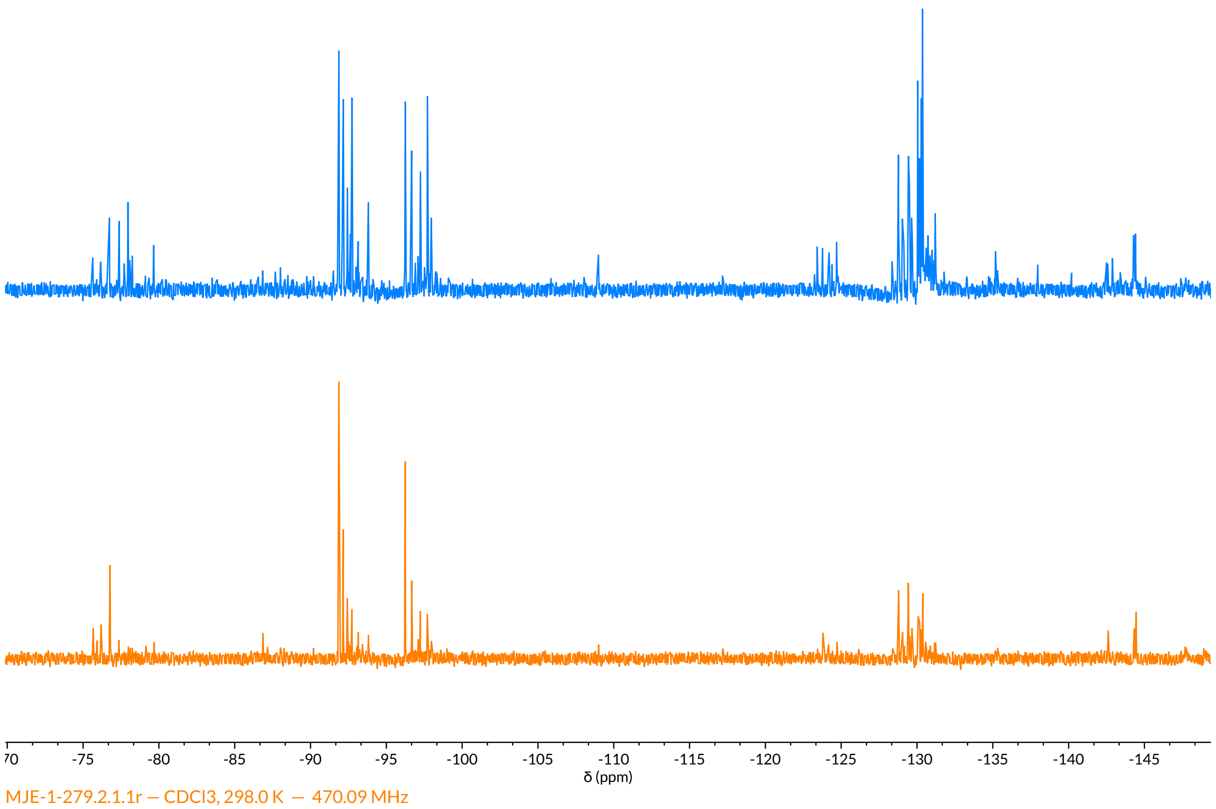

Figure S51: Full stacked ^{19}F NMR spectra comparing **F-Bull-PMA-Br$_2$-FF** (bottom) and **F-Bull-PMA-Br$_2$-Warmed** (top) ($CDCl_3$, 470 MHz).

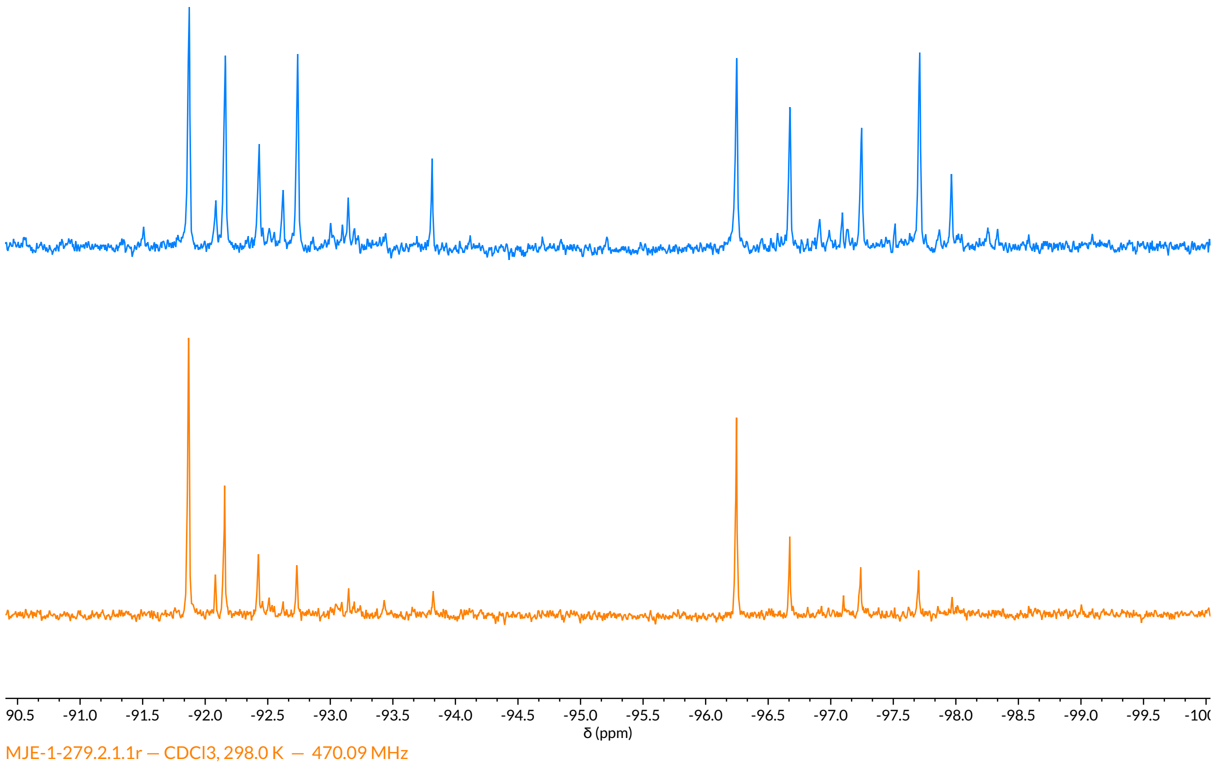

Figure S52: ^{19}F NMR spectra comparing **F-Bull-PMA-Br$_2$-FF** (bottom) and **F-Bull-PMA-Br$_2$-Warmed** (top) zoomed into the –90 to –100 ppm region ($CDCl_3$, 470 MHz).

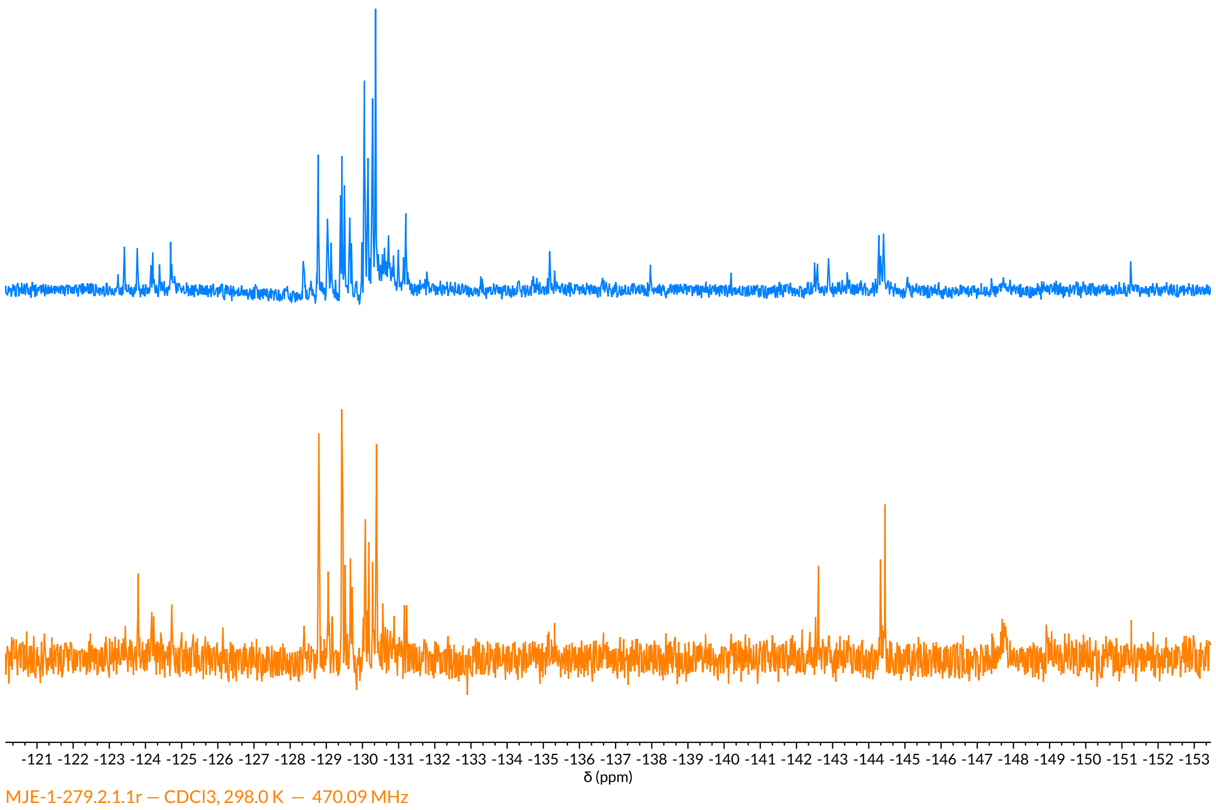

Figure S53: ^{19}F NMR spectra comparing **F-Bull-PMA-Br$_2$-FF** (bottom) and **F-Bull-PMA-Br$_2$-Warmed** (top) zoomed into the –120 to –150 ppm region ($CDCl_3$, 470 MHz).

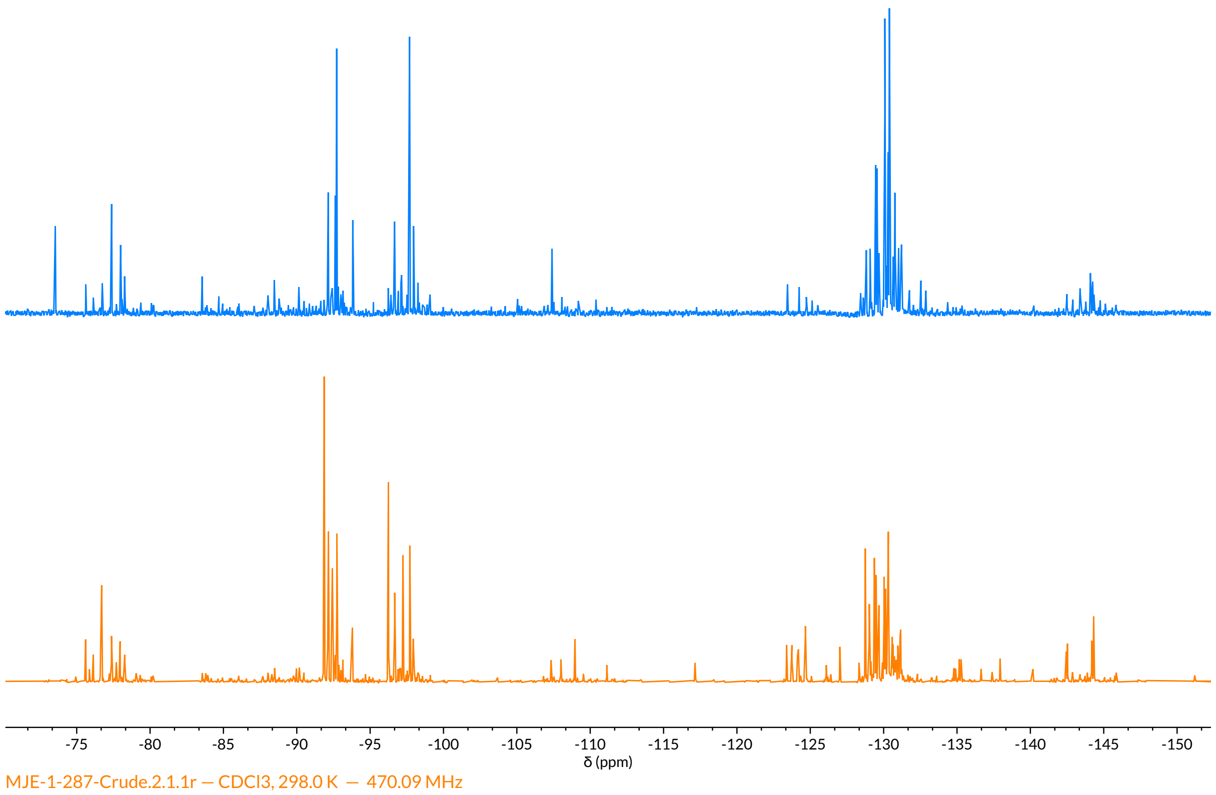

Figure S54: Full ^{19}F NMR spectra comparing **F-Bull-ATRP-Br$_2$-FF** (bottom) and **F-Bull-ATRP-Br$_2$-Warmed** (top) ($CDCl_3$, 470 MHz).

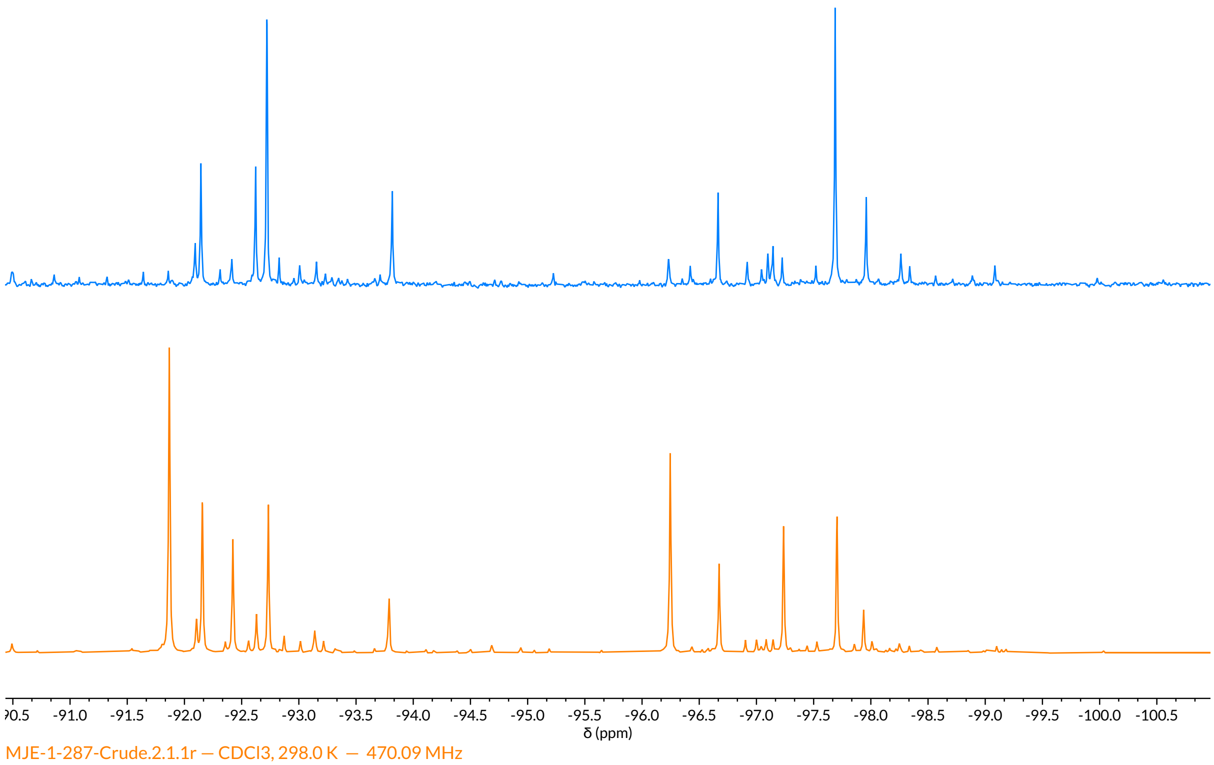

Figure S55: ^{19}F NMR spectra comparing **F-Bull-ATRP-Br$_2$-FF** (bottom) and **F-Bull-ATRP-Br$_2$-Warmed** (top) zoomed into the –90 to –100 ppm region ($CDCl_3$, 470 MHz).

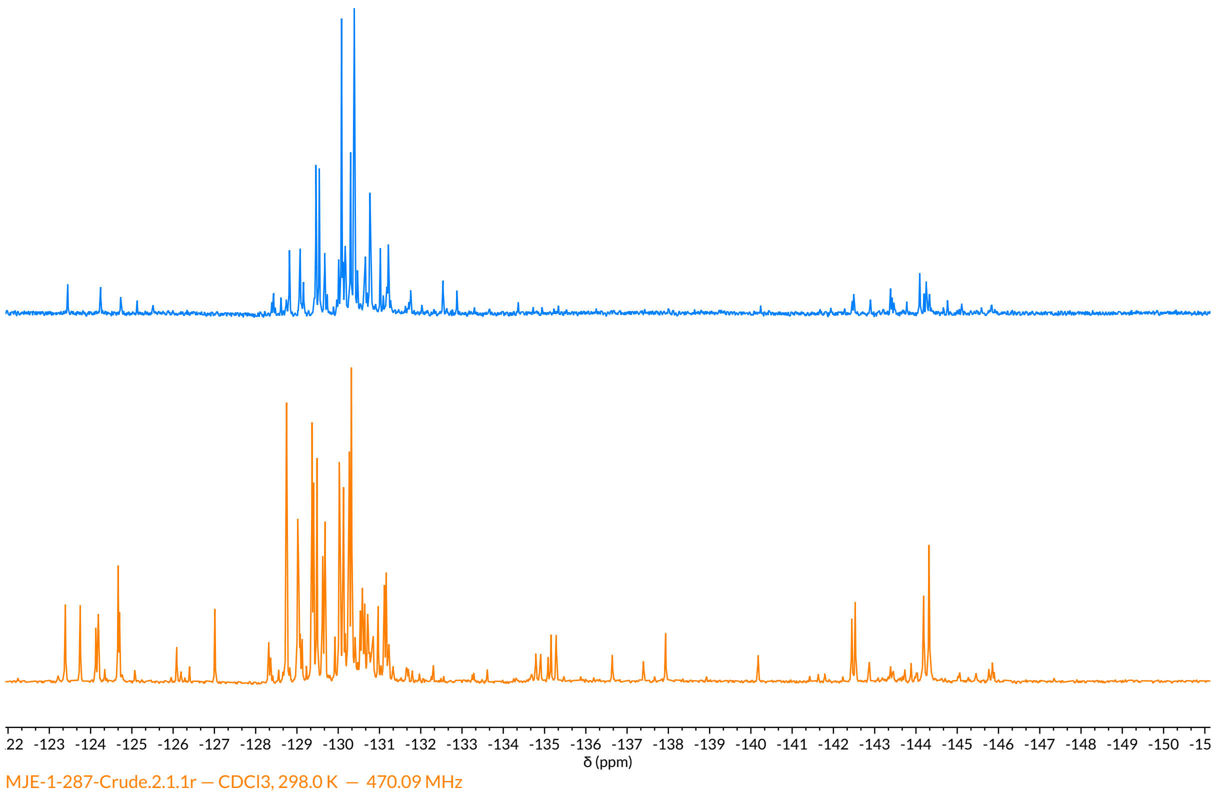

Figure S56: ^{19}F NMR spectra comparing **F-Bull-ATRP-Br$_2$-FF** (bottom) and **F-Bull-ATRP-Br$_2$-Warmed** (top) zoomed into the –120 to –150 ppm region ($CDCl_3$, 470 MHz).

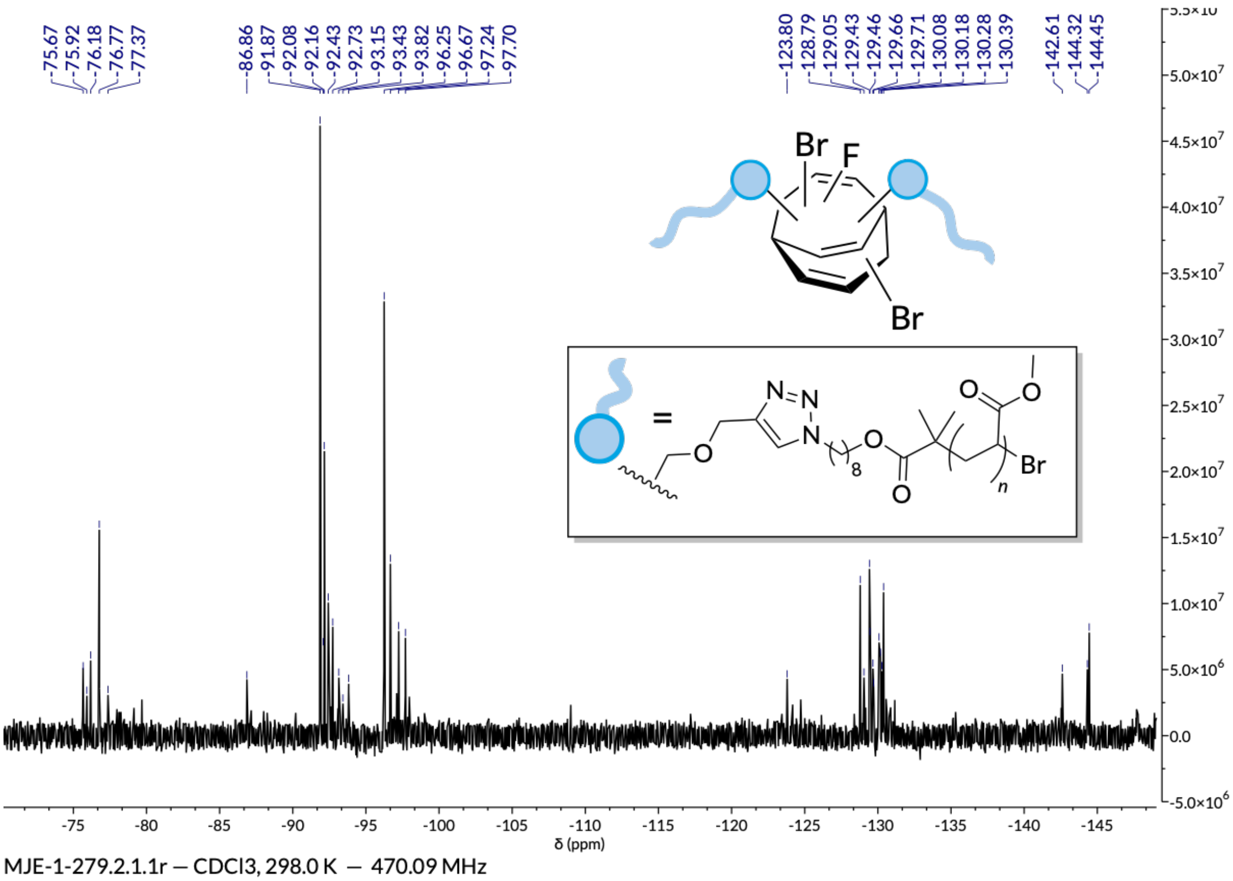

Figure S57: ^{19}F NMR spectrum of **F-Bull-PMA-Br$_2$-FF** ($CDCl_3$, 470 MHz)

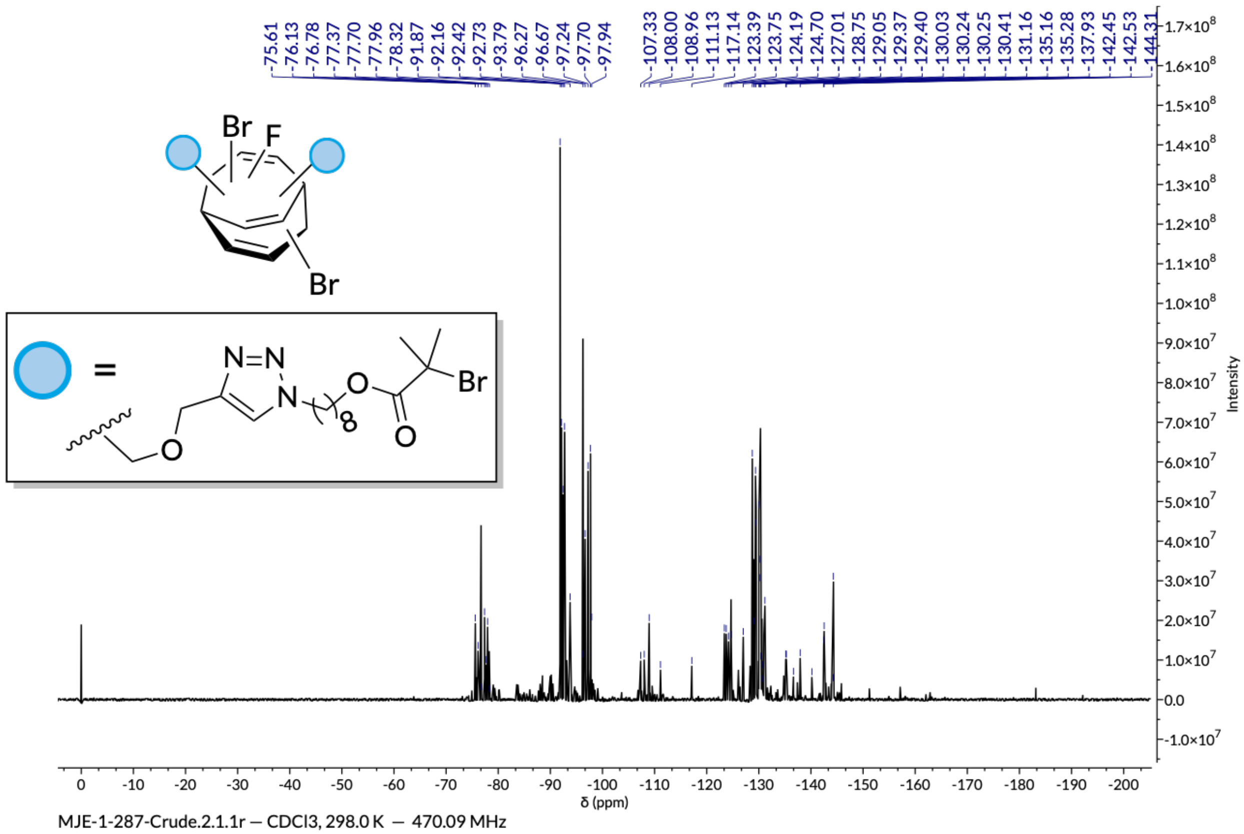

Figure S58: ^{19}F NMR spectrum of **F-Bull-ATRP-Br$_2$-FF** ($CDCl_3$, 470 MHz)

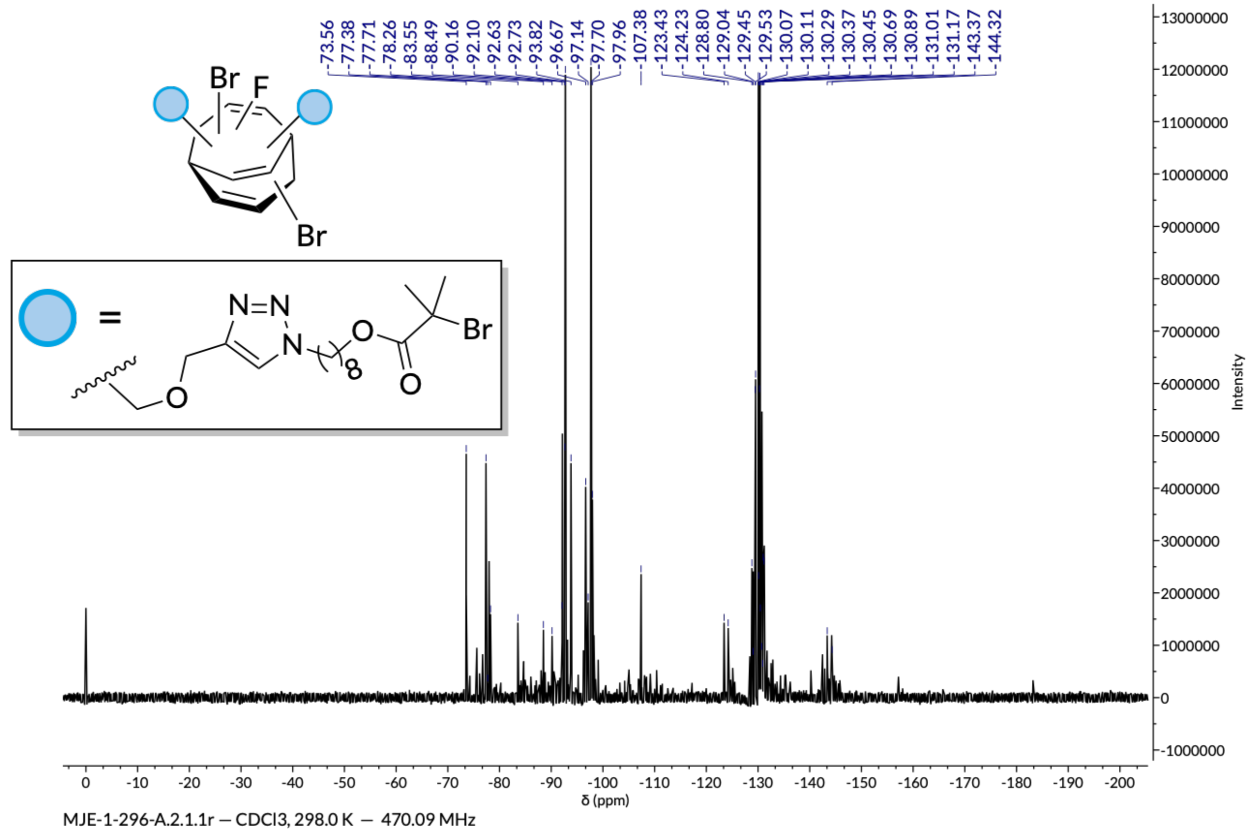

Figure S59: ^{19}F NMR spectrum of **F-Bull-ATRP-Br$_2$-Cold** ($CDCl_3$, 470 MHz)

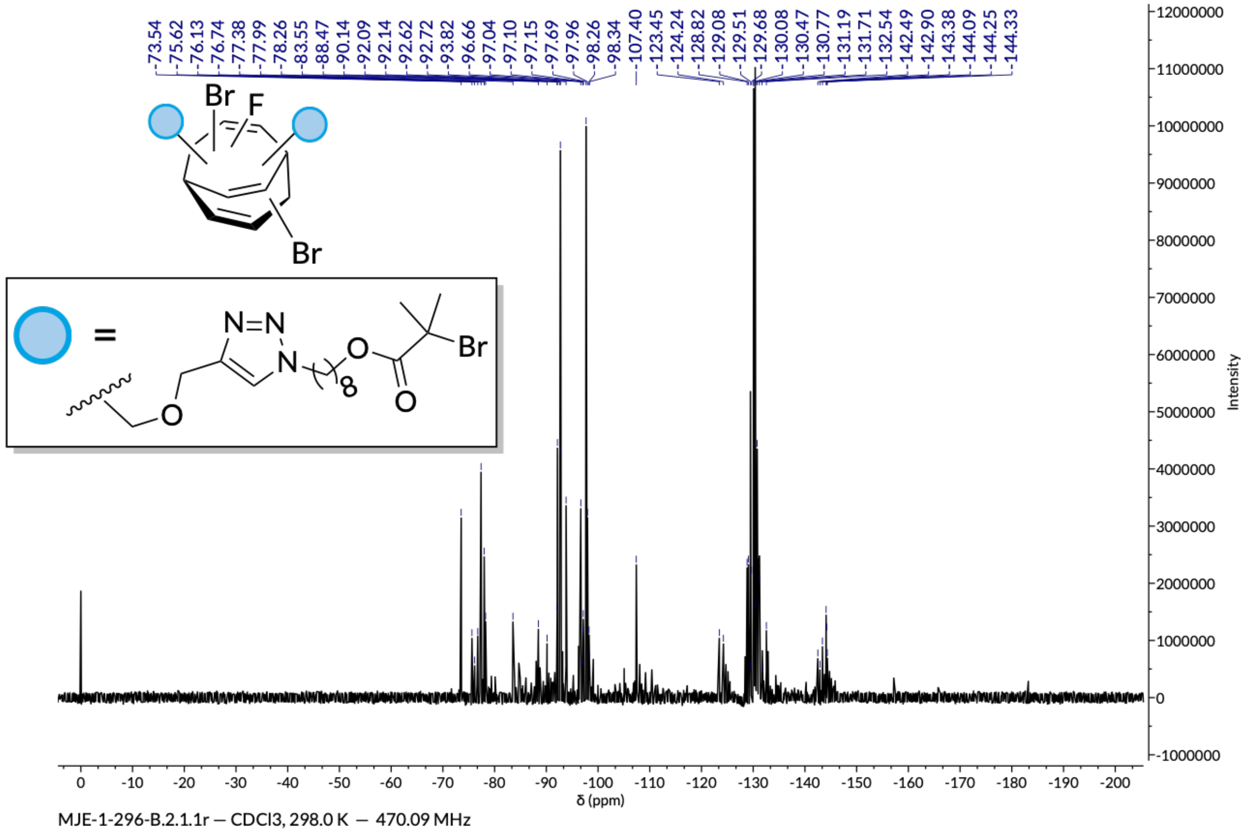

Figure S60: ^{19}F NMR spectrum of **F-Bull-ATRP-Br$_2$-Warmed** (CDCl$_3$, 470 MHz)

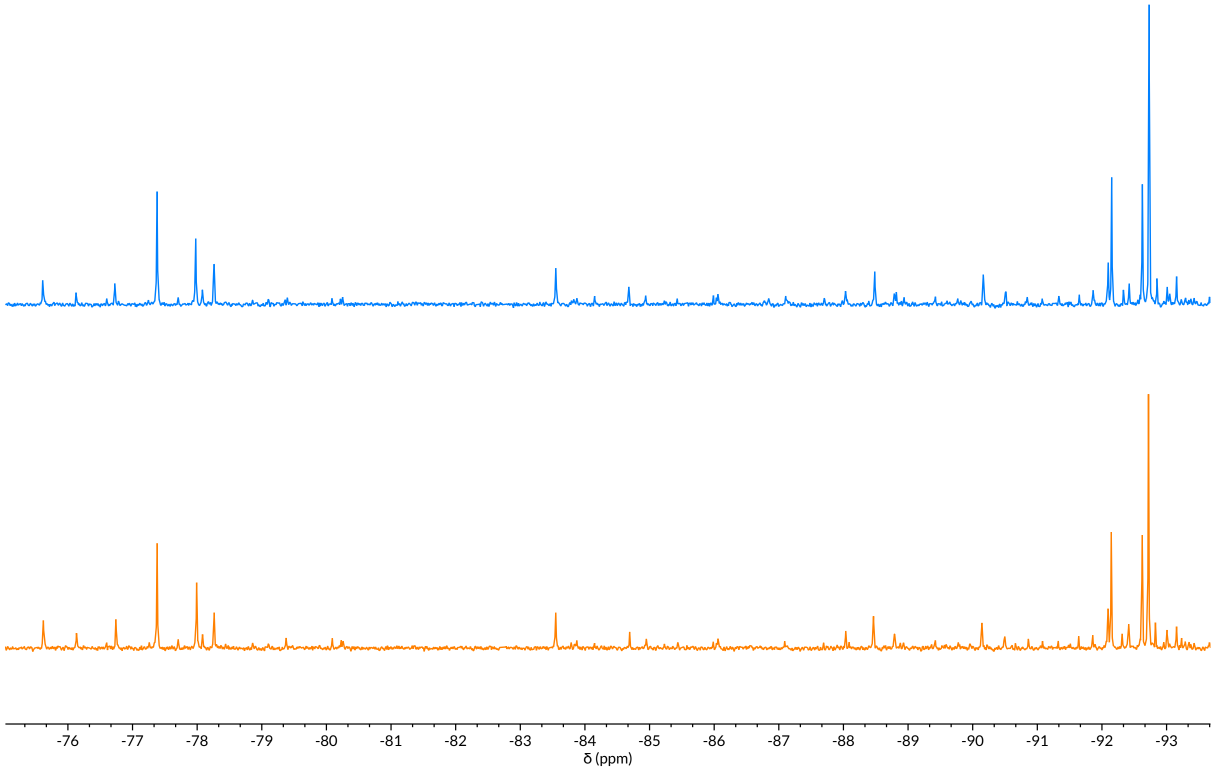

Figure S61: Stacked ^{19}F NMR spectra of **F-Bull-ATRP-Br$_2$-Cold** (top) and **F-Bull-ATRP-Br$_2$-Warmed** (bottom) zoomed into the –70 to –90 ppm region ($CDCl_3$, 470 MHz)

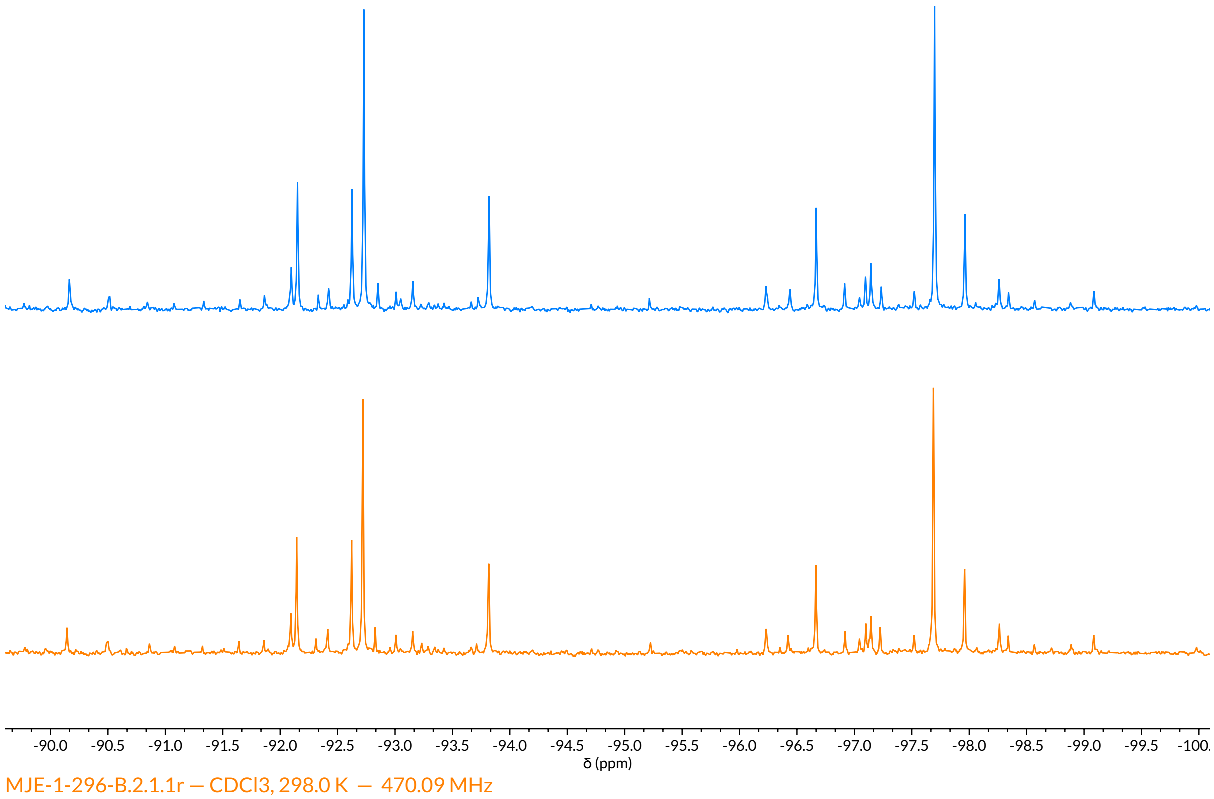

Figure S62: Stacked ^{19}F NMR spectra of **F-Bull-ATRP-Br$_2$-Cold** (top) and **F-Bull-ATRP-Br$_2$-Warmed** (bottom) zoomed into the –90 to –100 ppm region ($CDCl_3$, 470 MHz)

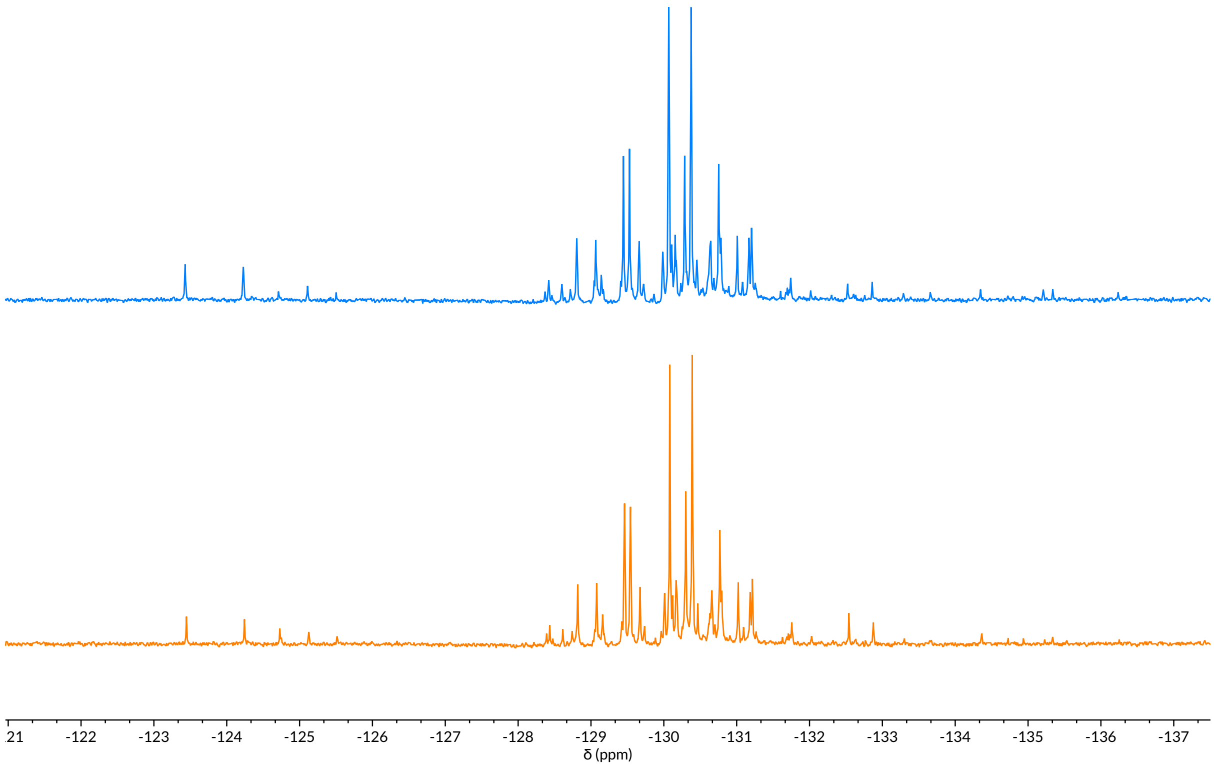

Figure S63: Stacked ^{19}F NMR spectra of **F-Bull-ATRP-Br$_2$-Cold** (top) and **F-Bull-ATRP-Br$_2$-Warmed** (bottom) zoomed into the –120 to –140 ppm region ($CDCl_3$, 470 MHz)

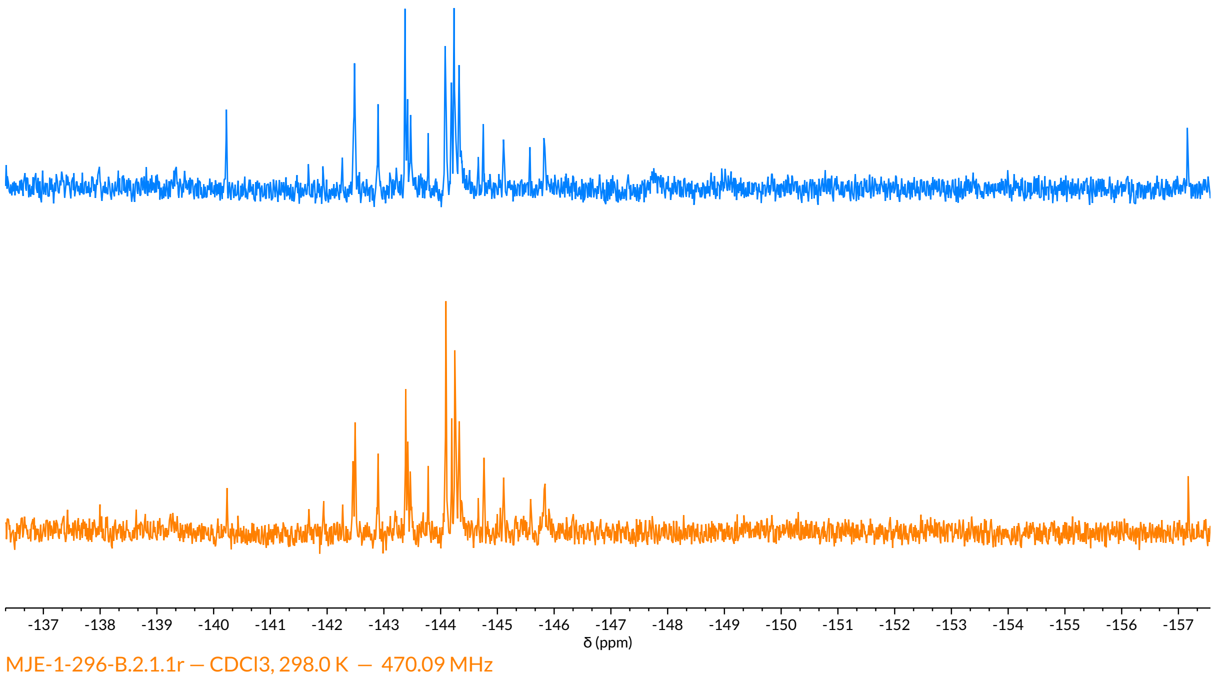

Figure S64: Stacked ^{19}F NMR spectra of **F-Bull-ATRP-Br_2-Cold** (top) and **F-Bull-ATRP-Br_2-Warmed** (bottom) zoomed into the –140 to –160 ppm region ($CDCl_3$, 470 MHz)

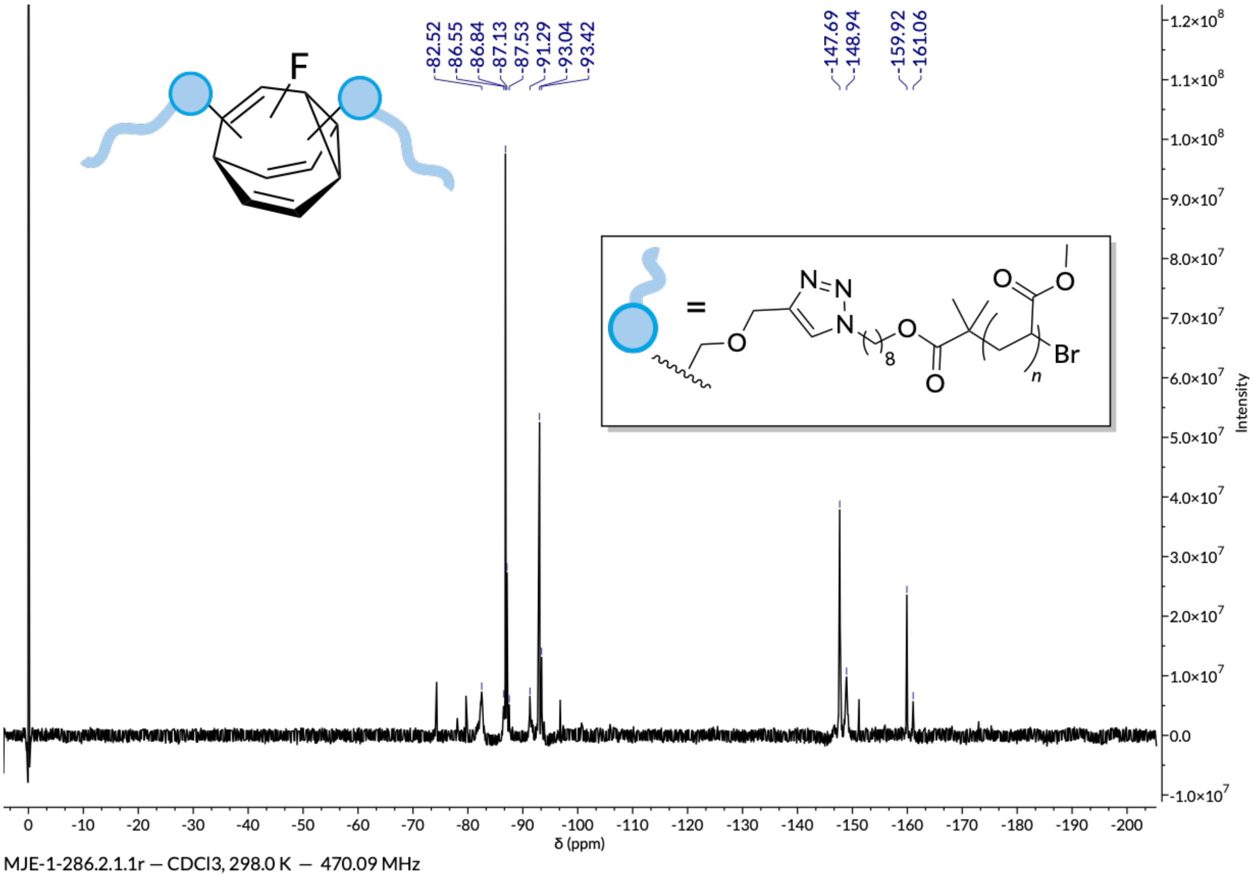

Figure S65: ^{19}F NMR spectrum of **F-Bull-PMA-Sonics-Control** ($CDCl_3$, 470 MHz)

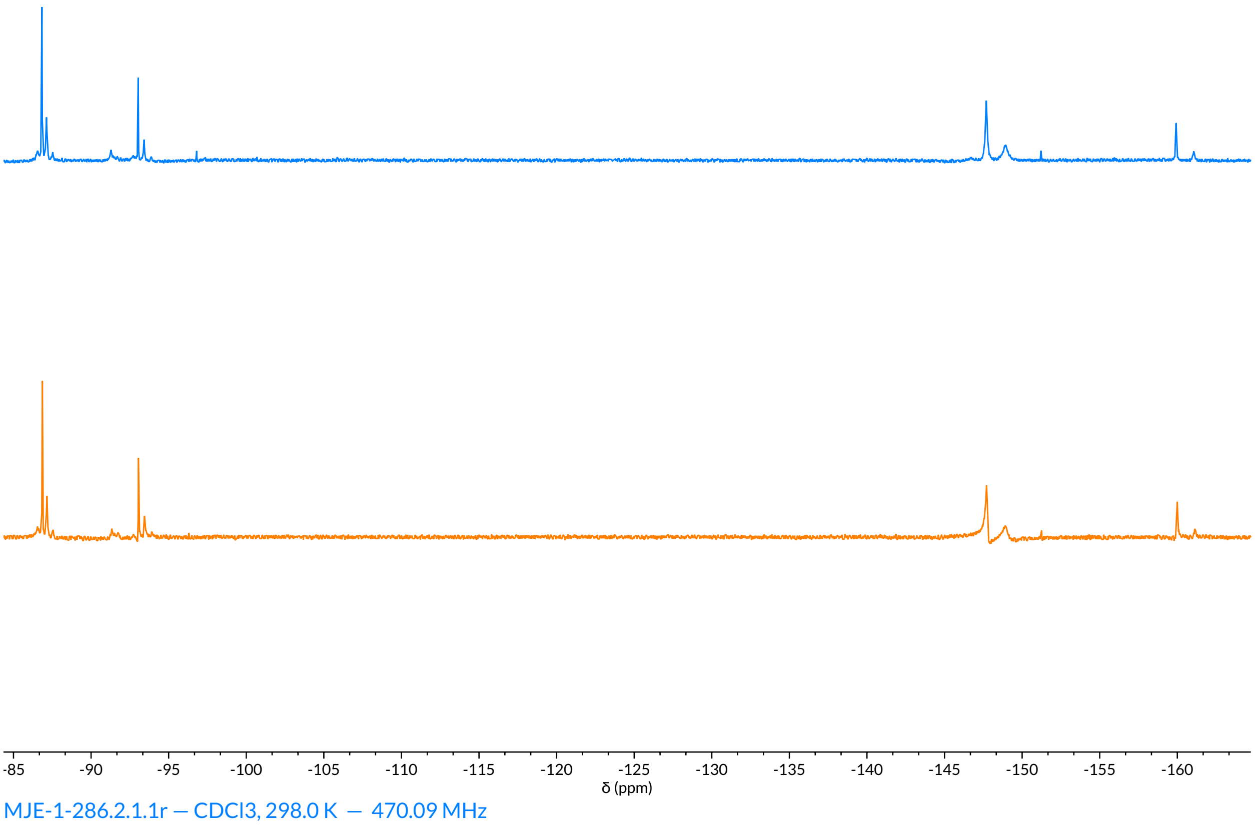

Figure S66: Stacked ^{19}F NMR spectra of **F-Bull-PMA-Sonics-Control** (top) and **F-Bull-PMA** (bottom) ($CDCl_3$, 470 MHz)

3.3: ^{13}C NMR SPECTRA

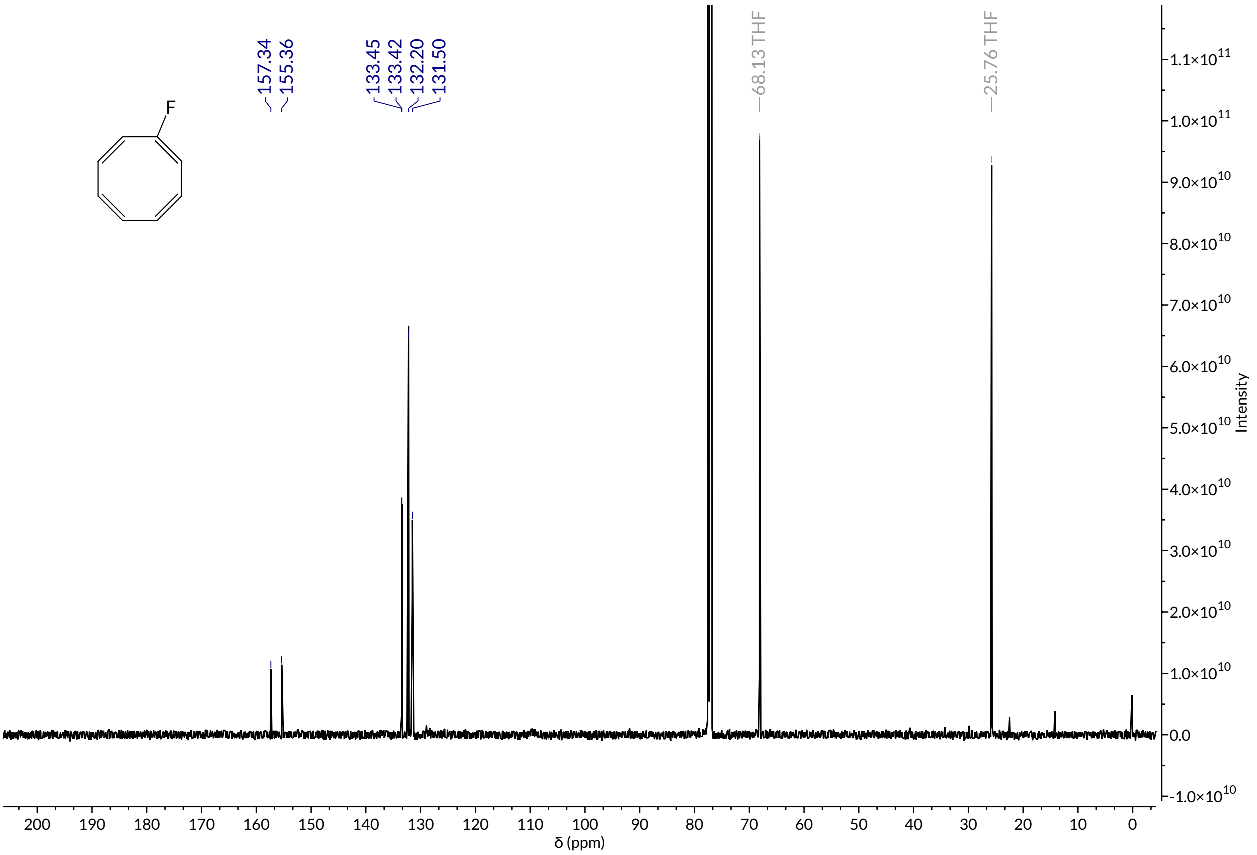

Figure S67: ^{13}C NMR spectrum of **F-COT** ($CDCl_3$, 126 MHz)

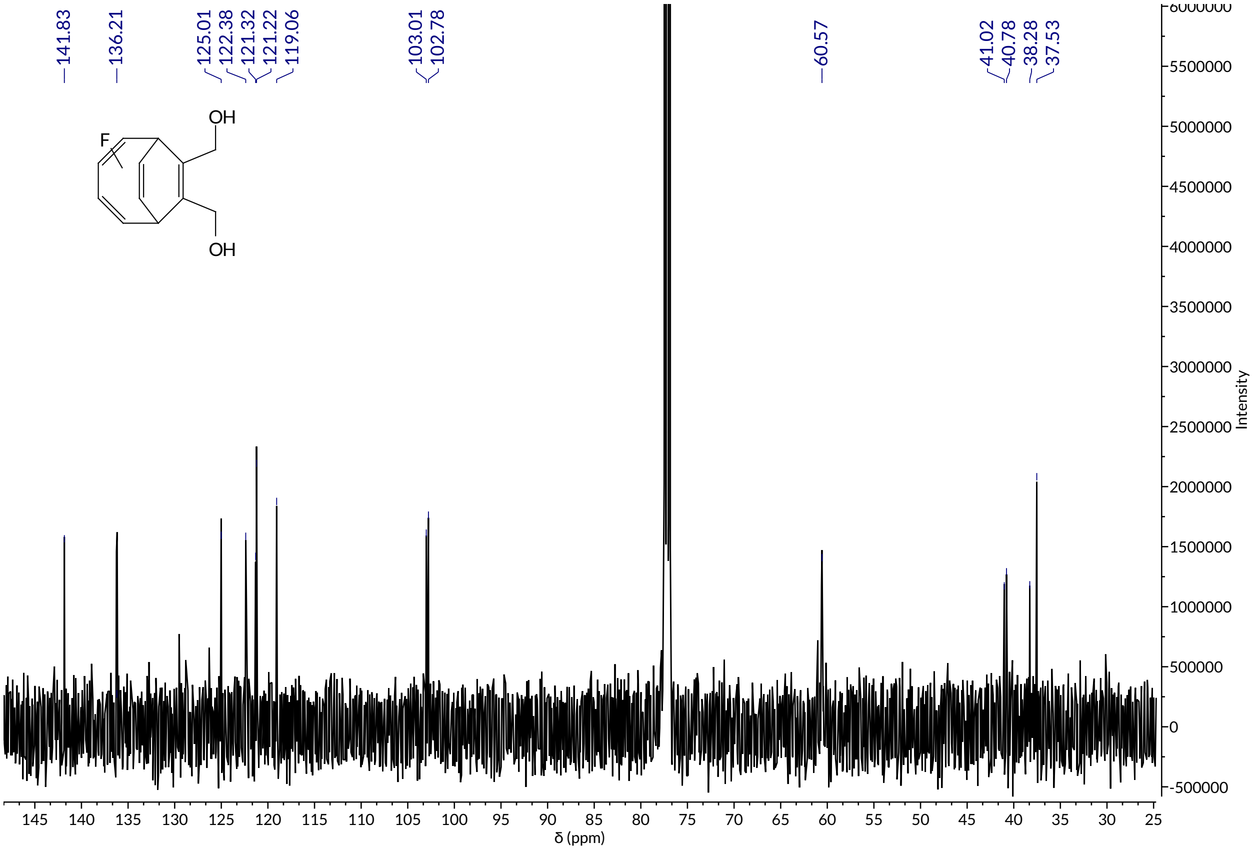

JDK-2-24_s3.3.1.1r – CDCl3, 298.0 K – 125.65 MHz

Figure S68: ^{13}C NMR spectrum of **F**-**CA-Diol** ($CDCl_3$, 126 MHz)

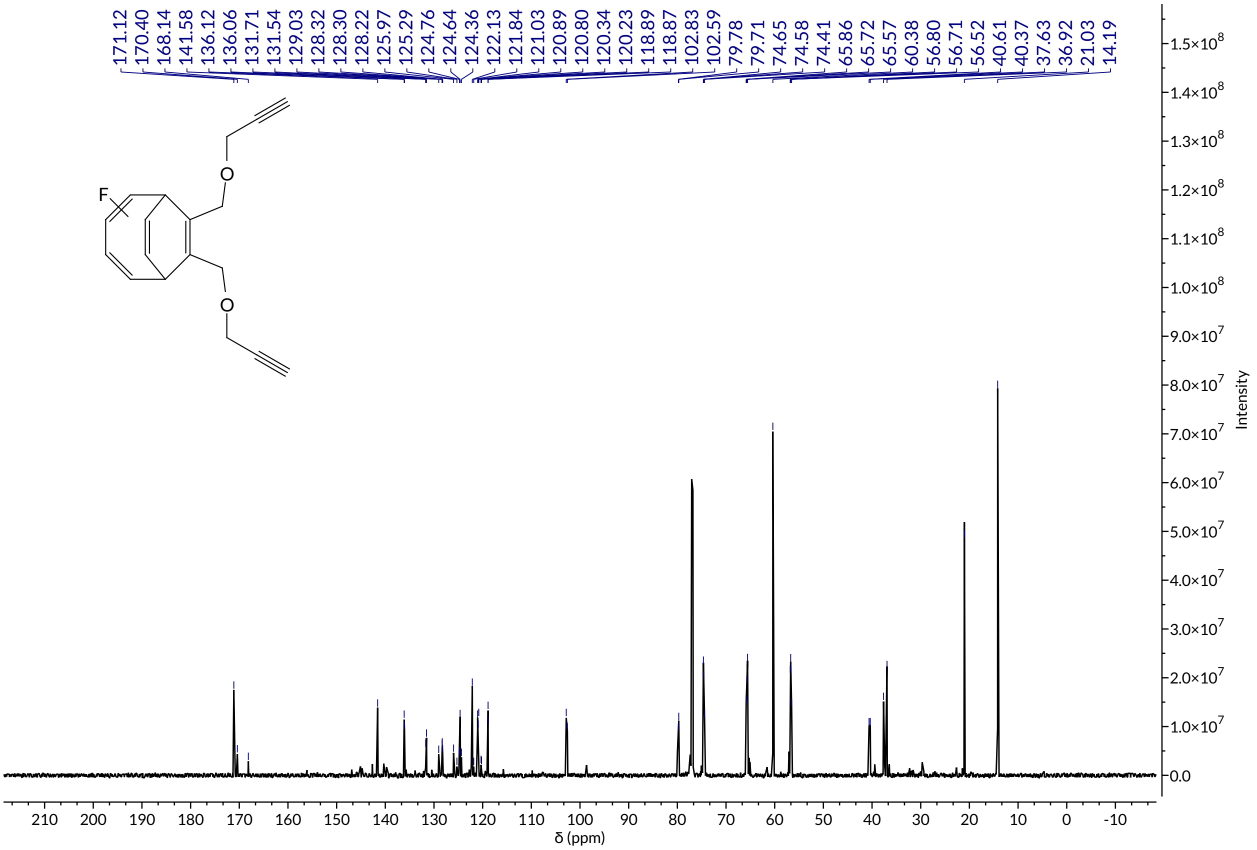

JDK-2-25_unspiked_13C.1.1.1r — CDCl3, 298.0 K — 125.65 MHz

Figure S69: ^{13}C NMR spectrum of **F-CA-PE** ($CDCl_3$, 126 MHz)

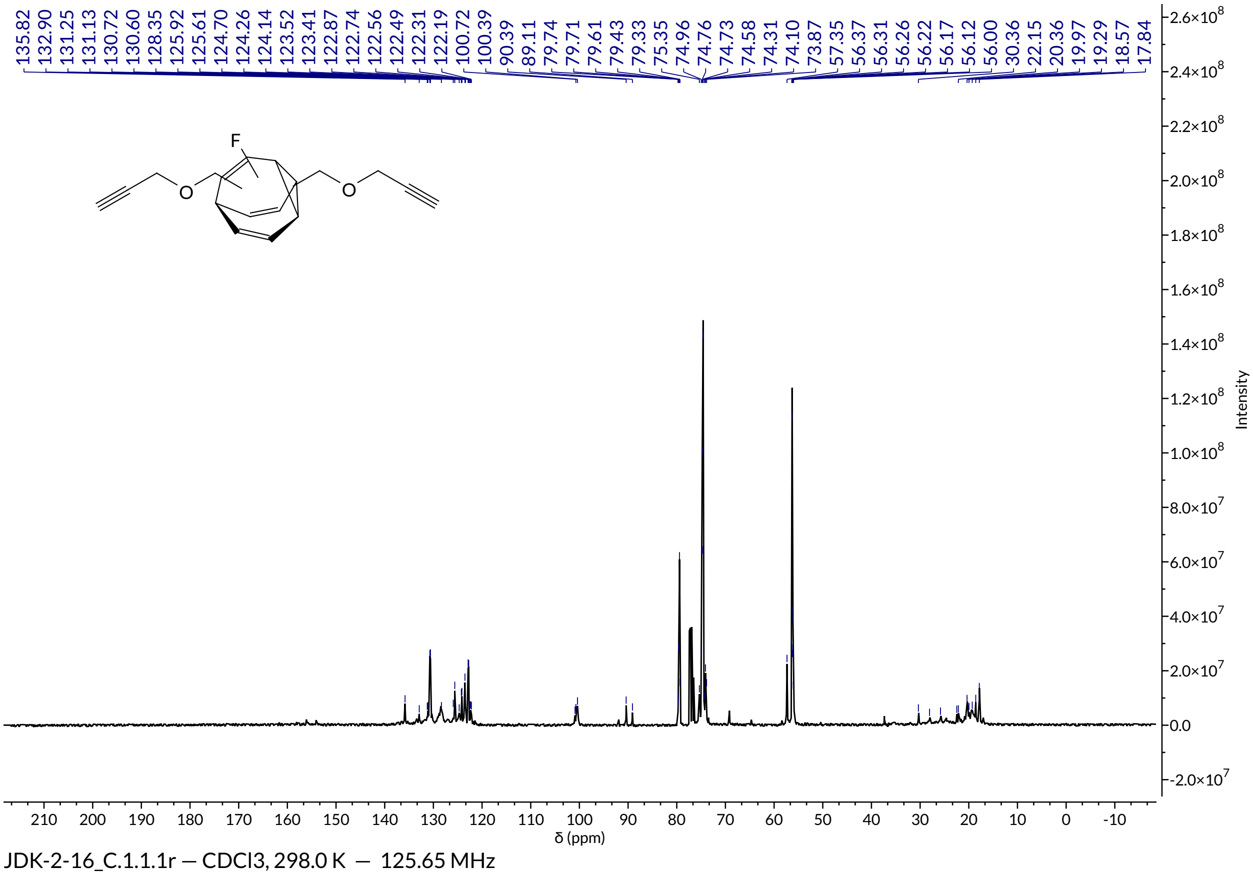

Figure S70: ^{13}C NMR spectrum of **F-Bull-PE** ($CDCl_3$, 126 MHz)

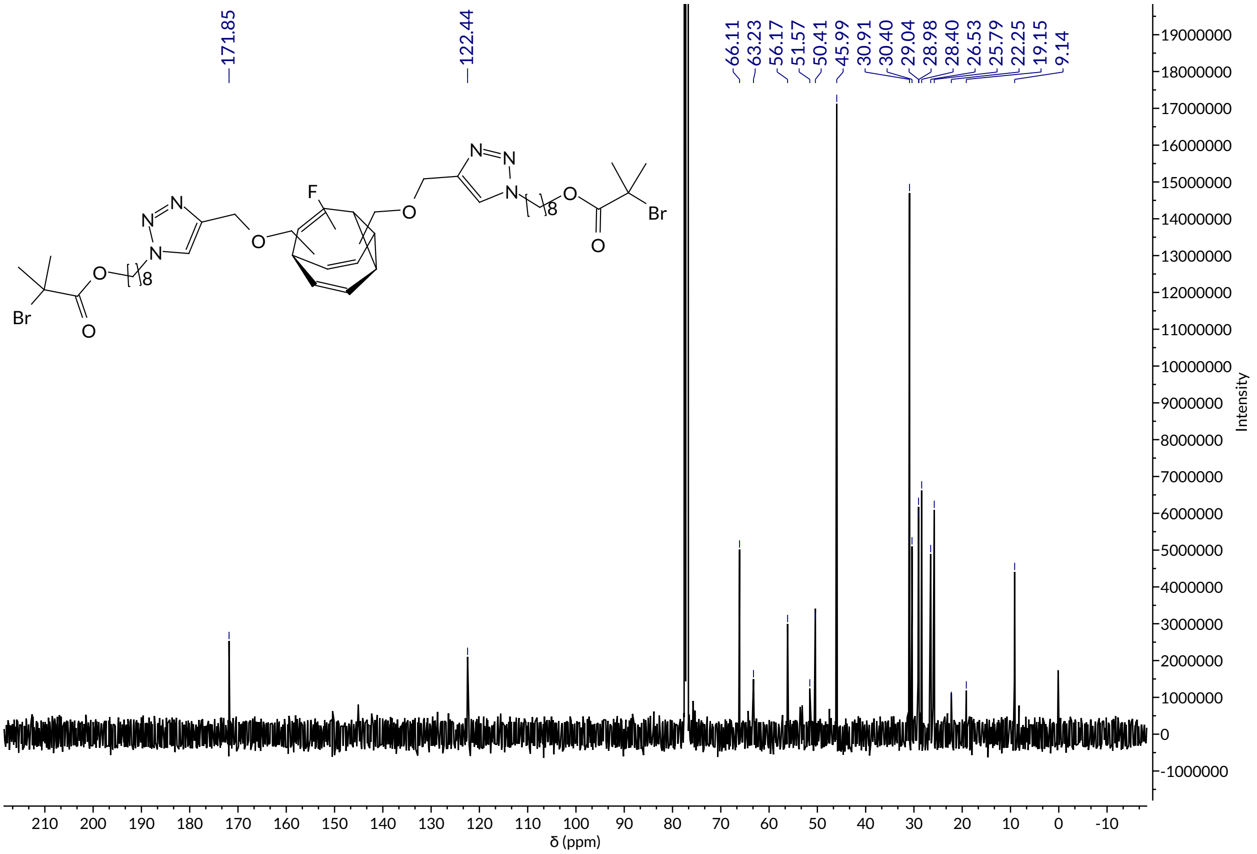

MJE-1-253-Column.3.1.1r — CDCl3, 298.0 K — 125.65 MHz

Figure S71: ^{13}C NMR spectrum of **F-Bull-ATRP** ($CDCl_3$, 126 MHz)

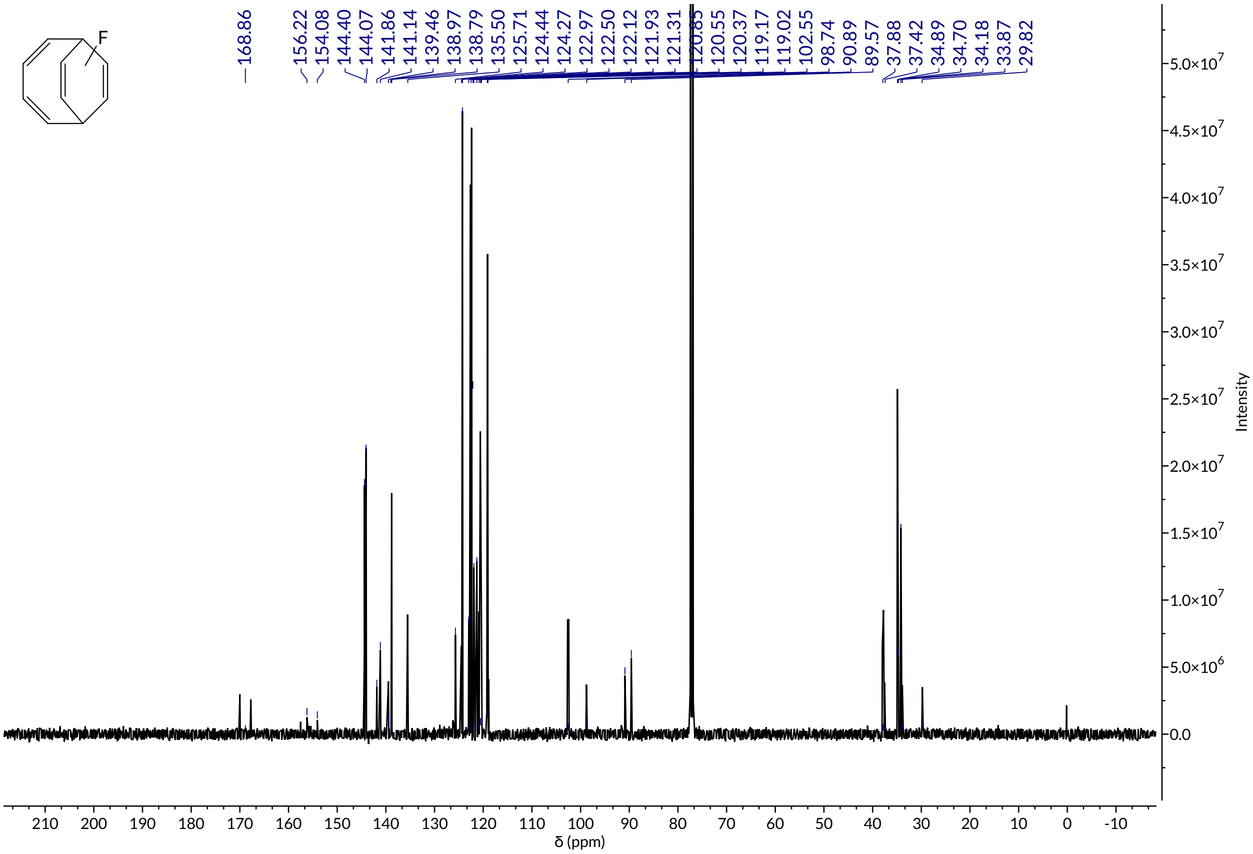

Figure S72: ^{13}C NMR spectrum of **F**-**CA** ($CDCl_3$, 126 MHz)

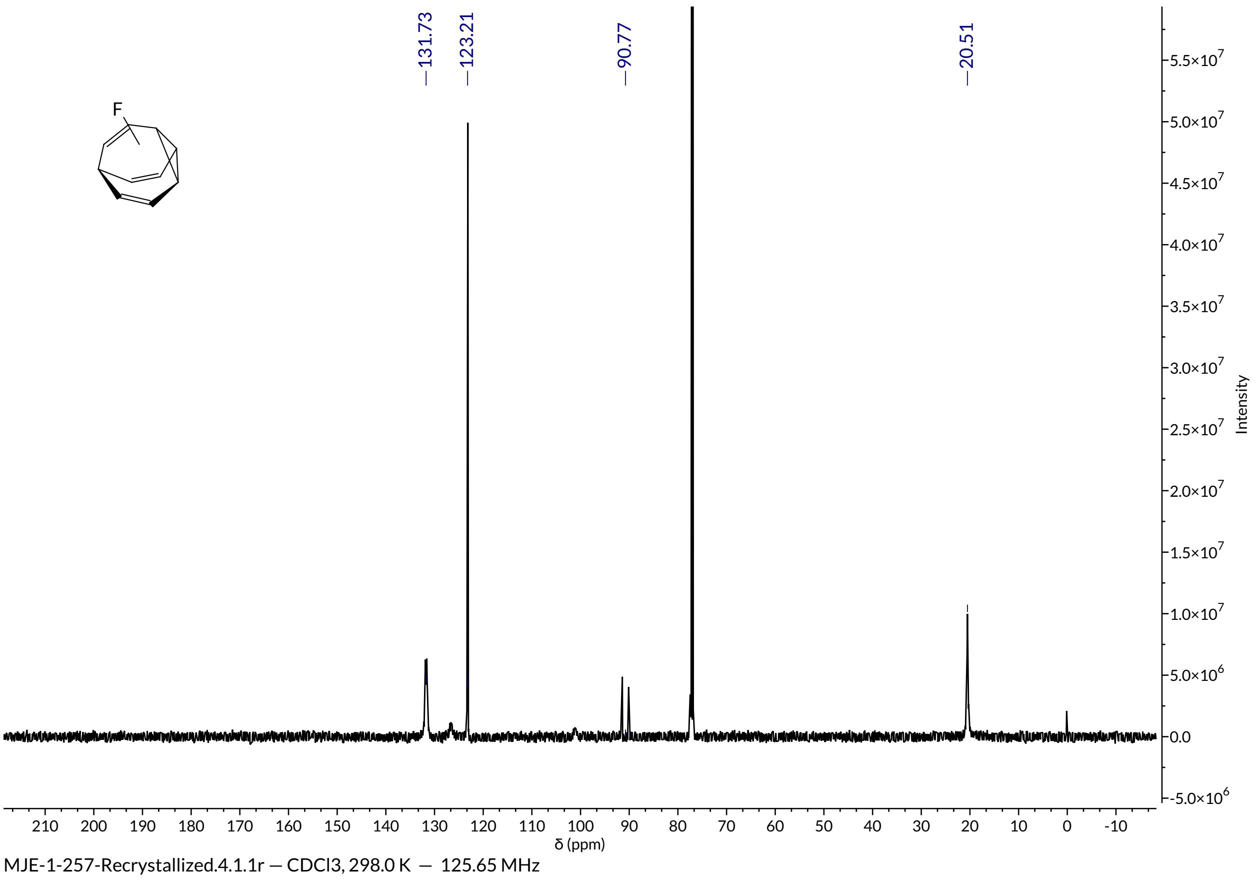

Figure S73: ^{13}C NMR spectrum of **F-Bull** ($CDCl_3$, 126 MHz)

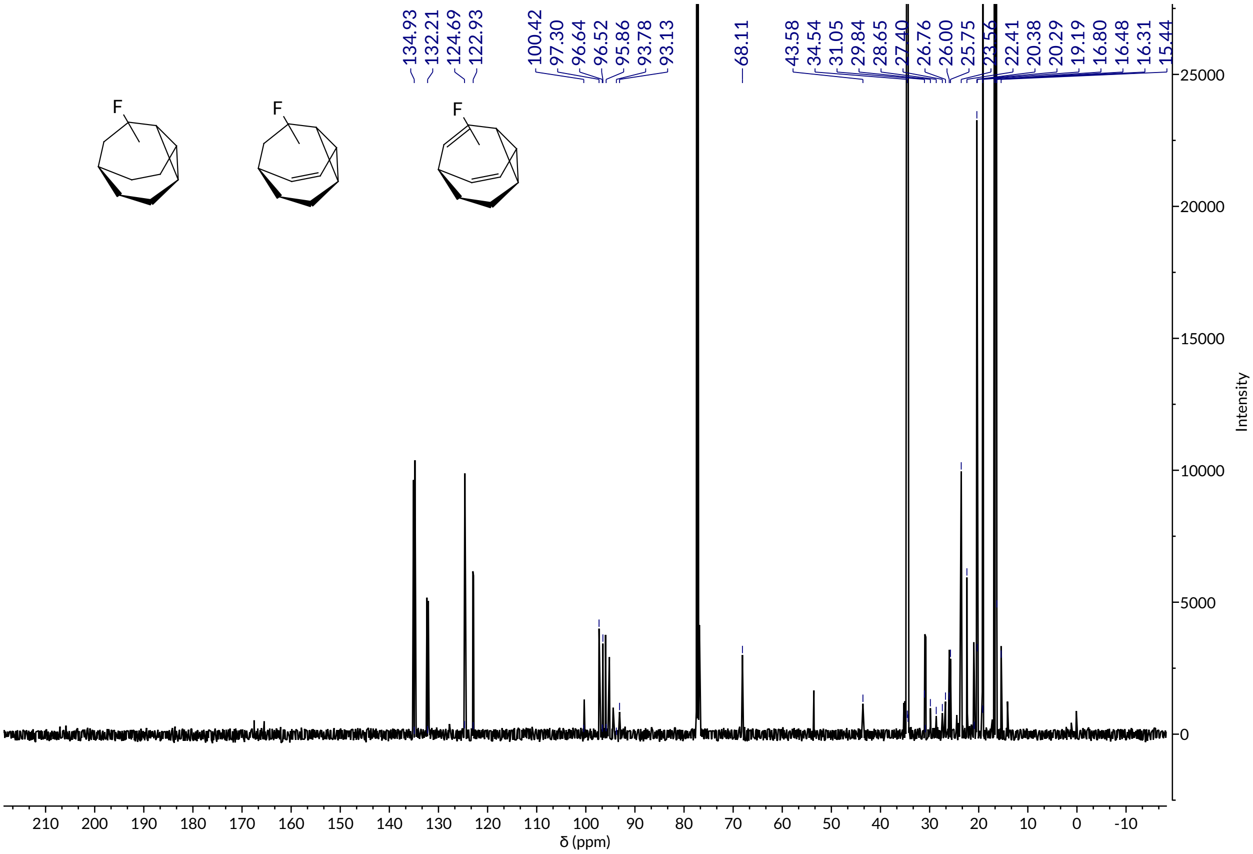

MJE-1-259-SP.4 — CDCl3, 298.0 K — 125.65 MHz

Figure S74: ^{13}C NMR spectrum of **F-Bull-Red^{n}** ($CDCl_3$, 126 MHz)

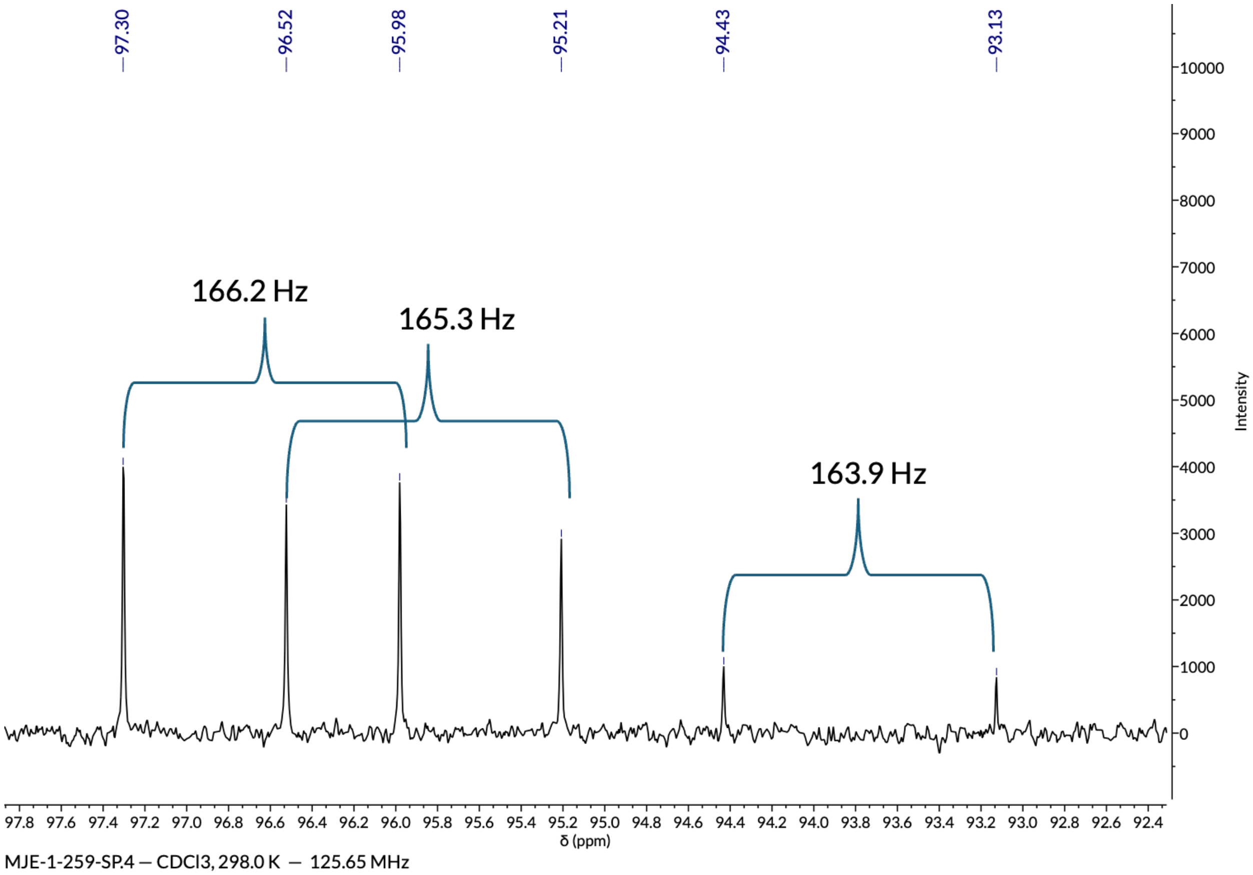

Figure S75: ^{13}C NMR spectrum of **F-Bull-Red^{n}** ($CDCl_3$, 126 MHz) zoomed into the bridgehead region, with 3 distinct isomers showing $^{1}J_{C\text{-}F}$ coupling constants.

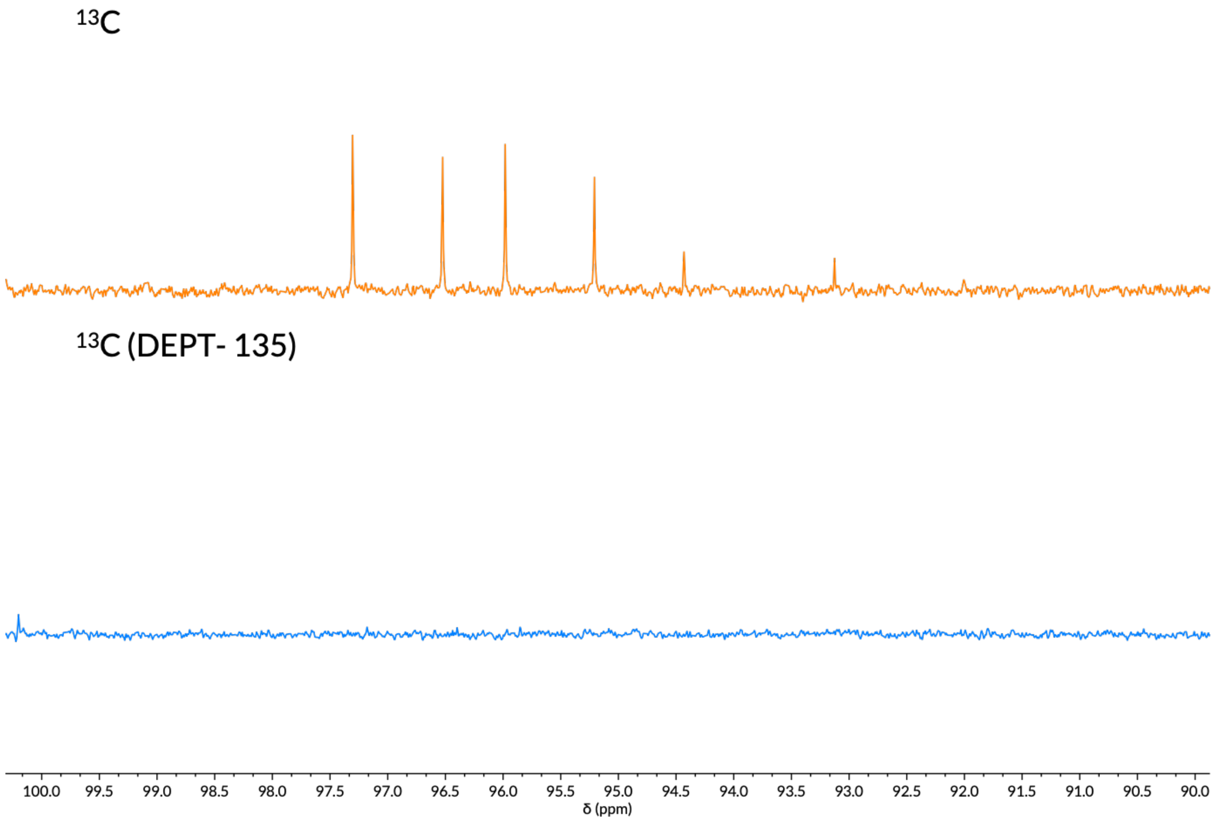

Figure S76: Stacked ^{13}C NMR spectrum (top) and ^{13}C DEPT-135 NMR spectrum (bottom) of **F-Bull-Red^{n}** ($CDCl_3$, 126 MHz) zoomed into the bridgehead region. The bridgehead signals do not show up in the DEPT-135 experiment, confirming they are quarternary and, therefore, ^{19}F-substituted.

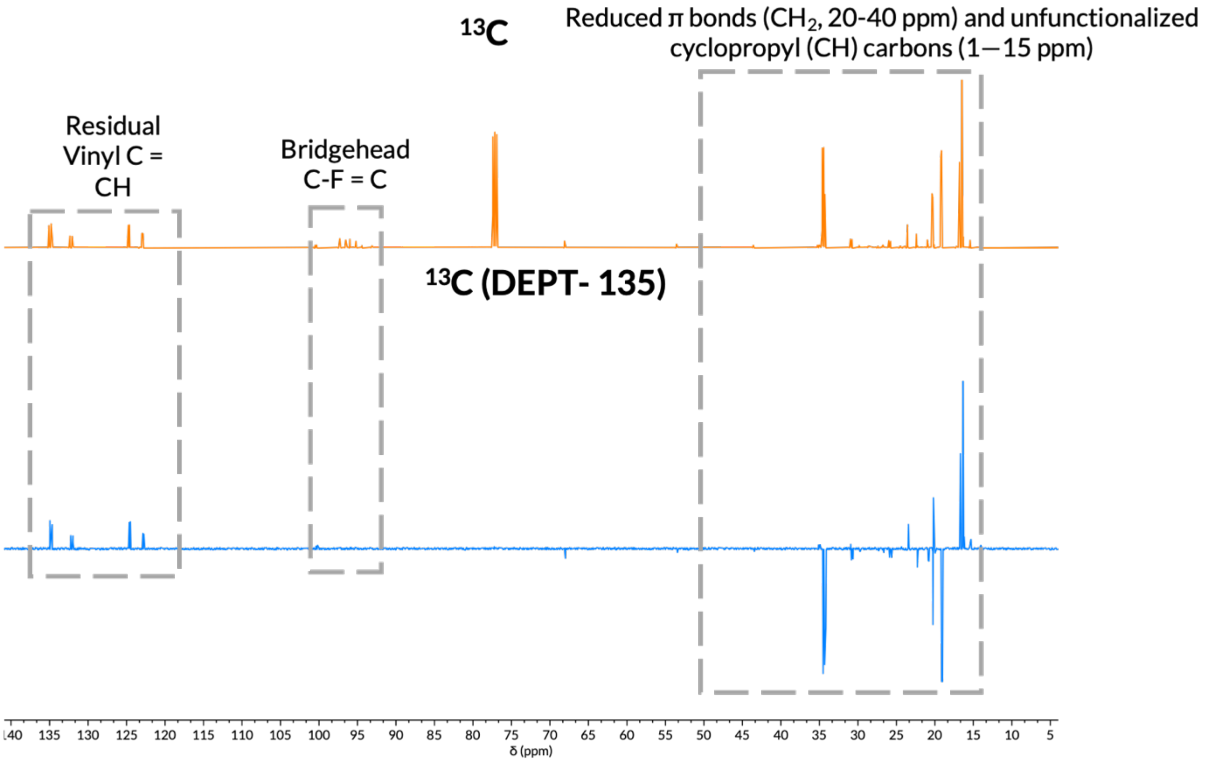

Figure S77: ^{13}C NMR spectrum (top) and ^{13}C DEPT-135 NMR spectrum (bottom) of **F-Bull-Red**^{n} ($CDCl_3$, 126 MHz). The signals are annotated to show how the assignment of the C-F regioisomers were determined.

3.6: ^{19}F EXCHANGE SPECTROSCOPY (EXSY) DATA

Fluorine-19 EXSY NMR spectra were recorded on a Bruker AVANCE-III spectrometer at 500 MHz, equipped with a two-channel BBFO iProbe. Data were collected for samples dissolved in deuterated acetone. Temperature was regulated at 273.0 ± 0.1 degrees, using cold N_2 gas delivered by a Bruker BCU05 chiller. The sample temperature was calibrated using a 4% methanol in deuterated methanol standard solution. A low gas-flow rate of 535 liters per hour (lph) was used to avoid potential spinner vibrations. Samples were equilibrated in the probe for at least 30 minutes prior to data collection. 1D NOESY experiments were performed using the standard Bruker pulse sequence 'selno', using a spectral width of 46,875 Hz (100 ppm), a recycle delay of two seconds, and 1024 scans. 21.2 millisecond (100 Hz bandwidth) Gaussian pulses (waveform truncated at 1%) were used for selective excitation of the targeted multiplets. An exchange delay of 250 milliseconds was used, to favor observation of signals arising from exchange over those that might arise due other transfer mechanisms.

A standard ^{19}F-spectrum was acquired at 273 K (**Figure S78**). Subsequently, separate 1D NOESY experiments were performed (*vide supra*) targeting the multiplets centered at –87.4 ppm (**Figure S79**) and –93.4 ppm (**Figure S80**). The integrals for the peaks in the standard ^{19}F pulse were determined, as were the integrals for the diagonal and cross-peak signals in each 1D NOESY experiment. Finally, the corresponding values were input to the program EXSYCalc by MestreLab[2] to determine the value of the exchange rate constants at 273 K (**Figure S81**).

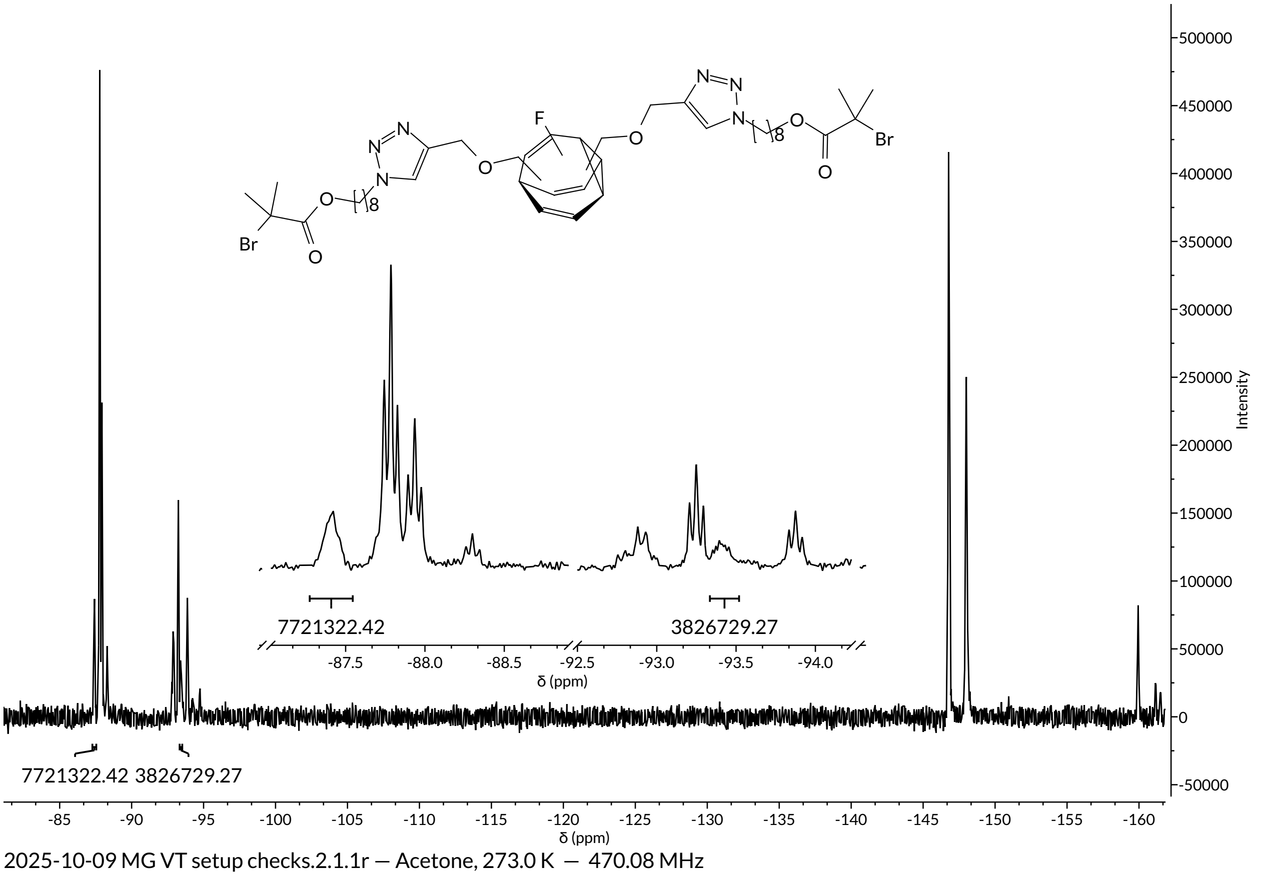

Figure S78: ^{19}F NMR spectrum of **F-Bull-ATRP** (($CO(CD_3)_2$, 470 MHz) at –0.15 °C (273 K). The peaks of interest at –87.4 ppm and –93.4 ppm have been integrated. The inset shows the regions of interest in finer detail.

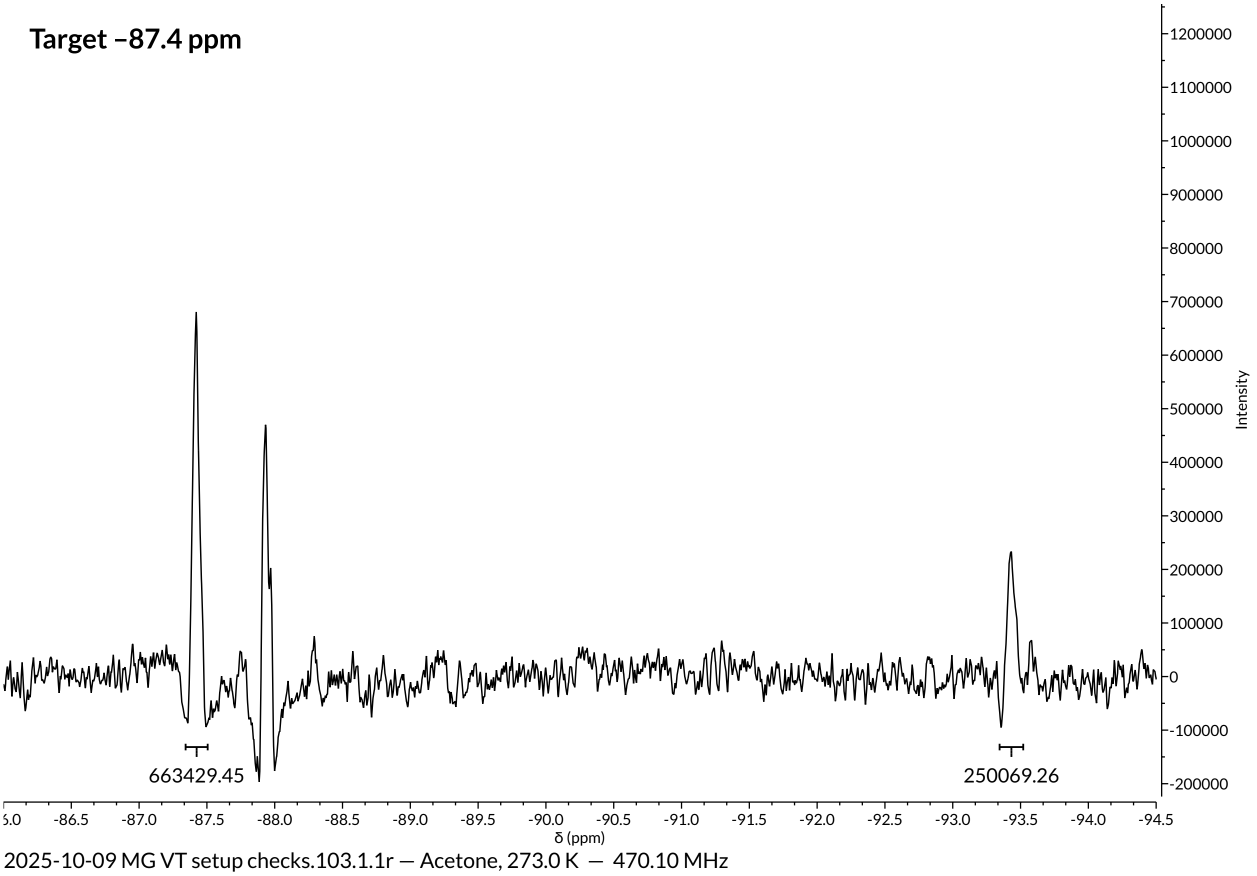

Figure S79: ^{19}F NMR 1D selno of **F-Bull-ATRP** ($(CO(CD_3)_2$, 470 MHz) at –0.15 °C (273 K), irradiating the peak with chemical shift = –87.4 ppm with d8 mixing time of 250 milliseconds. The crosspeak of interest at –93.4 ppm has been integrated.

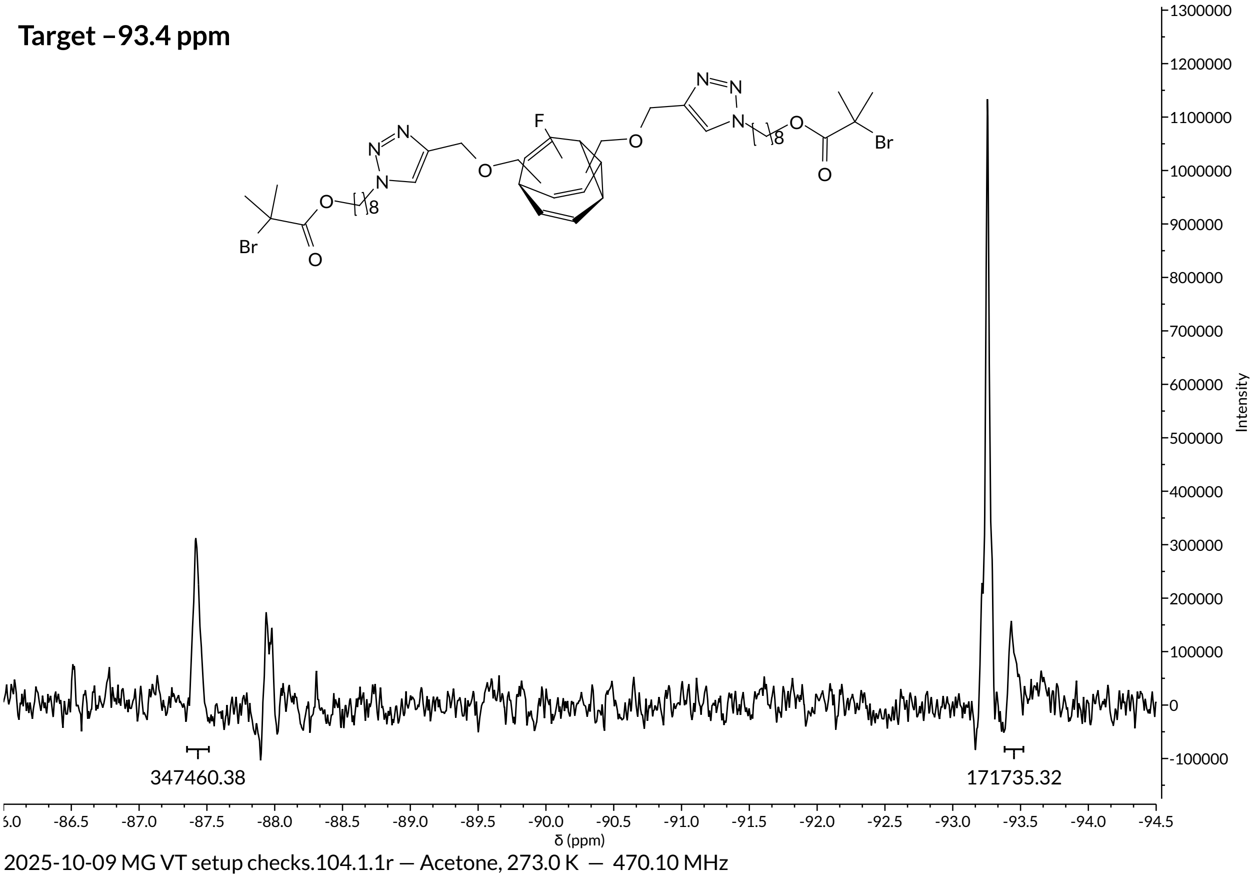

Figure S80: ^{19}F NMR 1D selno of **F-Bull-ATRP** ($(CO(CD_3)_2$, 470 MHz) at –0.15 °C (273 K), irradiating the peak with chemical shift = –93.4 ppm with d8 mixing time of 250 milliseconds. The crosspeak of interest at –87.4 ppm has been integrated.

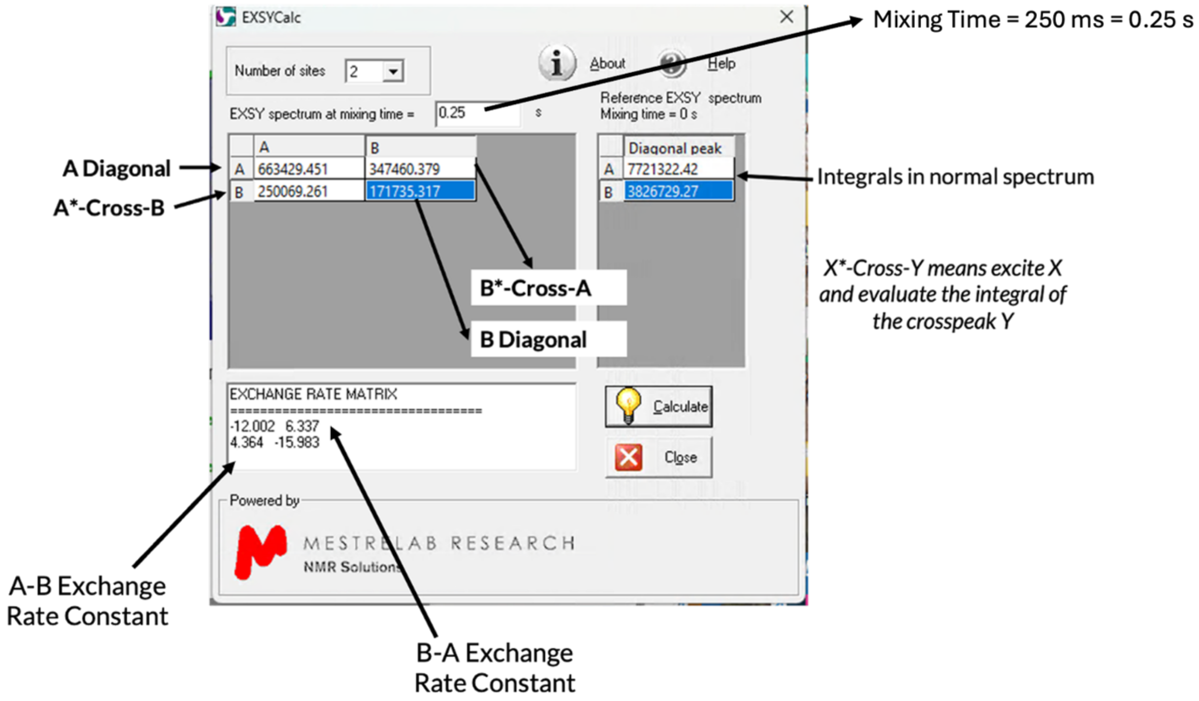

Figure S81: Results of inputting the relevant integrals into the EXSYCalc software program for the evaluation of the exchange rate constants. The rate constants were determined at –0.15 °C (273 K) in deuterated acetone.

Low Temperature Rate Constant Estimation

The rate constant of a reaction at a particular temperature is described by the Arrhenius equation as follows:

$$k_{T_1} = Ae^{-\frac{E_A}{RT_1}} \qquad \textbf{(Equation S2)}$$

Where A is the pre-exponential factor (with units s^{-1} for a unimolecular reaction), E_A is the activation barrier (with units kcal • mol^{-1}), R is the gas constant (and is defined as 1.987 * 10^{-3} kcal • [mol^{-1} • K^{-1}]), and T_1 is the thermodynamic temperature (in K). Therefore, to compare the ratio of the rate constant for a given reaction at two temperatures, T_1 and T_2, we have:

$$\frac{k_{T_1}}{k_{T_2}} = \frac{Ae^{-\frac{E_A}{RT_1}}}{Ae^{-\frac{E_A}{RT_2}}} \qquad \textbf{(Equation S3)}$$

Solving for k_{T_2} and simplifying yields the following expression, which relates the rate constant at any arbitrary temperature T_2 in terms of a known rate constant k_{T_1} at a particular temperature T_1 and the activation barrier E_A:

$$k_{T_2} = k_{T_1}\left(\frac{e^{-\frac{1}{RT_2}}}{e^{-\frac{1}{RT_1}}}\right)^{E_A} \qquad \textbf{(Equation S4)}$$

Using **Equation S4** and the known rate constant measured *via* EXSY at 0 °C ≈ 6.3 s^{-1} (*vide supra*), one can construct a plot relating the expected rate constant at –72 °C (the temperature at which isomer trapping occurred) to the range of activation barriers expected for the many possible isomerizations in our bullvalene network, *i.e.*, 11 kcal/mol < E_A < 20 kcal/mol:

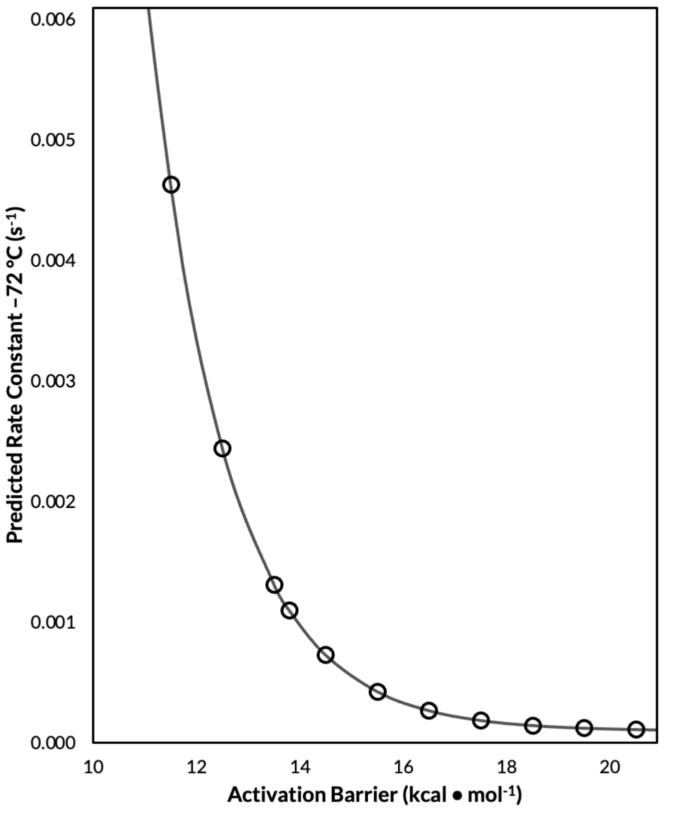

Figure S82: Predicted rate constant for the possible bullvalene isomerization reactions described herein at –72 °C as a function of activation barrier.

Furthermore, for a unimolecular reaction, the half-life $\left(t_{\frac{1}{2}}\right)$ in seconds of a particular species is related to its rate constant *via* the following expression, where:

$$t_{\frac{1}{2}} = \frac{\ln(2)}{k} \qquad \textbf{(Equation S5)}$$

Taking 3 minutes (= 180 seconds) as the amount of time elapsed between the end of sonication and the reaction being quenched (as timed with a stopwatch during experimentation), one can estimate the amount of "out-of-equilibrium" bullvalene isomers remaining (*i.e.,* the percentage of isomers which have not yet undergone isomerization) at the time of quenching *via* **Equation S6**:

$$\%\ Bullvalene\ Isomers\ Remaining = 0.5^{\frac{180}{t_{\frac{1}{2}}}} * 100\% \qquad \textbf{(Equation S6)}$$

It is thus possible to prepare a plot (from **Equation S6**) of the estimated percentage of the force-perturbed isomer distribution (i.e., population has not yet undergone thermal isomerization back to its equilibrium states) relative to the activation barrier for the various isomerization reactions:

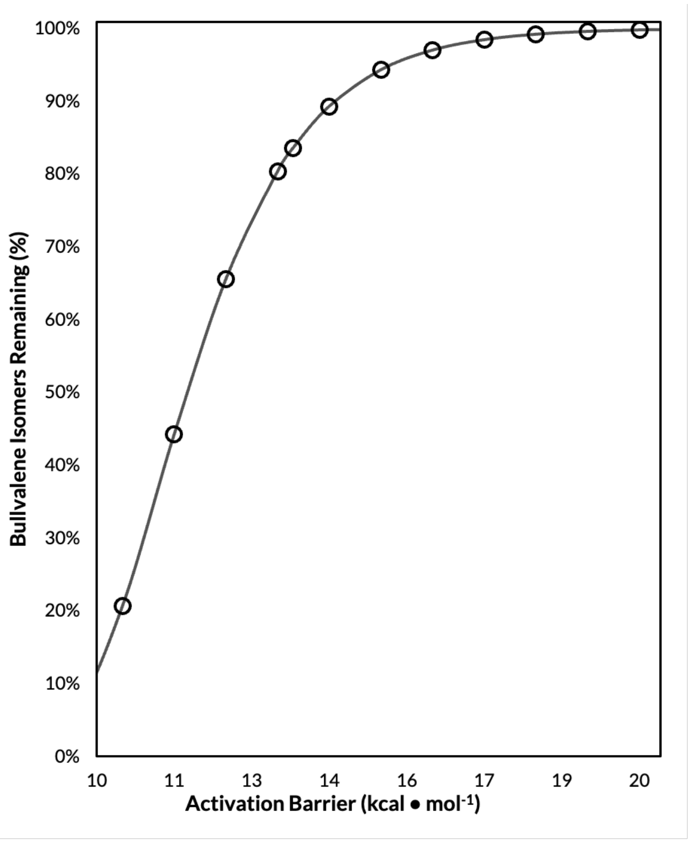

Figure S83: Predicted percentage of "out of equilibrium" bullvalene isomers remaining at the time of quenching, for the possible bullvalene isomerization reactions described herein at –72 °C, as a function of the activation barrier

Thus, for a bullvalene isomerization network with activation barriers in the 11-17 kcal/mol range (see **Table S5** for calculated E_A values), we anticipate ca. 50-95% of the perturbed bullvalene isomer distribution to remain at the time of quenching, assuming similar values for the pre-exponential factor across isomerizations. Literature precedent indicates that the ßgγ transition for fluorobullvalene is the fastest of the possible rearrangement pathways due to a substantially larger pre-exponential factor than the other transitions[7]. Collectively, we expect that this approach delivers a very conservative estimate of the upper bound for rate constants in our system.

3.7: GAS CHROMATAGRAMS AND MASS SPECTRA

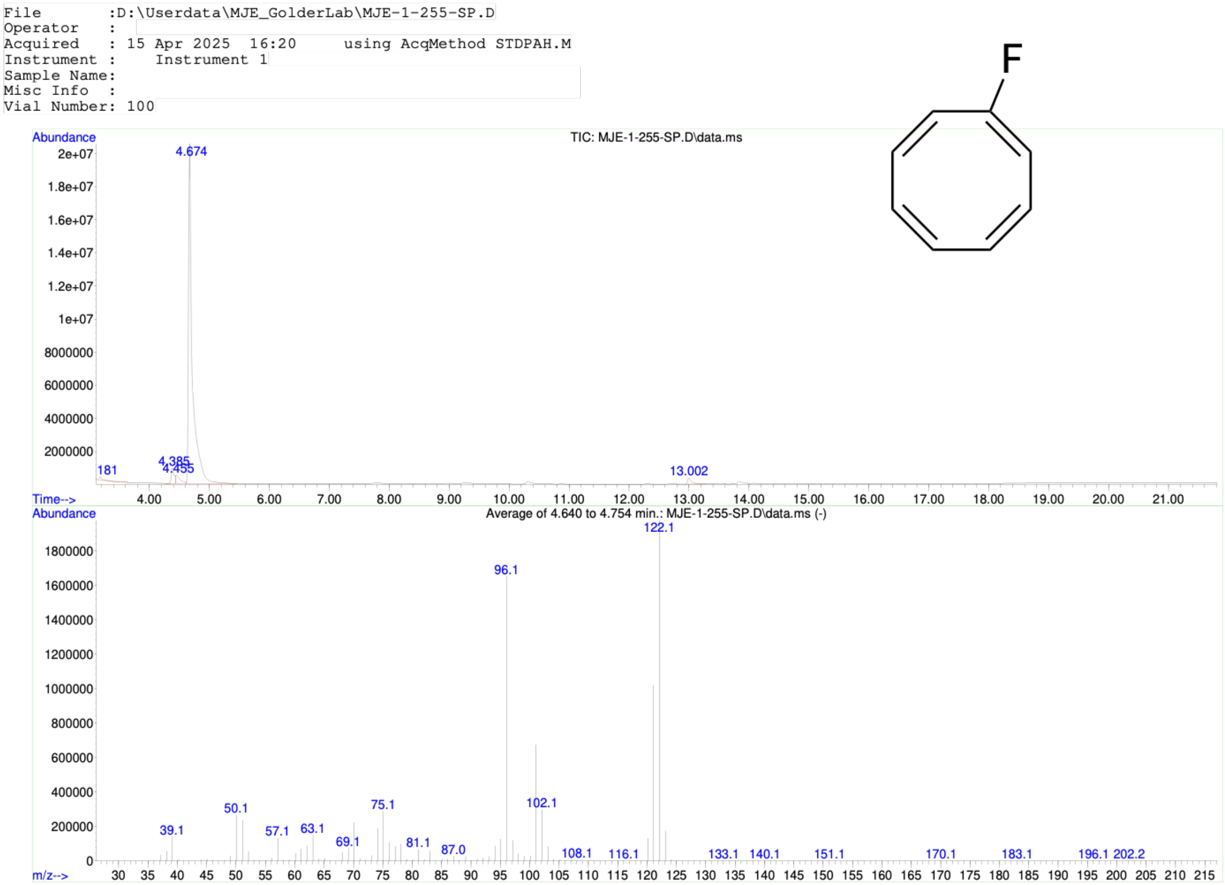

Figure S84: TOP: Gas chromatogram for **F-COT**. BOTTOM: Mass spectrum (EI) for the peak with retention time = 4.674 minutes. Calculated for C_8H_7F [$M^{\bullet+}$]: 122.1; found: 122.1

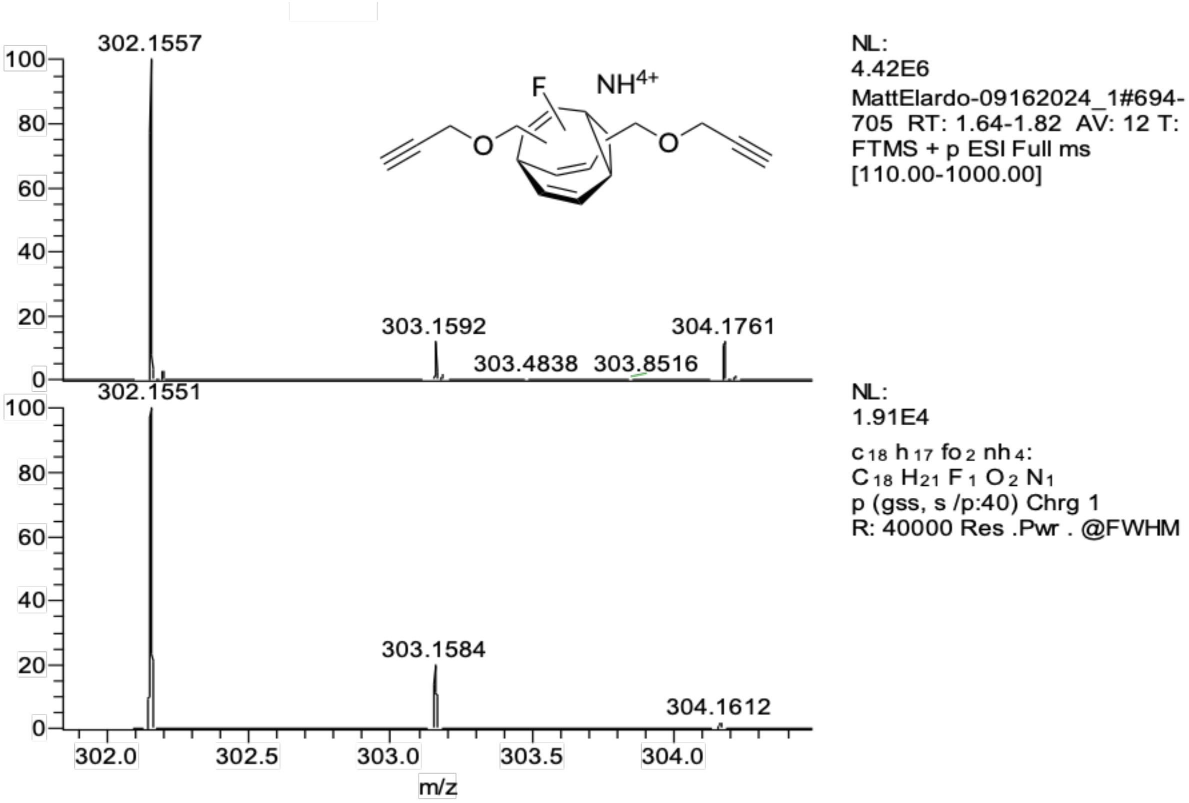

Figure S85: TOP: Obtained HRMS (ESI) spectrum for **F-Bull-PE** ammonium adduct. BOTTOM: Computed spectrum for $C_{18}H_{21}O_2NF$. Calculated for $C_{18}H_{18}FO_2N$ [M•NH_4^+]: 302.1551; found: 302.1557 The sample was prepared as a solution in methanol with added ammonium hydroxide. Methanol was used as the mobile phase.

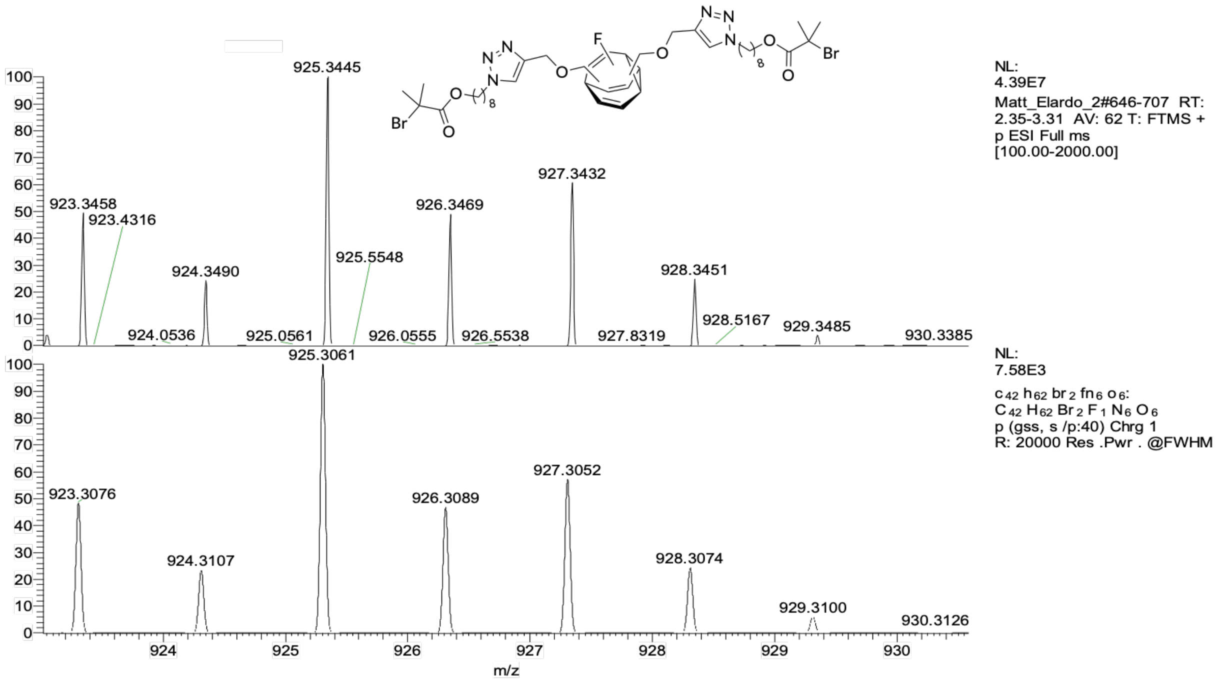

Figure S86: TOP: Obtained HRMS (ESI) spectrum for **F-Bull-ATRP**. BOTTOM: Computed spectrum for $C_{42}H_{62}Br_2N_6O_6F$. Calculated for $C_{42}H_{62}Br_2FN_6O_6N$ [M•H+]: 925.3061; found: 925.3046. The solution was prepared as a solution in methanol and MeCN/H_2O (+0.1% formic acid) was used as the mobile phase.

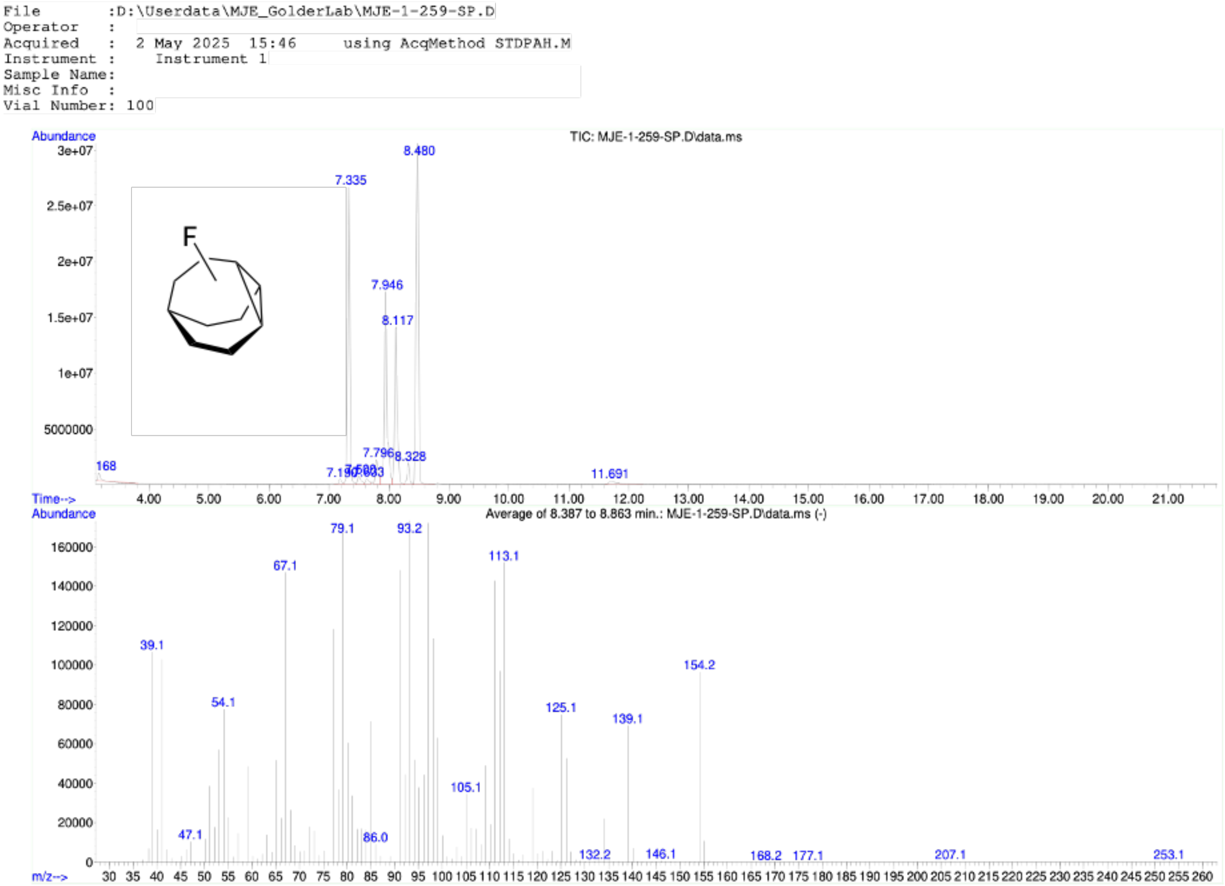

Figure S87: TOP: Gas chromatogram for **F-Bull-Red^{n}**. BOTTOM: Mass spectrum (EI) for the peak with retention time = 8.480 minutes (**F-Bull-Red^{3}**). Calculated for $C_{10}H_{15}F$ [$M^{\bullet +}$]: 154.2; found: 154.2

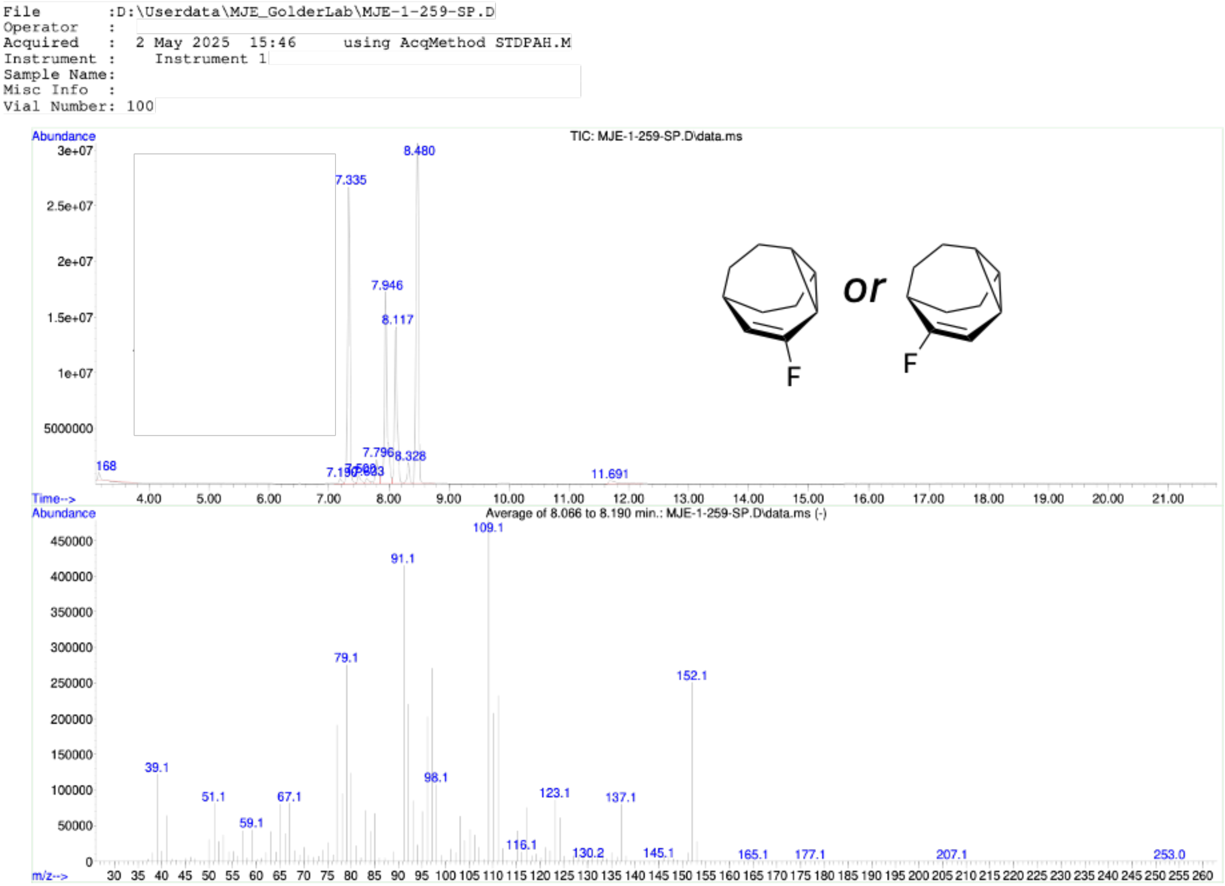

Figure S88: TOP: Gas chromatogram for **F-Bull-Red^{n}**. BOTTOM: Mass spectrum (EI) for the peak with retention time = 8.117 minutes (**F-Bull-Red^{2}**). Calculated for $C_{10}H_{15}F$ $[M^{\bullet+}]$: 152.2; found: 152.1

```
File        :D:\Userdata\MJE_GolderLab\MJE-1-259-SP.D
Operator    :
Acquired    :  2 May 2025  15:46      using AcqMethod STDPAH.M
Instrument  :    Instrument 1
Sample Name:
Misc Info   :
Vial Number: 100
```

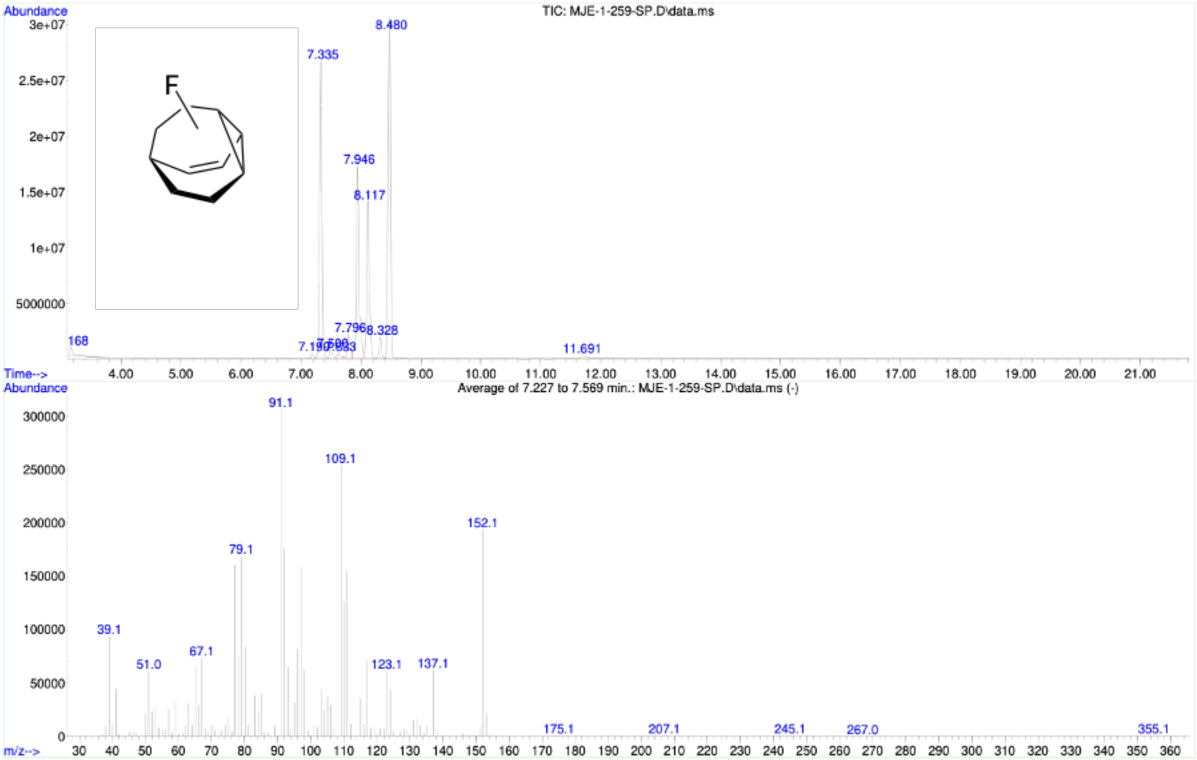

Figure S89: TOP: Gas chromatogram for **F-Bull-Red**^{n}. BOTTOM: Mass spectrum (EI) for the peak with retention time = 7.335 minutes (**F-Bull-Red**^{2}). Calculated for $C_{10}H_{15}F$ [$M^{\bullet +}$]: 152.2; found: 152.1

```
File        :D:\Userdata\MJE_GolderLab\MJE-1-259-SP.D
Operator    :
Acquired    :  2 May 2025  15:46     using AcqMethod STDPAH.M
Instrument :    Instrument 1
Sample Name:
Misc Info  :
Vial Number: 100
```

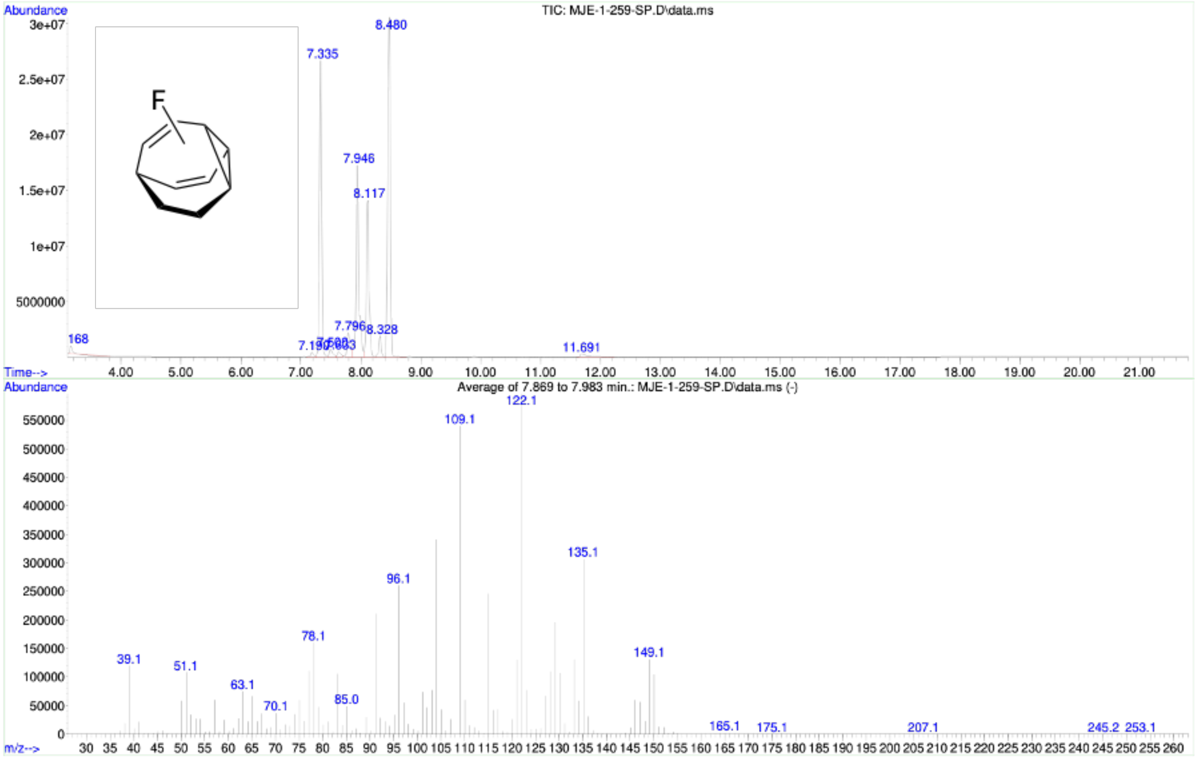

Figure S90: TOP: Gas chromatogram for **F-Bull-Red**[n]. BOTTOM: Mass spectrum (EI) for the peak with retention time = 7.946 minutes (**F-Bull-Red**[1]). Calculated for $C_{10}H_{15}F$ [$M^{\bullet+}$]: 150.2; found: 149.1

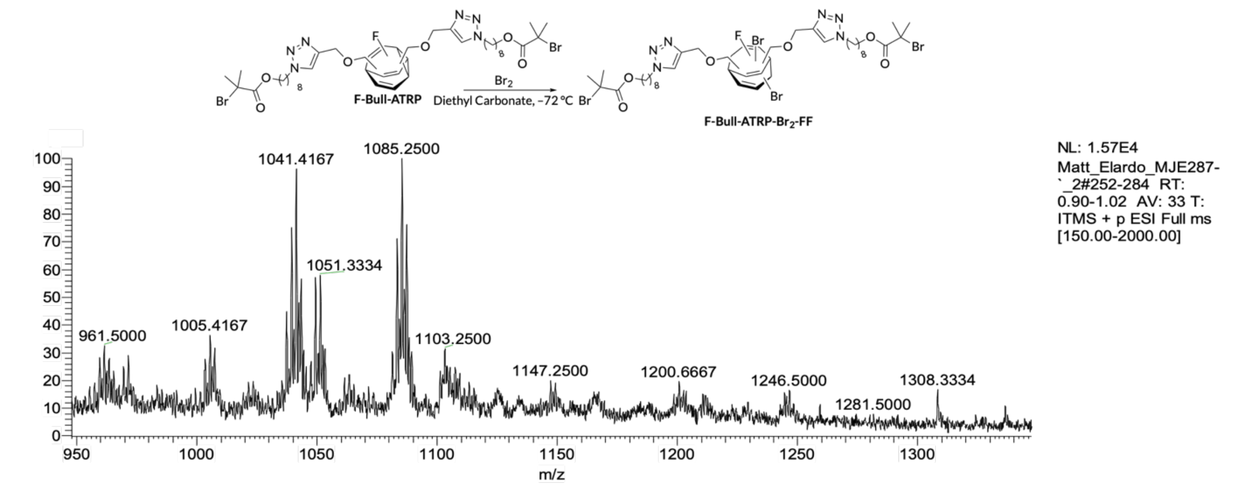

Figure S91: Full HRMS (ESI) spectrum for **F-Bull-ATRP-Br$_2$-FF**. The solution was prepared as a solution in methanol and MeCN/H_2O (+0.1% formic acid) was used as the mobile phase. The expected product peak is present at m/z = 1085 (Zoom-in depicted in **Figure S92**). Other signals at m/z = 1041 (**Figure S93**) and 1051 (**Figure S94**) are arising from halogen exchange (Br→Cl), presumably during ionization[13], and reactivity with trace ethanol in the diethyl carbonate solution used during bromination.

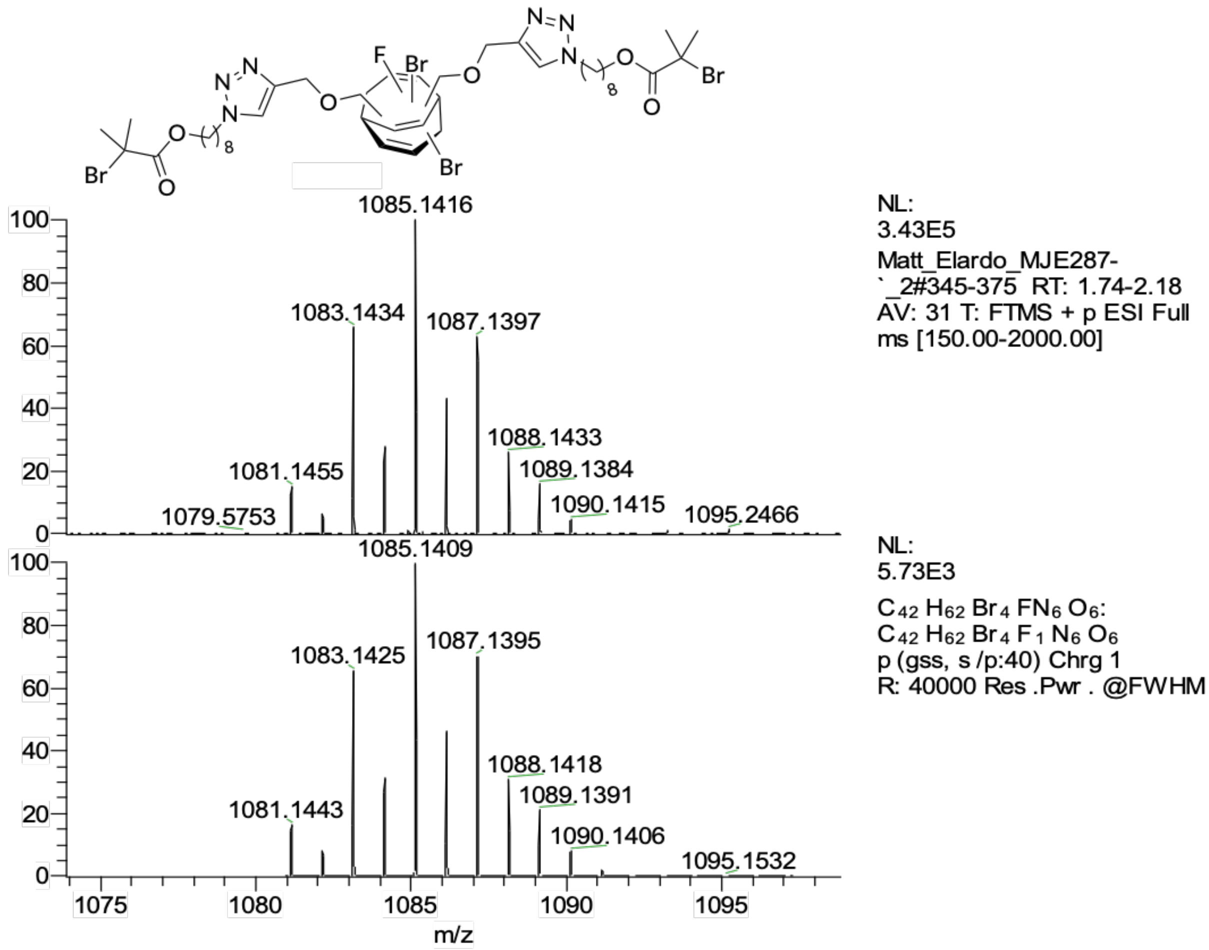

Figure S92: TOP: Obtained HRMS (ESI) spectrum for **F-Bull-ATRP-Br$_2$-FF**. BOTTOM: Computed spectrum for $C_{42}H_{62}Br_4FN_6O_6$. Calculated for $C_{42}H_{62}Br_4FN_6O_6$ [M•H$^+$]: 1,085.1409; found: 1,085.1416. The solution was prepared as a solution in methanol and MeCN/H_2O (+0.1% formic acid) was used as the mobile phase.

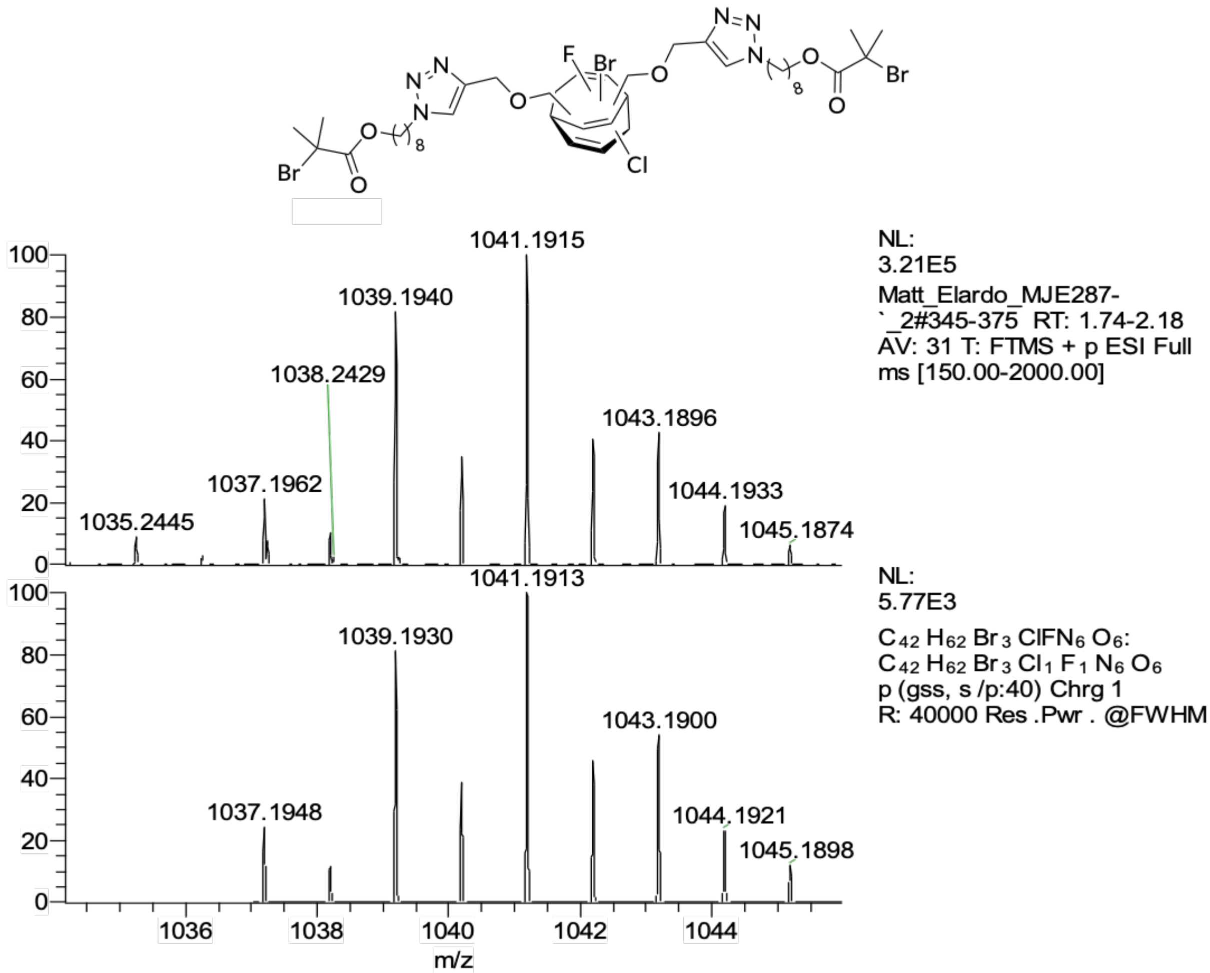

Figure S93: TOP: Obtained HRMS (ESI) spectrum for **F-Bull-ATRP-Br$_2$-FF**[–Br, +Cl]. BOTTOM: Computed spectrum for $C_{42}H_{62}Br_3ClFN_6O_6$. Calculated for $C_{42}H_{62}Br_3ClFN_6O_6$ [M•H+, –Br +Cl]: 1,041.1913; found: 1,041.1915. The solution was prepared as a solution in methanol and MeCN/H_2O (+0.1% formic acid) was used as the mobile phase.

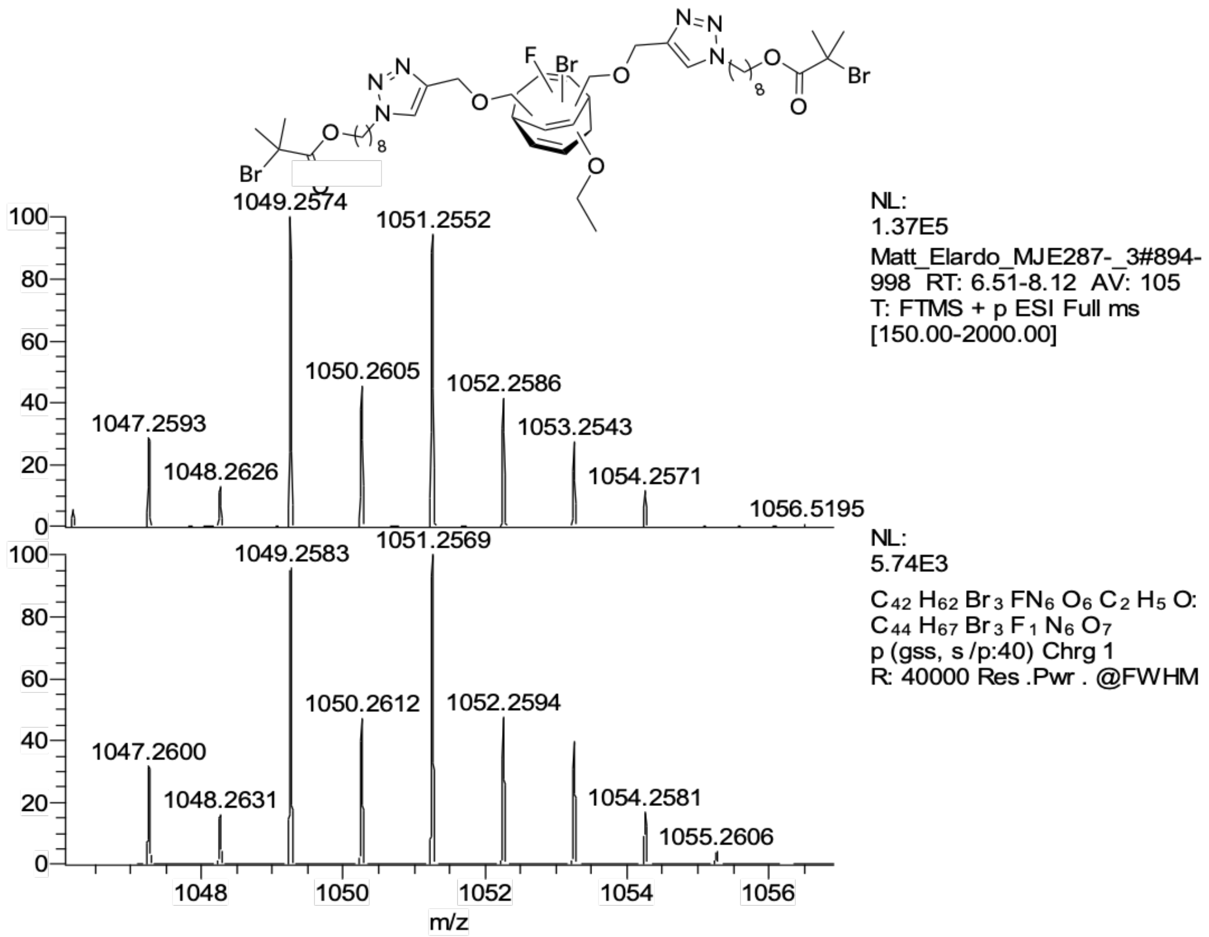

Figure S94: TOP: Obtained HRMS (ESI) spectrum for **F-Bull-ATRP-Br$_2$-FF**[–Br, +OEt]. BOTTOM: Computed spectrum for $C_{44}H_{67}Br_3FN_6O_7$. Calculated for $C_{44}H_{67}Br_3FN_6O_7$ [M•H+, –Br +OEt]: 1,051.2569; found: 1,051.2552. The solution was prepared as a solution in methanol and MeCN/H_2O (+0.1% formic acid) was used as the mobile phase.

3.8: GPC CHROMATAGRAMS

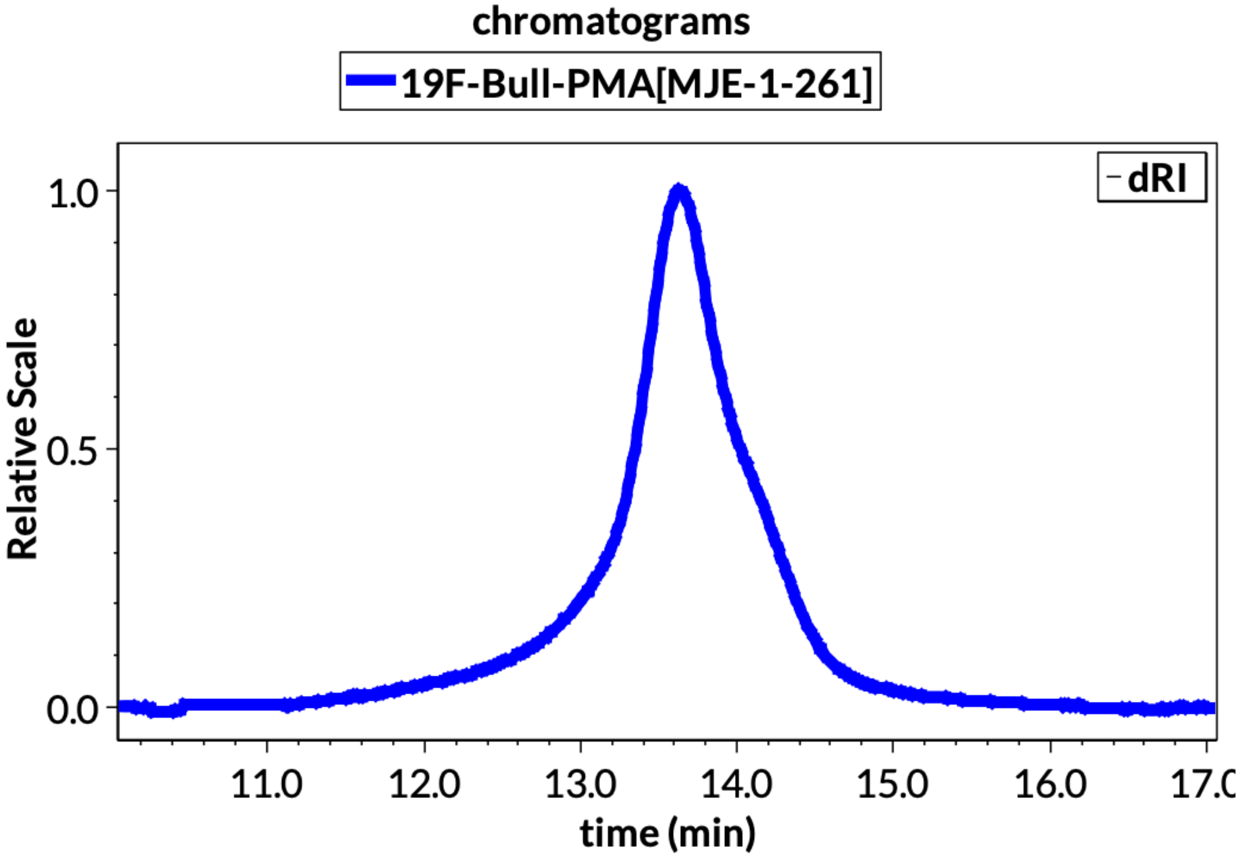

Figure S95: Differential Refractive index trace of **F-Bull-PMA** with M_n = 77 kDa, M_w = 91 kDa, Đ = 1.3, *dn/dc* (100% mass recovery assumed) = 0.0381. *This polymer sample was used for all sonication–bromination reactions and all relevant controls.*

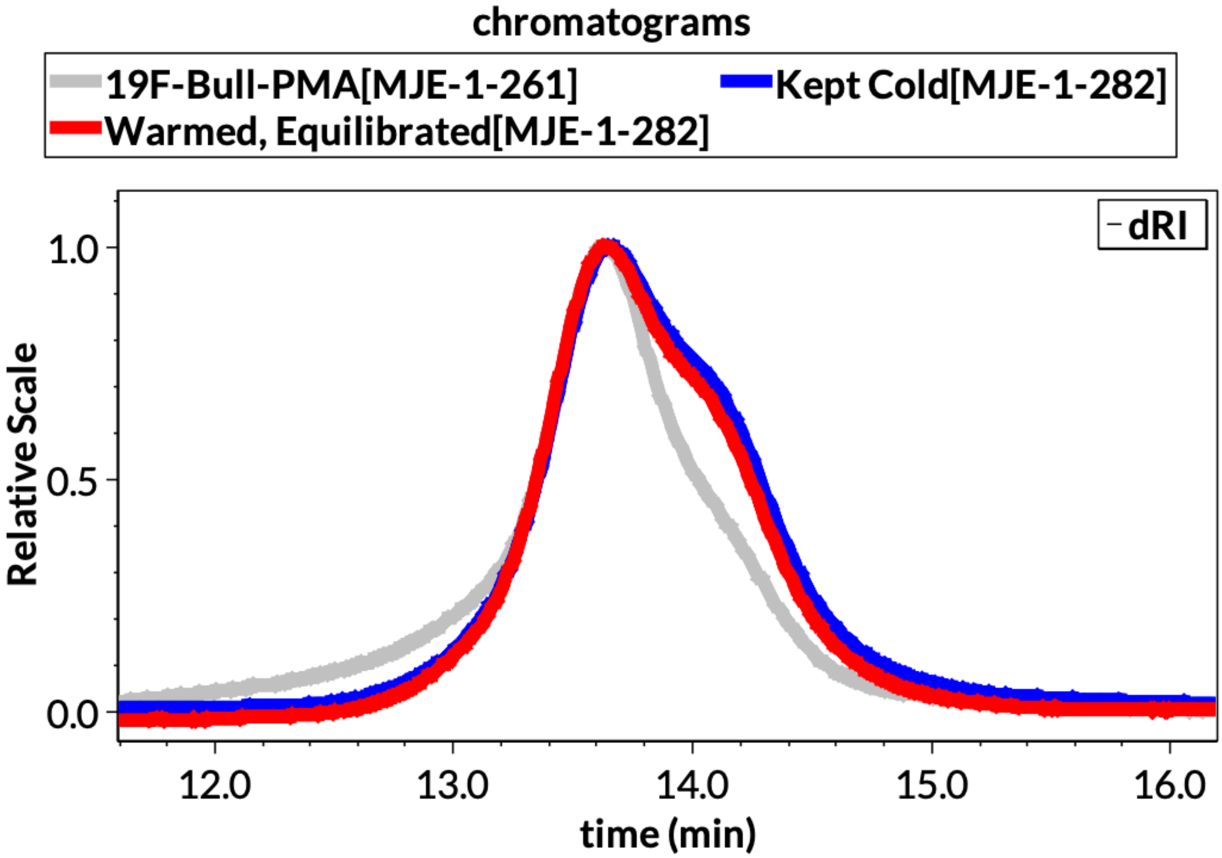

Figure S96: Differential Refractive index traces of: **F-Bull-PMA** with M_n = 77 kDa, M_w = 91 kDa, Đ = 1.2 (silver trace); Representative differential refractive index trace of **F-Bull-PMA-Br$_2$-Cold** with M_n = 57 kDa, M_w = 64 kDa, Đ = 1.1 (blue trace); and representative differential refractive index trace of **F-Bull-PMA-Br$_2$-Warmed** with M_n = 60 kDa, M_w = 68 kDa, Đ = 1.1 (red trace). A *dn/dc* = 0.0381 (100% mass recovery; obtained from original polymer) was used for all samples.

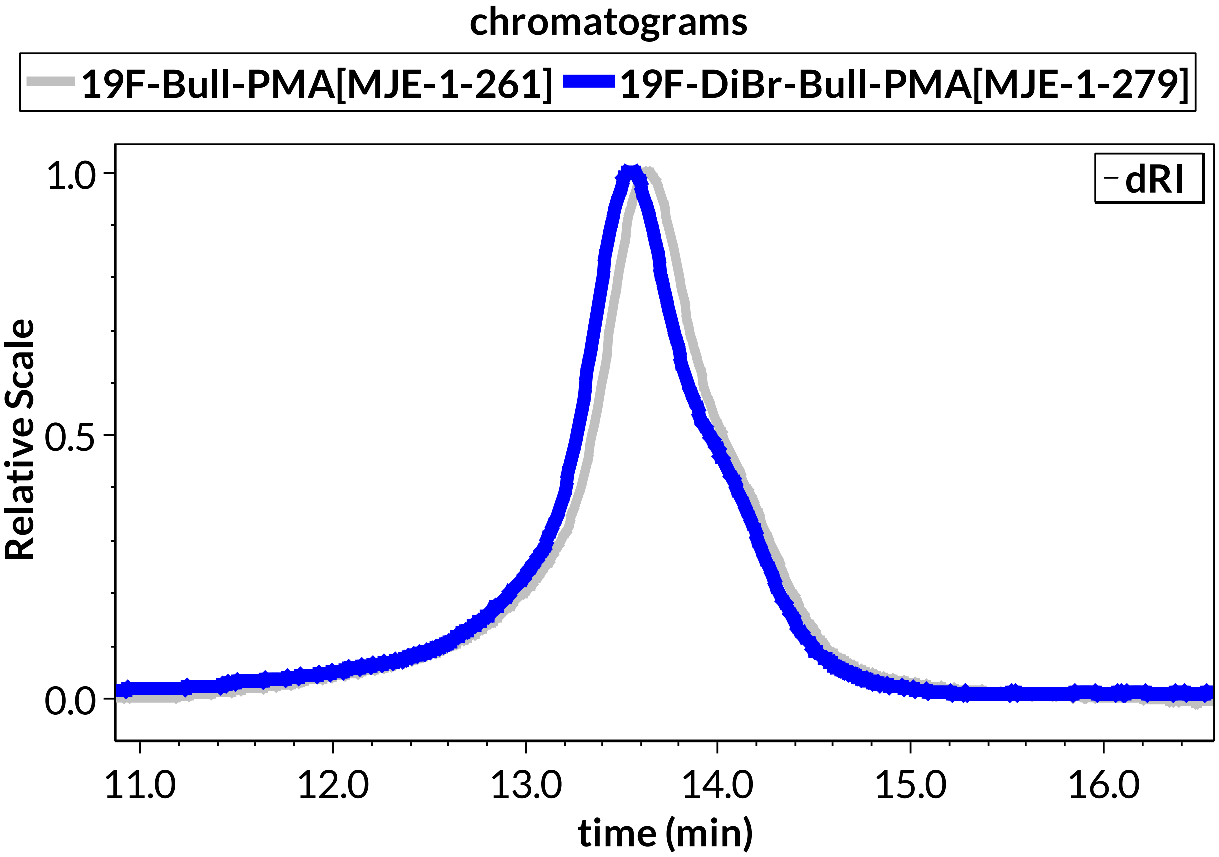

Figure S97: Differential Refractive index traces of: **F-Bull-PMA** with M_n = 77 kDa, M_w = 91 kDa, Đ = 1.2 (silver trace) and **F-Bull-PMA-Br$_2$-FF** with M_n = 88 kDa, M_w = 112 kDa, Đ = 1.3 (blue trace). A *dn/dc* = 0.0381 (100% mass recovery; obtained from original polymer) was used for all samples.

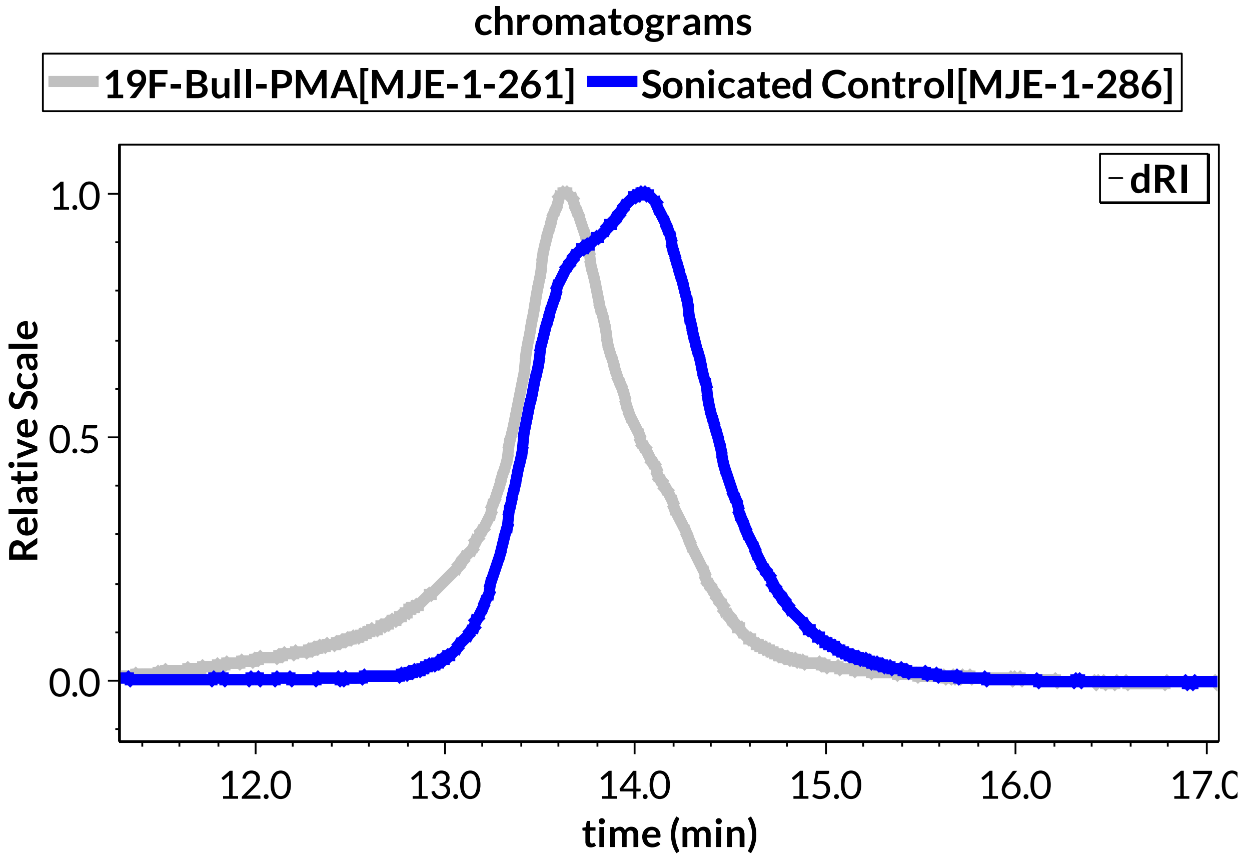

Figure S98: Differential Refractive index traces of: **F-Bull-PMA** with M_n = 77 kDa, M_w = 91 kDa, Đ = 1.2 (silver trace) and **F-Bull-PMA-Sonics-Control** with M_n = 48 kDa, M_w = 53 kDa, Đ = 1.1 (blue trace). A *dn*/*dc* = 0.0381 (100% mass recovery; obtained from original polymer) was used for all samples.

4.0: ISOMER DISTRIBUTION STATISTICAL TESTING RESULTS

Comment on Integration Methodology:

The relative abundance of each isomer in the –90 to –100 ppm region was determined by integrating each peak in this region and normalizing the total integration to 100. Integration values are reported as an average from 3 separate sonication experiements ± one standard deviation; significance testing was perfomed by computing p-values from two-tailed independent samples t-tests. Signficant values (*, **, ***) were reported for p-values ≤0.05, 0.01, or 0.001, respectively. We elected to use this method of integration for the following reasons:

1) Due to inhomogeneities in the amount of sample, and the sample molecular weight following sonication, determination of the molarity of each NMR sample was nontrivial. This precluded analysis using internal standard, as the absolute concentration of each isomer was not constant between experiments, and normalizing based on the molarity of each sample was not possible due to error in determing the actual molar mass of each polymer sample following ultrasonication. Therefore, even with added internal standard, the integrated peaks must be normalized to relative abundance within each experiment in order to compare between replicates; this process is identical to the process described herein, except with the additional step of adding and integrating nanomolar amounts of internal standard, which would compound errors in integration.
2) Alternatively, one could chose one peak in the –90 to –100 ppm range on which to normalize the rest of the integrations, then compare the relative abundance of each isomer relative to the normalized peak. However, this approach requires arbitratily picking a single isomer peak and assuming that particular isomer remains constant in terms of relative abundance following ultrasonication. This assumption therefore distorts the analyses in the event that the selected isomer does not remain constant in terms of relative abundance, preventing accurate analysis.

Group Statistics

	Treatment	N	Mean	Std. Deviation	Std. Error Mean
Integral	Cold	3	15.2333	2.35867	1.36178
	Warm	3	19.6667	2.11975	1.22384

Independent Samples Test

		Levene's Test for Equality of Variances		t-test for Equality of Means							
						Significance				95% Confidence Interval of the Difference	
		F	Sig.	t	df	One-Sided p	Two-Sided p	Mean Difference	Std. Error Difference	Lower	Upper
Integral	Equal variances assumed	.021	.893	-2.421	4	.036	.073	-4.43333	1.83091	-9.51675	.65008
	Equal variances not assumed			-2.421	3.955	.037	.073	-4.43333	1.83091	-9.53952	.67286

Independent Samples Effect Sizes

		Standardizer[a]	Point Estimate	95% Confidence Interval Lower	Upper
Integral	Cohen's d	2.24239	-1.977	-3.987	.163
	Hedges' correction	2.81042	-1.577	-3.181	.130
	Glass's delta	2.11975	-2.091	-4.505	.447

a. The denominator used in estimating the effect sizes.
Cohen's d uses the pooled standard deviation.
Hedges' correction uses the pooled standard deviation, plus a correction factor.
Glass's delta uses the sample standard deviation of the control (i.e., the second) group.

Figure S99: Results of a t-test comparing the relative abundanve of isomer –91.8 ppm between **F-Bull-PMA-Br$_2$-Cold** and **F-Bull-PMA-Br$_2$-Warmed** (p = 0.073)

Group Statistics

	Treatment	N	Mean	Std. Deviation	Std. Error Mean
I922	Warm	3	17.2333	.68069	.39299
	Cold	3	22.2667	1.66533	.96148

Independent Samples Test

		t-test for Equality of Means							
				Significance				95% Confidence Interval of the Difference	
		t	df	One-Sided p	Two-Sided p	Mean Difference	Std. Error Difference	Lower	Upper
I922	Equal variances assumed	-4.846	4	.004	.008	-5.03333	1.03870	-7.91722	-2.14945
	Equal variances not assumed	-4.846	2.650	.011	.022	-5.03333	1.03870	-8.60007	-1.46660

Homogeneity of Variance Test

		Levene's Test for Equality of Variances F	Sig.
I922	Equal variances assumed	3.092	.154

Independent Samples Effect Sizes

		Standardizer[a]	Point Estimate	95% Confidence Interval Lower	Upper
I922	Cohen's d	1.27214	-3.957	-6.994	-.836
	Hedges' correction	1.59439	-3.157	-5.580	-.667
	Glass's delta	1.66533	-3.022	-6.157	.141

a. The denominator used in estimating the effect sizes.
Cohen's d uses the pooled standard deviation.
Hedges' correction uses the pooled standard deviation, plus a correction factor.
Glass's delta uses the sample standard deviation of the control (i.e., the second) group.

Figure S100: Results of a t-test comparing the relative abundanve of isomer –92.8 ppm between **F-Bull-PMA-Br$_2$-Cold** and **F-Bull-PMA-Br$_2$-Warmed** (p = 0.008)

Group Statistics

	Treatment	N	Mean	Std. Deviation	Std. Error Mean
Integral	Cold	3	4.8667	1.00664	.58119
	Warm	3	7.1667	.35119	.20276

Independent Samples Test

		Levene's Test for Equality of Variances		t-test for Equality of Means							
						Significance		Mean Difference	Std. Error Difference	95% Confidence Interval of the Difference	
		F	Sig.	t	df	One-Sided p	Two-Sided p			Lower	Upper
Integral	Equal variances assumed	2.264	.207	-3.737	4	.010	.020	-2.30000	.61554	-4.00901	-.59099
	Equal variances not assumed			-3.737	2.480	.023	.046	-2.30000	.61554	-4.51326	-.08674

Independent Samples Effect Sizes

		Standardizer[a]	Point Estimate	95% Confidence Interval Lower	Upper
Integral	Cohen's d	.75388	-3.051	-5.581	-.408
	Hedges' correction	.94485	-2.434	-4.453	-.325
	Glass's delta	.35119	-6.549	-12.746	-.715

a. The denominator used in estimating the effect sizes.
Cohen's d uses the pooled standard deviation.
Hedges' correction uses the pooled standard deviation, plus a correction factor.
Glass's delta uses the sample standard deviation of the control (i.e., the second) group.

Figure S101: Results of a t-test comparing the relative abundanve of isomer –92.4 ppm between **F-Bull-PMA-Br_2-Cold** and **F-Bull-PMA-Br_2-Warmed** (p = 0.020)

Group Statistics

	Treatment	N	Mean	Std. Deviation	Std. Error Mean
Integral	Cold	3	12.5333	2.47857	1.43101
	Warm	3	11.4667	1.85023	1.06823

Independent Samples Test

		Levene's Test for Equality of Variances		t-test for Equality of Means							
						Significance		Mean Difference	Std. Error Difference	95% Confidence Interval of the Difference	
		F	Sig.	t	df	One-Sided p	Two-Sided p			Lower	Upper
Integral	Equal variances assumed	.659	.463	.597	4	.291	.582	1.06667	1.78575	-3.89136	6.02469
	Equal variances not assumed			.597	3.701	.292	.585	1.06667	1.78575	-4.05350	6.18683

Independent Samples Effect Sizes

		Standardizer[a]	Point Estimate	95% Confidence Interval Lower	Upper
Integral	Cohen's d	2.18708	.488	-1.173	2.093
	Hedges' correction	2.74110	.389	-.936	1.670
	Glass's delta	1.85023	.577	-1.165	2.203

a. The denominator used in estimating the effect sizes.
Cohen's d uses the pooled standard deviation.
Hedges' correction uses the pooled standard deviation, plus a correction factor.
Glass's delta uses the sample standard deviation of the control (i.e., the second) group.

Figure S102: Results of a t-test comparing the relative abundanve of isomer –92.7 ppm between **F-Bull-PMA-Br_2-Cold** and **F-Bull-PMA-Br_2-Warmed** (p = 0.582)

Group Statistics

	Treatment	N	Mean	Std. Deviation	Std. Error Mean
Integral	Cold	3	11.6333	1.02632	.59255
	Warm	3	14.8333	.97125	.56075

Independent Samples Test

		Levene's Test for Equality of Variances		t-test for Equality of Means							
						Significance				95% Confidence Interval of the Difference	
		F	Sig.	t	df	One-Sided p	Two-Sided p	Mean Difference	Std. Error Difference	Lower	Upper
Integral	Equal variances assumed	.016	.907	-3.922	4	.009	.017	-3.20000	.81582	-5.46507	-.93493
	Equal variances not assumed			-3.922	3.988	.009	.017	-3.20000	.81582	-5.46778	-.93222

Independent Samples Effect Sizes

		Standardizer[a]	Point Estimate	95% Confidence Interval Lower	95% Confidence Interval Upper
Integral	Cohen's d	.99917	-3.203	-5.815	-.482
	Hedges' correction	1.25227	-2.555	-4.640	-.385
	Glass's delta	.97125	-3.295	-6.652	.061

a. The denominator used in estimating the effect sizes.
Cohen's d uses the pooled standard deviation.
Hedges' correction uses the pooled standard deviation, plus a correction factor.
Glass's delta uses the sample standard deviation of the control (i.e., the second) group.

Figure S103: Results of a t-test comparing the relative abundanve of isomer –96.2 ppm between **F-Bull-PMA-Br$_2$-Cold** and **F-Bull-PMA-Br$_2$-Warmed** (p = 0.017)

Group Statistics

	Treatment	N	Mean	Std. Deviation	Std. Error Mean
Integral	Cold	3	13.1667	1.66233	.95975
	Warm	3	8.8333	.80829	.46667

Independent Samples Test

		Levene's Test for Equality of Variances		t-test for Equality of Means							
						Significance				95% Confidence Interval of the Difference	
		F	Sig.	t	df	One-Sided p	Two-Sided p	Mean Difference	Std. Error Difference	Lower	Upper
Integral	Equal variances assumed	1.226	.330	4.061	4	.008	.015	4.33333	1.06719	1.37035	7.29632
	Equal variances not assumed			4.061	2.896	.014	.029	4.33333	1.06719	.86677	7.79990

Independent Samples Effect Sizes

		Standardizer[a]	Point Estimate	95% Confidence Interval Lower	95% Confidence Interval Upper
Integral	Cohen's d	1.30703	3.315	.537	5.989
	Hedges' correction	1.63812	2.645	.428	4.779
	Glass's delta	.80829	5.361	.458	10.501

a. The denominator used in estimating the effect sizes.
Cohen's d uses the pooled standard deviation.
Hedges' correction uses the pooled standard deviation, plus a correction factor.
Glass's delta uses the sample standard deviation of the control (i.e., the second) group.

Figure S104: Results of a t-test comparing the relative abundanve of isomer –96.7 ppm between **F-Bull-PMA-Br$_2$-Cold** and **F-Bull-PMA-Br$_2$-Warmed** (p = 0.015)

T-Test

Group Statistics

	Treatment	N	Mean	Std. Deviation	Std. Error Mean
I972	Warm	3	8.0667	.45092	.26034
	Cold	3	5.0667	1.00167	.57831

Independent Samples Test

		t-test for Equality of Means							
				Significance				95% Confidence Interval of the Difference	
		t	df	One-Sided p	Two-Sided p	Mean Difference	Std. Error Difference	Lower	Upper
I972	Equal variances assumed	4.730	4	.005	.009	3.00000	.63421	1.23915	4.76085
	Equal variances not assumed	4.730	2.779	.011	.021	3.00000	.63421	.88758	5.11242

Homogeneity of Variance Test

		Levene's Test for Equality of Variances	
		F	Sig.
I972	Equal variances assumed	2.888	.164

Independent Samples Effect Sizes

				95% Confidence Interval	
		Standardizer[a]	Point Estimate	Lower	Upper
I972	Cohen's d	.77675	3.862	.793	6.845
	Hedges' correction	.97351	3.082	.633	5.461
	Glass's delta	1.00167	2.995	-.149	6.107

a. The denominator used in estimating the effect sizes.
Cohen's d uses the pooled standard deviation.
Hedges' correction uses the pooled standard deviation, plus a correction factor.
Glass's delta uses the sample standard deviation of the control (i.e., the second) group.

Figure S105: Results of a t-test comparing the relative abundanve of isomer –97.2 ppm between **F-Bull-PMA-Br$_2$-Cold** and **F-Bull-PMA-Br$_2$-Warmed** (p = 0.009)

T-Test

Group Statistics

	Treatment	N	Mean	Std. Deviation	Std. Error Mean
I977	Warm	3	12.7000	1.30000	.75056
	Cold	3	15.2000	1.70587	.98489

Independent Samples Test

		t-test for Equality of Means							
				Significance				95% Confidence Interval of the Difference	
		t	df	One-Sided p	Two-Sided p	Mean Difference	Std. Error Difference	Lower	Upper
I977	Equal variances assumed	-2.019	4	.057	.114	-2.50000	1.23828	-5.93801	.93801
	Equal variances not assumed	-2.019	3.737	.059	.119	-2.50000	1.23828	-6.03552	1.03552

Independent Samples Effect Sizes

				95% Confidence Interval	
		Standardizer[a]	Point Estimate	Lower	Upper
I977	Cohen's d	1.51658	-1.648	-3.528	.358
	Hedges' correction	1.90074	-1.315	-2.815	.286
	Glass's delta	1.70587	-1.466	-3.464	.697

a. The denominator used in estimating the effect sizes.
Cohen's d uses the pooled standard deviation.
Hedges' correction uses the pooled standard deviation, plus a correction factor.
Glass's delta uses the sample standard deviation of the control (i.e., the second) group.

Figure S106: Results of a t-test comparing the relative abundanve of isomer –97.7 ppm between **F-Bull-PMA-Br$_2$-Cold** and **F-Bull-PMA-Br$_2$-Warmed** (p = 0.114)

5.0: COMPUTATIONAL DETAILS

5.1: DFT CALCULATIONS AND POPULATION ANALYSIS AT 213 K

The small molecule **F-Bull-MeOMe** (Main Text **Figure 2**) was used as a small-molecule model system for all computational studies. All initial sets of Cartesian coordinates sampling the shapeshifting and conformational isomers and transition state geometries of **F-Bull-MeOMe** were generated *via* the in-house-developed *bullviso* code[14]. *bullviso* is publicly available under the GNU Public License (GPLv3) on GitLab. The constitutional isomers/transition states of **F-Bull-MeOMe** were generated systematically/exhaustively using *bullviso*; the configurational and conformational isomers/transition states were generated according to the experimental torsion distance geometry (ETDG) with 'basic knowledge' (+K) embedding approach (ETKDGv3)[15,16] [1415]as implemented in RDKit[17,18]. The PMA substituent chains were approximated as CH_2OMe groups. Root mean square deviation (RMSD) filter with a threshold of 0.5 Å was used to prune the initial set of configurational and conformational isomers/transition states. All configurational and ten lowest-energy conformational isomers/transition states passing the RMSD filter were subsequently (pre-) optimized at the GFN2-xTB[19,20] (extended tight binding; xTB) level of theory using *xtb* (v6.4.1)[21]. An SCF convergence criterion of 1.0×10^{-6} a.u. was used with convergence criteria of 5.0×10^{-6} and 1.0×10^{-3} a.u. for the energy change and gradient, respectively, in all geometry optimizations. All unique configurational and ten conformational isomers/transition states verified at the GFN2-xTB level of theory were progressed to density functional theory (DFT) geometry optimization. All transition state labels were generated according to earlier published method by Fallon *et al.*[22]

All DFT geometry optimizations and energy evaluations of **F-Bull-MeOMe** were carried out at the PBE0-D3 level of theory (i.e., with the PBE0[23–25] density functional of Adamo and Barone coupled with the D3[26] dispersion correction of Grimme et al.) using Gaussian 16[27]. All calculations were carried out under the resolution-of-identity (RI) approximation for the Coulomb integrals (RIJONX). A tightened SCF convergence criterion of 1.0×10^{-9} a.u. was used in all calculations; convergence criteria of 2.0×10^{-7} and $3.0{\times}10^{-5}$ a.u. were used for the energy change and gradient, respectively, in all geometry optimizations. The def2-SV(P)[28] basis set of Weigend and Ahlrichs was coupled with the def2/J auxilliary basis set; the two were used together throughout.

The proper convergence of all geometry optimizations to real minima/transition state was verified using in-house developed python script (**SI Section 5.4**). Python script was used to process raw ground and transition state DFT data, where the ground state convergence was verified *via* vibrational frequency

inspection and a single rotamer with the lowest energy for each isomer was kept and used for further analysis. At the first instance the transition state geometry has been verified *via* vibrational frequency expecting, where only the structures with a single negative vibrational frequency were kept. Transition states corresponding to the rotation of a chain substituent have been filtered out by checking the maximum atom displacement corresponding to the single negative vibrational frequency mode. Those transition states that showed the largest atom displacement for the atoms not belonging to the bullvalene core were removed from analysis. Finally, a single rotamer with the lowest energy for each transition state was kept and used for further analysis.

Energies of the optimised ground state (**Table S3**) were used to calculated Boltzmann population percentages for each isomer (**Table S4**). Energies of the optimized ground state (**Table S3**) and transition geometries (**Table S5**) were used for the calculation of the energy barriers and Cope rearrangement rate constants at 213 K. The forward and reverse rate constants (and energy barriers) have been calculated relative to the starting isomer, respectively. Those values were used for the kinetic modelling (**SI Section 5.3**).

Thermodynamic network (**Figure S107**) and kinetic network (**Figure S108**) of **F-Bull-MeOMe** were built using the ground state and transition state DFT optimised energies, respectively, in Cytoscape (https://cytoscape.org).[29,30]

Table S3: Summary of Gibbs energies, $G_{298.15\ \mathrm{K}}$, and relative Gibbs energies, $\Delta G_{298.15\ \mathrm{K}}$, for the isomers of **F-Bull-MeOMe**. All values of $\Delta G_{298.15\ \mathrm{K}}$ are tabulated relative to the lowest-energy isomer of **F-Bull-MeOMe**. All values are given as evaluated at the PBE0-D3/def2-SV(P) level of theory.

Isomer Barcode	$G_{298.15\ \mathrm{K}}$ (Hartree)	$G_{298.15\ \mathrm{K}}$ (kcal·mol^{-1})	$\Delta G_{298.15\ \mathrm{K}}$ (kcal·mol^{-1})
0000000112	–792.1594	–492259.664	7.2575
0000000121	–792.16304	–492261.925	4.9968
0000000211	–792.16252	–492261.606	5.3156
0000001012	–792.16738	–492264.625	2.2961
0000001021	–792.16289	–492261.832	5.0894
0000001102	–792.16376	–492262.375	4.5469
0000001120	–792.16258	–492261.639	5.2826
0000001201	–792.16371	–492262.345	4.5767
0000001210	–792.16679	–492264.256	2.6659
0000002011	–792.15908	–492259.467	7.4545

0000002101	–792.15944	–492259.691	7.2308
0000002110	–792.1567	–492257.989	8.9322
0000010012	–792.16653	–492264.094	2.8281
0000010021	–792.15969	–492259.844	7.0773
0000010102	–792.16794	–492264.974	1.8313
0000010120	–792.16956	–492265.981	0.9408
0000010201	–792.16327	–492262.073	4.8489
0000010210	–792.16949	–492265.937	0.6655
0000011002	–792.16675	–492264.235	2.6764
0000011020	–792.1674	–492264.636	1.4641
0000011200	–792.16793	–492264.964	1.9568
0000012001	–792.15831	–492258.989	7.9330
0000012010	–792.16597	–492263.748	2.6901
0000012100	–792.16329	–492262.081	4.8408
0000020011	–792.15969	–492259.844	7.0773
0000020101	–792.16424	–492262.674	4.1977
0000020110	–792.16232	–492261.478	5.4032
0000021001	–792.16306	–492261.942	4.9794
0000021010	–792.16749	–492264.690	1.8524
0000021100	–792.1631	–492261.962	4.9595
0000100012	–792.16813	–492265.090	1.8313
0000100021	–792.16432	–492262.724	4.1977
0000100102	–792.16784	–492264.908	2.0134
0000100120	–792.16675	–492264.230	2.6920
0000100201	–792.16586	–492263.677	3.2444
0000100210	–792.16944	–492265.902	1.0191
0000101002	–792.16748	–492264.687	2.2346
0000101020	–792.16742	–492264.651	2.2707
0000101200	–792.16492	–492263.098	2.6025
0000102001	–792.16147	–492260.954	5.8146
0000102010	–792.16584	–492263.667	3.2550
0000102100	–792.16255	–492261.620	5.3019
0000110020	–792.16238	–492261.518	5.4032
0000110200	–792.16192	–492261.229	4.8582
0000112000	–792.15894	–492259.378	7.5440
0000120010	–792.16916	–492265.730	0.9408
0000120100	–792.16675	–492264.230	2.6920
0000121000	–792.16681	–492264.269	2.6522
0000200011	–792.16204	–492261.305	4.8489

0000200101	–792.16586	–492263.677	3.2444
0000200110	–792.16326	–492262.063	4.8582
0000201001	–792.16371	–492262.346	4.0423
0000201010	–792.16878	–492265.494	1.4280
0000201100	–792.16346	–492262.186	4.5730
0000210010	–792.17001	–492266.256	0.6655
0000210100	–792.16911	–492265.701	1.0191
0000211000	–792.16826	–492265.169	1.7337
0001000012	–792.16677	–492264.245	2.6764
0001000021	–792.16306	–492261.942	4.9794
0001000102	–792.16672	–492264.215	2.2346
0001000120	–792.16624	–492263.918	2.6522
0001000201	–792.16457	–492262.879	4.0423
0001000210	–792.16829	–492265.188	1.7337
0001001002	–792.16014	–492260.123	6.7989
0001001020	–792.15977	–492259.892	7.0294
0001001200	–792.15973	–492259.870	7.0512
0001002001	–792.15984	–492259.940	6.9816
0001002010	–792.16359	–492262.272	4.6500
0001002100	–792.15853	–492259.123	7.7155
0001010020	–792.1681	–492265.069	1.8524
0001010200	–792.16804	–492265.034	1.4280
0001012000	–792.16196	–492261.254	5.5996
0001020010	–792.16872	–492265.458	1.4641
0001020100	–792.16742	–492264.651	2.2707
0001021000	–792.15976	–492259.891	7.0294
0001100020	–792.16309	–492261.957	4.9595
0001100200	–792.16372	–492262.349	4.5730
0001102000	–792.15798	–492258.781	8.1393
0001200010	–792.16793	–492264.965	1.9568
0001200100	–792.16689	–492264.319	2.6025
0001201000	–792.15838	–492259.034	7.0512
0002000011	–792.15828	–492258.971	7.9330
0002000101	–792.16172	–492261.107	5.8146
0002000110	–792.15815	–492258.886	7.5440
0002001001	–792.15789	–492258.729	6.9816
0002001010	–792.16207	–492261.322	5.5996
0002001100	–792.15798	–492258.782	8.1393
0002010010	–792.16675	–492264.232	2.6901

0002010100	–792.16387	–492262.441	3.2550
0002011000	–792.1617	–492261.097	4.6500
0002100010	–792.16329	–492262.080	4.8408
0002100100	–792.16254	–492261.619	5.3019
0002101000	–792.15866	–492259.206	7.7155
0010010020	–792.17108	–492266.922	0.0000
0010010200	–792.17036	–492266.477	0.4443
0010012000	–792.16607	–492263.809	3.1127
0010020100	–792.17015	–492266.342	0.5599
0010021000	–792.16875	–492265.474	1.2422
0010100020	–792.17018	–492266.362	0.5599
0010100200	–792.16805	–492265.041	0.7967
0010102000	–792.16544	–492263.418	3.5042
0010200100	–792.1698	–492266.125	0.7967
0010201000	–792.16882	–492265.518	1.3429
0011000020	–792.16908	–492265.679	1.2422
0011000200	–792.16892	–492265.579	1.3429
0011002000	–792.16197	–492261.262	5.6592
0012000100	–792.16375	–492262.366	3.5042
0012001000	–792.16188	–492261.205	5.6592
0020100100	–792.16846	–492265.293	1.6281
0020101000	–792.16676	–492264.236	2.6858
0021000100	–792.16641	–492264.018	2.6858
0021001000	–792.16076	–492260.507	6.4142
0100100200	–792.16755	–492264.732	2.1899
0100102000	–792.16449	–492262.830	4.0920
0100201000	–792.1676	–492264.762	2.1600
0101000200	–792.16713	–492264.471	2.1600
0101002000	–792.16332	–492262.099	4.8222
0102001000	–792.16319	–492262.021	4.8222
0201001000	–792.15984	–492259.938	6.9835
1001002000	–792.15593	–492257.509	9.4126

Table S4: Boltzmann distribution population percentages of **F-Bull-MeOMe** isomers at 213 K.

Isomer Barcode	Botlzmann Population Percentage (%)
0010010020	28.366
0010010200	9.929
0010020100	7.556
0010100020	7.556
0000010210	5.887
0000210010	5.887
0010100200	4.319
0010200100	4.319
0000010120	3.072
0000120010	3.072
0000100210	2.553
0000210100	2.553
0010021000	1.507
0011000020	1.507
0010201000	1.188
0011000200	1.188
0000201010	0.972
0001010200	0.972
0000011020	0.892
0001020010	0.892
0020100100	0.606
0000211000	0.472
0001000210	0.472
0000010102	0.375
0000100012	0.375
0000021010	0.356
0001010020	0.356
0000011200	0.279
0001200010	0.279
0000100102	0.244
0100201000	0.172
0101000200	0.172
0100100200	0.161
0000101002	0.145
0001000102	0.145
0000101020	0.133
0001020100	0.133

0000001012	0.125
0000101200	0.061
0001200100	0.061
0000121000	0.054
0001000120	0.054
0000001210	0.052
0000011002	0.051
0001000012	0.051
0020101000	0.050
0021000100	0.050
0000012010	0.049
0002010010	0.049
0000100120	0.049
0000120100	0.049
0000010012	0.036
0010012000	0.018
0000100201	0.013
0000200101	0.013
0000102010	0.013
0002010100	0.013
0010102000	7.20E-03
0012000100	7.20E-03
0000201001	2.02E-03
0001000201	2.02E-03
0100102000	1.79E-03
0000020101	1.40E-03
0000100021	1.40E-03
0000001102	6.13E-04
0000201100	5.76E-04
0001100200	5.76E-04
0000001201	5.71E-04
0001002010	4.80E-04
0002011000	4.80E-04
0101002000	3.20E-04
0102001000	3.20E-04
0000012100	3.06E-04
0002100010	3.06E-04
0000010201	3.00E-04
0000200011	3.00E-04

0000110200	2.94E-04
0000200110	2.94E-04
0000021100	2.31E-04
0001100020	2.31E-04
0000021001	2.21E-04
0001000021	2.21E-04
0000000121	2.12E-04
0000001021	1.70E-04
0000001120	1.08E-04
0000102100	1.03E-04
0002100100	1.03E-04
0000000211	9.97E-05
0000020110	8.10E-05
0000110020	8.10E-05
0001012000	5.09E-05
0002001010	5.09E-05
0011002000	4.42E-05
0012001000	4.42E-05
0000102001	3.07E-05
0002000101	3.07E-05
0021001000	7.43E-06
0001001002	3.00E-06
0001002001	1.95E-06
0002001001	1.95E-06
0201001000	1.94E-06
0001001020	1.74E-06
0001021000	1.74E-06
0001001200	1.65E-06
0001201000	1.65E-06
0000010021	1.55E-06
0000020011	1.55E-06
0000002101	1.08E-06
0000000112	1.01E-06
0000002011	6.36E-07
0000112000	5.15E-07
0002000110	5.15E-07
0001002100	3.44E-07
0002101000	3.44E-07
0000012001	2.06E-07

0002000011	2.06E-07
0001102000	1.26E-07
0002001100	1.26E-07
0000002110	1.94E-08
1001002000	6.23E-09

Table S5. Summary of transition state energies for the isomers of **F-Bull-PMA**. All values of the relative transition state (TS) energies are tabulated relative to the lowest-energy ground state isomer of **F-Bull-PMA**. All E_a values are tabulated relative to the source isomer ground state energy. All values are given as evaluated at the PBE0-D3/def2-SV(P) level of theory at 298.15 K.

Source isomer barcode	Target isomer barcode	TS label	TS Energy (Hartree)	TS Energy (kcal·mol^{-1})	Relative TS Energy (kcal·mol^{-1})	E_a (kcal·mol^{-1})
0000000112	0000002110	0000000112	–792.1375	–492246.06	20.87	13.61
0000000112	0002001100	0000110002	–792.13441	–492244.14	22.78	15.53
0000000112	0001102000	0001102000	–792.13381	–492243.76	23.16	15.90
0000000121	0000001210	0000000121	–792.14034	–492247.83	19.10	14.10
0000000121	0001002100	0000120001	–792.13365	–492243.67	23.26	18.26
0000000121	0002101000	0002101000	–792.13428	–492244.05	22.87	17.87
0000000211	0000001120	0000000211	–792.13895	–492246.96	19.96	14.65
0000000211	0001001200	0000210001	–792.13525	–492244.67	22.26	16.95
0000000211	0001201000	0001201000	–792.13525	–492244.67	22.26	16.95
0000001012	0000002101	0000001012	–792.14249	–492249.16	17.76	15.47
0000001012	0002001010	0001010002	–792.14055	–492247.94	18.97	16.67
0000001012	0001012000	0001012000	–792.13866	–492246.77	20.14	17.85
0000001021	0000001201	0000001021	–792.14148	–492248.52	18.39	13.31
0000001021	0001002010	0001020001	–792.1372	–492245.87	21.05	15.96
0000001021	0002011000	0002011000	–792.13721	–492245.87	21.05	15.96
0000001102	0000002011	0000001102	–792.13848	–492246.65	20.26	15.71
0000001102	0002000110	0001100002	–792.13718	–492245.87	21.06	16.52
0000001102	0000112000	0000112000	–792.13644	–492245.39	21.53	16.98
0000001120	0000000211	0000000211	–792.13895	–492246.96	19.96	14.68
0000001120	0000002110	0001120000	–792.13193	–492242.59	24.33	19.05
0000001120	0000002110	0002110000	–792.13193	–492242.59	24.33	19.05
0000001201	0000001021	0000001021	–792.14148	–492248.52	18.39	13.82
0000001201	0001000210	0001200001	–792.13991	–492247.56	19.37	14.79
0000001201	0000211000	0000211000	–792.13991	–492247.56	19.37	14.79
0000001210	0000000121	0000000121	–792.14034	–492247.83	19.10	16.43
0000001210	0000001210	0001210000	–792.13929	–492247.18	19.75	17.09
0000001210	0000001210	0001210000	–792.13929	–492247.18	19.75	17.09
0000002011	0000001102	0000001102	–792.13848	–492246.65	20.26	12.80
0000002011	0001001020	0002010001	–792.13543	–492244.77	22.15	14.70
0000002011	0001021000	0001021000	–792.13446	–492244.17	22.75	15.30
0000002101	0000001012	0000001012	–792.14249	–492249.16	17.76	10.53
0000002101	0001000120	0002100001	–792.13698	–492245.75	21.19	13.96

0000002101	0000121000	0000121000	–792.13698	–492245.75	21.19	13.96
0000002110	0000000112	0000000112	–792.1375	–492246.06	20.87	11.93
0000002110	0000001120	0002110000	–792.13193	–492242.59	24.33	15.40
0000002110	0000001120	0001120000	–792.13193	–492242.59	24.33	15.40
0000010012	0002101000	0000010012	–792.13977	–492247.47	19.46	16.63
0000010012	1001002000	0010010002	–792.13608	–492245.17	21.75	18.92
0000010012	0001002100	0001002100	–792.13927	–492247.16	19.77	16.94
0000010021	0001201000	0000010021	–792.13581	–492245.00	21.92	14.84
0000010021	1001002000	0010020001	–792.13383	–492243.79	23.15	16.07
0000010021	0002001100	0002001100	–792.13177	–492242.50	24.43	17.35
0000010102	0002011000	0000010102	–792.14198	–492248.85	18.08	16.25
0000010102	0101002000	0010100002	–792.14081	–492248.11	18.81	16.98
0000010102	0000102100	0000102100	–792.14091	–492248.18	18.75	16.92
0000010120	0000211000	0000010120	–792.14259	–492249.21	17.70	16.76
0000010120	0002101000	0010120000	–792.13859	–492246.73	20.19	19.25
0000010120	0002100100	0002100100	–792.13924	–492247.13	19.78	18.84
0000010201	0001021000	0000010201	–792.13747	–492246.03	20.89	16.04
0000010201	0201001000	0010200001	–792.13363	–492243.64	23.27	18.42
0000010201	0000201100	0000201100	–792.13615	–492245.22	21.70	16.86
0000010210	0000121000	0000010210	–792.14406	–492250.14	16.79	16.12
0000010210	0001201000	0010210000	–792.13902	–492246.99	19.92	19.26
0000010210	0001200100	0001200100	–792.1422	–492248.97	17.95	17.28
0000011002	0002001001	0000011002	–792.13953	–492247.32	19.60	16.93
0000011002	0011002000	0011000002	–792.13945	–492247.28	19.65	16.98
0000011002	0000012100	0000012100	–792.14097	–492248.21	18.71	16.04
0000011020	0000201001	0000011020	–792.14304	–492249.50	17.43	15.96
0000011020	0002011000	0011020000	–792.14093	–492248.18	18.73	17.27
0000011020	0002010100	0002010100	–792.14014	–492247.71	19.22	17.76
0000011200	0000021001	0000011200	–792.14122	–492248.37	18.55	16.60
0000011200	0000211000	0011200000	–792.14056	–492247.97	18.96	17.01
0000011200	0000210100	0000210100	–792.1422	–492248.97	17.94	15.99
0000012001	0001001002	0000012001	–792.13801	–492246.37	20.55	12.62
0000012001	0021001000	0012000001	–792.13582	–492245.00	21.91	13.98
0000012001	0000021100	0000021100	–792.13568	–492244.93	21.99	14.06
0000012010	0000101002	0000012010	–792.14341	–492249.74	17.19	14.50
0000012010	0001021000	0012010000	–792.13849	–492246.68	20.25	17.56
0000012010	0001020100	0001020100	–792.14118	–492248.35	18.58	15.89
0000012100	0000011002	0000012100	–792.14097	–492248.21	18.71	13.87
0000012100	0000121000	0012100000	–792.13779	–492246.25	20.69	15.84

0000012100	0000120100	0000120100	−792.139	−492246.99	19.93	15.09
0000020011	0001102000	0000020011	−792.13177	−492242.50	24.42	17.35
0000020011	1001002000	0020010001	−792.13306	−492243.28	23.63	16.55
0000020011	0001001200	0001001200	−792.13477	−492244.36	22.56	15.48
0000020101	0001012000	0000020101	−792.1383	−492246.56	20.37	16.17
0000020101	0102001000	0020100001	−792.13545	−492244.79	22.14	17.94
0000020101	0000101200	0000101200	−792.14069	−492248.04	18.89	14.69
0000020110	0000112000	0000020110	−792.13611	−492245.20	21.73	16.33
0000020110	0001102000	0020110000	−792.13451	−492244.19	22.72	17.32
0000020110	0001100200	0001100200	−792.1399	−492247.54	19.38	13.97
0000021001	0001002001	0000021001	−792.13583	−492245.00	21.91	16.93
0000021001	0012001000	0012001000	−792.13658	−492245.48	21.44	16.46
0000021001	0000011200	0000011200	−792.14122	−492248.37	18.55	13.57
0000021010	0000102001	0000021010	−792.13994	−492247.59	19.35	17.49
0000021010	0001012000	0021010000	−792.13913	−492247.06	19.85	18.00
0000021010	0001010200	0001010200	−792.14336	−492249.69	17.23	15.37
0000021100	0000012001	0000021100	−792.13568	−492244.93	21.99	17.04
0000021100	0000112000	0021100000	−792.13584	−492245.03	21.90	16.94
0000021100	0000110200	0000110200	−792.1375	−492246.06	20.87	15.91
0000100012	0002100100	0000100012	−792.14069	−492248.04	18.89	17.05
0000100012	0102001000	0100010002	−792.14081	−492248.11	18.81	16.98
0000100012	0001002010	0001002010	−792.142	−492248.85	18.07	16.24
0000100021	0001200100	0000100021	−792.14069	−492248.04	18.88	14.69
0000100021	0101002000	0100020001	−792.13544	−492244.79	22.14	17.95
0000100021	0002001010	0002001010	−792.13696	−492245.72	21.20	17.00
0000100102	0002010100	0000100102	−792.143	−492249.47	17.45	15.43
0000100102	0100102000	0100100002	−792.13972	−492247.44	19.49	17.47
0000100102	0000102010	0000102010	−792.14093	−492248.18	18.73	16.72
0000100120	0000210100	0000100120	−792.14309	−492249.52	17.39	14.70
0000100120	0002100100	0100120000	−792.13692	−492245.70	21.23	18.53
0000100120	0002100010	0002100010	−792.13925	−492247.13	19.78	17.09
0000100201	0001020100	0000100201	−792.14228	−492249.02	17.90	14.65
0000100201	0101000200	0100200001	−792.13979	−492247.49	19.44	16.20
0000100201	0000201010	0000201010	−792.14053	−492247.94	18.98	15.74
0000100210	0000120100	0000100210	−792.14335	−492249.69	17.23	16.21
0000100210	0001200100	0100210000	−792.14005	−492247.63	19.28	18.26
0000100210	0001200010	0001200010	−792.1422	−492248.97	17.94	16.92
0000101002	0002000101	0000101002	−792.14102	−492248.23	18.68	16.45
0000101002	0010102000	0010102000	−792.14138	−492248.47	18.45	16.22

0000101002	0000012010	0000012010	–792.14341	–492249.74	17.19	14.96
0000101020	0000200101	0000101020	–792.14173	–492248.69	18.24	15.97
0000101020	0002010100	0101020000	–792.13753	–492246.08	20.84	18.57
0000101020	0002010010	0002010010	–792.13944	–492247.25	19.66	17.39
0000101200	0000020101	0000101200	–792.14069	–492248.04	18.89	16.28
0000101200	0000210100	0101200000	–792.14005	–492247.63	19.28	16.68
0000101200	0000210010	0000210010	–792.14239	–492249.09	17.83	15.23
0000102001	0001000102	0000102001	–792.13934	–492247.20	19.72	13.91
0000102001	0020101000	0020101000	–792.13866	–492246.77	20.15	14.33
0000102001	0000021010	0000021010	–792.13994	–492247.59	19.35	13.53
0000102010	0000100102	0000102010	–792.14093	–492248.18	18.73	15.48
0000102010	0001020100	0102010000	–792.13863	–492246.75	20.16	16.91
0000102010	0001020010	0001020010	–792.14035	–492247.83	19.09	15.84
0000102100	0000010102	0000102100	–792.14091	–492248.18	18.75	13.45
0000102100	0000120100	0102100000	–792.1363	–492245.32	21.61	16.31
0000102100	0000120010	0000120010	–792.13951	–492247.30	19.62	14.31
0000110020	0000201100	0000110020	–792.1399	–492247.54	19.38	13.97
0000110020	0002001100	0110020000	–792.13451	–492244.19	22.72	17.32
0000110020	0002000110	0002000110	–792.13632	–492245.32	21.60	16.19
0000110200	0000021100	0000110200	–792.1375	–492246.06	20.87	16.01
0000110200	0000201100	0110200000	–792.13363	–492243.64	23.27	18.41
0000110200	0000200110	0000200110	–792.13755	–492246.08	20.83	15.97
0000112000	0000001102	0000112000	–792.13644	–492245.39	21.53	13.98
0000112000	0000021100	0021100000	–792.13584	–492245.03	21.90	14.35
0000112000	0000020110	0000020110	–792.13611	–492245.20	21.73	14.19
0000120010	0000102100	0000120010	–792.13951	–492247.30	19.62	18.67
0000120010	0001002100	0120010000	–792.1386	–492246.75	20.18	19.24
0000120010	0001000210	0001000210	–792.14325	–492249.62	17.29	16.35
0000120100	0000012100	0000120100	–792.139	–492246.99	19.93	17.24
0000120100	0000102100	0102100000	–792.1363	–492245.32	21.61	18.92
0000120100	0000100210	0000100210	–792.14335	–492249.69	17.23	14.54
0000121000	0000002101	0000121000	–792.13698	–492245.75	21.19	18.53
0000121000	0000012100	0012100000	–792.13779	–492246.25	20.69	18.03
0000121000	0000010210	0000010210	–792.14406	–492250.14	16.79	14.14
0000200011	0001100200	0000200011	–792.13415	–492243.98	22.95	18.10
0000200011	0201001000	0200010001	–792.13363	–492243.64	23.27	18.42
0000200011	0001001020	0001001020	–792.13887	–492246.92	20.01	15.17
0000200101	0001010200	0000200101	–792.1405	–492247.92	19.00	15.75
0000200101	0100201000	0100201000	–792.13979	–492247.47	19.44	16.20

0000200101	0000101020	0000101020	–792.14173	–492248.69	18.24	14.99
0000200110	0000110200	0000200110	–792.13755	–492246.08	20.83	15.97
0000200110	0001100200	0200110000	–792.13363	–492243.64	23.27	18.41
0000200110	0001100020	0001100020	–792.13935	–492247.20	19.72	14.86
0000201001	0001000201	0000201001	–792.13818	–492246.49	20.44	16.40
0000201001	0010201000	0010201000	–792.13905	–492247.01	19.90	15.86
0000201001	0000011020	0000011020	–792.14304	–492249.50	17.43	13.38
0000201010	0000100201	0000201010	–792.14053	–492247.94	18.98	17.55
0000201010	0001010200	0201010000	–792.1404	–492247.85	19.06	17.63
0000201010	0001010020	0001010020	–792.14253	–492249.19	17.74	16.31
0000201100	0000010201	0000201100	–792.13615	–492245.22	21.70	17.13
0000201100	0000110200	0110200000	–792.13363	–492243.64	23.27	18.70
0000201100	0000110020	0000110020	–792.1399	–492247.54	19.38	14.80
0000210010	0000101200	0000210010	–792.14239	–492249.09	17.83	17.16
0000210010	0001001200	0210010000	–792.13955	–492247.32	19.59	18.93
0000210010	0001000120	0001000120	–792.14502	–492250.72	16.19	15.53
0000210100	0000011200	0000210100	–792.1422	–492248.97	17.94	16.92
0000210100	0000101200	0101200000	–792.14005	–492247.63	19.28	18.26
0000210100	0000100120	0000100120	–792.14309	–492249.52	17.39	16.37
0000211000	0000001201	0000211000	–792.13991	–492247.56	19.37	17.64
0000211000	0000011200	0011200000	–792.14056	–492247.97	18.96	17.23
0000211000	0000010120	0000010120	–792.14259	–492249.21	17.70	15.97
0001000012	0002100010	0001000012	–792.14179	–492248.73	18.20	15.52
0001000012	0012001000	1000010002	–792.13945	–492247.28	19.65	16.98
0001000012	0001002001	0001002001	–792.13953	–492247.32	19.60	16.93
0001000021	0001200010	0001000021	–792.14203	–492248.88	18.05	13.07
0001000021	0011002000	1000020001	–792.13655	–492245.46	21.46	16.48
0001000021	0002001001	0002001001	–792.13583	–492245.00	21.91	16.93
0001000102	0002010010	0001000102	–792.14255	–492249.19	17.73	15.49
0001000102	0012000100	0100012000	–792.14138	–492248.47	18.45	16.22
0001000102	0000102001	0000102001	–792.13934	–492247.20	19.72	17.49
0001000120	0000210010	0001000120	–792.14502	–492250.72	16.19	13.54
0001000120	0002100010	1000120000	–792.1377	–492246.18	20.74	18.09
0001000120	0000002101	0002100001	–792.13698	–492245.75	21.19	18.53
0001000201	0001020010	0001000201	–792.14304	–492249.50	17.43	13.38
0001000201	0011000200	0200011000	–792.13956	–492247.35	19.58	15.54
0001000201	0000201001	0000201001	–792.13818	–492246.49	20.44	16.40
0001000210	0000120010	0001000210	–792.14325	–492249.62	17.29	15.56
0001000210	0001200010	1000210000	–792.13971	–492247.42	19.49	17.76

0001000210	0000001201	0001200001	–792.13991	–492247.56	19.37	17.63
0001001002	0002000011	0001001002	–792.13801	–492246.37	20.55	13.75
0001001002	0010012000	0010012000	–792.14194	–492248.80	18.11	11.31
0001001002	0000012001	0000012001	–792.13801	–492246.37	20.55	13.75
0001001020	0000200011	0001001020	–792.13887	–492246.92	20.01	12.98
0001001020	0002010010	1001020000	–792.14022	–492247.75	19.18	12.15
0001001020	0000002011	0002010001	–792.13543	–492244.77	22.15	15.12
0001001200	0000020011	0001001200	–792.13477	–492244.36	22.56	15.51
0001001200	0000210010	0210010000	–792.13955	–492247.32	19.59	12.54
0001001200	0000000211	0000210001	–792.13525	–492244.67	22.26	15.21
0001002001	0001000012	0001002001	–792.13953	–492247.32	19.60	12.62
0001002001	0011000020	0020011000	–792.14009	–492247.66	19.25	12.27
0001002001	0000021001	0000021001	–792.13583	–492245.00	21.91	14.92
0001002010	0000100012	0001002010	–792.142	–492248.85	18.07	13.42
0001002010	0001020010	1002010000	–792.14093	–492248.18	18.73	14.08
0001002010	0000001021	0001020001	–792.1372	–492245.87	21.05	16.40
0001002100	0000010012	0001002100	–792.13927	–492247.16	19.77	12.05
0001002100	0000120010	0120010000	–792.1386	–492246.75	20.18	12.47
0001002100	0000000121	0000120001	–792.13365	–492243.67	23.26	15.54
0001010020	0000201010	0001010020	–792.14253	–492249.19	17.74	15.89
0001010020	0002001010	1010020000	–792.14016	–492247.71	19.22	17.36
0001010020	0002000101	0002000101	–792.1414	–492248.47	18.44	16.59
0001010200	0000021010	0001010200	–792.14336	–492249.69	17.23	15.80
0001010200	0000201010	0201010000	–792.1404	–492247.85	19.06	17.63
0001010200	0000200101	0000200101	–792.1405	–492247.92	19.00	17.57
0001012000	0000001012	0001012000	–792.13866	–492246.77	20.14	14.54
0001012000	0000021010	0021010000	–792.13913	–492247.06	19.85	14.25
0001012000	0000020101	0000020101	–792.1383	–492246.56	20.37	14.77
0001020010	0000102010	0001020010	–792.14035	–492247.83	19.09	17.63
0001020010	0001002010	1002010000	–792.14093	–492248.18	18.73	17.27
0001020010	0001000201	0001000201	–792.14304	–492249.50	17.43	15.96
0001020100	0000012010	0001020100	–792.14118	–492248.35	18.58	16.31
0001020100	0000102010	0102010000	–792.13863	–492246.75	20.16	17.89
0001020100	0000100201	0000100201	–792.14228	–492249.02	17.90	15.63
0001021000	0000002011	0001021000	–792.13446	–492244.17	22.75	15.72
0001021000	0000012010	0012010000	–792.13849	–492246.68	20.25	13.22
0001021000	0000010201	0000010201	–792.13747	–492246.03	20.89	13.86
0001100020	0000200110	0001100020	–792.13935	–492247.20	19.72	14.76
0001100020	0002000110	1100020000	–792.13289	–492243.19	23.73	18.77

0001100020	0002000011	0002000011	–792.13541	–492244.77	22.16	17.20
0001100200	0000020110	0001100200	–792.1399	–492247.54	19.38	14.80
0001100200	0000200110	0200110000	–792.13363	–492243.64	23.27	18.69
0001100200	0000200011	0000200011	–792.13415	–492243.98	22.95	18.37
0001102000	0000000112	0001102000	–792.13381	–492243.76	23.16	15.02
0001102000	0000020110	0020110000	–792.13451	–492244.19	22.72	14.58
0001102000	0000020011	0000020011	–792.13177	–492242.50	24.42	16.28
0001200010	0000100210	0001200010	–792.1422	–492248.97	17.94	15.99
0001200010	0001000210	1000210000	–792.13971	–492247.42	19.49	17.54
0001200010	0001000021	0001000021	–792.14203	–492248.88	18.05	16.09
0001200100	0000010210	0001200100	–792.1422	–492248.97	17.95	15.35
0001200100	0000100210	0100210000	–792.14005	–492247.63	19.28	16.68
0001200100	0000100021	0000100021	–792.14069	–492248.04	18.88	16.28
0001201000	0000000211	0001201000	–792.13525	–492244.67	22.26	15.21
0001201000	0000010210	0010210000	–792.13902	–492246.99	19.92	12.87
0001201000	0000010021	0000010021	–792.13581	–492245.00	21.92	14.86
0002000011	0001100020	0002000011	–792.13541	–492244.77	22.16	14.23
0002000011	0021001000	1000021000	–792.13344	–492243.55	23.39	15.45
0002000011	0001001002	0001001002	–792.13801	–492246.37	20.55	12.62
0002000101	0001010020	0002000101	–792.1414	–492248.47	18.44	12.63
0002000101	0021000100	0100021000	–792.13866	–492246.77	20.15	14.33
0002000101	0000101002	0000101002	–792.14102	–492248.23	18.68	12.87
0002000110	0000110020	0002000110	–792.13632	–492245.32	21.60	14.05
0002000110	0001100020	1100020000	–792.13289	–492243.19	23.73	16.18
0002000110	0000001102	0001100002	–792.13718	–492245.87	21.06	13.52
0002001001	0001000021	0002001001	–792.13583	–492245.00	21.91	14.92
0002001001	0010021000	0010021000	–792.14009	–492247.66	19.25	12.27
0002001001	0000011002	0000011002	–792.13953	–492247.32	19.60	12.62
0002001010	0000100021	0002001010	–792.13696	–492245.72	21.20	15.60
0002001010	0001010020	1010020000	–792.14016	–492247.71	19.22	13.62
0002001010	0000001012	0001010002	–792.14055	–492247.94	18.97	13.37
0002001100	0000010021	0002001100	–792.13177	–492242.50	24.43	16.29
0002001100	0000110020	0110020000	–792.13451	–492244.19	22.72	14.58
0002001100	0000000112	0000110002	–792.13441	–492244.14	22.78	14.64
0002010010	0000101020	0002010010	–792.13944	–492247.25	19.66	16.97
0002010010	0001001020	1001020000	–792.14022	–492247.75	19.18	16.49
0002010010	0001000102	0001000102	–792.14255	–492249.19	17.73	15.04
0002010100	0000011020	0002010100	–792.14014	–492247.71	19.22	15.97
0002010100	0000101020	0101020000	–792.13753	–492246.08	20.84	17.59

0002010100	0000100102	0000100102	–792.143	–492249.47	17.45	14.19
0002011000	0000001021	0002011000	–792.13721	–492245.87	21.05	16.40
0002011000	0000011020	0011020000	–792.14093	–492248.18	18.73	14.08
0002011000	0000010102	0000010102	–792.14198	–492248.85	18.08	13.43
0002100010	0000100120	0002100010	–792.13925	–492247.13	19.78	14.94
0002100010	0001000120	1000120000	–792.1377	–492246.18	20.74	15.90
0002100010	0001000012	0001000012	–792.14179	–492248.73	18.20	13.36
0002100100	0000010120	0002100100	–792.13924	–492247.13	19.78	14.48
0002100100	0000100120	0100120000	–792.13692	–492245.70	21.23	15.92
0002100100	0000100012	0000100012	–792.14069	–492248.04	18.89	13.58
0002101000	0000000121	0002101000	–792.13428	–492244.05	22.87	15.15
0002101000	0000010120	0010120000	–792.13859	–492246.73	20.19	12.48
0002101000	0000010012	0000010012	–792.13977	–492247.47	19.46	11.74
0010010020	0201001000	0010010020	–792.14335	–492249.69	17.23	17.23
0010010020	0102001000	0010020010	–792.14308	–492249.52	17.40	17.40
0010010020	0101002000	0020010010	–792.14347	–492249.76	17.16	17.16
0010010200	0021001000	0010010200	–792.1419	–492248.78	18.13	17.68
0010010200	0100201000	0010200010	–792.14019	–492247.73	19.19	18.75
0010010200	0101000200	0200010010	–792.14187	–492248.78	18.15	17.71
0010012000	0001001002	0010012000	–792.14194	–492248.80	18.11	15.00
0010012000	0021000100	0012000010	–792.14163	–492248.61	18.30	15.19
0010012000	0020101000	1000020100	–792.14155	–492248.57	18.35	15.23
0010020100	0012001000	0010020100	–792.14315	–492249.57	17.36	16.80
0010020100	0100102000	0020100010	–792.14082	–492248.11	18.81	18.25
0010020100	0100201000	0100010020	–792.14285	–492249.38	17.54	16.98
0010021000	0002001001	0010021000	–792.14009	–492247.66	19.25	18.01
0010021000	0012000100	0012000100	–792.13949	–492247.30	19.63	18.39
0010021000	0010201000	1000010020	–792.14295	–492249.45	17.48	16.24
0010100020	0101000200	0010100020	–792.14244	–492249.12	17.80	17.24
0010100020	0100102000	0100020010	–792.13897	–492246.96	19.95	19.39
0010100020	0011002000	0020010100	–792.14259	–492249.21	17.70	17.14
0010100200	0020101000	0010100200	–792.14241	–492249.12	17.82	17.02
0010100200	0100100200	0100200010	–792.14158	–492248.59	18.33	17.53
0010100200	0011000200	0200010100	–792.14067	–492248.02	18.89	18.10
0010102000	0000101002	0010102000	–792.14138	–492248.47	18.45	14.95
0010102000	0020100100	0020100100	–792.1407	–492248.04	18.88	15.38
0010102000	0011000020	1000020010	–792.14056	–492247.94	18.97	15.46
0010200100	0010201000	0010200100	–792.14177	–492248.71	18.21	17.42
0010200100	0100100200	0100200100	–792.14158	–492248.59	18.33	17.54

0010200100	0021000100	0100010200	–792.14244	–492249.12	17.80	17.00
0010201000	0000201001	0010201000	–792.13905	–492247.01	19.90	18.56
0010201000	0010200100	0010200100	–792.14177	–492248.71	18.21	16.87
0010201000	0010021000	1000010020	–792.14295	–492249.45	17.48	16.14
0011000020	0011000200	0011000020	–792.14163	–492248.61	18.30	17.06
0011000020	0010102000	1000020010	–792.14056	–492247.94	18.97	17.72
0011000020	0001002001	0020011000	–792.14009	–492247.66	19.25	18.01
0011000200	0011000020	0011000020	–792.14163	–492248.61	18.30	16.96
0011000200	0010100200	0200010100	–792.14067	–492248.02	18.89	17.55
0011000200	0001000201	0200011000	–792.13956	–492247.35	19.58	18.24
0011002000	0000011002	0011000002	–792.13945	–492247.28	19.65	13.99
0011002000	0010100020	0020010100	–792.14259	–492249.21	17.70	12.04
0011002000	0001000021	1000020001	–792.13655	–492245.46	21.46	15.80
0012000100	0010021000	0012000100	–792.13949	–492247.30	19.63	16.13
0012000100	0020100100	0100020100	–792.14067	–492248.02	18.90	15.39
0012000100	0001000102	0100012000	–792.14138	–492248.47	18.45	14.95
0012001000	0000021001	0012001000	–792.13658	–492245.48	21.44	15.78
0012001000	0010020100	0010020100	–792.14315	–492249.57	17.36	11.70
0012001000	0001000012	1000010002	–792.13945	–492247.28	19.65	13.99
0020100100	0010102000	0020100100	–792.1407	–492248.04	18.88	17.25
0020100100	0100100200	0100100020	–792.14119	–492248.35	18.57	16.95
0020100100	0012000100	0100020100	–792.14067	–492248.02	18.90	17.27
0020101000	0000102001	0020101000	–792.13866	–492246.77	20.15	17.46
0020101000	0010100200	0010100200	–792.14241	–492249.12	17.82	15.13
0020101000	0010012000	1000020100	–792.14155	–492248.57	18.35	15.66
0021000100	0010012000	0012000010	–792.14163	–492248.61	18.30	15.62
0021000100	0010200100	0100010200	–792.14244	–492249.12	17.80	15.11
0021000100	0002000101	0100021000	–792.13866	–492246.77	20.15	17.46
0021001000	0000012001	0012000001	–792.13582	–492245.00	21.91	15.49
0021001000	0010010200	0010010200	–792.1419	–492248.78	18.13	11.71
0021001000	0002000011	1000021000	–792.13344	–492243.55	23.39	16.97
0100100200	0020100100	0100100020	–792.14119	–492248.35	18.57	16.38
0100100200	0010200100	0100200100	–792.14158	–492248.59	18.33	16.14
0100100200	0010100200	0100200010	–792.14158	–492248.59	18.33	16.14
0100102000	0000100102	0100100002	–792.13972	–492247.44	19.49	15.39
0100102000	0010020100	0020100010	–792.14082	–492248.11	18.81	14.71
0100102000	0010100020	0100020010	–792.13897	–492246.96	19.95	15.86
0100201000	0000200101	0100201000	–792.13979	–492247.47	19.44	17.28
0100201000	0010010200	0010200010	–792.14019	–492247.73	19.19	17.03

0100201000	0010020100	0100010020	–792.14285	–492249.38	17.54	15.38
0101000200	0010100020	0010100020	–792.14244	–492249.12	17.80	15.64
0101000200	0010010200	0200010010	–792.14187	–492248.78	18.15	15.99
0101000200	0000100201	0100200001	–792.13979	–492247.49	19.44	17.28
0101002000	0000010102	0010100002	–792.14081	–492248.11	18.81	13.99
0101002000	0010010020	0020010010	–792.14347	–492249.76	17.16	12.34
0101002000	0000100021	0100020001	–792.13544	–492244.79	22.14	17.32
0102001000	0000020101	0020100001	–792.13545	–492244.79	22.14	17.32
0102001000	0010010020	0010020010	–792.14308	–492249.52	17.40	12.57
0102001000	0000100012	0100010002	–792.14081	–492248.11	18.81	13.99
0201001000	0000010201	0010200001	–792.13363	–492243.64	23.27	16.29
0201001000	0010010020	0010010020	–792.14335	–492249.69	17.23	10.25
0201001000	0000200011	0200010001	–792.13363	–492243.64	23.27	16.29
1001002000	0000010012	0010010002	–792.13608	–492245.17	21.75	12.33
1001002000	0000020011	0020010001	–792.13306	–492243.28	23.63	14.21
1001002000	0000010021	0010020001	–792.13383	–492243.79	23.15	13.73

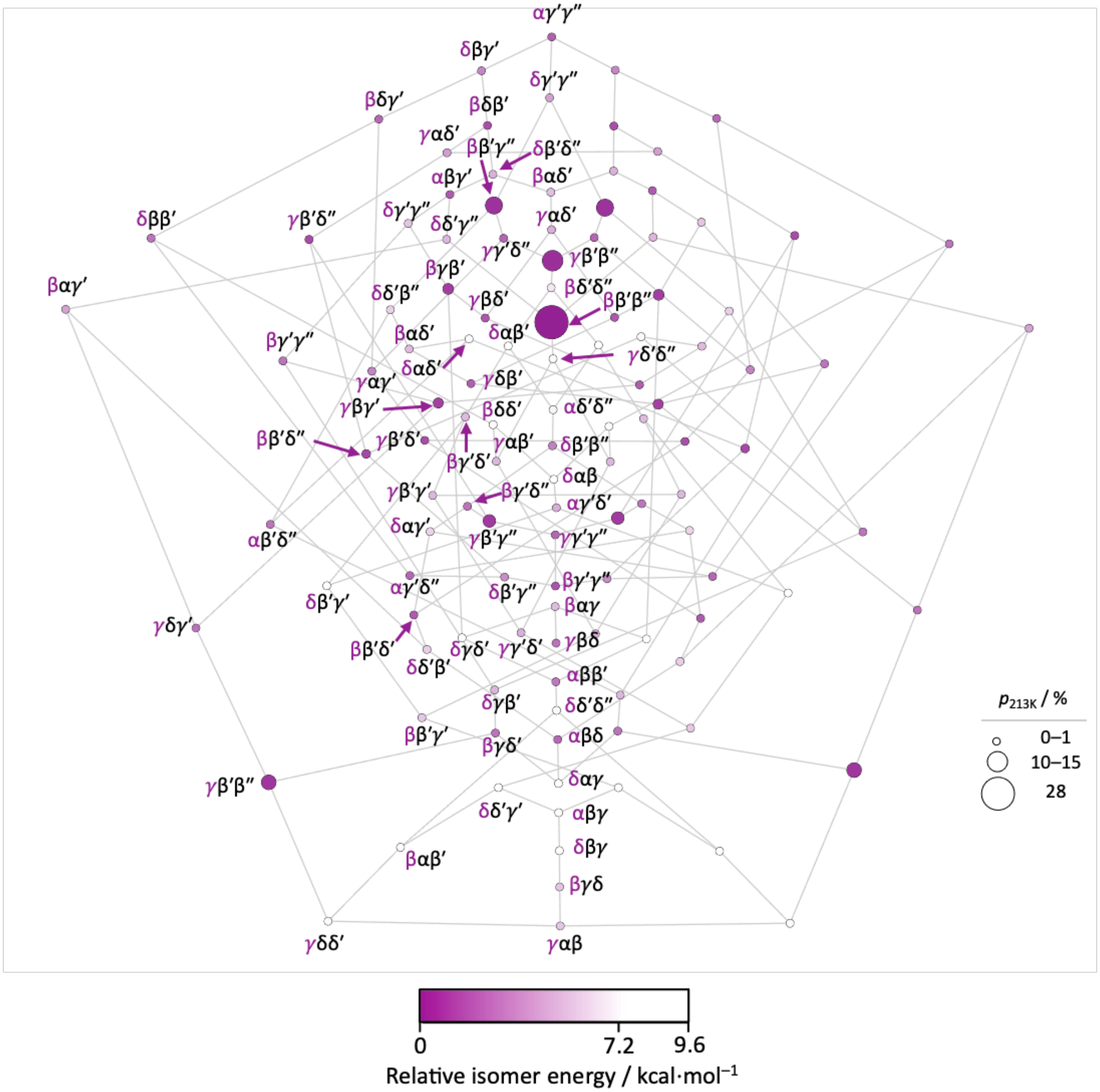

Figure S107: Thermodynamic reaction network of **F-Bull-MeOMe**, showing populations of isomers at 213 K.

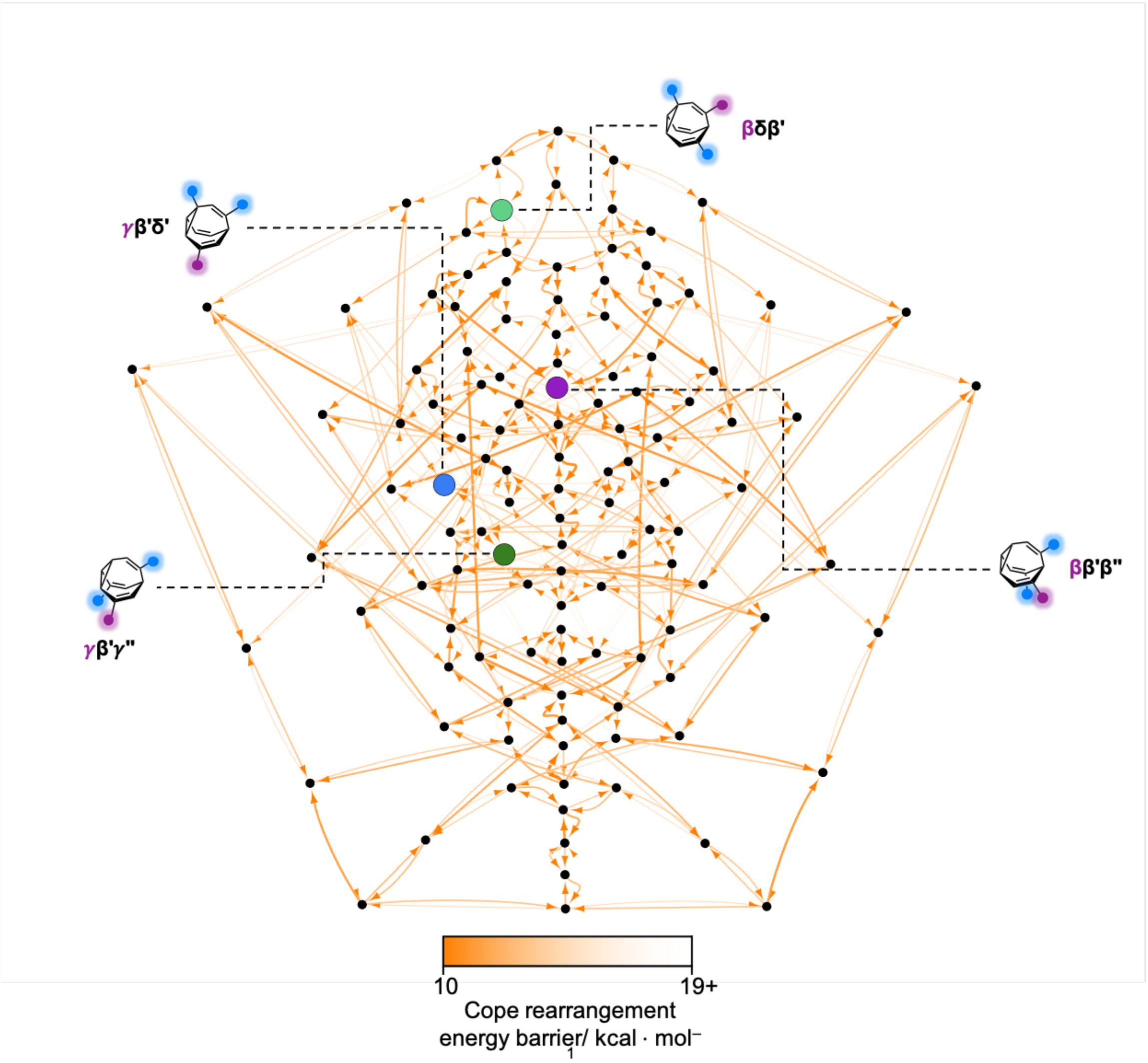

Figure S108: Kinetic reaction network of **F-Bull-MeOMe** at 213 K, showing **βδβ'**, ***γ*β'*γ*''**, ***γ*β'δ'** and **ββ'β''** isomers of **F-Bull-MeOMe**.

5.2: ISOMER STRETCHING AND COMPRESSING CALCULATIONS

To model the effect of a mechanical force on the potential energy of **F-Bull-MeOMe** isomers, ten conformers were generated for each constitutional isomer using *bullviso*[14] (i.e., 120 isomers × 10 conformers = 1200 structures) and minimised using the MMFF94 force field. A series of relaxed potential energy surface scans were performed starting from these geometries using the DFT conditions at the PBE0-D3 level of theory (i.e., with the PBE0[23,25,31] density functional of Adamo and Barone coupled with the D3[26] dispersion correction of Grimme *et al.*) using Gaussian 16.[27] The distances between the two C atoms attached to the bullvalene core were compressed (30 steps of 0.05 A increments = 1.5 Å in total) and expanded (50 steps of 0.05 Å increments = 2.5 Å in total), performing a geometry optimisation at each step.

The data (120 isomers × 10 conformers × 80 steps = 96000 optimised structures) was then processed to extract the energy vs distance profiles. Duplicates arising from enantiomers were removed (leaving 72 isomers) and the lowest energy conformer at each point along the potential energy surface (PES) scan was extracted for each constitutional isomer. This analysis produced a series of 72 distance vs energy curves, one for each unique bullvalene isomer in the network (24 meso compounds + 48 enantiomeric compounds). The resulting curves were manually checked and pruned to remove any errors, e.g., points in the expansion/compression where the covalent structure changed, for instance as a result of bond cleavage, and the resulting curves are plotted in **Figures S109** - **Figure S126**.

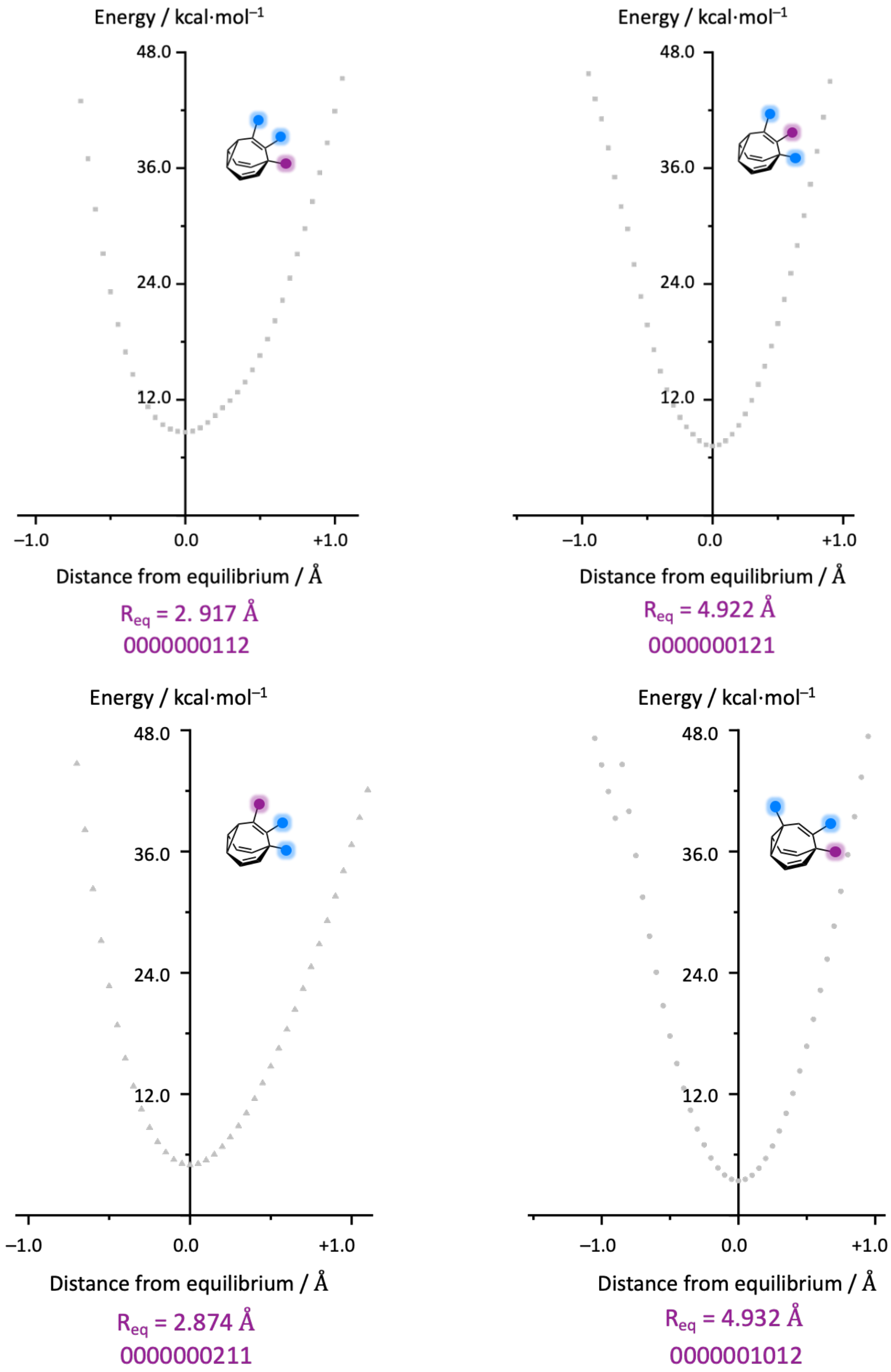

Figure S109: Potential energy surfaces of **F-Bull-MeOMe** isomers 0000000112, 0000000121, 0000000221 and 0000001012.

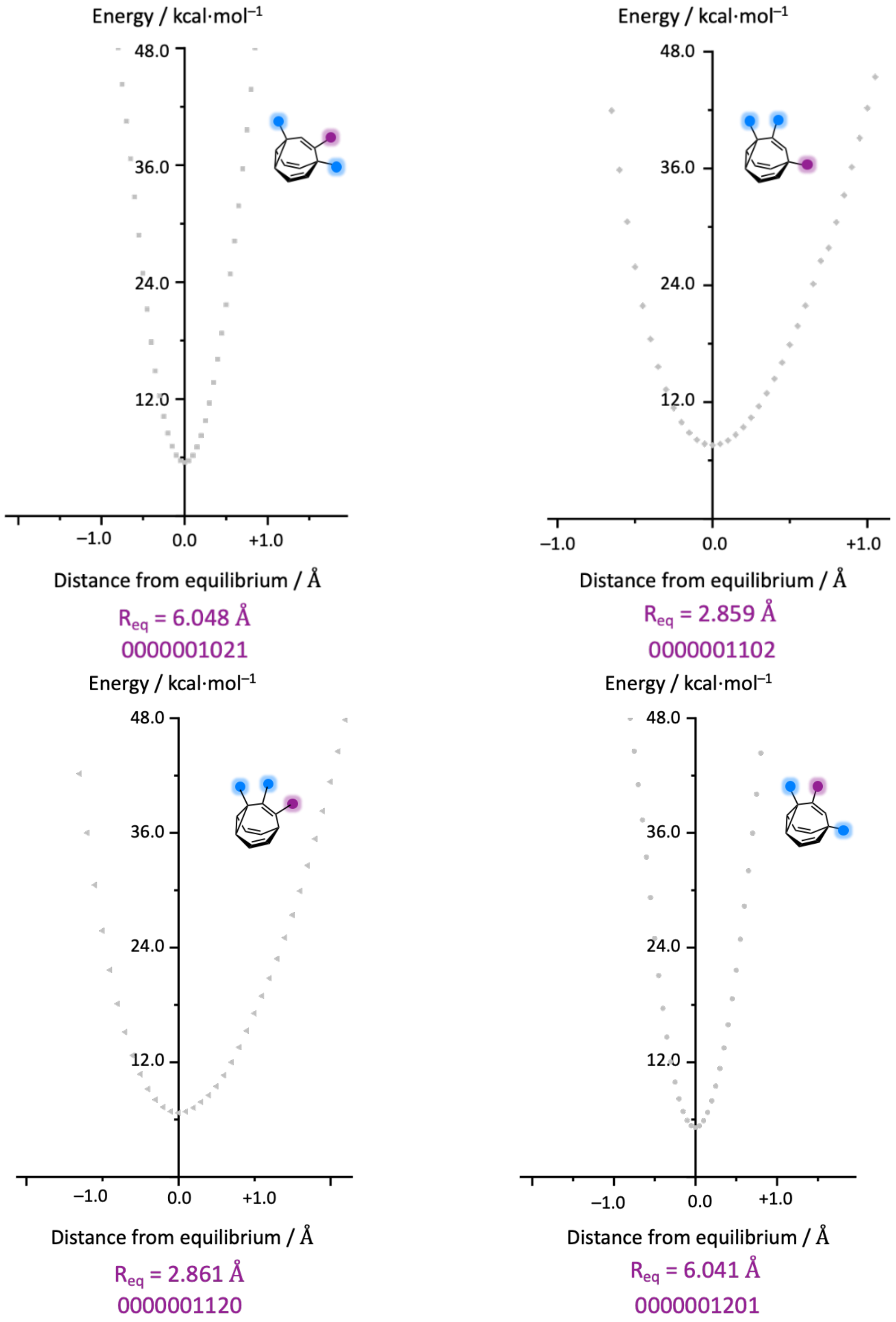

Figure S110: Potential energy surfaces of **F-Bull-MeOMe** isomers 0000001021, 0000001102, 00000001120 and 0000001201.

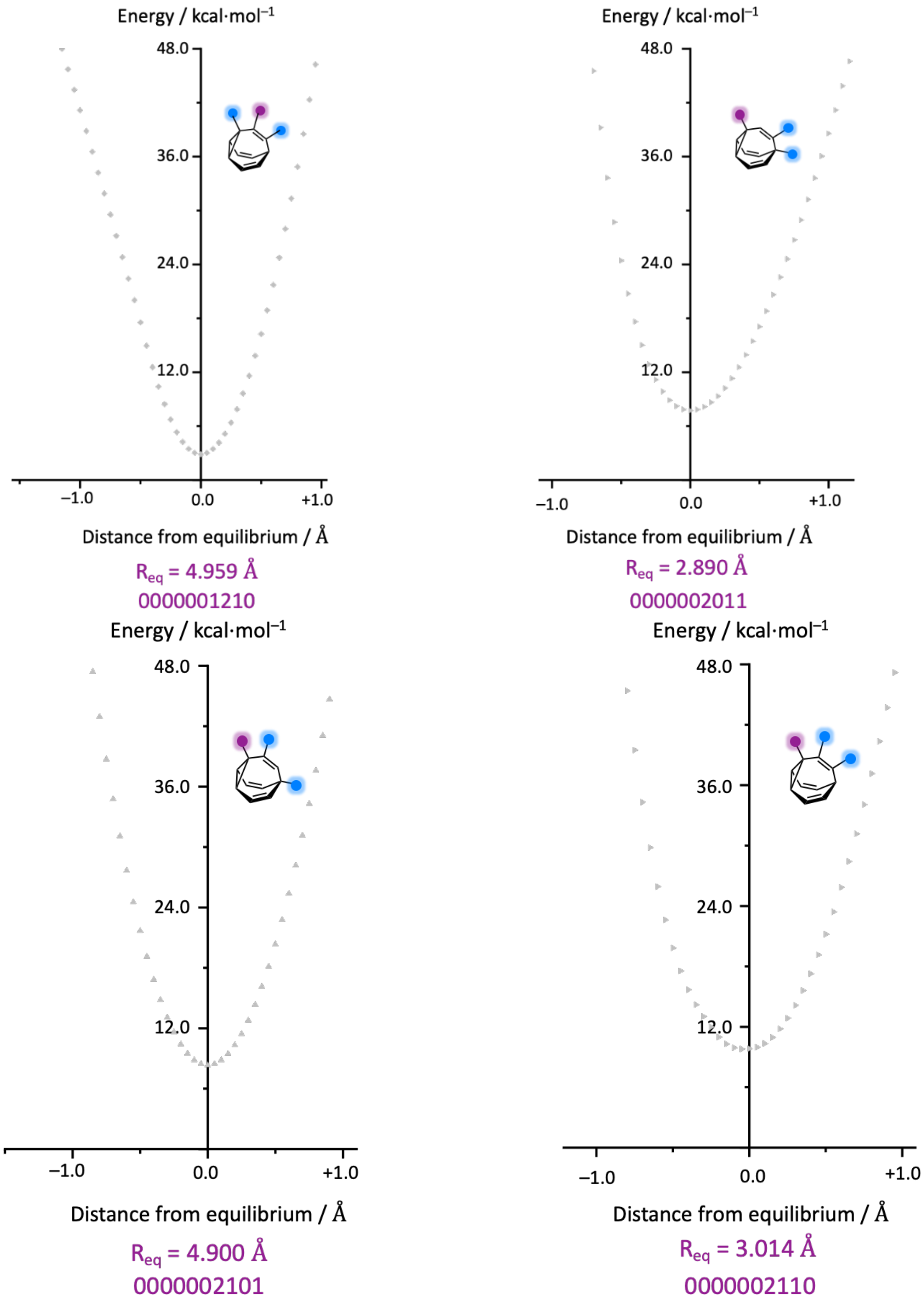

Figure S111: Potential energy surfaces of **F-Bull-MeOMe** isomers 0000001210, 0000002011, 00000002101 and 0000002110.

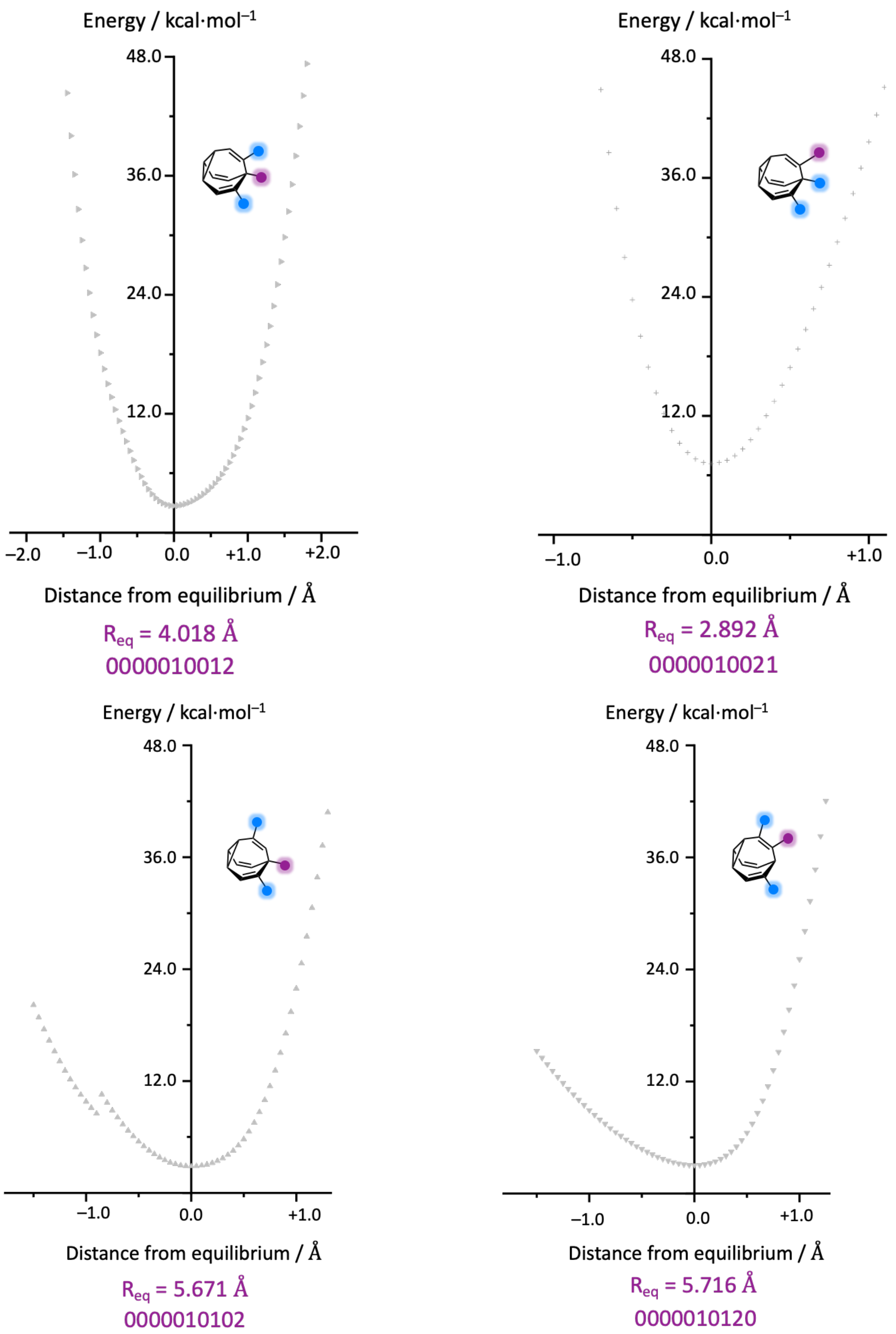

Figure S112: Potential energy surfaces of **F-Bull-MeOMe** isomers 0000010012, 0000010021, 00000010102 and 0000010120.

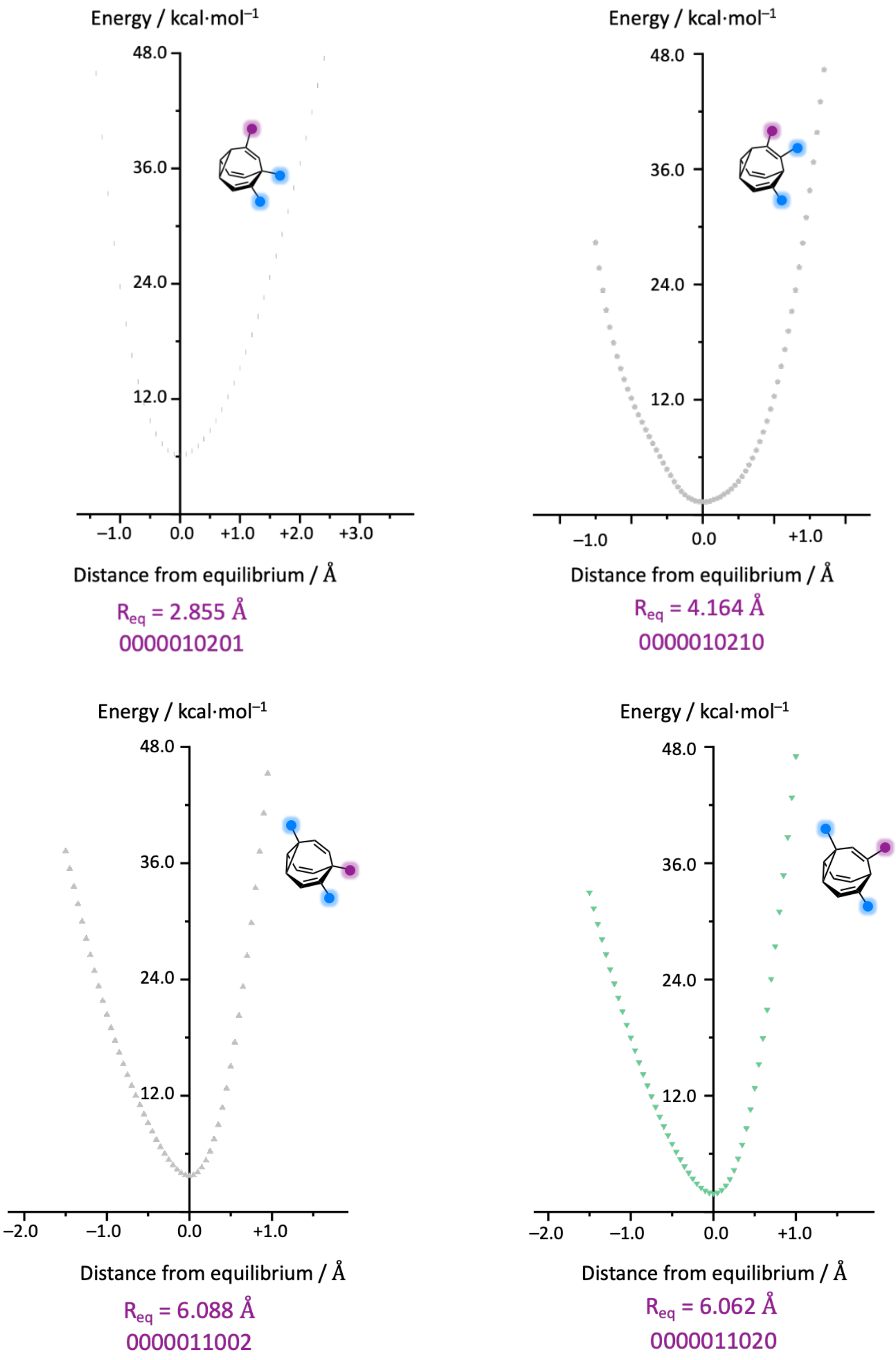

Figure S113: Potential energy surfaces of **F-Bull-MeOMe** isomers 0000010201, 0000010210, 0000011002 and 0000011020.

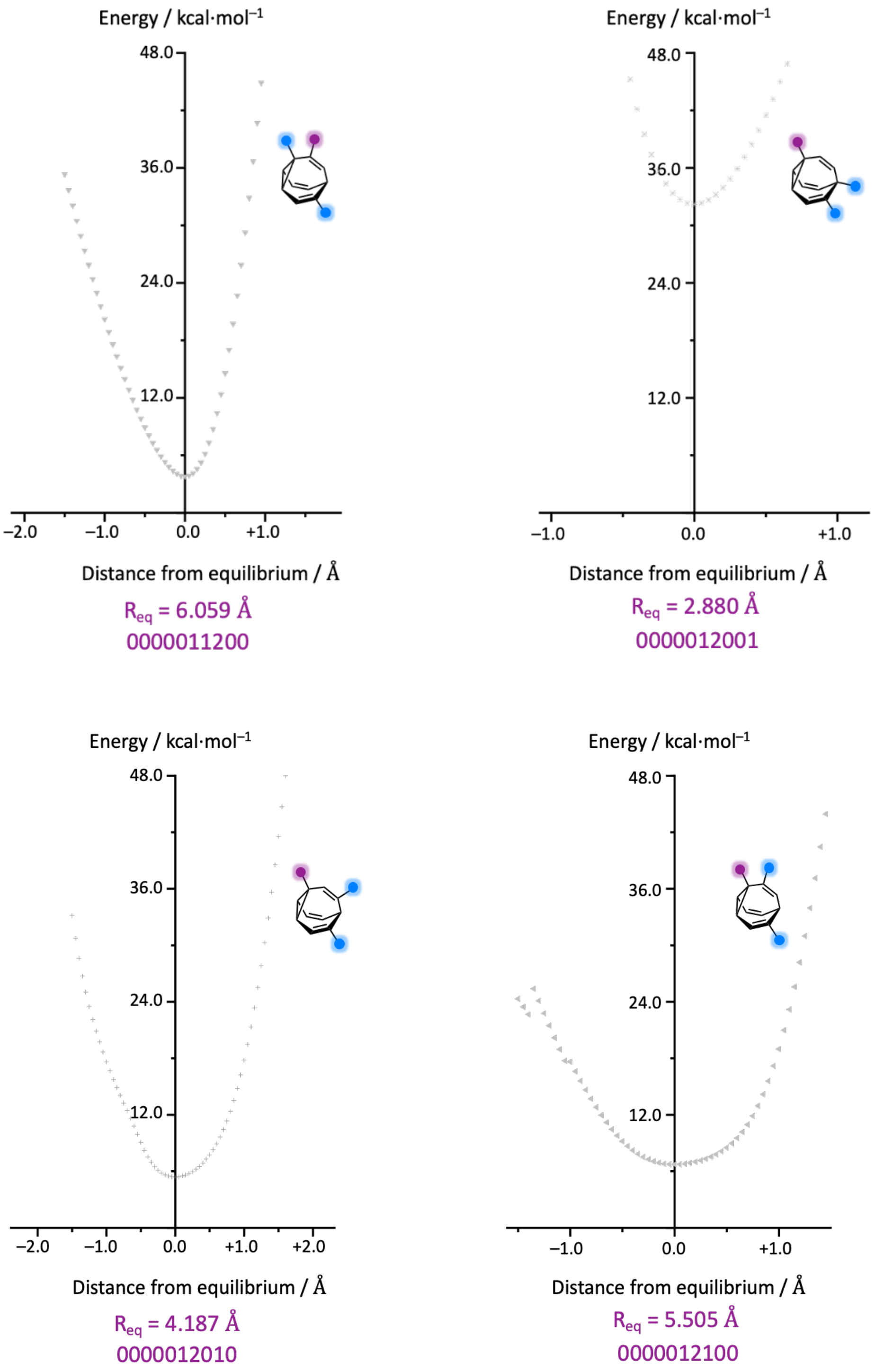

Figure S114: Potential energy surfaces of **F-Bull-MeOMe** isomers 0000011200, 0000012001, 0000012010 and 0000012100.

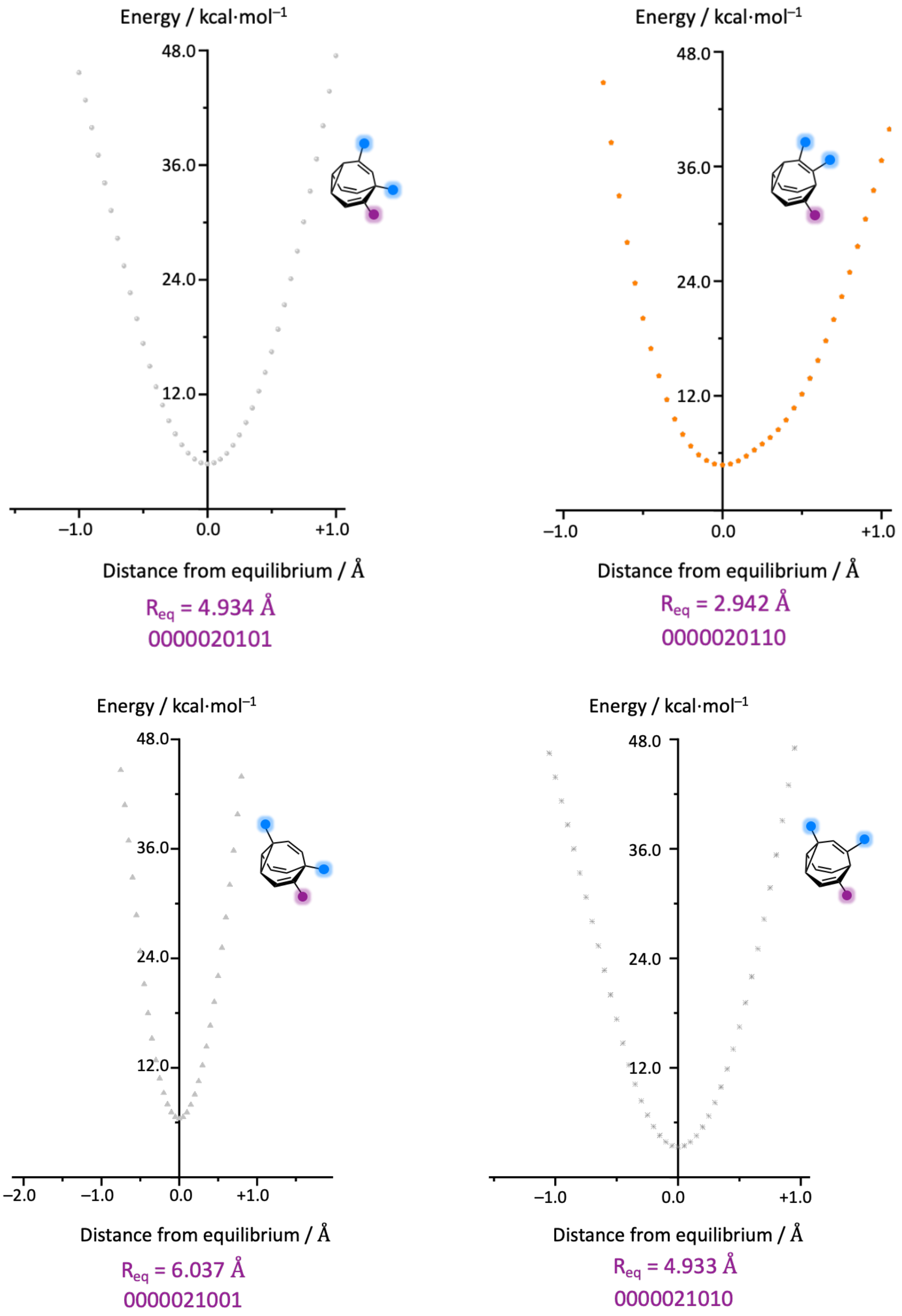

Figure S115: Potential energy surfaces of **F-Bull-MeOMe** isomers 0000020101, 0000020110, 0000021001 and 0000021010.

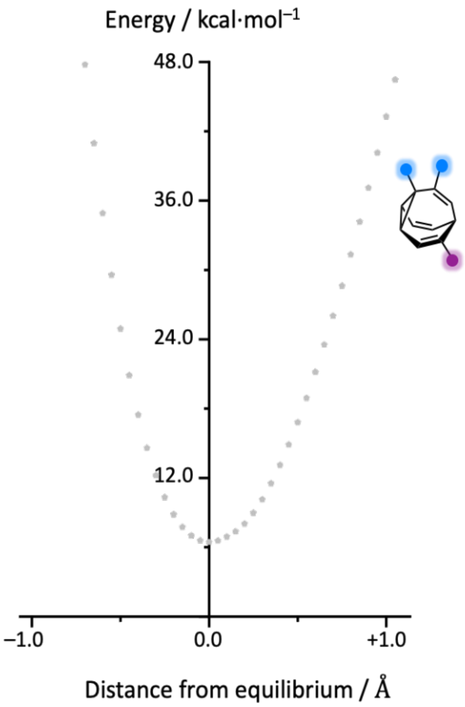

R_{eq} = 2.866 Å
0000021100

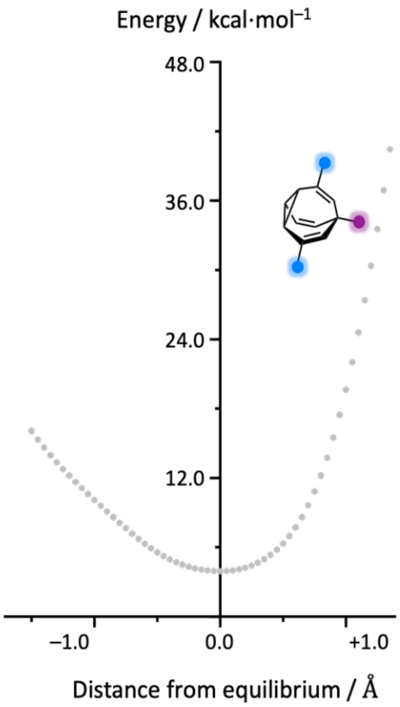

R_{eq} = 5.611 Å
0000100102

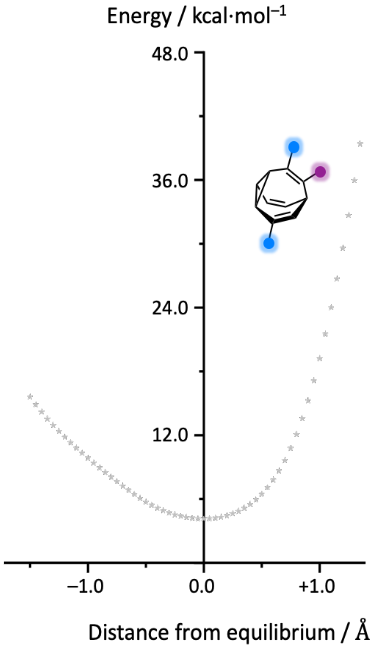

R_{eq} = 5.614 Å
00000100120

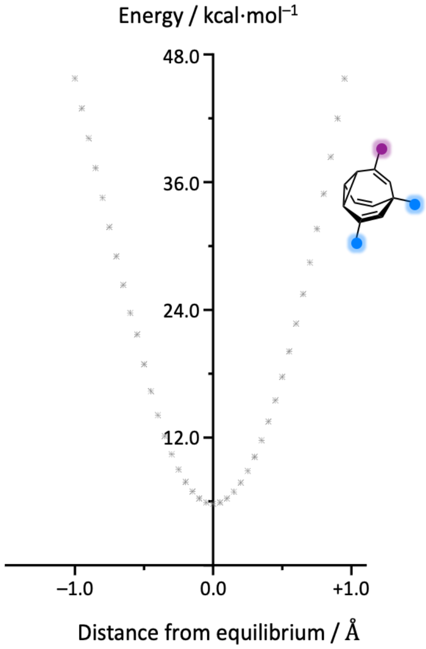

R_{eq} = 4.898 Å
0000100201

Figure S116: Potential energy surfaces of **F-Bull-MeOMe** isomers 0000021100, 0000100102, 00000100120, and 0000100201.

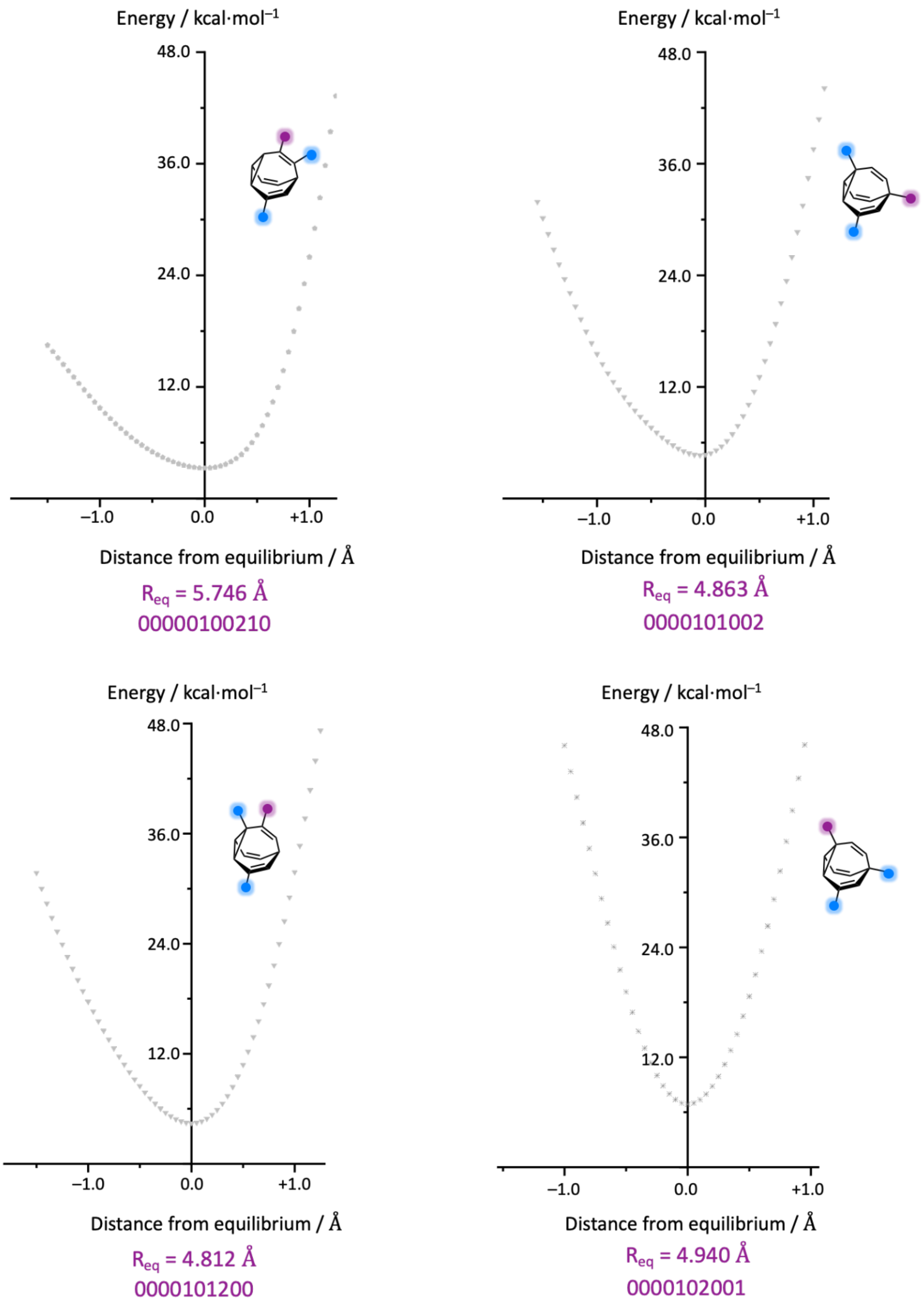

Figure S117: Potential energy surfaces of **F-Bull-MeOMe** isomers 0000100210, 0000101002, 00000101200 and 0000102001.

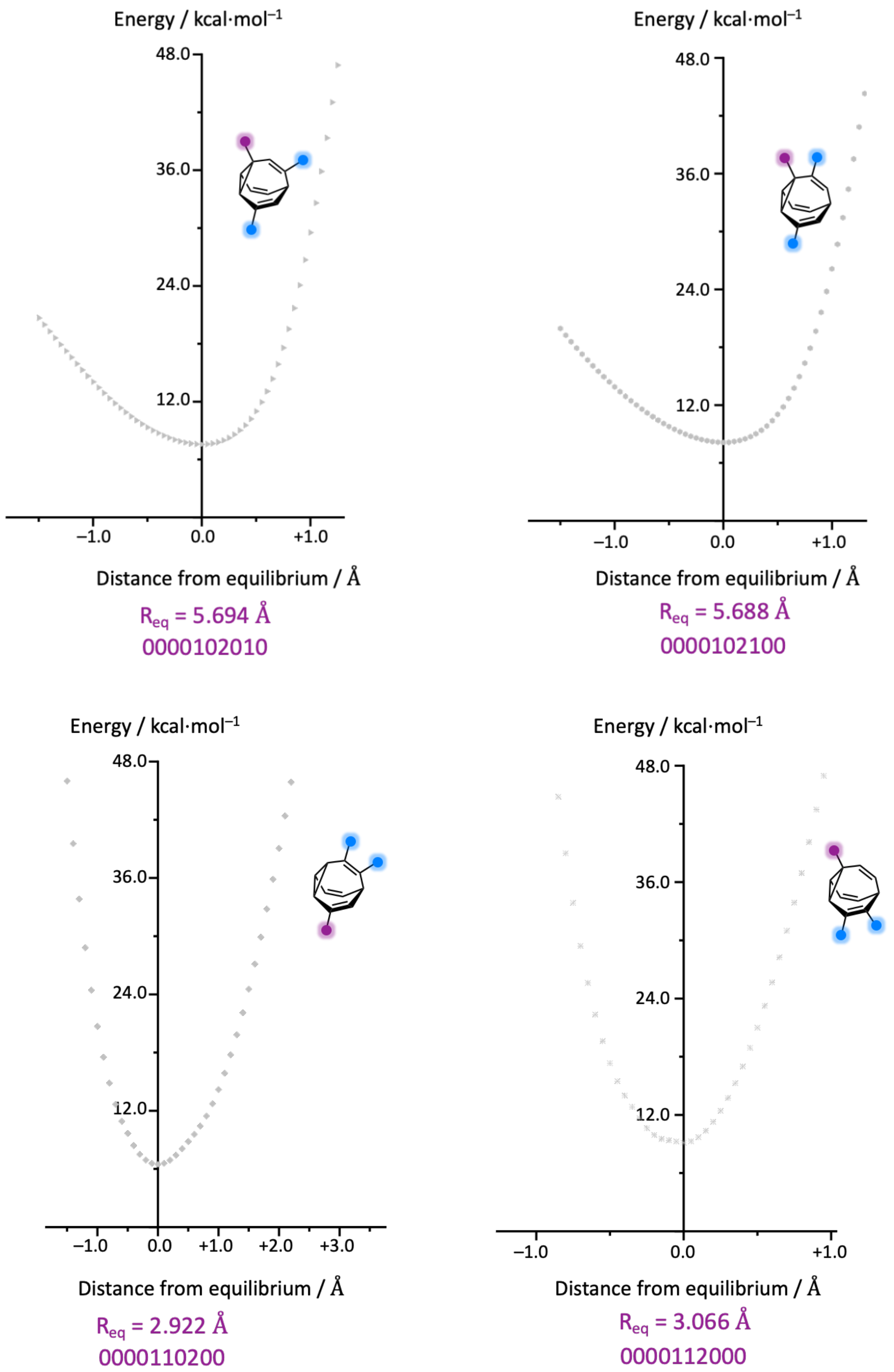

Figure S118: Potential energy surfaces of **F-Bull-MeOMe** isomers 0000102010, 0000102100, 0000110200 and 0000112000.

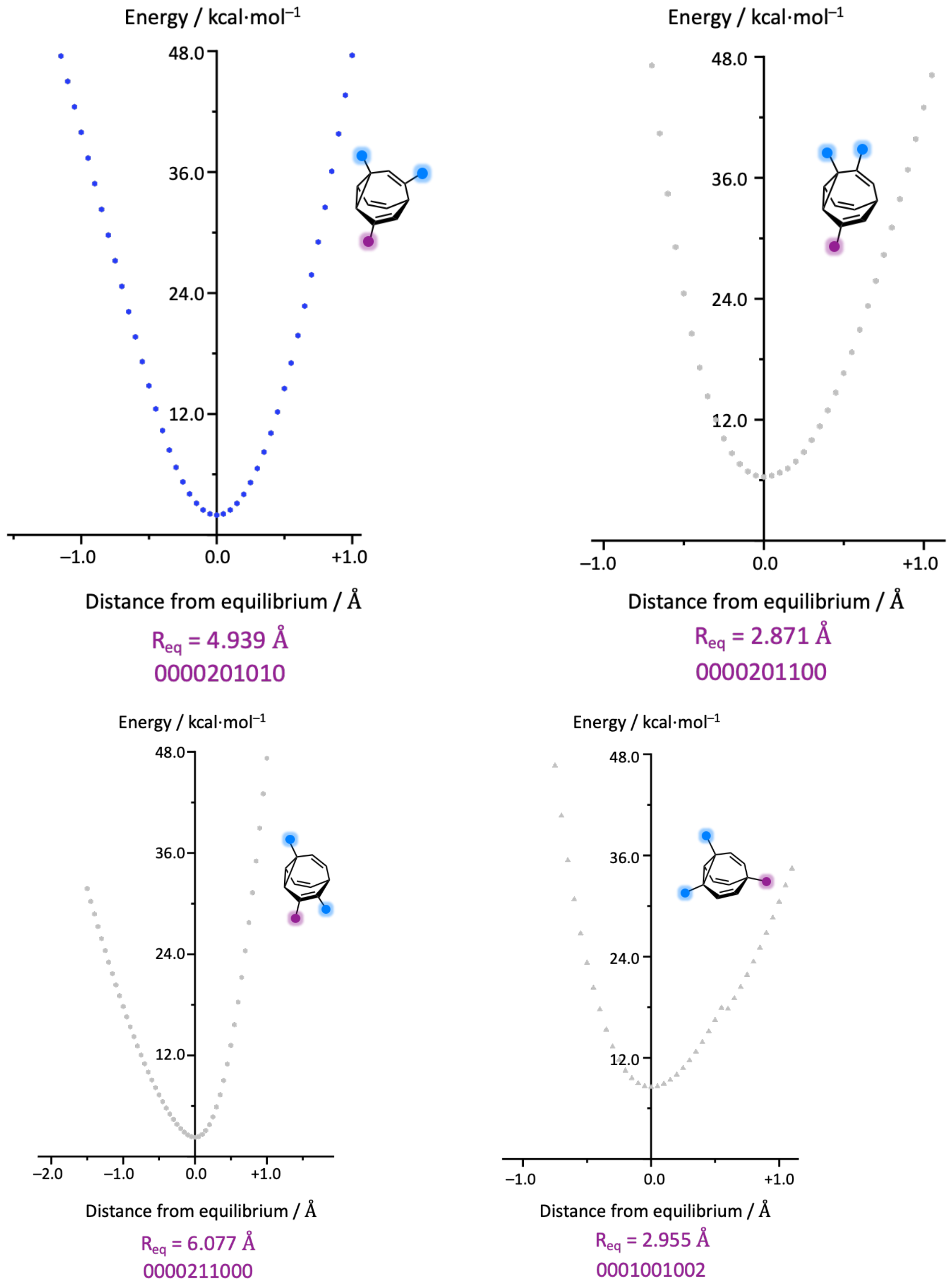

Figure S119: Potential energy surfaces of **F-Bull-MeOMe** isomers 0000201010, 0000201100, 0000211000 and 0001001002.

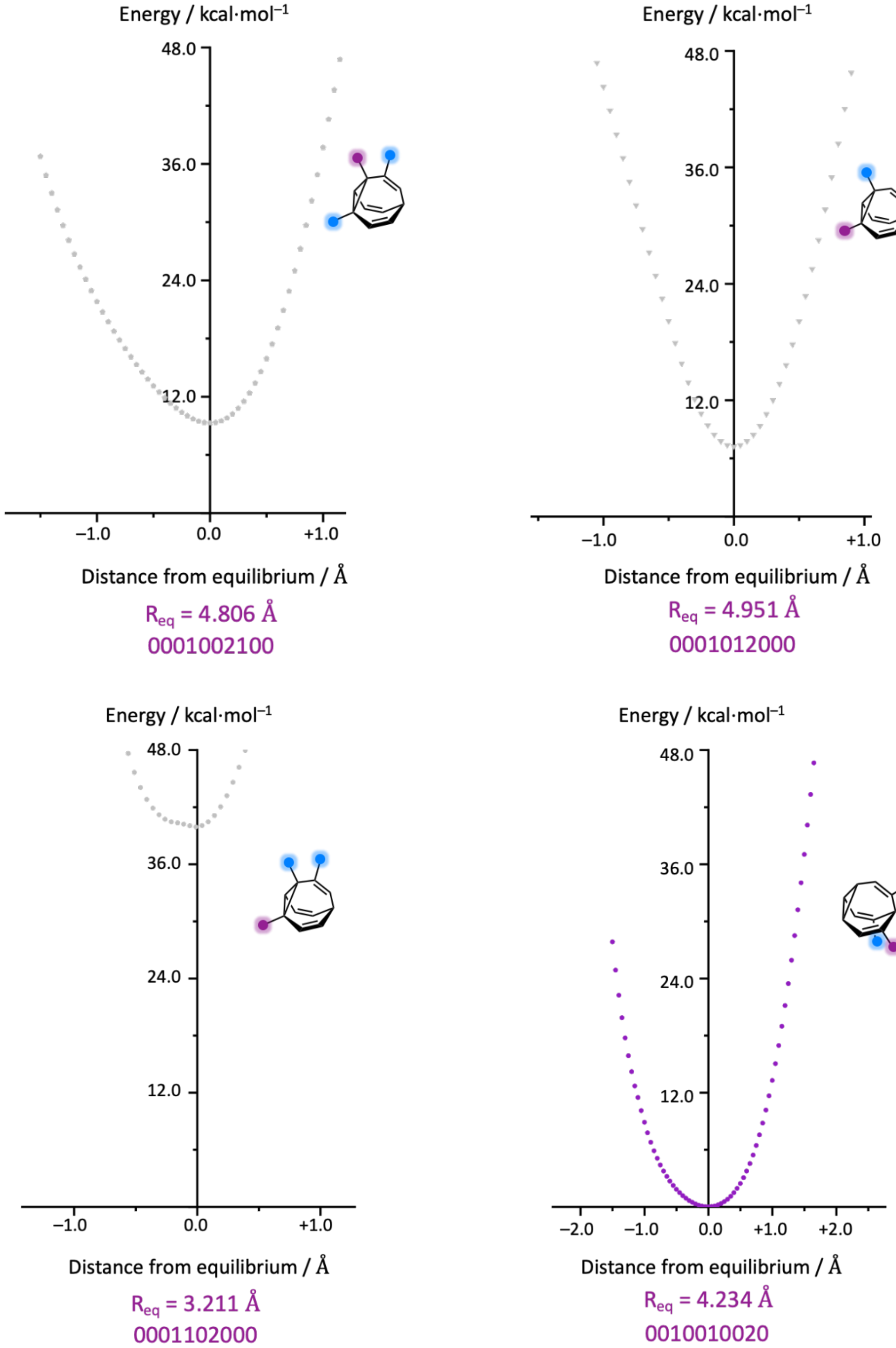

Figure S120: Potential energy surfaces of **F-Bull-MeOMe** isomers 0001002100, 0001012000, 0001102000, and 010010020.

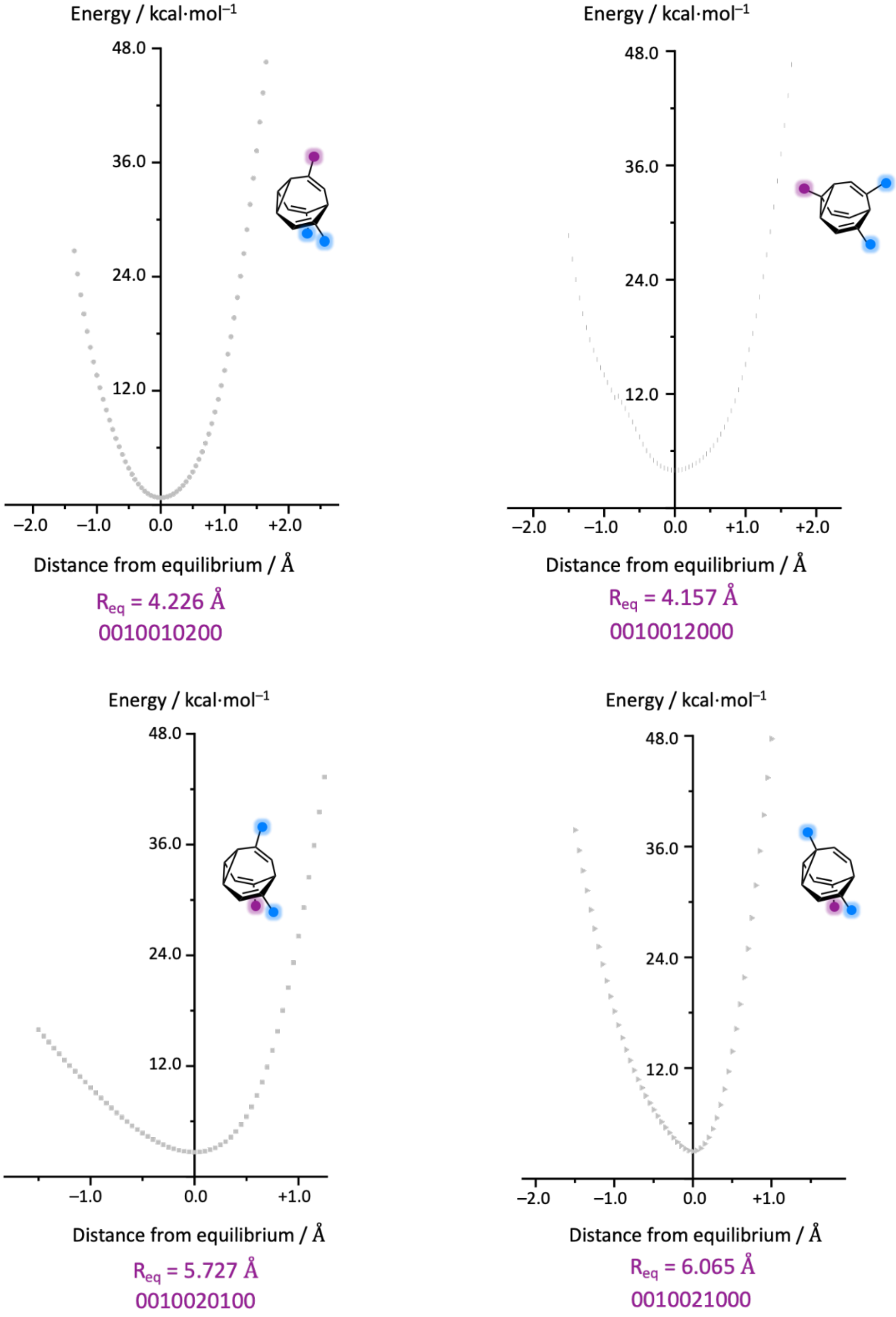

Figure S121: Potential energy surfaces of **F-Bull-MeOMe** isomers 0010010200, 0010012000, 0010020100 and 0010021000.

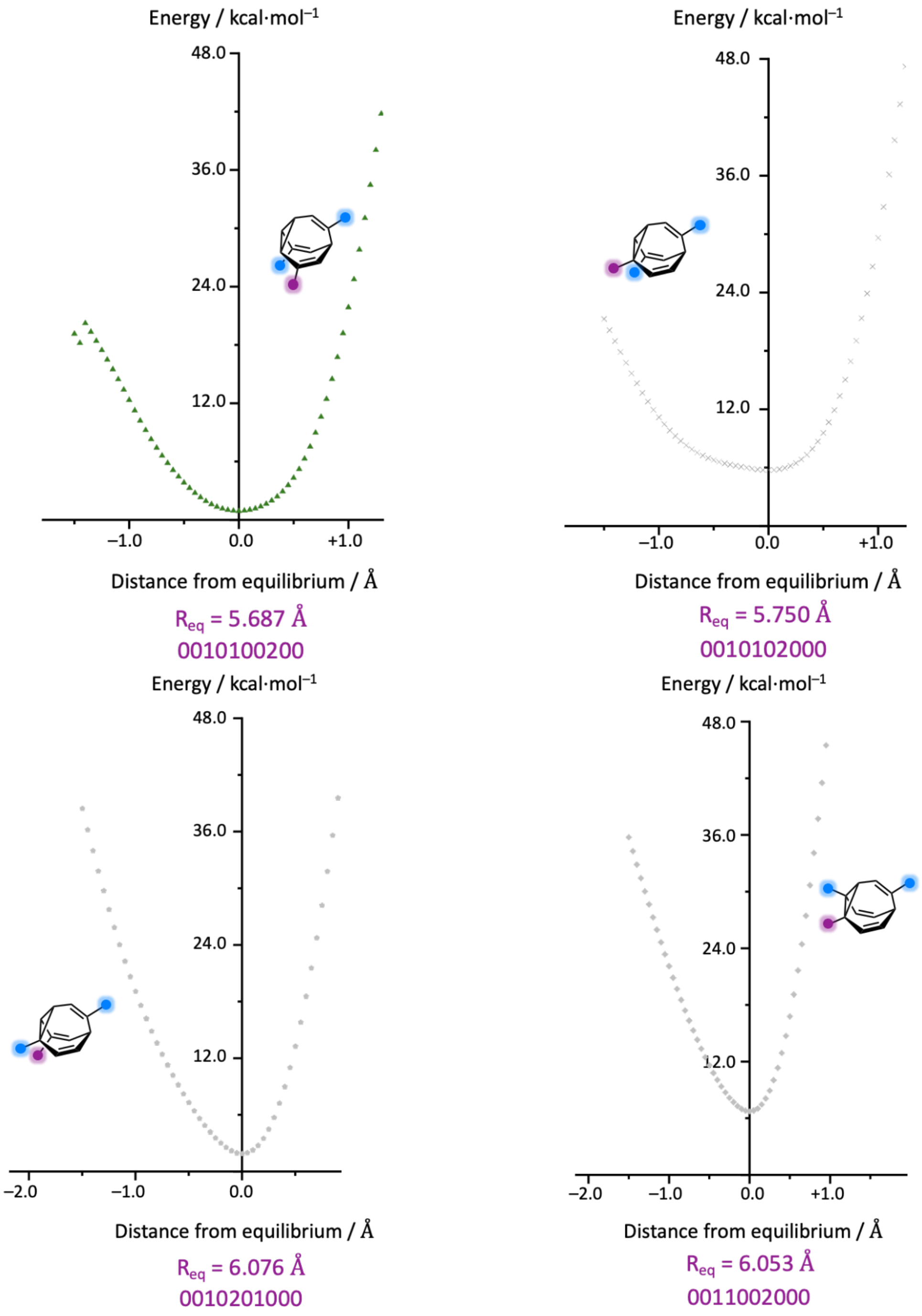

Figure S122: Potential energy surfaces of **F-Bull-MeOMe** isomers 0010100200, 0010102000, 0010201000 and 0011002000.

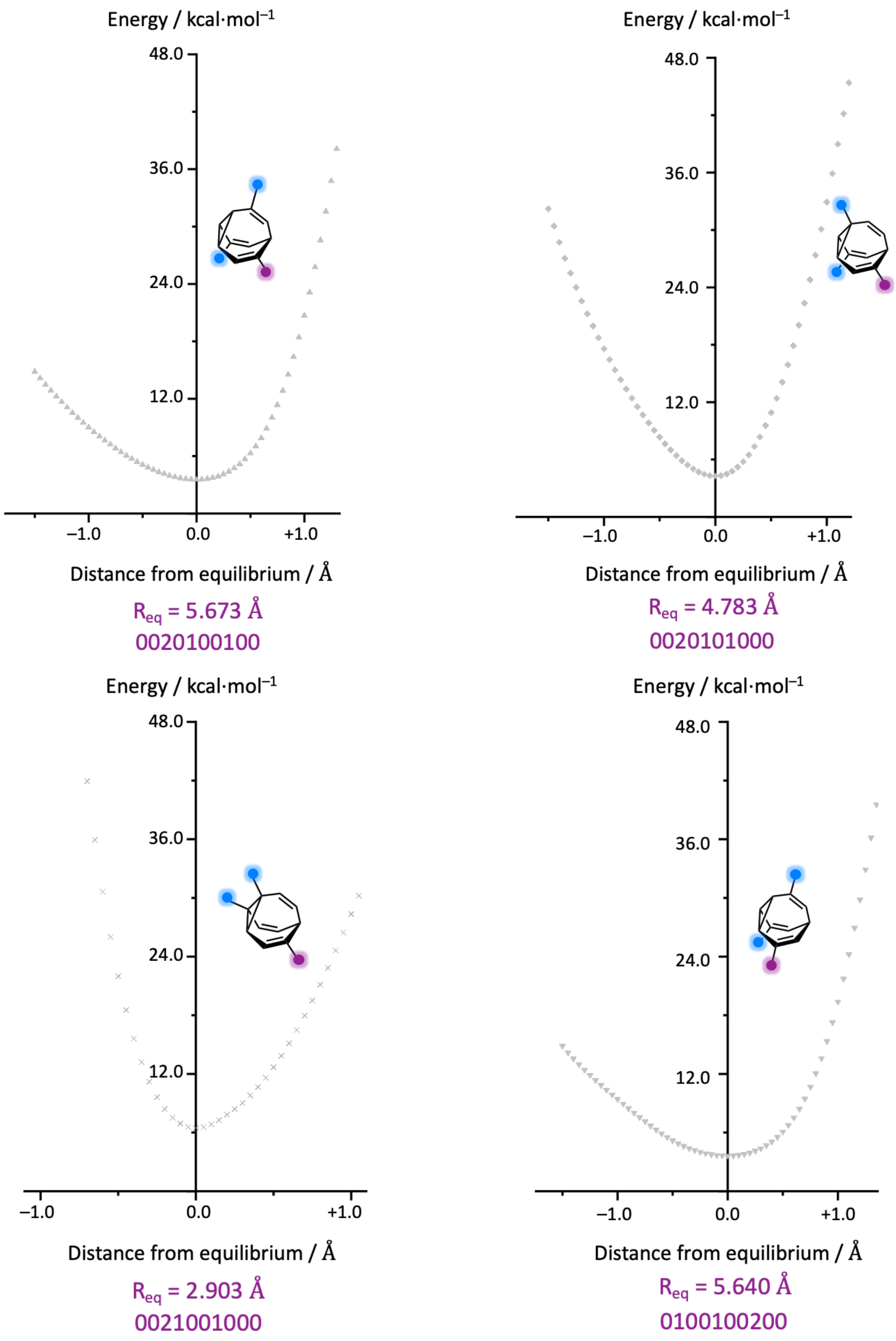

Figure S123: Potential energy surfaces of **F-Bull-MeOMe** isomers 0020100100, 002010100, 0021001000 and 0100100200.

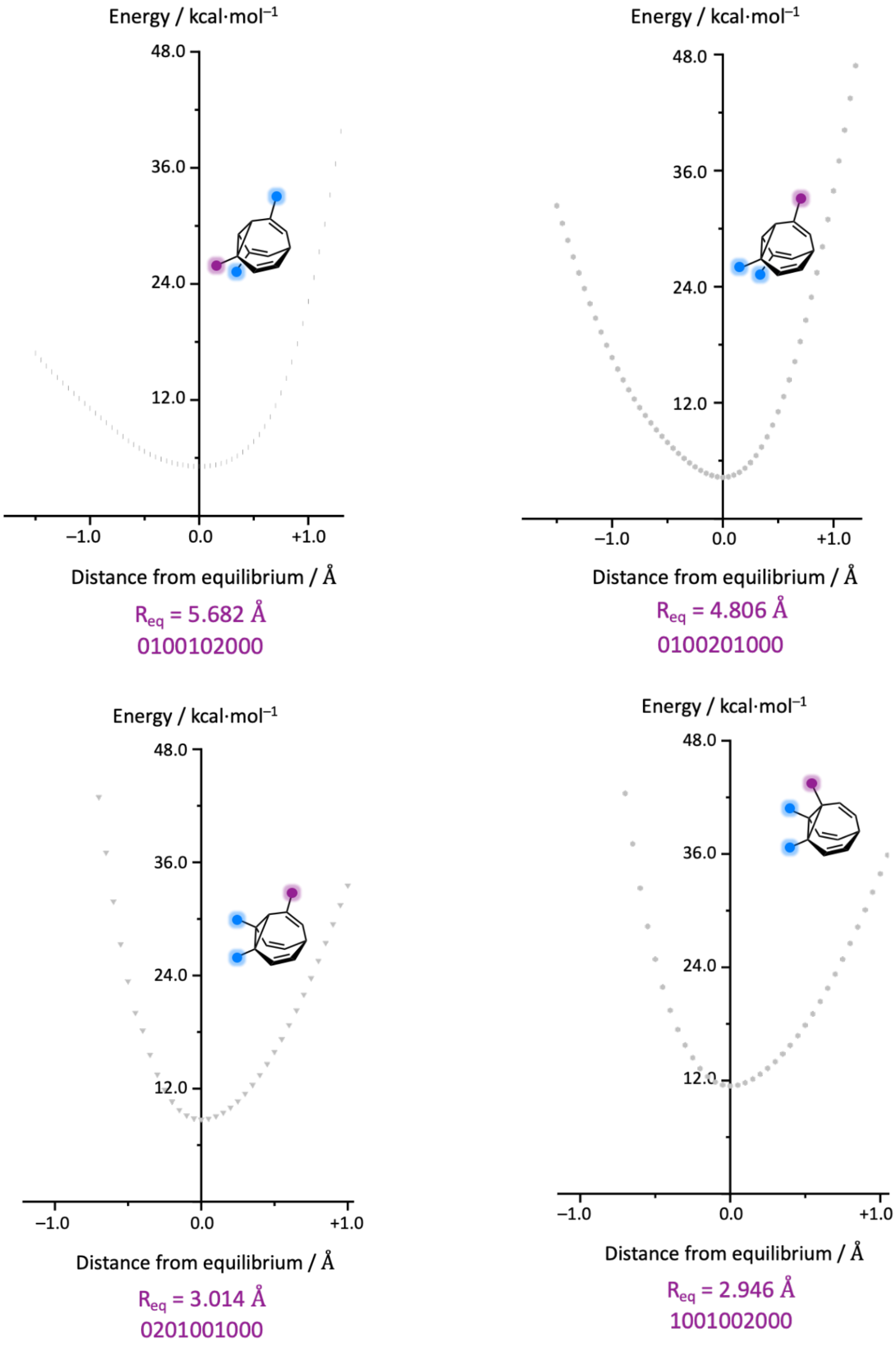

Figure S124: Potential energy surfaces of **F-Bull-MeOMe** isomers 0100102000, 0100201000 0201001000, and 1001002000.

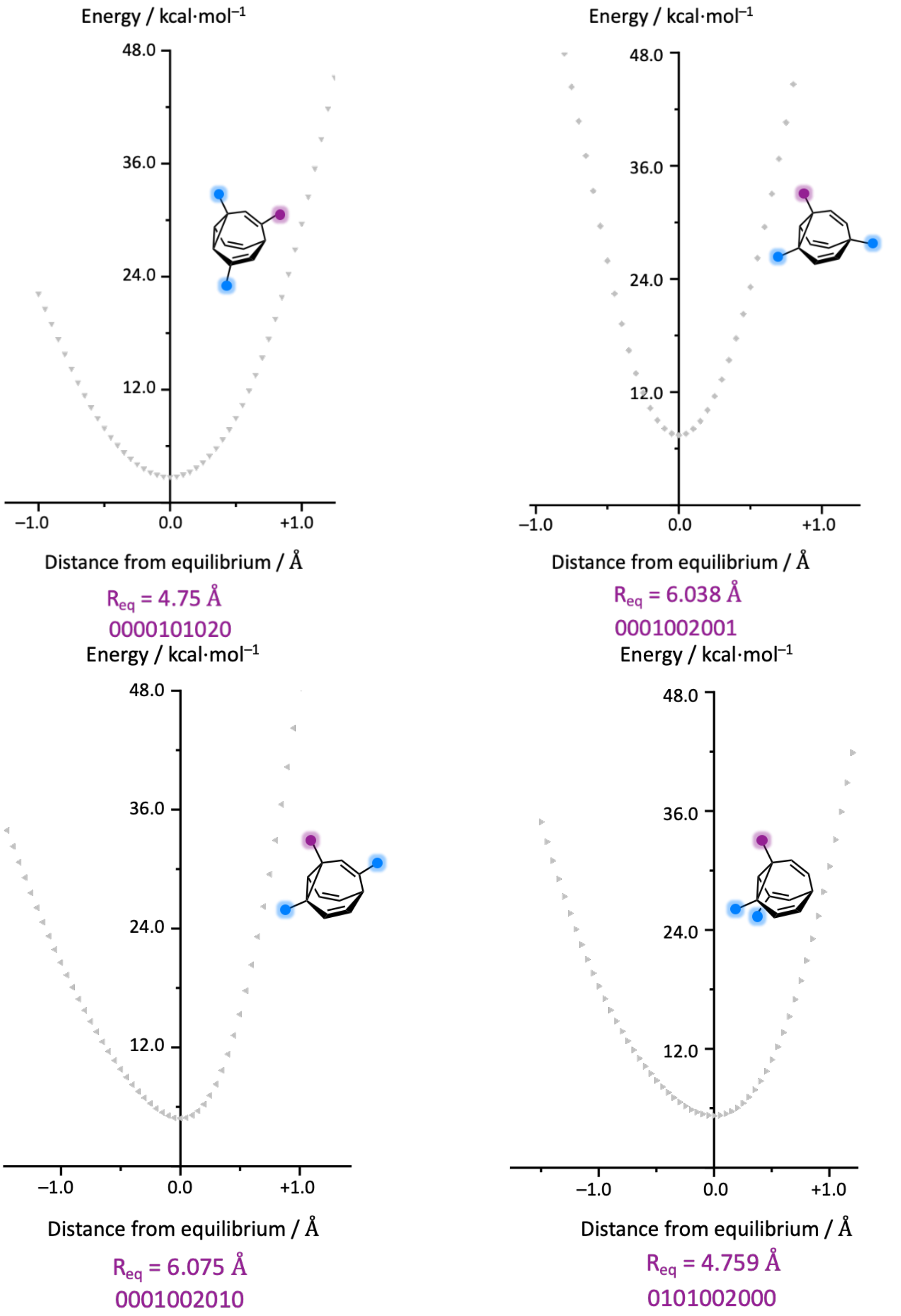

Figure S125: Potential energy surfaces of **F-Bull-MeOMe** isomers 0000101020, 0001002001, 0001002010, and 0101002000

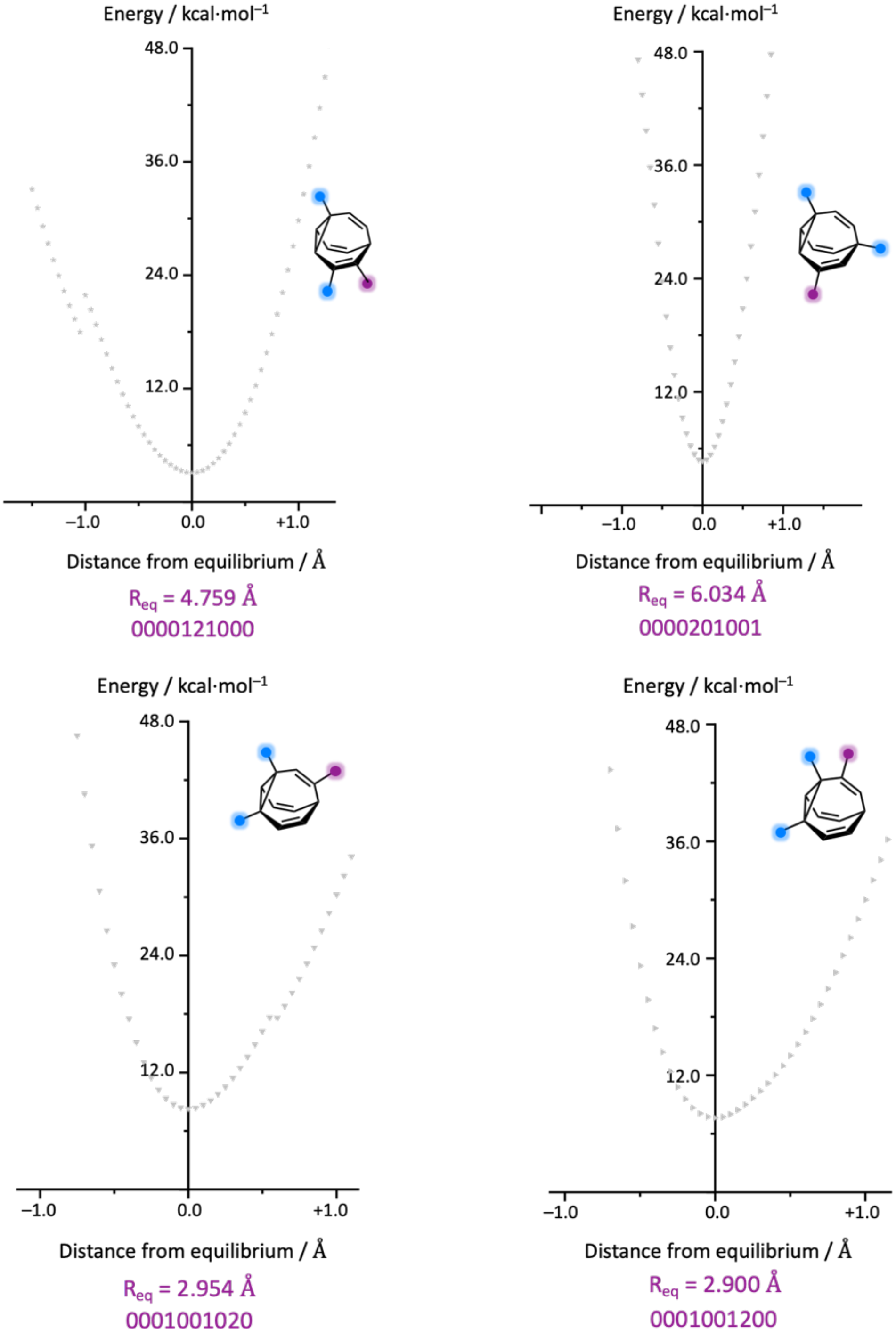

Figure S126: Potential energy surfaces of **F-Bull-MeOMe** isomers 0000121000, 0000201001, 0001001020, and 0001001200

5.3: KINETIC MODELLING

Stochastic kinetic simulations of **F-Bull-MeOMe** bullvalene network were performed using the Kinetiscope software package (http://hinsberg.net/kinetiscope/) at 213 K[32]. The simulation box contained 100,000 particles. The following mechanism parameters where used:

Table S6: Kinetic parameters for bullvalene **F-Bull-MeOMe** based on the energies from single point ground and transition state DFT calculations obtained at the PBE0-D3/def2-SV(P) at 298.15 K.

Source isomer barcode	Target isomer barcode	k_f at 213 K / s^{-1}	k_b at 213 K / s^{-1}
0000001120	0000000211	0.00385455	0.00416645
0000001120	0000002110	1.27E-07	0.00070618
0000001201	0000001021	0.02949023	0.09901312
0000001210	0000000121	6.09E-05	0.01501015
0000001210	0000001210	1.31E-05	1.31E-05
0000002011	0000001102	0.32424126	0.00033695
0000002101	0000001012	69.0811711	0.00059735
0000002110	0000000112	2.52478253	0.04829582
0000002110	0000001120	0.00070618	1.27E-07
0000012100	0000011002	0.02599232	0.00015635
0000021001	0000011200	0.05243422	4.15E-05
0000021100	0000012001	1.47E-05	0.01656166
0000101002	0000012010	0.0019851	0.00582287
0000101200	0000020101	8.72E-05	0.00377613
0000102001	0000021010	0.05785402	4.98E-06
0000102010	0000100102	0.00058177	3.10E-05
0000102100	0000010102	0.070847	1.95E-05
0000110200	0000021100	0.00016605	0.00021095
0000112000	0000001102	0.01998554	1.68E-05
0000112000	0000021100	0.00833125	1.86E-05
0000112000	0000020110	0.01238398	7.88E-05
0000120010	0000102100	3.06E-07	0.00913858
0000120100	0000012100	9.06E-06	0.00145202
0000120100	0000102100	1.72E-07	8.21E-05
0000120100	0000100210	0.0053633	0.00010305
0000121000	0000002101	4.26E-07	0.02125665
0000121000	0000012100	1.39E-06	0.00024539
0000121000	0000010210	0.01392733	0.00012749
0000200101	0000101020	0.0018338	0.00018376

0000200110	0000110200	0.00018055	0.00018055
0000201001	0000011020	0.08229169	0.0001862
0000201010	0000100201	4.34E-06	0.00031727
0000201100	0000010201	1.17E-05	2.25E-05
0000201100	0000110200	2.91E-07	5.72E-07
0000201100	0000110020	0.00287801	0.02046055
0000210010	0000101200	1.09E-05	0.00105588
0000210100	0000011200	1.92E-05	0.00017558
0000210100	0000101200	8.13E-07	3.43E-05
0000210100	0000100120	7.07E-05	0.00367765
0000211000	0000001201	3.57E-06	0.00295074
0000211000	0000011200	9.30E-06	1.58E-05
0000211000	0000010120	0.00018295	2.81E-05
0001000102	0000102001	5.08E-06	0.02394087
0001000120	0000210010	0.05676032	0.00051959
0001000120	0000002101	4.26E-07	0.02125665
0001000201	0000201001	6.58E-05	6.58E-05
0001000210	0000120010	0.0004814	7.39E-05
0001000210	0000001201	3.58E-06	0.00295508
0001001002	0000012001	0.03445586	0.50219356
0001001020	0000200011	0.21119858	0.00122286
0001001020	0000002011	0.0013592	0.00371019
0001001200	0000020011	0.00054219	0.00057667
0001001200	0000210010	0.59981864	1.68E-07
0001001200	0000000211	0.00109697	1.82E-05
0001002001	0001000012	0.49632985	1.90E-05
0001002001	0000021001	0.00216153	1.91E-05
0001002010	0000100012	0.07579678	9.72E-05
0001002010	0000001021	6.62E-05	0.00018702
0001002100	0000010012	1.90740809	1.84E-05
0001002100	0000120010	0.71642176	8.02E-08
0001002100	0000000121	0.00049866	8.10E-07
0001010020	0000201010	0.00022142	8.12E-05
0001010200	0000021010	0.00027435	0.00074779
0001010200	0000201010	3.58E-06	3.58E-06
0001010200	0000200101	4.16E-06	0.0003036
0001012000	0000001012	0.00532407	2.17E-06
0001012000	0000021010	0.01056818	1.51E-06
0001012000	0000020101	0.00312921	0.00011403

0001020010	0000102010	3.61E-06	0.00024828
0001020010	0001002010	8.51E-06	0.01580159
0001020010	0001000201	0.0001862	0.08229169
0001020100	0000012010	8.26E-05	0.0002224
0001020100	0000102010	1.95E-06	2.00E-05
0001020100	0000100201	0.00041202	0.00411176
0001021000	0000002011	0.00032768	0.00089448
0001021000	0000012010	0.1208942	4.27E-06
0001021000	0000010201	0.02696404	0.00015612
0001100020	0000200110	0.00321301	0.00252921
0001100200	0000020110	0.00287379	0.02043054
0001100200	0000200110	2.92E-07	5.73E-07
0001100200	0000200011	6.26E-07	1.20E-06
0001102000	0000000112	0.00171404	0.00021344
0001102000	0000020110	0.00485373	7.56E-06
0001102000	0000020011	8.68E-05	7.06E-06
0001200010	0000100210	0.00017532	1.91E-05
0001200010	0001000210	4.50E-06	2.66E-06
0001200010	0001000021	0.0001368	0.17271922
0001200100	0000010210	0.00079887	8.22E-06
0001200100	0000100210	3.44E-05	8.16E-07
0001200100	0000100021	8.73E-05	0.00378168
0001201000	0000000211	0.00109697	1.82E-05
0001201000	0000010210	0.27669938	7.76E-08
0001201000	0000010021	0.00248869	0.00264697
0002000011	0001100020	0.01112541	9.90E-06
0002000011	0001001002	0.50145683	0.03440531
0002000101	0001010020	0.49342372	4.25E-05
0002000101	0000101002	0.27955763	5.93E-05
0002000110	0000110020	0.01690559	0.00010753
0002000110	0001100020	0.00011025	2.46E-07
0002000110	0000001102	0.05975312	5.03E-05
0002001001	0001000021	0.00216153	1.91E-05
0002001001	0000011002	0.49778933	1.90E-05
0002001010	0000100021	0.00043694	1.59E-05
0002001010	0001010020	0.04752214	6.80E-06
0002001010	0000001012	0.08486829	3.46E-05
0002001100	0000010021	8.65E-05	7.04E-06
0002001100	0000110020	0.00485373	7.56E-06

0002001100	0000000112	0.0041787	0.00052035
0002010010	0000101020	1.72E-05	6.40E-06
0002010010	0001001020	5.38E-05	1.52367316
0002010010	0001000102	0.00166201	0.0005666
0002010100	0000011020	0.00018295	2.66E-06
0002010100	0000101020	3.98E-06	3.89E-07
0002010100	0000100102	0.01222143	0.00065045
0002011000	0000001021	6.63E-05	0.0001873
0002011000	0000011020	0.01582481	8.52E-06
0002011000	0000010102	0.07360357	9.44E-05
0002100010	0000100120	0.00209283	1.31E-05
0002100010	0001000120	0.00021533	1.22E-06
0002100010	0001000012	0.08752556	0.0005265
0002100100	0000010120	0.00615695	2.06E-07
0002100100	0000100120	0.00020394	4.28E-07
0002100100	0000100012	0.05129215	1.41E-05
0002101000	0000000121	0.00126671	2.06E-06
0002101000	0000010120	0.70287816	7.87E-08
0002101000	0000010012	3.99752085	3.86E-05
0010012000	0001001002	0.00182039	11.0247268
0010021000	0002001001	1.47E-06	1.13765535
0010102000	0000101002	0.00204425	0.00010184
0010201000	0000201001	4.04E-07	0.00023759
0010201000	0010200100	2.17E-05	5.97E-06
0010201000	0010021000	0.0001229	9.69E-05
0011000020	0010102000	2.90E-06	0.00060708
0011000020	0001002001	1.47E-06	1.13598637
0011000200	0011000020	1.77E-05	1.40E-05
0011000200	0010100200	4.35E-06	1.20E-06
0011000200	0001000201	8.54E-07	0.00050234
0011002000	0000011002	0.01940725	1.69E-05
0011002000	0010100020	1.94416149	1.14E-05
0011002000	0001000021	0.00027516	5.52E-05
0012000100	0010021000	0.000126	6.02E-07
0012000100	0001000102	0.00204125	0.00010169
0012001000	0000021001	0.00028628	5.74E-05
0012001000	0010020100	4.41720031	2.59E-05
0012001000	0001000012	0.01943576	1.69E-05
0020100100	0010102000	8.85E-06	0.00074451

0020100100	0012000100	8.52E-06	0.00071662
0020101000	0000102001	5.41E-06	0.00878342
0020101000	0010100200	0.00132375	1.53E-05
0020101000	0010012000	0.0003795	0.0010405
0021000100	0010012000	0.00042119	0.00115481
0021000100	0010200100	0.00138539	1.60E-05
0021000100	0002000101	5.41E-06	0.00878342
0021001000	0000012001	0.00056163	0.02031091
0021001000	0010010200	4.23930318	3.18E-06
0021001000	0002000011	1.71E-05	0.00061877
0100100200	0020100100	6.87E-05	1.82E-05
0100100200	0010200100	0.00012164	4.52E-06
0100100200	0010100200	0.00012182	4.53E-06
0100102000	0000100102	0.00071348	5.26E-06
0100102000	0010020100	0.00355554	8.45E-07
0100102000	0010100020	0.00023654	5.62E-08
0100201000	0000200101	8.20E-06	0.00010627
0100201000	0010010200	1.48E-05	2.57E-07
0100201000	0010020100	0.00073798	1.68E-05
0101000200	0010100020	0.00039951	9.11E-06
0101000200	0010010200	0.00017353	3.01E-06
0101000200	0000100201	8.22E-06	0.00010659
0101002000	0000010102	0.01981027	1.69E-05
0101002000	0010010020	0.97799939	1.10E-05
0101002000	0000100021	7.51E-06	1.72E-06
0102001000	0000020101	7.52E-06	1.72E-06
0102001000	0010010020	0.55736575	6.29E-06
0102001000	0000100012	0.01972321	1.68E-05
0201001000	0000010201	8.60E-05	5.55E-07
0201001000	0010010020	136.121862	9.30E-06
0201001000	0000200011	8.59E-05	5.54E-07
1001002000	0000010012	0.98087524	1.72E-07
1001002000	0000020011	0.01155829	4.64E-05
1001002000	0000010021	0.03606023	0.00014486

Kinetic simulations were run starting from 100% populations of isomers **γβ'δ'** (0001010200), **γβ'γ"** (0010200100), or **βδβ'** (0000011020).

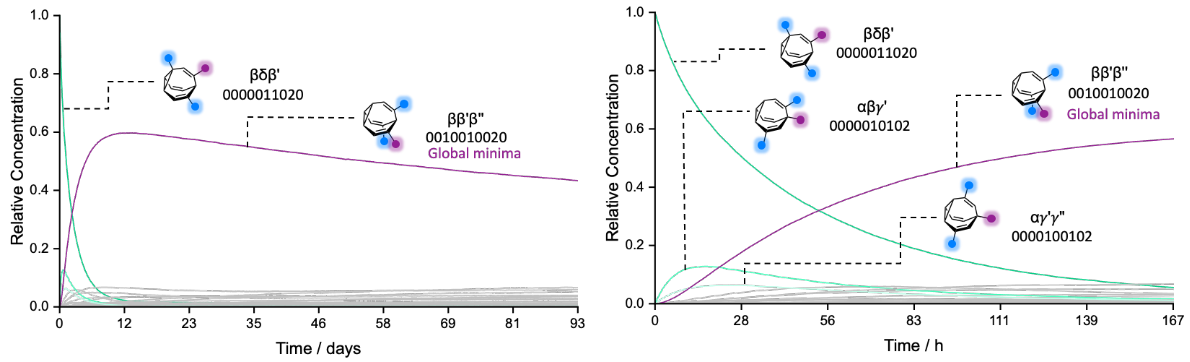

Figure S127. Kinetic simulation starting from 100% of **βδβ'** (0000011020).

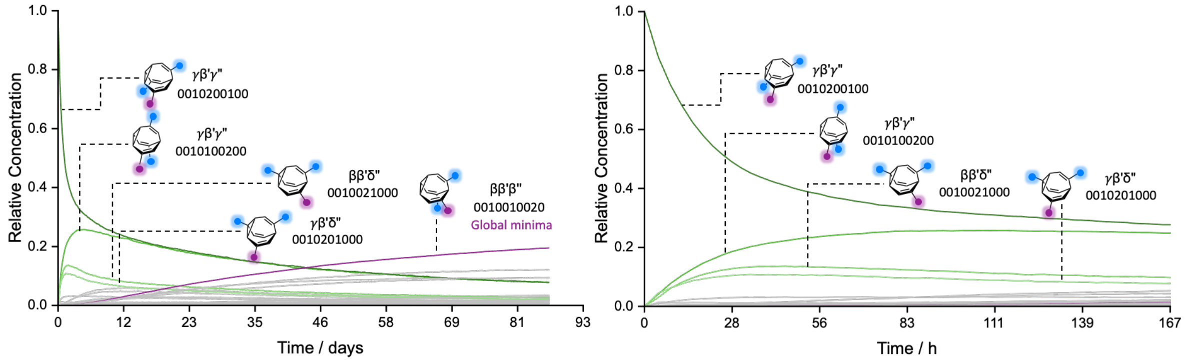

Figure S128. Kinetic simulation starting from 100% of **γβ'γ"** (0010200100).

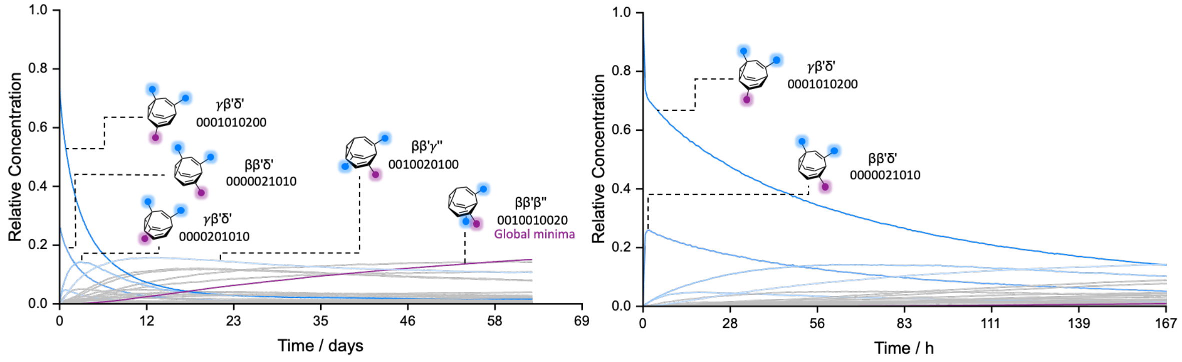

Figure S129. Kinetic simulation starting from 100% of **γβ'δ'** (0001010200).

5.4: PYTHON SCRIPT

To process output data from the **F-Bull-MeOMe** bullvalene DFT calculations the following python script was developed. First, barcode labels are generated for all possible ground states[33], followed by extraction of single point energies from output .log files. The output file 'Processed_raw_GS_data.csv' contains information about each ground state calculation (1200 data points in total, 10 conformers for each of 120 isomers), indicating whether it was successfully terminated and how many negative frequencies were found, as well as the Hartree energy for each conformer. Unsuccessful calculations were filtered out from the analysis. The other output .csv file (filename specified by a user) contains refined data where only the lowest energy conformer for each isomer was kept (leaving 120 data points in total). The chirality of each barcode was determined and its enantiomer barcode specified (if the isomer is chiral). To ensure the symmetry of the network, if the energies of two enantiomers are not identical, the lowest energy was taken and applied to both. The relative energies of each of the 120 isomers were then tabulated relative to the lowest energy conformer of the lowest energy isomer as also specified in **Table S3**. XYZ coordinates of all successful DFT calculations are saved in 'XYZ Coordinates From Successful DFT Calcualtions.csv' file.

Similarly, to process results of transition state DFT calculations, the python code first generates barcode for all possible transition states[34]. Next, the code checks for successful termination of Gaussian and the presence of a single negative frequency. Occasionally a wrong transition state would be found (e.g., corresponding to the rotation of substituent rather than a Cope rearrangement). To filter such transition states from true Cope rearrangements, the displacement of all **F-Bull-MeOMe** atoms during the negative frequency vibration mode is calculated. If atoms showing the largest displacement do not belong to the **F-Bull-MeOMe** bullvalene core atoms (atoms 0, 3, 2, 5, 17, 18, 19, 20, 21, 22, 23), those transitions states are likely to correspond to a different transition state and those were filtered out. Raw transition state data can be found in 'Processed_raw_TS_data.csv' output file, where for each calculation (1800 in total, 10 conformers for each of 180 transition states) it is indicated whether the calculation was successfully completed and whether the transition state corresponds to a Cope rearrangement transition state. Following this initial processing, the data is further refined to keep only the lowest energy conformer for each transition state (giving 180 transition states in total). The results of these transition state energy calculations are given in **Table S5**.

To calculate energy barriers and rate constants for each of the Cope rearrangements used in kinetic analysis, each transition state barcode was linked to a pair of corresponding ground state barcodes (**Scheme S1**). Each ground state isomer can participate in three possible Cope rearrangements (one on each face of a bullvalene). First, for each of the three Cope rearrangements, each arm of the ground state isomer was labelled as a participating arm (P) or non-participating (NP) arm based on whether or not that arm is directly involved in the Cope rearrangement[35]. A transition state label for each Cope rearrangement was then generated[34]. Depending whether the core substituent is on the P or NP arm, its final position after each Cope rearrangement can be predicted, giving the barcode of the resulting ground state isomer. Each of the three resulting ground state barcodes labels was optimized again to its lowest number sequence. Transition state barcode labels are different depending on the direction of a Cope rearrangement (forward or backward). Conventionally, only the lowest number sequence transition state label was kept. Forward and backward Cope rearrangements between a pair of ground state isomers were treated as two separate edges and energy barriers in each direction were calculated separately giving a table of 360 edges. The energy barriers were then calculated in the forward direction relative to each starting isomer (Ground state 1, **Scheme S1**). Rate constants are calculated based on the energy barriers using Eyring equation at a user specified temperature. The edge data is saved to .csv file (filename specified by a user) giving **Table S6**. XYZ coordinates of all successful transition state DFT calculations are saved in 'XYZ Coordinates From Successful TS DFT Calcualtions.csv' file.

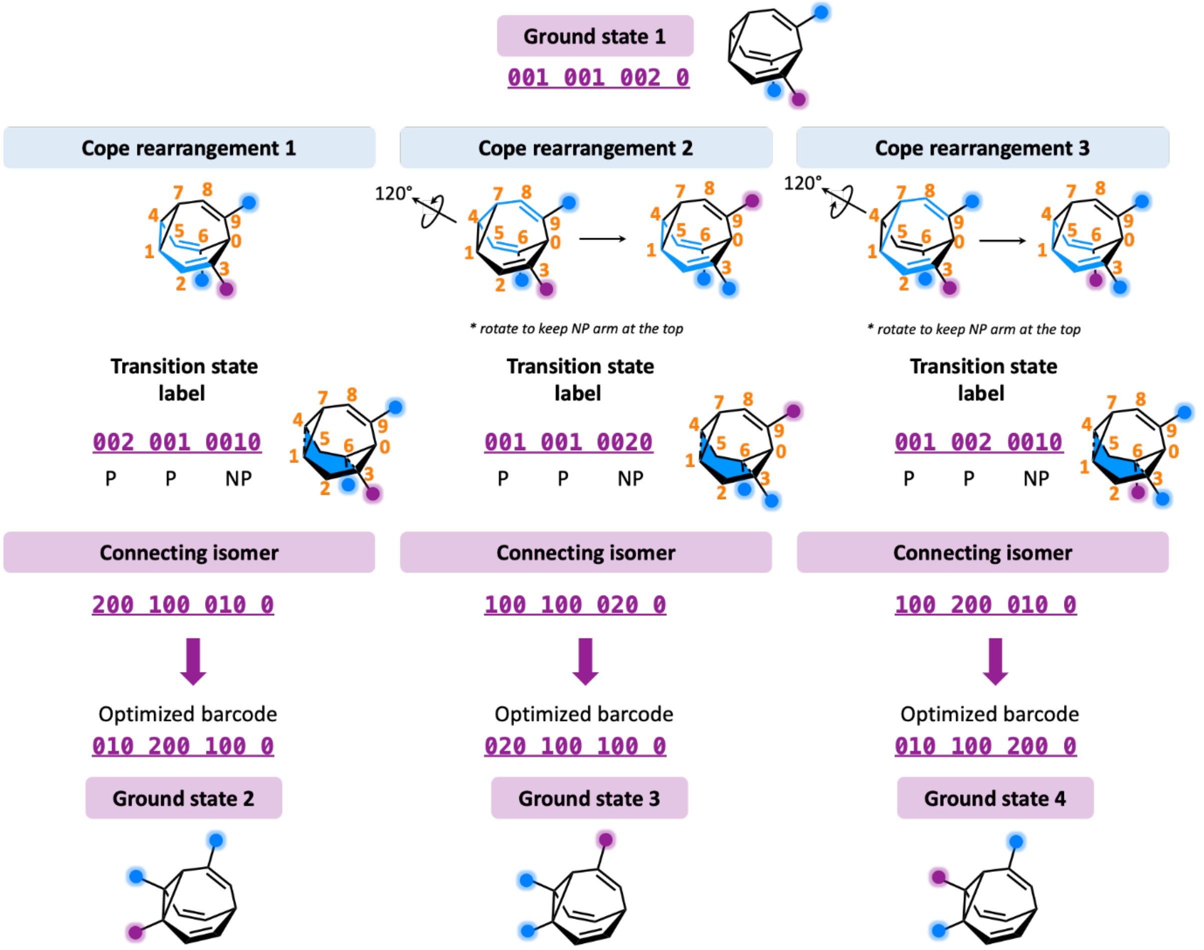

Scheme S1: Example of how transition state labels and resulting isomer were generated for **ββ'β"** (0010010020) ground state isomer. NP = non-participating bullvalene arm, P = participating bullvalene arm.

Python Script:

```
################################################################################################
######################
################################################################################################
######################
from itertools import permutations
import pandas as pd
import re
import os
import math

user_action = input('Type the BV substitution pattern(e.g., 1120000000):')

GS_energy_file = input('Enter the directory of the DFT .log files (ground states):')
TS_energy_file = input('Enter the directory of the DFT .log files (transition states):')

node_file = input('Enter a filename of a file (.csv) where the NODE data will be saved: ')
edge_file = input('Enter a filename of a file (.csv) where the EDGE data will be saved: ')

############################################### DEFINE FUNCTIONS
#####################################################
################################################################################################
######################

def optimise_barcode_permutation(BV_barcode):
  #This function takes a barcode (as a [0,0,0,0,0,0,0,0,0,0]) and optimises it to return the lowest permutation

  #Split the barcode into three arms and apical position
    arm1 = BV_barcode[0:3]
    arm2 = BV_barcode[3:6]
    arm3 = BV_barcode[6:9]
    methineC = BV_barcode[-1]

  #Define three possible barcodes for each isomer:
  #(arm1,arm2,arm3), (arm3, arm1,arm2) and (arm2, arm3,arm1)

    rotation_1 = []
    rotation_1.extend(arm1)
    rotation_1.extend(arm2)
    rotation_1.extend(arm3)
    rotation_1.append(methineC)
    rotation_1 = [str(i) for i in rotation_1]
    rotation_1_number = int(''.join(rotation_1))
    rotation_1 = ''.join(rotation_1)

    rotation_2 = []
```

```
        rotation_2.extend(arm3)
        rotation_2.extend(arm1)
        rotation_2.extend(arm2)
        rotation_2.append(methineC)
        rotation_2 = [str(i) for i in rotation_2]
        rotation_2_number = int(''.join(rotation_2))
        rotation_2 = ''.join(rotation_2)

        rotation_3 = []
        rotation_3.extend(arm2)
        rotation_3.extend(arm3)
        rotation_3.extend(arm1)
        rotation_3.append(methineC)
        rotation_3 = [str(i) for i in rotation_3]
        rotation_3_number = int(''.join(rotation_3))
        rotation_3 = ''.join(rotation_3)

        list_of_all_rotations = [rotation_1_number, rotation_2_number, rotation_3_number]
        list_of_all_rotations.sort()
        correct_rotation = list_of_all_rotations[0]

        #Append only correct isomers into a new dictionary
        if rotation_1_number == correct_rotation:
            return rotation_1
        elif rotation_2_number == correct_rotation:
            return rotation_2
        else:
            return rotation_3
def determine_chirality (BV_barcode):

    #Split the barcode into three arms and apical position
        arm1 = BV_barcode[0:3]
        arm2 = BV_barcode[3:6]
        arm3 = BV_barcode[6:9]

    #Bullvalene isomer is chiral if three arms are not equivalent

        if arm1 != arm2 and arm1 != arm3 and arm2 != arm3:
            return 'yes'
        else:
            return 'no'
def find_enantiomer (BV_barcode):
     #Split the barcode into three arms and apical position
        arm1 = BV_barcode[0:3]
        arm2 = BV_barcode[3:6]
        arm3 = BV_barcode[6:9]
        methineC = BV_barcode[-1]
```

```
  #Enantimer of any barcode (e.g., (arm1, arm2, arm3)) will have two arms swaped in its barcode (arm1, arm3, arm2)
    enantiomer = []
    enantiomer.extend(arm1)
    enantiomer.extend(arm3)
    enantiomer.extend(arm2)
    enantiomer.append(methineC)
    enantiomer = ''.join([str(e) for e in enantiomer])

    return enantiomer
def get_DFT_energies(directory):
  all_energies = pd.DataFrame (columns = ['id', 'Completed', 'Negative frequencies','Energy (Hartree)'])
  for root, dirs, files in os.walk(directory):
    for file in files:
      if file.endswith(('.log', '.com')):
        with open(os.path.join(root, file), 'r') as f:
          lines = f.readlines()
          completed = 'Normal termination of Gaussian' in lines[-1]
          gibbs_energy = next((float(line.split('=')[-1]) for line in lines if 'Sum of electronic and thermal Free Energies=' in
line), None)
          negative_freqs = 0
          barcode = file[:10]

        #Check if any negative frequencies are present in the .log file
          for line in lines:
            if line.startswith(' Frequencies --'):
              freqs = re.findall(r'-?\d+\.\d+', line)
              negative_freqs += sum(1 for freq in freqs if float(freq) < 0)

        #Get the xyz coordinates of successful ground state calculations
          if negative_freqs == 0 and completed == True:

            results_line = (next((str(line) for line in lines if ' Optimization completed.' in line or ' Optimization completed on
the basis of negligible forces.' in line), None))
            results_line_index = lines.index(results_line)
            xyz_start = [line for line in lines if '                    Standard orientation:' in line]

            xyz_start_index = [lines.index(i, results_line_index) for i in xyz_start][0]

            index_1 = xyz_start_index + 5
            index_2 = index_1 + 34

            xyz_matrix = lines[index_1:index_2]

            atom_number_list = []
            atom_type_list = []
            X_val = []
```

```
                Y_val = []
                Z_val = []

                for xyz_line in xyz_matrix:

                  atom_number = int(str(xyz_line[0:8]).strip())
                  atom_number_list.append(atom_number)

                  atom_type = str(xyz_line[8:19]).strip()
                  atom_type_list.append(atom_type)

                  X = float(str(xyz_line[37:47]).strip())
                  X_val.append(X)

                  Y = float(str(xyz_line[49:59]).strip())
                  Y_val.append(Y)

                  Z = float(str(xyz_line[61:71]).strip())
                  Z_val.append(Z)

                xyz_coordinates = pd.DataFrame(columns = ['Atomic number', 'Atomic type', 'X', 'Y', 'Z'])
                xyz_coordinates['Atomic number'] = pd.Series(atom_number_list)
                xyz_coordinates['Atomic type'] = pd.Series(atom_type_list)
                xyz_coordinates['X'] = pd.Series(X_val)
                xyz_coordinates['Y'] = pd.Series(Y_val)
                xyz_coordinates['Z'] = pd.Series(Z_val)

                #Save all xyz coordinated of successful DFT calcualtons to 'XYZ Coordinates From Successful DFT
Calcualtions Golder.csv' file
                with open("XYZ Coordinates From Successful DFT Calcualtions.csv", "a") as f:
                  f.writelines(f'Coordinates (Angstrom) for {barcode} ground state \n\n')

                xyz_coordinates.to_csv("XYZ Coordinates From Successful DFT Calcualtions.csv", mode = 'a', index=False,
header=True)

                with open("XYZ Coordinates From Successful DFT Calcualtions.csv", "a") as f:
                  f.writelines('\n\n')

            all_energies.at[len(all_energies),'id'] = barcode
            all_energies.at[len(all_energies)-1,'Completed'] = completed
            all_energies.at[len(all_energies)-1,'Energy (Hartree)'] = gibbs_energy
            all_energies.at[len(all_energies)-1, 'Negative frequencies'] = negative_freqs
            all_energies.to_csv('Processed_raw_GS_data.csv')
            all_energies['Energy (Hartree)'] = all_energies.groupby('id')['Energy (Hartree)'].transform('min')  #Keeps only the
lowest energy conformer form each isomer of the ground state

  all_energies = all_energies.drop_duplicates(subset=['id'])
```

```
    all_energies['Energy (kJ/mol)'] = all_energies['Energy (Hartree)'] * 2600

    return all_energies
def get_TS_DFT_energies(directory):
    all_energies = pd.DataFrame (columns = ['TS label', 'Completed', 'Wavenumber','Negative frequencies','TS Energy
(Hartree)', 'Cope?'])
    for root, dirs, files in os.walk(directory):
        for file in files:
            if file.endswith(('.log', '.com')):
                barcode = file[:10]
                with open(os.path.join(root, file), 'r') as f:
                    lines = f.readlines()
                    negative_freqs = 0
                    completed = 'FALSE'
                    negative_freq_wavenumber = pd.NaT
                    gibbs_energy = pd.NaT

                    #Checks how many negative frequecies are present and that the calcualtion terminated successfully
                    for line in lines:
                        if line.startswith(' Frequencies --'):
                            freqs = re.findall(r'-?\d+\.\d+', line)
                            negative_freqs += sum(1 for freq in freqs if float(freq) < 0)
                        if line.startswith(' Normal termination of Gaussian'):
                            completed = 'TRUE'

                    if completed == 'TRUE':

                        gibbs_energy = next((float(line.split('=')[-1]) for line in lines if 'Sum of electronic and thermal Free Energies=' in
line), None)

                        #Get information about the negative frequncy mode to check which atoms had the largest diplacents and
filter out substituent rotations from Cope rearangements TS
                        low_freq_wavenumbers = [next(((str(line.split('---')[-1])[1:10]) for line in lines if 'Low frequencies ---' in line),
None)]
                        low_freq_wavenumbers.sort()
                        negative_freq_wavenumber = str(low_freq_wavenumbers[0])
                        negative_freq_wavenumber_2 = negative_freq_wavenumber[:-3]

                        negative_freq_mode = next((str(line) for line in lines if f'Frequencies --   {negative_freq_wavenumber_2}' in
line), None)
                        lines = list(lines)

                        negative_freq_mode_line_index = lines.index(negative_freq_mode)
                        negative_freq_coordinates_lineindex1 = negative_freq_mode_line_index + 5
                        negative_freq_coordinates_lineindex2 = negative_freq_mode_line_index + 39

                        xyz_coordinates_lines = lines[negative_freq_coordinates_lineindex1:negative_freq_coordinates_lineindex2]
```

```
coordinates = pd.DataFrame(columns = ['Atom_type', 'X', 'Y', 'Z', 'Distance moved'])
atom_type = []
X_values = []
Y_values = []
Z_values = []
distance_moved_values = []

for c in xyz_coordinates_lines:
  atom = str(c[9])
  atom = int(str(c[9]))
  atom_type.append(atom)

  X = float(str(c[14:21]))
  X_values.append(X)

  Y = float(str(c[22:27]))
  Y_values.append(Y)

  Z = float(str(c[29:34]))
  Z_values.append(Z)

  distance_moved = math.sqrt(X**2 + Y**2 + Z**2)
  distance_moved_values.append(distance_moved)

coordinates['Atom_type'] = pd.Series(atom_type)
coordinates['X'] = pd.Series(X_values)
coordinates['Y'] = pd.Series(Y_values)
coordinates['Z'] = pd.Series(Z_values)
coordinates['Distance moved'] = pd.Series(distance_moved_values)

maximum_values = set(coordinates.loc[:,['Distance moved']].idxmax())

check_list = set([0, 3, 2, 5, 17, 18, 19, 20, 21, 22, 23])

coordinates_C = coordinates.drop(coordinates[coordinates['Atom_type'] != 6].index)

maximum_values_C = set(coordinates_C.loc[:,['Distance moved']].idxmax())

check_list_C = set([0, 3, 2, 5])

all_atom_displacemnts = (maximum_values.issubset(check_list))
correct_C_displacement = (maximum_values_C.issubset(check_list_C))

#Extract optimised coordinates for TS geometries
```

```
        results_line = (next((str(line) for line in lines if ' Optimization completed.' or ' Optimization completed on the basis of negligible forces.' in line), None))
        results_line_index = lines.index(results_line)
        xyz_start = [line for line in lines if '                    Standard orientation:' in line]
        xyz_start_index = [lines.index(i, results_line_index) for i in xyz_start][0]

        index_1 = xyz_start_index + 5
        index_2 = index_1 + 34

        xyz_matrix = lines[index_1:index_2]

        atom_number_list = []
        atom_type_list = []
        X_val = []
        Y_val = []
        Z_val = []

        for xyz_line in xyz_matrix:

          atom_number = int(str(xyz_line[0:8]).strip())
          atom_number_list.append(atom_number)

          atom_type = str(xyz_line[8:19]).strip()
          atom_type_list.append(atom_type)

          X = float(str(xyz_line[37:47]).strip())
          X_val.append(X)

          Y = float(str(xyz_line[49:59]).strip())
          Y_val.append(Y)

          Z = float(str(xyz_line[61:71]).strip())
          Z_val.append(Z)

        xyz_coordinates = pd.DataFrame(columns = ['Atomic number', 'Atomic type', 'X', 'Y', 'Z'])
        xyz_coordinates['Atomic number'] = pd.Series(atom_number_list)
        xyz_coordinates['Atomic type'] = pd.Series(atom_type_list)
        xyz_coordinates['X'] = pd.Series(X_val)
        xyz_coordinates['Y'] = pd.Series(Y_val)
        xyz_coordinates['Z'] = pd.Series(Z_val)

        xyz_coordinates.index = xyz_coordinates.index + 1

        if all_atom_displacemnts == True and correct_C_displacement == True:
          Cope = all_atom_displacemnts

        else:
```

```
                Cope = False

            if Cope == True:
                with open("XYZ Coordinates From Successful TS DFT Calcualtions.csv", "a") as f:
                    f.writelines(f'Coordinates (Angstrom) for {barcode} transition state \n\n')

                xyz_coordinates.to_csv("XYZ Coordinates From Successful TS DFT Calcualtions.csv", mode = 'a',
index=False, header=True)

                with open("XYZ Coordinates From Successful TS DFT Calcualtions.csv", "a") as f:
                    f.writelines('\n\n')

        else:
            Cope = pd.NaT

        barcode = file[:10]
        all_energies.at[len(all_energies),'TS label'] = barcode
        all_energies.at[len(all_energies)-1,'Completed'] = completed
        all_energies.at[len(all_energies)-1,'Wavenumber'] = negative_freq_wavenumber
        all_energies.at[len(all_energies)-1,'TS Energy (Hartree)'] = gibbs_energy
        all_energies.at[len(all_energies)-1, 'Negative frequencies'] = negative_freqs
        all_energies.at[len(all_energies)-1, 'Cope?'] = Cope

  all_energies.to_csv('Processed_raw_TS_data.csv')
  all_energies = all_energies.dropna()

  all_energies.drop(all_energies[all_energies['Cope?'] == False].index, inplace=True)
  all_energies['TS Energy (Hartree)'] = all_energies.groupby('TS label')['TS Energy (Hartree)'].transform('min') #Keeps only
the lowest energy conformer form each isomer of the transtion state

  all_energies = all_energies.drop_duplicates(subset=['TS label'])
  all_energies['TS Energy (kJ/mol)'] = all_energies['TS Energy (Hartree)'] * 2600
  all_energies = all_energies.drop('Wavenumber', axis=1)
  all_energies = all_energies.reset_index(drop=True)

  return all_energies
def determine_chirality_TS (BV_barcode):
  #Determines if a TS is chiral

    arm1 = BV_barcode[0:3]
    arm2 = BV_barcode[3:6]

    if arm1 != arm2:
      return 'yes'
    else:
      return 'no'
```

```
################################################ GENERATE GROUND STATES
################################################
#################################################################################################
######################

barcode = [int(digit) for digit in user_action]
nodes_info = pd.DataFrame(columns = ['id','chiral?','enantiomer'])

GS_energies = get_DFT_energies(GS_energy_file)

if len(barcode) != 10:
    print('Check the input and try again!')

else:
    perm = permutations(barcode)
    unique_permutations = list(set(perm)) #removes dublicates arising from permuatations

    for raw_barcode in unique_permutations:
        row_index = unique_permutations.index(raw_barcode)

        optimised_barcode = optimise_barcode_permutation(raw_barcode)
        nodes_info.at[row_index,'id'] = optimised_barcode

        barcode_chirality = determine_chirality (raw_barcode)
        nodes_info.at[row_index,'chiral?'] = barcode_chirality

        if barcode_chirality == 'yes':
            enantiomer = optimise_barcode_permutation(find_enantiomer(raw_barcode))
            nodes_info.at[row_index,'enantiomer'] = enantiomer
        else:
            nodes_info.at[row_index,'enantiomer'] = pd.NA

nodes_info = nodes_info.drop_duplicates(subset=['id'], ignore_index=True)
nodes_info=pd.merge(nodes_info, GS_energies, how = 'left', on = ['id'])

lowest_energy = nodes_info['Energy (kJ/mol)'].min()
nodes_info['Relative Energy (kJ/mol)'] = nodes_info['Energy (kJ/mol)'] - lowest_energy

for i, row in nodes_info.iterrows():
    isomer = row['id']
    isomer_energy = row['Relative Energy (kJ/mol)']

    chiral = row['chiral?']

    #If bullvalne is chiral the energy of two enantiomers is set to the lowest energy of the two
    if chiral == 'yes':
        enantiomer = row['enantiomer']
```

```
        enantiomer_index = nodes_info.isin([enantiomer]).any(axis=1).idxmax()
        enantiomer_energy = nodes_info.loc[enantiomer_index,['Relative Energy (kJ/mol)']].iloc[0]

        if isomer_energy <= enantiomer_energy:
            nodes_info.loc[enantiomer_index,'Relative Energy (kJ/mol)'] = isomer_energy

        elif isomer_energy > enantiomer_energy:
            nodes_info.loc[i, 'Relative Energy (kJ/mol)'] = enantiomer_energy

#Save as a csv file
nodes_info.to_csv(f'{node_file}')

################################################## GENERATE TRANSITION STATES
##############################################
####################################################################################################
######################

edge_info = pd.DataFrame(columns = ['source','target','TS label','TS chiral?'])
TS_energies = get_TS_DFT_energies(TS_energy_file)

for i, row in nodes_info.iterrows():
    barcode = row['id']
    c1 = [barcode[0]]
    c2 = [barcode[1]]
    c3 = [barcode[2]]
    c4 = [barcode[3]]
    c5 = [barcode[4]]
    c6 = [barcode[5]]
    c7 = [barcode[6]]
    c8 = [barcode[7]]
    c9 = [barcode[8]]
    c0 = [barcode[9]]

    #Determine which atoms change their possitions after each of three possible Cope rearrangements and link two
ground state label with the respective TS label for this Cope rearrangment
    #For each TS check if the reverse TS labelling gives lower number

    #Define the transition state for the 1st permutation
    transition_state_1 = []
    transition_state_1_forward_barcode = c1 + c2 + c3 + c4 + c5 + c6 + c7 + c8 + c9 + c0
    transition_state_1_reverse_barcode = c6 + c5 + c4 + c3 + c2 + c1 + c0 + c9 + c8 + c7
    if int(''.join([str(o) for o in transition_state_1_forward_barcode])) < int(''.join([str(o1) for o1 in
transition_state_1_reverse_barcode])):
        transition_state_1 = ''.join([str(i) for i in transition_state_1_forward_barcode])
    else:
        transition_state_1 = ''.join([str(i) for i in transition_state_1_reverse_barcode])
```

```
    connection_1 = []
    connection_1 = c0 + c9 + c8 + c6 + c5 + c4 + c3 + c2 + c1 +c7
    connection_1 = optimise_barcode_permutation(connection_1)

    edge_info.at[len(edge_info),'source'] = barcode
    edge_info.at[len(edge_info)-1,'target'] = connection_1
    edge_info.at[len(edge_info)-1,'TS label'] = transition_state_1
    edge_info.at[len(edge_info)-1,'TS chiral?'] = determine_chirality_TS(transition_state_1)


    #Define the transition state for the 2nd permutation
    transition_state_2 = []
    transition_state_2_forward_barcode = c4 + c5 + c6 + c7 + c8 + c9 + c1 + c2 + c3 + c0
    transition_state_2_reverse_barcode = c9 + c8 + c7 + c6 + c5 + c4 + c0 + c3 + c2 + c1
    if int(''.join([str(o2) for o2 in transition_state_2_forward_barcode])) < int(''.join([str(o3) for o3 in
transition_state_2_reverse_barcode])):
        transition_state_2 = ''.join([str(i) for i in transition_state_2_forward_barcode])
    else:
        transition_state_2 = ''.join([str(i) for i in transition_state_2_reverse_barcode])

    connection_2 = []
    connection_2 = c9 + c8 + c7 + c6 + c5 + c4 + c0 + c3 + c2 +c1
    connection_2 = optimise_barcode_permutation(connection_2)

    edge_info.at[len(edge_info),'source'] = barcode
    edge_info.at[len(edge_info)-1,'target'] = connection_2
    edge_info.at[len(edge_info)-1,'TS label'] = transition_state_2
    edge_info.at[len(edge_info)-1,'TS chiral?'] = determine_chirality_TS(transition_state_2)

    #Define the transition state for the 3rd permutation
    transition_state_3 = []
    transition_state_3_forward_barcode = c7 + c8 + c9 + c1 + c2 + c3 + c4 + c5 + c6 + c0
    transition_state_3_reverse_barcode = c3 + c2 + c1 + c9 + c8 + c7 + c0 + c6 + c5 + c4
    if int(''.join([str(o4) for o4 in transition_state_3_forward_barcode])) < int(''.join([str(o5) for o5 in
transition_state_3_reverse_barcode])):
        transition_state_3 = ''.join([str(i) for i in transition_state_3_forward_barcode])
    else:
        transition_state_3 = ''.join([str(i) for i in transition_state_3_reverse_barcode])

    connection_3 = []
    connection_3 = c9 + c8 + c7 + c0 + c6 + c5 + c3 + c2 + c1 +c4
    connection_3 = optimise_barcode_permutation(connection_3)

    edge_info.at[len(edge_info),'source'] = barcode
    edge_info.at[len(edge_info)-1,'target'] = connection_3
    edge_info.at[len(edge_info)-1,'TS label'] = transition_state_3
    edge_info.at[len(edge_info)-1,'TS chiral?'] = determine_chirality_TS(transition_state_3)
```

```
edge_info=pd.merge(edge_info, TS_energies, how = 'left', on = ['TS label'])
edge_info['Relative TS Energy (kJ/mol)'] = edge_info['TS Energy (kJ/mol)'] - lowest_energy

for i, row in edge_info.iterrows():
   ground_state_id = row['source']
   ground_state_index = nodes_info.isin([ground_state_id]).any(axis=1).idxmax()
   edge_info.loc[i, 'Energy barrier (kJ/mol)'] = row['Relative TS Energy (kJ/mol)'] -
nodes_info.loc[ground_state_index,['Relative Energy (kJ/mol)']].iloc[0]

user_T = input('T for the rate constant calculation (K):')
edge_info[f'Rate constant at {user_T} K'] = (1.380649*(10**-23)*(float(user_T))/(6.62607015*(10**-34)))*(math.e**(-
(edge_info['Energy barrier (kJ/mol)'])*1000/(float(user_T)*8.314462618)))

#Save as a csv file
edge_info.to_csv(f'{edge_file}')
```

6.0: REFERENCES

(1) Berkowski, K. L.; Potisek, S. L.; Hickenboth, C. R.; Moore, J. S. Ultrasound-Induced Site-Specific Cleavage of Azo-Functionalized Poly(Ethylene Glycol). *Macromolecules* **2005**, *38* (22), 8975–8978. https://doi.org/10.1021/ma051394n.
(2) MestreLab. EXSYCalc. https://mestrelab.com/main-product/freeware (accessed 2025-12-18).
(3) Ayub, R.; Papadakis, R.; Jorner, K.; Zietz, B.; Ottosson, H. Cyclopropyl Group: An Excited-State Aromaticity Indicator? *Chem. – Eur. J.* **2017**, *23* (55), 13684–13695. https://doi.org/10.1002/chem.201701404.
(4) Sun, P. B.; Pomfret, M. N.; Elardo, M. J.; Suresh, A.; Rentería-Gómez, Á.; Lalisse, R. F.; Keating, S.; Chen, C.; Hilburg, S. L.; Chakma, P.; Wu, Y.; Bell, R. C.; Rowan, S. J.; Gutierrez, O.; Golder, M. R. Molecular Ball Joints: Mechanochemical Perturbation of Bullvalene Hardy–Cope Rearrangements in Polymer Networks. *J. Am. Chem. Soc.* **2024**, *146* (28), 19229–19238. https://doi.org/10.1021/jacs.4c04401.
(5) Sommer, R.; Neres, J.; Piton, J.; Dhar, N.; Van Der Sar, A.; Mukherjee, R.; Laroche, T.; Dyson, P. J.; McKinney, J. D.; Bitter, W.; Makarov, V.; Cole, S. T. Fluorescent Benzothiazinone Analogues Efficiently and Selectively Label Dpre1 in Mycobacteria and Actinobacteria. *ACS Chem. Biol.* **2018**, *13* (11), 3184–3192. https://doi.org/10.1021/acschembio.8b00790.
(6) Osler, S. K.; McFadden, M. E.; Zeng, T.; Robb, M. J. Mechanochemical Reactivity of a Multimodal 2H-Bis-Naphthopyran Mechanophore. *Polym. Chem.* **2023**, *14* (22), 2717–2723. https://doi.org/10.1039/d3py00344b.
(7) Poupko, R.; Zimmermann, H.; Müller, K.; Luz, Z. Dynamic NMR Investigation of the Cope Rearrangement in Solutions of Monosubstituted Bullvalenes. *J. Am. Chem. Soc.* **1996**, *118* (34), 7995–8005. https://doi.org/10.1021/ja954004t.
(8) Myers, A. G.; Zheng, B.; Movassaghi, M. Preparation of the Reagent O-Nitrobenzenesulfonylhydrazide. *J. Org. Chem.* **1997**, *62* (21), 7507–7507. https://doi.org/10.1021/jo9710137.
(9) Bloom, S.; Knippel, J. L.; Holl, M. G.; Barber, R.; Lectka, T. A Cooperative Allylic Fluorination: Combination of Nucleophilic and Electrophilic Fluorine Sources. *Tetrahedron Lett.* **2014**, *55* (33), 4576–4580. https://doi.org/10.1016/j.tetlet.2014.05.093.
(10) Vasilopoulos, A.; Golden, D. L.; Buss, J. A.; Stahl, S. S. Copper-Catalyzed C–H Fluorination/Functionalization Sequence Enabling Benzylic C–H Cross Coupling with Diverse Nucleophiles. *Org. Lett.* **2020**, *22* (15), 5753–5757. https://doi.org/10.1021/acs.orglett.0c02238.
(11) Haufe, G.; Alvernhe, G.; Anker, D.; Laurent, A.; Saluzzo, C. Synthesis of Fluoroalkyl Methyl Thioethers by Formal Addition of Methanesulfenyl Fluoride to Alkenes. *J. Org. Chem.* **1992**, *57* (2), 714–719. https://doi.org/10.1021/jo00028a056.
(12) L'Heureux, A.; Beaulieu, F.; Bennett, C.; Bill, D. R.; Clayton, S.; LaFlamme, F.; Mirmehrabi, M.; Tadayon, S.; Tovell, D.; Couturier, M. Aminodifluorosulfinium Salts: Selective Fluorination Reagents with Enhanced Thermal Stability and Ease of Handling,. *J. Org. Chem.* **2010**, *75* (10), 3401–3411. https://doi.org/10.1021/jo100504x.
(13) Tong, H.; Bell, D.; Tabei, K.; Siegel, M. M. Automated Data Massaging, Interpretation, and e-Mailing Modules for High Throughput Open Access Mass Spectrometry. *J. Am. Soc. Mass Spectrom.* **1999**, *10* (11), 1174–1187. https://doi.org/10.1016/S1044-0305(99)00090-2.
(14) Bullviso, 2024. https://gitlab.com/conorrankine/bullviso (accessed 2026-01-30).
(15) Riniker, S.; Landrum, G. A. Better Informed Distance Geometry: Using What We Know To Improve Conformation Generation. *J. Chem. Inf. Model.* **2015**, *55* (12), 2562–2574. https://doi.org/10.1021/acs.jcim.5b00654.

(16) Wang, S.; Witek, J.; Landrum, G. A.; Riniker, S. Improving Conformer Generation for Small Rings and Macrocycles Based on Distance Geometry and Experimental Torsional-Angle Preferences. *J. Chem. Inf. Model.* **2020**, *60* (4), 2044–2058. https://doi.org/10.1021/acs.jcim.0c00025.
(17) *RDKit*. https://www.rdkit.org/ (accessed 2026-01-28).
(18) *RDKit*. RDKit. https://rdkit.org/ (accessed 2025-12-17).
(19) Bannwarth, C.; Ehlert, S.; Grimme, S. GFN2-xTB—An Accurate and Broadly Parametrized Self-Consistent Tight-Binding Quantum Chemical Method with Multipole Electrostatics and Density-Dependent Dispersion Contributions. *J. Chem. Theory Comput.* **2019**, *15* (3), 1652–1671. https://doi.org/10.1021/acs.jctc.8b01176.
(20) Grimme, S.; Bannwarth, C.; Shushkov, P. A Robust and Accurate Tight-Binding Quantum Chemical Method for Structures, Vibrational Frequencies, and Noncovalent Interactions of Large Molecular Systems Parametrized for All Spd-Block Elements (Z = 1–86). *J. Chem. Theory Comput.* **2017**, *13* (5), 1989–2009. https://doi.org/10.1021/acs.jctc.7b00118.
(21) Bannwarth, C.; Caldeweyher, E.; Ehlert, S.; Hansen, A.; Pracht, P.; Seibert, J.; Spicher, S.; Grimme, S. Extended Tight-binding Quantum Chemistry Methods. *WIREs Comput. Mol. Sci.* **2020**, *11*. https://doi.org/10.1002/wcms.1493.
(22) Yahiaoui, O.; Pašteka, L. F.; Blake, C. J.; Newton, C. G.; Fallon, T. Network Analysis of Substituted Bullvalenes. *Org. Lett.* **2019**, *21* (23), 9574–9578. https://doi.org/10.1021/acs.orglett.9b03737.
(23) Perdew, J. P.; Burke, K.; Ernzerhof, M. Generalized Gradient Approximation Made Simple. *Phys. Rev. Lett.* **1996**, *77* (18), 3865–3868. https://doi.org/10.1103/PhysRevLett.77.3865.
(24) Perdew, J. P.; Burke, K.; Ernzerhof, M. Generalized Gradient Approximation Made Simple [Phys. Rev. Lett. 77, 3865 (1996)]. *Phys. Rev. Lett.* **1997**, *78* (7), 1396–1396. https://doi.org/10.1103/PhysRevLett.78.1396.
(25) Adamo, C.; Barone, V. Toward Reliable Density Functional Methods without Adjustable Parameters: The PBE0 Model. *J. Chem. Phys.* **1999**, *110* (13), 6158–6170. https://doi.org/10.1063/1.478522.
(26) Grimme, S.; Antony, J.; Ehrlich, S.; Krieg, H. A Consistent and Accurate Ab Initio Parametrization of Density Functional Dispersion Correction (DFT-D) for the 94 Elements H-Pu. *J. Chem. Phys.* **2010**, *132* (15), 154104. https://doi.org/10.1063/1.3382344.
(27) Frisch, M. J.; Trucks, G. W.; Schlegel, H. B.; Scuseria, G. E.; Robb, M. A.; Cheeseman, J. R.; Scalmani, G.; Barone, V.; Peterson, G. A.; Nakatsuji, H.; Li, X.; Caricato, M.; Marenich, A. V.; Bloino, J.; Janesko, B. G.; Gomperts, R.; Mennucci, B.; Hratchian, H. P.; Ortiz, J. V.; Izmaylov, A. F.; Sonnenberg, J. L.; Williams-Young, D.; Ding, F.; Lipparini, F.; Egidi, F.; Goings, J.; Peng, B.; Petrone, A.; Henderson, T.; Ranasinghe, D.; Zakrzewski, V. G.; Gao, J.; Rega, N.; Zheng, G.; Liang, W.; Hada, M.; Ehara, M.; Toyota, K.; Fukuda, R.; Hasegawa, J.; Ishida, M.; Nakajima, T.; Honda, Y.; Kitao, O.; Nakai, H.; Vreven, T.; Throssell, K.; Montgomery Jr., J. A.; Peralta, J. E.; Ogliaro, F.; Bearpark, M. J.; Heyd, J. J.; Brothers, E. N.; Kudin, K. N.; Staroverov, V. N.; Keith, T. A.; Kobayashi, R.; Normand, J.; Rafhavachari, K.; Rendell, A. P.; Burant, J. C.; Iyengar, S. S.; Tomasi, J.; Cossi, M.; Millam, J. M.; Klene, M.; Adamo, C.; Cammi, R.; Ochterski; Martin, R. L.; Morokuma, K.; Farkas, O.; Forseman, J. B. Gaussian 16, 2016.
(28) Weigend, F.; Ahlrichs, R. Balanced Basis Sets of Split Valence, Triple Zeta Valence and Quadruple Zeta Valence Quality for H to Rn: Design and Assessment of Accuracy. *Phys. Chem. Chem. Phys.* **2005**, *7* (18), 3297–3305. https://doi.org/10.1039/B508541A.
(29) Cytoscape. https://cytoscape.org. Accessed January 2026
(30) Otasek, D.; Morris, J. H.; Bouças, J.; Pico, A. R.; Demchak, B. Cytoscape Automation: Empowering Workflow-Based Network Analysis. *Genome Biol.* **2019**, *20* (1), 185. https://doi.org/10.1186/s13059-019-1758-4.

(31) Perdew, J. P.; Burke, K.; Ernzerhof, M. Generalized Gradient Approximation Made Simple [Phys. Rev. Lett. 77, 3865 (1996)]. *Phys. Rev. Lett.* **1997**, *78* (7), 1396–1396. https://doi.org/10.1103/PhysRevLett.78.1396.
(32) Hinsberg, W.; Houle, F. Kinetiscope. https://hinsberg.net/kinetiscope/index.html (accessed 2026-01-30).
(33) He, M.; Bode, J. W. E Pluribus Unum: Isolation, Structure Determination, Network Analysis and DFT Studies of a Single Metastable Structure from a Shapeshifting Mixture of 852 Bullvalene Structural Isomers. *Org Biomol Chem* **2013**, *11* (8), 1306–1317. https://doi.org/10.1039/C2OB26954F.
(34) Yahiaoui, O.; Pašteka, L. F.; Judeel, B.; Fallon, T. Synthesis and Analysis of Substituted Bullvalenes. *Angew. Chem. - Int. Ed.* **2018**, *57* (10), 2570–2574. https://doi.org/10.1002/ANIE.201712157.
(35) Ives, R. A.; Maturi, W.; Gill, M. T.; Rankine, C.; McGonigal, P. R. A Guide to Bullvalene Stereodynamics. *Chem. Sci.* **2024**, *15* (36), 14608–14617. https://doi.org/10.1039/d4sc03700f.
